# Towards Novel Antiplasmodial Agents—Design, Synthesis and Antimalarial Activity of Second-Generation β-Carboline/Chloroquine Hybrids

**DOI:** 10.3390/molecules29245991

**Published:** 2024-12-19

**Authors:** Ana Penava, Marina Marinović, Lais Pessanha de Carvalho, Jana Held, Ivo Piantanida, Dijana Pavlović Saftić, Zrinka Rajić, Ivana Perković

**Affiliations:** 1 Faculty of Pharmacy and Biochemistry, University of Zagreb, A. Kovačića 1, 10000 Zagreb, Croatia; ana.penava@pharma.unizg.hr (A.P.); marina.marinovic@pharma.unizg.hr (M.M.); 2 Institute of Tropical Medicine, University of Tübingen, Wilhelmstraße 27, 72074 Tübingen, Germany; lais.pessanha-de-carvalho@uni-tuebingen.de (L.P.d.C.); jana.held@uni-tuebingen.de (J.H.); 3 Partner Site Tübingen, German Center for Infection Research (DZIF), 72074 Tübingen, Germany; 4 Rudjer Bošković Institute, Bijenička cesta 54, 10000 Zagreb, Croatia; ivo.piantanida@irb.hr (I.P.); dijana.pavlovic.saftic@irb.hr (D.P.S.)

**Keywords:** β-carboline, chloroquine, hybrid, synthesis, malaria, fluorimetric probes

## Abstract

As the resistance of *Plasmodium* to the existing antimalarials increases, there is a crucial need to expand the antimalarial drug pipeline. We recently identified potent antimalarial compounds, namely harmiquins, hybrids derived from the β-carboline alkaloid harmine and 4-amino-7-chloroquinoline, a key structural motif of chloroquine (CQ). To further explore the structure−activity relationship, we synthesised 13 novel hybrid compounds at the position *N*-9 of the β-carboline ring and evaluated their efficacy in vitro against *Plasmodium falciparum* 3D7 and Dd2 strains (CQ sensitive and multi-drug resistant, respectively). All compounds exhibit persistent antimalarial activity against both strains of *P. falciparum*. The most interesting derivatives had low nanomolar activity against both strains (IC_50_ (**33**) = 4.7 ± 1.3 nM against *Pf*3D7 and 6.5 ± 2.5 nM against *Pf*Dd2; IC_50_ (**37**) = 4.6 ± 0.6 nM against 3D7 and 10.5 ± 0.4 nM against Dd2). Resistance indices (RIs) ranged from 0.9 to 5.3 compared to CQ (RI = 14.4), highlighting their superior consistency in activity against both strains. The cytotoxicity screening performed on HepG2 revealed over 3 orders of magnitude higher IC_50_ for most of the compounds, with SIs from 711.0 to 8081.8. Spectroscopic studies explored the affinities of newly synthesised compounds for DNA, RNA, and HSA. Both tested hybrids, **34** and **39**, were intrinsically fluorescent in an aqueous medium, characterised by remarkable Stokes shifts of emission maxima (Δ*λ* = +103 and +93 nm for **34** and **39**, respectively). Fluorimetric experiments revealed that compound **34**, with its shorter and more flexible linker, exhibited at least an order of magnitude higher affinity toward ds-DNAs versus ds-RNA and two orders of magnitude higher affinity toward GC-DNAs compared to **39**. The behaviour of the investigated compounds upon binding to HSA is very similar, showing a strong hypsochromic shift of the emission maximum (almost Δ*λ* = −70 nm) and demonstrating their effectiveness as fluorimetric probes for distinguishing between DNA/RNA and proteins.

## 1. Introduction

Malaria, a parasitic disease prevalent in tropical and subtropical regions, is caused by various *Plasmodium* species and is transmitted through the bite of infected female *Anopheles* mosquitoes. While six species of the *Plasmodium* parasite are known to infect humans, *Plasmodium falciparum* stands out for its capacity to produce substantial levels of blood-stage parasites, contributing significantly to malaria-related fatalities, with African children under 5 years of age being the most vulnerable demographic group [1].

Despite the considerable progress in malaria control due to efforts made since 2000, a steady stagnation has been noted in the last decade. In 2022, a global estimate suggests there were approximately 249 million malaria cases worldwide, marking a rise of 5 million cases from the previous year. In 2015, which serves as the baseline year for the Global Technical Strategy for Malaria 2016–2030 (GTS), an estimated 231 million malaria cases were reported [2]. The rise in malaria-related deaths in 2022, totalling 608,000 fatalities, from which more than 75% account for children under the age of 5, additionally underscores the persistent burden malaria imposes on affected populations. The impact of climate change is increasingly being acknowledged as a significant factor contributing to the distribution and transmission dynamics of malaria, together with disruptions in malaria prevention and treatment programs and overloaded healthcare systems during the COVID-19 pandemic [2,3,4]. *Plasmodium* resistance to antimalarials and mosquito resistance to insecticides are constantly on the rise. As current antimalarial treatment has liabilities and both emerging and well-documented resistance issues, there is a continued need to refill the development pipeline with new and improved candidates [5]. Intensive efforts in antimalarial research have resulted in the identification of numerous active compounds, which are currently progressing through various stages of drug development, ranging from preclinical studies to clinical trials. Our particular interests lie in the compounds derived from chloroquine (CQ), a 4-amino-7-chloroquinoline antimalarial drug, and β-carboline, due to their significant antimalarial activities.

CQ has been a prominent antimalarial drug in use since the 1940s. However, similar to many other antimalarials, its effectiveness in therapy has been significantly corrupted by the emergence of resistant *Plasmodium* parasites [6,7]. Derivatisation of its quinoline core, which has been confirmed as crucial for antimalarial activity, with various scaffolds offers a strategy to combat resistance, as resistance appears to be specific to the chemical structure of CQ [8]. Thus, various attempts have been undertaken to address the resistance issues with CQ and enhance its antimalarial efficacy by linking a wide range of structural motifs to the 4-amino-7-chloroquine core [7]. Frequently, derivatisation of the 4-amino-7-chloroquine core yields compounds exhibiting notable in vitro activity against both sensitive and resistant *P. falciparum* strains, with IC_50_ values ranging from micromolar to low nanomolar levels, depending on the scaffold linked to the 4-amino-7-chloroquinoline. Additionally, certain compounds have demonstrated superior activity compared to CQ [7,9,10,11,12,13,14,15,16,17,18,19].

β-Carbolines are a group of diverse, both naturally derived and synthetic compounds that have been studied for their CNS-related, anti-inflammatory, antioxidant, and anticancer properties, highlighting their multifaceted biological activities [19,20]. The antimalarial activity of harmine, one of the most extensively investigated β-carboline that was first isolated from *Peganum harmala* seeds, has been well documented and has paved the way for the extensive research of synthetic and natural β-carbolines as potential antimalarial agents delivering not only novelty in chemical structures but also in mechanism of antimalarial activity [21,22,23]. Derivatisation of closely related tetrahydro-β-carbolines with CQ to obtain conjugates with antimalarial activity has recently been explored. These conjugates demonstrated moderate in vitro antimalarial activity against the CQ-resistant *P. falciparum* strain (W2) in the low IC_50_ micromolar range (IC_50_ from 0.49 to 9.28 μM) [24].

**Figure 1:**
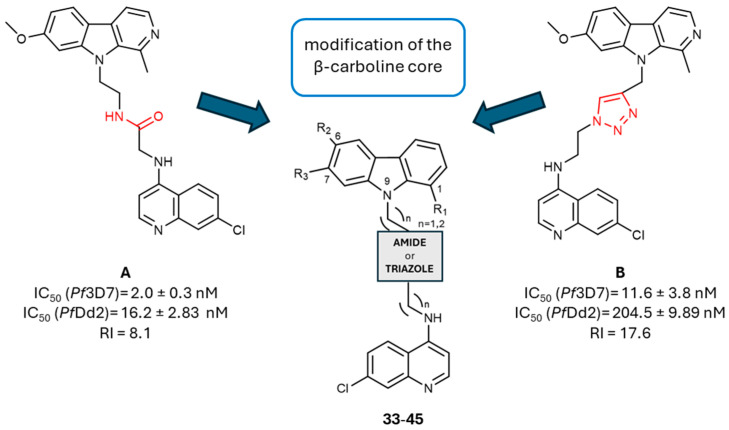
The most active representatives from the study of harmiquins [28] (**A**,**B**) and schematic display of the title compounds **33**–**45**.

Our research group has been extensively exploring β-carboline hybrids as potential antimalarial compounds [25,26,27,28,29,30,31], and the most active ones were hybrids composed of β-carboline and CQ core. Poje et al. have studied the impact of various positions of substitution on the β-carboline on the antimalarial activity and have established that derivatives obtained by substitution at position 9 of the β-carboline are the most active compounds with IC_50_ values in the low nanomolar range (Figure 1) against CQ-sensitive (3D7) and resistant (Dd2) *P. falciparum* strains [28].

Building upon the favourable outcomes of our prior research [28], and recognising the importance of the quinoline core for antimalarial activity, we have set to prepare two series of novel hybrid compounds, triazole-type (TT) and amide-type (AT) harmiquins of the 2nd generation. Our objective was to deepen our understanding of the structure–activity relationships (SAR) and investigate the impact of modifications to the β-carboline core on antimalarial potency as well as to study their cytotoxic profile.

In addition, two representatives from the title compound series differing in the length and flexibility of a linker connecting two pharmacologically active subunits, namely, the short, flexible AT derivative 34 and the longer, more rigid TT derivative 39, were chosen for more detailed investigation of their interactions with important biological targets: DNA, RNA, and HSA.

## 2. Results

### 2.1. Chemistry

In this work, we report the synthesis of the second generation of harmiquins, hybrid compounds composed of harmine/β-carboline and 4-amino-7-chloroquinoline moieties, covalently bound at the *N*-9 position of the β-carboline ring via an amide bond (AT harmiquins **33**–**37**) or a triazole ring (TT harmiquins **38**–**45**), differing in substituents at the *C*-1, *O*-6, and *O*-7 positions of the β-carboline ring (Figure 1).

1,2,3-triazoles are an important class of compounds in medicinal chemistry due to their low cytotoxicity, high internal absorption, and good stability. Moreover, 1,2,3-triazoles show a wide range of biological activities, such as antimalarial and anticancer activity [32,33,34]. Amide bond, a bioisoster of a triazole ring, was chosen as an alternative linker [35].

TT harmiquins were synthesised by Cu(I)-catalysed azide-alkyne cycloaddition (CuAAC), known as “click“ chemistry, whereas the synthesis of AT harmiquins was performed by a standard coupling reaction. Synthesis of the title compounds required the synthesis of their building blocks: quinoline-based azide **3** and carboxylic acid **4**, and β-carboline-based amines **12**–**14**, **22,** and **24,** as well as alkynes **15**–**17**, **25**, **26,** and **30**–**32**, which were all obtained in multiple reaction steps. To this end, their β-carboline precursors **5** (harmole), **18, 19,** and **27**–**29**, 7-chloroquinoline-based azide **3**, and carboxylic acid **4** were prepared according to procedures published by us or others [25,26,27,28,36].

**Scheme 1:**
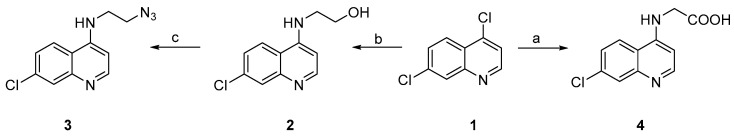
Synthesis of 7-chloroquinoline-based intermediates **3** and **4**. Reagents and reaction conditions: (a) H_2_NCH_2_COOH, C_6_H_5_OH, 125 °C, 18 h; (b) H_2_N(CH_2_)_2_OH, TEA, 120 °C, 2 h; (c) ADMP, DBU, THF, 0 °C, 1.5 h.

Harmole **5** was obtained by acid-catalysed hydrolysis of harmine under MW irradiation. *O*-6 β-carboline **18** and *C*-1 β-carbolines **27**–**29** were obtained by Pictet–Spengler condensation of 5-methoxytriptamine/tryptamine with the corresponding aldehyde to give tetrahydro-β-carbolines, followed by oxidation of piperidine. Phenol **19** was synthesised by acid-catalysed hydrolysis of the β-carboline **18** under MW irradiation. Carboxylic acid **4** was obtained by substitution of 4,7-dichloroquinoline with glycine, while the synthesis of azide **3** involved a two-step procedure, the nucleophilic substitution of 4,7-dichloroquinoline with 2-aminoethanol, followed by the conversion of the corresponding alcohol **2** to azide **3** using ADMP and DBU (Scheme 1).

**Scheme 2:**
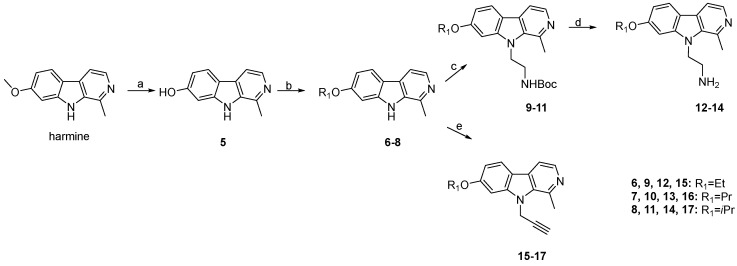
Synthesis of *O*-7-substituted β-carbolines **6**–**8**, Boc-protected amines **9**–**11,** amines **12**–**14** and alkynes **15**–**17**. Reagents and reaction conditions: (a) HBr/AcOH, MW, 140 °C, 25 min; (b) R_1_Br, Cs_2_CO_3_, DMF, 90 min–2 h, rt—60 °C; (c) BocNH(CH_2_)_2_Br, Cs_2_CO_3_, DMF, 90 °C, 18 h; (d) HCl/MeOH, 50 °C, 18 h; (e) propargyl bromide, NaH, DMF, rt, 2 h.

β-carboline-based intermediates (**6**–**8**, **18**, **20**, **27**–**29**) were all obtained in multiple reaction steps. *O*-7-substituted β-carbolines **6**–**8** were synthesised by alkylation of harmole **5** with ethyl, propyl, or isopropyl bromide, respectively (Scheme 2). In order to secure selective O-alkylation of harmole in good yields and to avoid the synthesis of N,O-bis-alkylated products, the reaction conditions needed to be optimised, and the final conditions are listed in Table 1. The synthesis of the desired compound **6** proceeded smoothly with 1.4 equivalents of Cs_2_CO_3_ and 1.2 equivalents of ethyl bromide. The preparation of compound **7** was conducted similarly in good yields using 1.4 equivalents of Cs_2_CO_3_ and 1.2 equivalents of propyl bromide, followed by the addition of another 3 equivalents of propyl bromide. On the other hand, the preparation of compound **8** was more challenging, but it was performed in moderate yields with 4.2 equivalents of Cs_2_CO_3_ and 8 equivalents of isopropyl bromide. Depending on the synthesis of compounds **6**–**8**, the reaction times varied between 2 and 4 h in total, and the reaction temperatures ranged from room temperature to 60 °C.

**Scheme 3:**
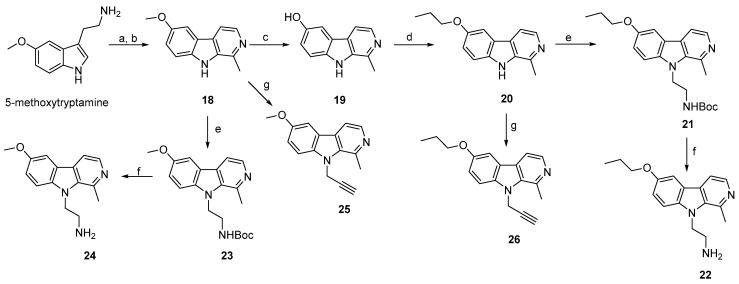
Synthesis of *O*-6-substituted β-carboline **20**, Boc-protected amines **21, 23**, amines **22, 24**, and alkynes **25, 26**. Reagents and reaction conditions: (a) (CH_3_O)_2_CHCH_3_, TFA, ACN, MW, 110 °C, 10 min; (b) Pd/C, Li_2_CO_3_, EtOH, MW, 150 °C, 25 min; (c) HBr/AcOH, MW, 140 °C, 25 min; (d) CH_3_(CH_2_)_2_Br, Cs_2_CO_3_, DMF, 40 °C, 2 h; (e) BocNH(CH_2_)_2_Br, Cs_2_CO_3_, DMF, 90 °C, 18 h; (f) HCl/MeOH, 50 °C, 18 h; (g) propargyl bromide, NaH, DMF, rt, 3 h.

Similarly, *O*-6-substituted β-carboline **20** was synthesised by alkylation of phenol **19** using 1.4 equivalents of Cs_2_CO_3_ and 1.2 equivalents of propyl bromide (Scheme 3, Table 1).

Synthesis of β-carboline-based amines at *N*-9 was carried out in two reaction steps. In the first step, *O*-7 substituted β-carbolines **6**–**8** and *O*-6 substituted β-carbolines **18** and **20** were alkylated with 2-(Boc-amino)ethyl bromide (4 equivalents)/Cs_2_CO_3_ (4.5 equivalents) at 90 °C for 18 h to yield Boc-protected amines **9**–**11**, **21,** and **23** (40–55%). In the second step, the Boc-protecting group was efficiently removed under acidic conditions to attain primary amines **12**–**14**, **22,** and **24** (Scheme 2 and Scheme 3). The removal of the Boc-protecting group for all the above-mentioned reactions was carried out at 50 °C for 18 h, except for 4 h for the compound **13**, and we managed to obtain the desired amines mainly in good yields (62–80%), except for the compound **14** (34%). In addition, we found that the removal of the Boc-protecting group resulted in slightly higher yields with *O*-7 substituted (**6**–**8**) in comparison with *O*-6 substituted β-carbolines (**18**, **20**) as starting materials.

However, this method was not applicable to the synthesis of the *C*-1 substituted β-carboline-based amines at *N-*9 due to the steric hindrance, which is the result of the ethyl (**27**)/propyl (**28**)/isopropyl (**29**) group located in position 1 of the β-carboline ring, unlike the methyl group with *O*-7 (**6**–**8**) and *O*-6 (**18**, **20**) substituted β-carbolines, respectively. Likewise, the Gabriel synthesis also did not afford the desired amines. Moreover, the Staudinger reduction of azides to amines, which involved the alkylation of the *C*-1 β-carbolines at *N-*9 with 2-bromoethan-1-ol and the conversion of the corresponding alcohol to azide using DBU, also was not efficient because we could not manage to obtain the desired alcohol in the first step. In our most recent attempt to acquire the desired amines, 2-chloroethan-1-amine was employed as an alkylating agent, but unfortunately, it failed to yield the intended products.

**Scheme 4:**
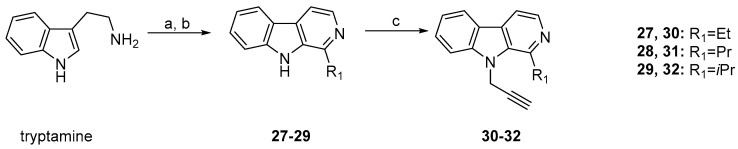
Synthesis of *C*-1-substituted β-carbolines **27**–**29** and alkynes **30**–**32**. Reagents and reaction conditions: (a) R_1_-CHO or R_1_-CH(OMe)_2_, TFA, DCE, MW, 110 °C, 3–6 min; (b) Li_2_CO_3_, Pd/C, EtOH, MW, 150 °C, 10 min; (c) propargyl bromide, NaH, DMF, rt, 3 h Et; Pr/iPr 24 h.

Alkylation of β-carbolines substituted at *O*-7 (**6**–**8**), *O*-6 (**18**, **20**), and *C*-1 (**27**–**29**) at *N-*9 with propargyl-bromide (3 equivalents)/60% NaH (2.66 equivalents) or Cs_2_CO_3_ (1.4 equivalents; compounds **31** and **32**) at room temperature for 2 h afforded β-carboline-based alkynes **15**–**17**, **25**, **26**, and **30**–**32** (Scheme 2, Scheme 3 and Scheme 4), mostly in moderate yields (40–55%), apart from the compounds **25** (10%) and **32** (29%), respectively. In general, the alkylation of *O*-7-substituted β-carbolines **6**–**8** resulted in the highest reaction yields.

**Scheme 5:**
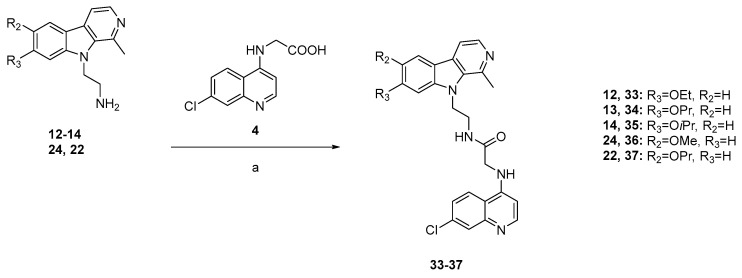
Synthesis of AT harmiquins **33**–**37**. Reagents and reaction conditions: (a) T3P, TEA, DMF, rt, 18 h.

AT harmiquins **33**–**37** were prepared by the coupling reaction of the previously synthesised β-carboline-based amines **12**–**14**, **22**, **24,** and 7-chloroquinoline-based carboxylic acid **4** using propylphosphonic anhydride (T3P) as a coupling agent and triethylamine (TEA) as a base. All the reactions were conducted at room temperature for 18 h and gave AT harmiquins in moderate yields (16–46%) (Scheme 5).

**Scheme 6:**
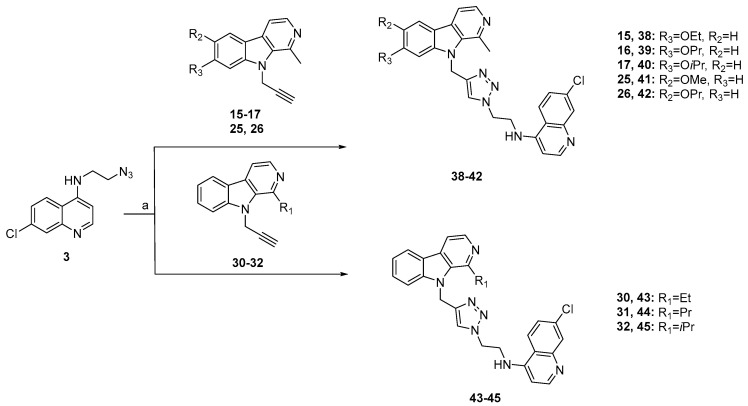
Synthesis of TT harmiquins **38**–**45**. Reagents and reaction conditions: (a) Cu(CH_3_COO)_2_, MeOH, rt, 24 h.

CuAAC was successfully applied to obtain TT harmiquins **38**–**45** from previously prepared β-carboline-based alkynes **15**–**17**, **25**, **26**, **30**–**32,** and 7-chloroquinoline-based azide **3** using the Cu(II) acetate precatalyst in methanol, in moderate to good yields (40–72%) (Scheme 6). The most substantial yield (72%) was achieved in the synthesis of TT harmiquin **39** using β-carboline-based alkyne **16** as a starting material.

β-carbolines with substituents at positions *O*-7 and *O*-6, β-carboline-based amines, and alkynes, as well as harmiquins, were fully characterised by standard spectrometric and spectroscopic methods, namely MS, IR, ^1^H, and ^13^C NMR. The data obtained are briefly given in the Materials and Methods section and in detail in the Supplementary Materials.

### 2.2. SwissADME Prediction Tool Calculations

Physicochemical descriptors of the title harmiquins **33**–**45** were assessed by using an online tool, SwissADME [37]. The following parameters were determined and are outlined in Table 2: molecular weight (MW), heavy atoms (HA), number of rotatable bonds (RB), number of H-bond acceptors (HBA), number of H-bond donors (HBD), topological polar surface area (TPSA), consensus partition coefficient (cLog P), Lipinski violations (LV), and Veber’s violations (VV). Although the number of H-bond acceptors (HBA), number of H-bond donors (HBD), and consensus partition coefficient (cLogP) comply with Lipinski’s rule of five, the majority of the title compounds, i.e., **34**, **35**, **37**–**40**, and **42**, deviate from this rule due to their molecular weight exceeding 500. Topological polar surface area (TPSA) represents the cumulative surface area of polar atoms or groups within a molecule, serving as a measure for assessing the compound’s cellular permeability [38]. TPSA values of all harmiquins **33**–**45** fall within the range of 73.45 to 82.68, indicating their favourable cellular permeability. On the other hand, all harmiquins comply with Veber’s guidelines (10 or fewer rotatable bonds and polar surface area equal to or less than 140 Å^2^ or 12 or fewer H-bond donors and acceptors), pointing to a high probability of their good oral bioavailability [39].

### 2.3. Biological Evaluations

#### 2.3.1. In Vitro Antiplasmodial Activity

The activity of the title compounds against the erythrocytic stage of the *Plasmodium* life cycle in vitro was investigated by using two *P. falciparum* strains, a CQ-sensitive (*Pf*3D7) and a multi-drug resistant (PfDd2) (Table 3), by employing previously described methods [40,41,42]. The resistance index (RI), which represents a ratio between IC_50_ obtained against *Pf*Dd2 and *Pf*3D7, was also calculated (Table 3).

The tested compounds can be divided into 3 subgroups for the purpose of comparison of their antiplasmodial activity and discussion about their structure–activity relationship (SAR) (Figure 1):

(1) AT harmiquins with alkoxy at positions 6 or 7 and methyl at position 1 of the β-carboline (**33**–**37**)
(2) TT harmiquins with alkoxy at positions 6 or 7 and methyl at position 1 of the β-carboline (**38**–**42**)
(3) TT harmiquins with alkyl at position 1 of the β-carboline (**43**–**45**)

Building upon the promising outcomes of our earlier investigation into harmiquins, we have directed our efforts towards the enrichment of our harmiquins library by synthesising analogues of the most potent compounds. Consequently, we have synthesised derivatives of the most promising compounds from both AT (**A**) and TT (**B**) harmiquins [28]. Structural elements that are known to enhance antimalarial activity, such as (1) the -NH- group directly attached to the quinoline and (2) the position of derivatisation on the β-carboline ring (*N-*9), have been retained in all newly synthesised compounds.

The results obtained from these experiments reveal exceptional activity of the tested compounds against the *P. falciparum* erythrocytic stage with slightly reduced activity against multi-drug-resistant *P. falciparum.* Thus, the modification of the β-carboline did not have a high influence on the activity of the compounds (with the exception of compounds **43** and **44**).

All newly prepared harmiquins were considerably more potent than the parent compound, harmine, against both tested strains. Compounds **33**, **37**, **39,** and **42** with IC_50_ values ranging from 4.7 ± 1.3 to 7.6 ± 0.2 nM against the *Pf*3D7 strain were more active than CQ (IC_50_ = 11 ± 3 nM). All tested compounds, except **43** and **44,** were notably more active than CQ (119.4 ± 39.2 nM) against the resistant strain *Pf*Dd2, with IC_50_ values ranging from 6.5 ± 2.5 to 98.7 ± 36.3 nM.

Resistance indices (RIs, Table 3) calculated as the ratio between IC_50_ obtained for Dd2 and (3D7) *P. falciparum* strain (IC_50_(*Pf*Dd2)/IC_50_(*Pf*3D7)) are in the range from 0.9 to 5.3 for all compounds, which demonstrates their persistent activity against both strains. In addition, all compounds exhibited lower RIs compared to CQ (14.4), indicating their more consistent activity against both strains. Almost identical activity against both strains (RIs < 2) was observed for the compounds **33**–**35**, **40** (compounds bearing alkoxy substituents at position 6 of the β-carboline), and **45**. These results demonstrate that the novel compounds exhibit greater activity against the resistant Dd2 strain compared to the most active first-generation harmiprims, **A** and **B** (Figure 1), whose resistance indices (RIs) were 17.6 and 8.1, respectively.

Comparing the activity of AT and TT harmiquins, we observed that AT harmiquins were generally more potent than TT harmiquins, which is consistent with our previous research [27,28]. In the present study, this observation appears more pronounced in the case of the resistant strain (*Pf*Dd2) compared to the CQ-sensitive strain (*Pf*3D7). The IC_50_ values for all AT harmiquins (first subgroup, **33**–**37**) exhibit a range spanning from 4.6 ± 0.6 to 28.1 ± 0.5 nM against the CQ-sensitive strain (*Pf*3D7) and from 6.5 ± 2.5 to 36.7 ± 4.0 nM against resistant strain (*Pf*Dd2). On the other hand, the range of IC_50_ values for TT harmiquins (second and third subgroup, **38**–**45**) spans from 6.8 ± 4.0 to 96.9 ± 1.5 nM for *Pf*3D7 and from 15.5 ± 1.7 to 213.8 ± 71.8 for *Pf*Dd2. The weakest activity against both strains tested was detected for compounds from the third subgroup, **43** and **44** (TT harmiquins that lack alkyloxy substituents at positions 6 and 7 of the β-carboline and have alkyl substituents at position 1 of the β-carboline with IC_50_ in the low micromolar range, rather than nanomolar as it is the case for other compounds from the series). The most active compound from the third subgroup was **45**, differing from **43** and **44** in the shape and size of the *C*-1 substituent (branched isopropyl in **45** vs. linear ethyl and propyl in **43** and **44**).

When we took a more detailed look at the type of the substituent and the position of the substitution on the β-carboline, we were able to draw the following conclusions:

(1) The activity of the compounds bearing alkoxy substituents at position 6 of the β-carboline: the ethoxy derivative was the most active in the case of AT compounds (**33**), the propoxy derivative was the most active in the case of the TT-type compounds (**39**), and the isopropoxy derivatives were the least active (**35** and **40**) against both strains tested.
(2) The activity of the compounds bearing alkoxy substituents at position 7 of the β-carboline: propoxy derivatives were more active than the methoxy derivatives in the case of both AT and TT compounds (**37** and **42**) against both strains tested.
(3) Depletion of the alkoxy substituents in positions 6 and 7 of the β-carboline strongly diminishes antimalarial activity except in the case of branched substituents at position 1 of the β-carboline (isopropyl).

#### 2.3.2. In Vitro Cytotoxicity Screening

The cytotoxicity of the second-generation harmiquins **33**–**45** was assessed against HepG2 cells to determine the selectivity of the tested compounds towards *P. falciparum* in comparison to mammalian cells (Table 4). Cytotoxicity of all tested compounds was comparable and within the range from 14 to 36 μM with the exception of compounds **43** and **44** (IC_50_ 68.2 ± 6.1 and 68.9 ± 4.3 μM, respectively) and compounds **35** and **45** (IC_50_ 126.7 ± 31.5 and 128.5 ± 2.7 μM, respectively). The selectivity indices (SIs) for all compounds ranged from 711.0 to 8081.8 and represented a marked improvement compared to the first generation of harmiquins [28]. The lowest SIs were calculated for compounds **43** and **44** (771.5 and 711.0, respectively), which were also compounds with the lowest antimalarial activity (Table 4). The highest SI was calculated for compound **45**, with an isopropyl substituent at position 1 of the β-carboline (8081.8), followed by compound **37** (AT harmiquin with propoxy at position 7 of the β-carboline), which was the most active compound in the antimalarial screening (7782.6). SIs for the compounds **33**–**38** and **40**–**42** were also very high, in the range from 1076.0 to 4508.9. Such high SIs show that second-generation harmiquins have marked selectivity against *P. falciparum* over mammalian cells, which demonstrates their favourable cytotoxicity.

#### 2.3.3. Study on Interactions with ds-DNA/RNA and Proteins

For the preliminary screening of interactions with ds-DNA/RNA and proteins, we selected two representative conjugates from a series of AT and TT harmiquins, **34** and **39**, respectively. We studied the interactions of derivatives **34** and **39** with the most commonly used ds-DNA/RNA representatives, namely naturally isolated calf thymus (ct)-DNA, synthetic sequences poly dAdT–dAdT and poly dGdC–dGdC, as well as the synthetic ds-RNA representative, poly A–poly U. Calf thymus DNA (ctDNA) is characterised by an equal amount of AT- and GC- base pairs and is a typical B-helical structure, as well as poly(dAdT)_2_ and poly(dGdC)_2_, while poly A–poly U is a representative of the typical RNA A-helical structure (Supplemental Information, Table S13). B-helical structures include minor grooves that can bind small molecules, and A-helices (poly A–poly U) contain a major groove as a potential target for small molecules [43]. For a model protein, we used human serum albumin (HSA).

##### Photophysical Studies of the **34** and **39** in Aqueous Solutions

Stock solutions of compounds were prepared in DMSO (*c* = 1 mM) and stored at +4 °C, whereas the daily working aliquots were kept at +25 °C. No visible precipitation in working solutions was noticed. The UV/vis spectra in aqueous solution are proportional to the concentration of the compound up to *c* = 2.5 × 10^−5^ M (Supplemental Information, Figures S1 and S2), yielding molar absorption coefficient (Table 5, *ε*). The obtained results do not support the formation of aggregates at the conditions used.

**Figure 2:**
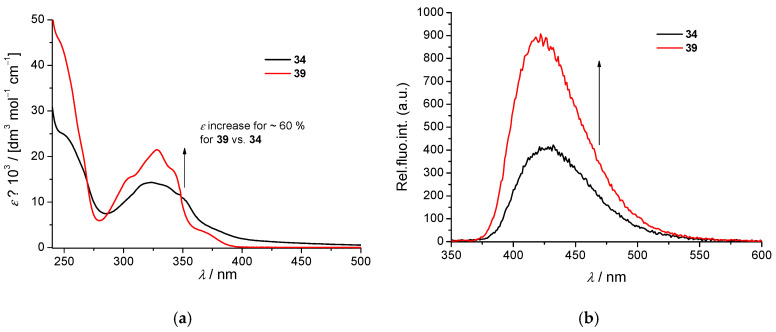
Comparison of: (**a**) UV/vis spectra, and (**b**) fluorescence spectra of compounds **34** and **39** at *c* = 2 × 10^−6^ M.

A detailed analysis of UV/vis spectra of close analogues **34** and **39** indicated a significant impact of the linker connecting two chromophore subunits on the overall spectral shape and intensity of the molecule. The absorption maximum (*λ*_abs_) for **39** is almost 60% higher and shifted for +4 nm compared to **34** (Figure 2a, Table 5). This increase in absorption intensity for **39** is due to π–π* electronic transitions, primarily originating from the triazole ring. Additionally, n–π* transitions in the UV/vis region are observed as a shoulder at *λ*_sh_ = 365 nm.

The fluorescence emission of both studied compounds follows the trend observed for UV/vis maxima (Supplemental Information, Figures S3 and S4), revealing stronger emission and a slight hypsochromic shift of -6 nm for **39** (Figure 2b, Table 5). Compounds exhibit remarkable emission Stokes shifts of 103 and 93 nm for **34** and **39**, respectively, which can be attributed to the 4-aminoquinoline chromophore. The emission intensity of compounds was proportional to concentration within the 5 × 10^−7^–2 × 10^−6^ M range (Supplemental Information, Figures S3 and S4), pointing to the absence of any compound aggregation.

##### Interactions with ds-DNA/RNA—Fluorimetric Titrations of the Tested Compounds with Polynucleotides and HSA

**Figure 3:**
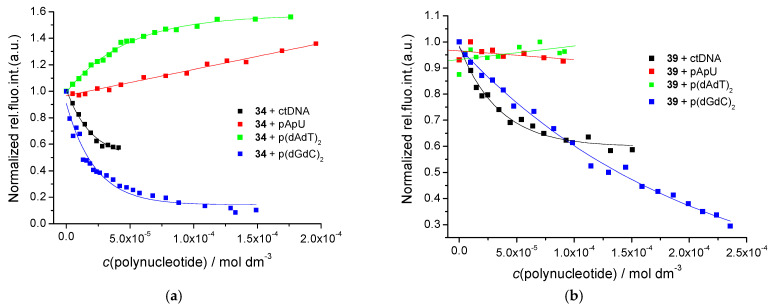

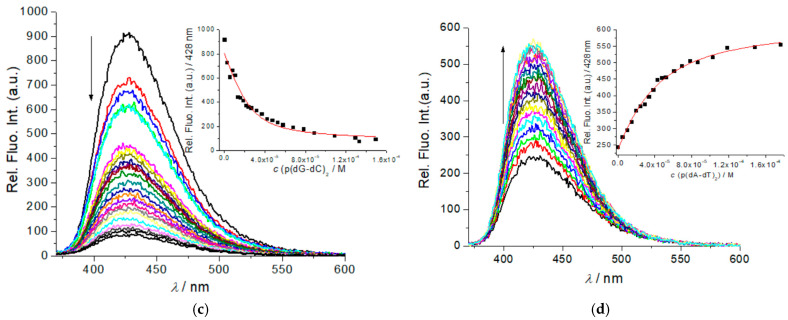
(**a**) Normalised fluorescence intensities of **34** (*c* = 5 × 10^−6^ M) upon titration with polynucleotides: ctDNA, pApU, poly(dAdT)_2_, and poly(dGdC)_2_) at 428 nm (*λ*_exc_ = 325 nm); (**b**) normalised fluorescence intensities of **39** (*c* = 5 × 10^−6^ M) upon titration with polynucleotides: ctDNA, pApU, poly(dAdT)_2_, and poly(dGdC)_2_) at 422 nm (*λ*_exc_ = 329 nm); (**c**) changes in the fluorescence spectrum of **34** (*c* = 5 × 10^−6^ M) upon titration with poly(dGdC)_2_ (*λ*_exc_ = 325 nm, *λ*_em_ = 428 nm), inset: dependence of **34** emission intensity at 428 nm on *c*(p(dGdC)_2_); (**d**) changes in the fluorescence spectrum of **34** (*c* = 5 × 10^−6^ M) upon titration with poly(dAdT)_2_ (*λ*_exc_ = 325 nm, *λ*_em_ = 428 nm), inset: dependence of **34** emission intensity at 428 nm on *c*(p(dAdT)_2_). Measured in sodium cacodylate buffer, pH 7, *I* = 0.05 M.

The strong emission and favourable Stokes shift of emission maxima allowed the performance of fluorimetric titrations with ds-DNA/RNA at very low (5 µM) concentrations of investigated compounds. In general, the addition of any ds-DNA or ds-RNA to studied compounds resulted in pronounced hyper- or hypochromic changes of the emission maxima (Supplemental Information, Figures S5–S12), showing selectivity in some cases (Figure 3a). For example, interaction with GC-containing DNAs (ctDNA and poly(dGdC)_2_) quenched derivative **34** fluorescence (Figure 3c), whereas its fluorescence is strongly increased upon the addition of poly(dAdT)_2_ (Figure 3d) or pApU polynucleotide.

At variance to **34**, the emission of conjugate **39** changed only upon the addition of ctDNA and poly(dGdC)_2_ (Figure 3b, Supplemental Information, Figures S6 and S12), and it was completely insensitive to the presence of AT-DNA or AU-RNA (Figure 3b, Supplemental Information, Figures S8 and S10).

The titration data are processed by a non-linear fitting procedure to the Scatchard equation [44,45,46], yielding binding constants (Supplemental Information, Figures S5–S12, Table 6). Generally, comparing binding constants between compounds revealed almost two orders of magnitude stronger affinity of **34** toward GC-containing DNAs (log *K*_s_ ≥ 6–7) compared to **39** (log *K*_s_ ≥ 4–5). Additionally, peptide derivative **34** showed significant emission change and moderate binding affinity toward AT-DNA and AU-RNA (log *K*_s_ ≥ 4–5), whereas the emission of analogue **39** did not change, thus not allowing the determination of the affinity.

Such results imply that linker structure strongly affects the fluorescence response of **34** compared to **39** and the affinity toward various ds-DNA/RNA. Further, both compounds’ emission response is strongly related to the basepair composition of DNA or RNA, as well as the difference between B-helical structures (ctDNA, p(dA–dT)_2_, and p(dG–dC)_2_) and A-helix (pApU) (Supplemental Information, Table S13). It seems that a shorter and more flexible linker of **34** allows better adaptation of this ligand within different ds-DNA or ds-RNA binding sites, resulting in the emission response being more sensitive to the local structural and/or H-bonding properties of DNA/RNA binding site(s). At variance to **34**, the longer and more rigid linker of **39**, characterised by planar and sterically demanding triazole, has a restricted possibility to adapt to ds-DNA/RNA binding site(s) and consequently shows significantly lower affinity. The observed emission change of **39** only upon binding to GC-containing DNAs could be attributed to the difference in the minor groove between AT- and GC-DNAs, the latter rich in guanine amino groups, which not only sterically direct ligand **39** in a very precise way but also can form H-bonding interactions with the triazole of **39**.

**Figure 4:**
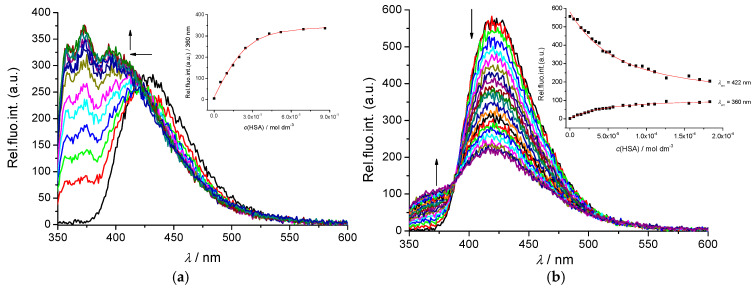
(**a**) Changes in the fluorescence spectrum of **34** (*c* = 5 × 10^−6^ M, *λ*_exc_ = 325 nm) upon titration with **HSA** (*c* = 5 × 10^−6^–1.5 × 10^−4^ M), inset: dependence of **34** emission intensity at 360 nm on c(HSA); (**b**) changes in the fluorescence spectrum of **39** (*c* = 5 × 10^−6^ M, *λ*_exc_= 329 nm) upon titration with **HSA** (*c* = 5 × 10^−6^–1.8 × 10^−4^ M), inset: dependence of **39** emission intensity at 360 and 422 nm on c(HSA). Measured in sodium cacodylate buffer, pH 7, *I* = 0.05 M.

As the pharmacokinetic properties of small molecules often depend on a binding affinity toward transport proteins (such as serum albumin, the most abundant protein in blood plasma), we performed fluorimetric titrations with human serum albumin (HSA). The fluorimetric response of tested compounds **34** and **39** upon HSA addition was significantly different; they both showed a strong hypsochromic shift of the emission maximum from *λ*_em_ = 428/422 nm to 360 nm, whereas smaller and more flexible **34** almost immediately completely converted to 360 nm (Figure 4a). In comparison, longer and more rigid **39** (Figure 4b) showed a gradual emission decrease at 422 nm accompanied by the appearance of a weak shoulder at 360 nm, with a clear isosbestic point indicating the formation of an equilibrium between the free chromophore **39** and a single type of HSA/**39** complex. Such a difference in response between **34** and **39** could be attributed to the better adaptation of the smaller and more flexible molecule **34** within the HSA binding site. These results are in good correlation with an order of magnitude higher affinity of **34** toward HSA compared to **39** (Table 6).

##### Circular Dichroism (CD) Experiments

To further investigate the binding of compounds **34** and **39** to ds-DNA/RNA in more structural detail, we used circular dichroism (CD) spectroscopy as a useful analytical tool in the binding study of small molecules to chiral macromolecules [47]. Small molecules can, upon binding, change the helical chirality of ds-DNA/RNA (observed as a change in spectral region *λ* < 300 nm), and also, achiral molecules can, upon binding to chiral DNA/RNA, acquire induced chirality (observed as an induced (I)CD signal(s) positioned at the absorption bands of the small molecule)—all these changes provide information on the binding mode (intercalation, agglomeration, groove binding, etc.) to polynucleotide [48,49].

It should be noted that studied compounds are achiral and thus do not interfere with the monitoring of the CD spectral properties of investigated polynucleotides.

**Figure 5:**
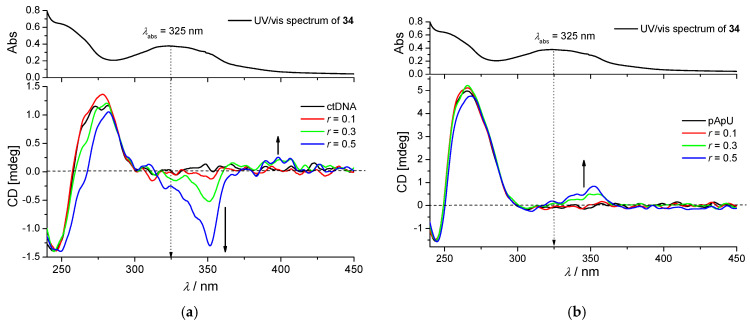
CD titration of: (**a**) ctDNA (c = 1 × 10^−5^ M) and (**b**) pApU (c = 2 × 10^−5^ M) with **34** at molar ratios *r* = [compound]/[polynucleotide]. Measured in sodium cacodylate buffer, pH 7, *I* = 0.05 M.

The addition of **34** or **39** resulted in small changes in the ds-DNA or ds-RNA CD spectrum (260–300 nm region; Figure 5, Supplemental Information, Figures S14–S15), implying only minor changes in the DNA/RNA helicity. These results consistently agree with negligible thermal (de)stabilisation effects (see below, Table 3).

However, induced CD (ICD) bands at the *λ* > 300 nm range were observed only upon binding of **34** to polynucleotides (Figure 5a,b). Only at the excess of **34** over ctDNA binding sites (Figure 5a, *r* > 0.2), a strong, bisignate ICD band appears, characterised by a negative sign at 350 nm coupled with a weak positive sign at about 400 nm. Such a bisignate ICD band is characteristic of the compound dimer (or oligomer) formation within the ctDNA minor groove [48]. The effect of **34** addition to AU-RNA was substantially different regarding the ICD band, ranging from 325–365 nm; only at molar ratios *r* > 0.1 did **34** cause a moderate increase in the positive ICD band (Figure 5b), which is characteristic of RNA major groove binding of a single molecule [48].

A significant difference in response signals obtained for ds-DNA and ds-RNA could be attributed to the difference in respective minor (DNA) and major (RNA) dimensions (Supplemental Information, Table S13), which controlled the formation of polynucleotide/**34** complex. In the case of triazole derivative **39**, no ICD bands in the spectral region *λ* > 300 nm were observed, suggesting that longer and more rigid linkers hampered the adaptation of small molecules within the DNA/RNA binding site, which would be uniformly oriented concerning the DNA/RNA chiral axis [47,48,49].

##### Thermal Denaturation of ds-DNA/RNA

Thermal denaturation experiments provide information about the ds-polynucleotide helix thermal stability as a function of interaction with added small molecules [50]. The difference between the *T*_m_ value of free ds-polynucleotide and a complex with a small molecule (Δ*T*_m_ value) is important in characterising small molecule/ds-polynucleotide interactions. For instance, moderate to strong stabilisation (Δ*T*_m_ > 5 °C) supports pronounced intercalative or minor groove binding interaction [51]. In contrast, weak or negligible stabilisation (Δ*T*_m_ = 0–5 °C) suggests a binding process driven mostly by hydrophobic effect and weak H-bonding and/or electrostatic interactions—usually excluding classical intercalation as a binding mode.

The addition of studied compounds to ds-DNA or ds-RNA yielded only negligible change in the thermal denaturation of ds-DNA (ctDNA) or ds-RNA (pApU) (Table 7, Supplemental Information, Figures S16–S19). Taking into account the high binding affinity of studied compounds toward dsRNA/RNA, the low stabilisation effect suggests that binding is mostly controlled by hydrophobic interactions inside DNA/RNA grooves, thus not stabilising the secondary structure of a polynucleotide.

## 3. Materials and Methods

### 3.1. Chemistry

#### 3.1.1. General Information

Melting points were determined on a Stuart Melting Point Apparatus (Barloworld Scientific, London, UK) in open capillaries and were uncorrected. FTIR-ATR spectra were recorded using a Fourier transform infrared attenuated total reflection (UATR) spectrometer (PerkinElmer, Waltham, MA, USA) in the range from 450 to 4000 cm^−1^. ^1^H and ^13^C NMR spectra were recorded on a Bruker Avance III HD operating at 300, 400, or 600 MHz for the ^1^H and 75, 101, or 151 MHz for the ^13^C nuclei (Bruker, Billerica, MA, USA). Samples were measured in DMSO-*d*_6_ solutions at 20 °C in 5 mm NMR tubes. Chemical shifts (*δ*) are reported in parts per million (ppm) using tetramethylsilane (TMS) as a reference in the ^1^H and DMSO residual peak as a reference in the ^13^C spectra (39.52 ppm). Coupling constants (*J*) are reported in hertz (Hz). Mass spectra were recorded on Agilent 1200 Series HPLC coupled with Agilent 6410 Triple Quad (Agilent Technologies, St. Clara, CA, USA). The mobile phase consisted of Milli Q water as component A and MeOH (HPLC grade, J. T. Baker) as component B, and as the stationary phase, the Zorbax XDC C18 column (4.6 × 75 mm, 3.5 μm) was used. Gradient elution was used at a flow rate of 0.5 mL/min, and 5 μL of analyte solution was injected per analysis. The starting conditions and gradient steepness were adjusted according to the analyte polarity. A diode array detector was utilised, while the data were presented as a total wavelength chromatogram (TWC). Mass spectrometry conditions were as follows: electrospray ionisation (ESI) in positive and negative modes was used. Capillary voltage and current were set to 4.0 kV and 20 nA, respectively. Nebulizer pressure was set to 15 psi, while the drying gas (nitrogen) temperature and flow were 300 °C and 11 L/min. For the MS data analysis, Agilent MassHunter software (Agilent Technologies, St. Clara, CA, USA) was used. Microwave-assisted reactions were performed in a microwave reactor, CEM Discover (CEM, Matthews, NC, USA), in a glass reaction vessel. All compounds were routinely checked by TLC with silica gel 60F-254 glass plates (Merck, Darmstadt, Germany) using DCM/MeOH or cyclohexane/EtOAc/MeOH as the solvent system. Spots were visualised by UV light (λ = 254 nm; 365 nm). Column chromatography was performed on silica gel 0.063–0.200 mm (Sigma-Aldrich, St. Louis, MO, USA) with the same eluents used for TLC. All chemicals and solvents were of analytical grade and purchased from commercial sources.

Harmole **5**, β-carbolines **18**–**20**, **24**, phenol **27**, 7-chloroquinoline-based azide **3**, and carboxylic acid **4** were prepared according to procedures published by us or others [20,25,26,28,36].

#### 3.1.2. General Procedure for the Synthesis of β-Carbolines **6**–**8**

To a stirred solution of harmole **5** in anhydrous DMF (7 mL), under an argon atmosphere, Cs_2_CO_3_ was added. The resulting suspension was stirred at room temperature for 20 min, followed by the addition of an appropriate alkyl bromide. The reaction mixture was stirred at room temperature, then at 40 °C or 60 °C, cooled down, poured into H_2_O (70 mL), and extracted with ethyl acetate (3 × 70 mL). The collected organic layers were washed with H_2_O and brine, dried over anhydrous sodium sulfate, filtered, and evaporated under reduced pressure. The crude product was purified by column chromatography (DCM:MeOH = 8:1, then DCM:MeOH = 75:25) and triturated with diethyl ether.

##### 7-Ethoxy-1-methyl-9*H*-pyrido[3,4-*b*]indole (**6**)

Harmole **5**: 0.500 g, 2.52 mmol; Cs_2_CO_3_: 1.151 g, 3.53 mmol, 1.4 equivalents; ethyl bromide: 3.03 mmol, 1.2 equivalents; 2 h at room temperature and 45 min at 40 °C; yield: 0.382 g (67%); mp 193–194.5 °C; IR (ATR, *ν*/cm^−1^) 2977, 2867, 1628, 1568, 1444, 1328, 1277, 1171, 1107, 1041, 966, 871, 832, 813, 769, 686, 653; ^1^H NMR (DMSO-*d_6_*) *δ* 11.43 (s, 1H), 8.15 (d, 1H, *J* = 5.2 Hz), 8.04 (d, 1H, *J* = 8.6 Hz), 7.81 (d, 1H, *J* = 5.3 Hz), 7.00 (d, 1H, *J* = 2.1 Hz), 6.83 (dd, 1H, *J* = 8.6, 2.2 Hz), 4.13 (q, 2H, *J* = 7.0 Hz), 2.51 (s, 2H), 1.40 (t, 3H, *J* = 6.9 Hz); ^13^C NMR (DMSO-*d_6_*) *δ* 158.78, 141.44, 140.52, 136.88, 133.90, 126.76, 122.04, 114.13, 111.35, 108.85, 94.54, 62.68, 19.61, 14.10; ESI-MS: *m*/*z* 227.0 (M + 1)^+^.

##### 1-Methyl-7-propoxy-9*H*-pyrido[3,4-*b*]indole (**7**)

Harmole **5**: 0.450 g, 2.27 mmol; Cs_2_CO_3_: 1.036 g, 3.18 mmol, 1.4 equivalents; propyl bromide: 0.335 g, 2.72 mmol, 1.2 equivalents. After stirring at room temperature for 2 h, propyl bromide (1.005 g, 8.16 mmol, 3.0 equivalents) was added. The reaction mixture was stirred at room temperature for 1 h and 30 min at 40 °C. Yield: 0.397 g (73%); mp 203–205.5 °C; IR (ATR, *ν*/cm^−1^) 2963, 2871, 2765, 1626, 1569, 1486, 1447, 1328, 1297, 1281, 1235, 1176, 1136, 1105, 1071, 985, 878, 837, 819, 801, 686, 655, 633; ^1^H NMR (DMSO-*d_6_*) *δ* 11.43 (s, 1H), 8.15 (d, 1H, *J* = 5.3 Hz), 8.04 (d, 1H, *J* = 8.6 Hz), 7.80 (d, 1H, *J* = 5.2 Hz), 7.00 (d, 1H, *J* = 1.9 Hz), 6.84 (dd, 1H, *J* = 8.6, 2.1 Hz), 4.03 (t, 2H, *J* = 6.5 Hz), 2.73 (s, 3H), 1.80 (h, 2H, *J* = 7.1 Hz), 1.03 (t, 3H, *J* = 7.4 Hz); ^13^C NMR (DMSO-*d_6_*) *δ* 158.91, 141.41, 140.56, 136.96, 133.92, 126.71, 122.01, 114.15, 111.33, 108.84, 94.61, 68.57, 21.50, 19.67, 9.89; ESI-MS: *m*/*z* 241.1 (M + 1)^+^.

##### 7-Isopropoxy-1-methyl-9*H*-pyrido[3,4-*b*]indole (**8**)

Harmole **5**: 0.400 g, 2.02 mmol; Cs_2_CO_3_: 2.762 g, 8.476 mmol, 4.2 equivalents; isopropyl bromide: 1.986 g, 16.144 mmol, 8 equivalents; 90 min at room temperature and 90 min at 60 °C; yield: 0.229 g (47%); mp 178–180 °C; IR (ATR, *ν*/cm^−1^) 3051, 2923, 2854, 1626, 1568, 1449, 1380, 1275, 1183, 1167, 978, 878, 805, 640; ^1^H NMR (DMSO-*d_6_*) *δ* 11.44 (s, 1H), 8.15 (d, 1H, *J* = 4.8 Hz), 8.04 (d, 1H, *J* = 8.5 Hz), 7.82 (d, 1H, *J* = 4.7 Hz), 7.00 (s, 1H), 6.82 (d, 1H, *J* = 8.1 Hz), 4.76–4.70 (m, 2H), 2.73 (s, 2H), 1.34 (d, 6H, *J* = 5.7 Hz); ^13^C NMR (DMSO-*d_6_*) *δ* 157.66, 141.56, 140.38, 136.61, 133.91, 126.87, 122.16, 114.10, 111.40, 109.77, 96.00, 68.98, 21.24, 19.46; ESI-MS: *m*/*z* 241.1 (M + 1)^+^.

#### 3.1.3. 1-Methyl-6-propoxy-9*H*-pyrido[3,4-*b*]indole (**20**)

To a stirred solution of phenol **19** (0.330 g, 1.66 mmol) in anhydrous DMF (6 mL), under an argon atmosphere, Cs_2_CO_3_ (0.759 g, 2.33 mmol, 1.4 equivalents) was added. The resulting suspension was stirred at room temperature for 20 min, followed by the addition of propyl bromide (0.246 g, 2.00 mmol, 1.2 equivalents). The reaction mixture was stirred at room temperature for 2 h, poured into H_2_O (60 mL), and extracted with ethyl acetate (3 × 60 mL). The collected organic layers were washed with H_2_O and brine, dried over anhydrous sodium sulfate, filtered, and evaporated under reduced pressure. After purification by column chromatography (DCM:MeOH = 8:1) and trituration with diethyl ether, 0.159 g (53%) of **20** was obtained; mp 190.5–191 °C; IR (ATR, *ν*/cm^−1^) 2954, 1580, 1486, 1448, 1324, 1287, 1212, 1047, 1022, 909, 877, 844, 815, 766, 622; ^1^H NMR (DMSO-*d_6_*) *δ* 11.35 (s, 1H), 8.15 (d, 1H, *J* = 5.3 Hz), 7.90 (d, 1H, *J* = 5.3 Hz), 7.73 (d, 1H, *J* = 2.4 Hz), 7.49 (d, 1H, *J* = 8.8 Hz), 7.17 (dd, 1H, *J* = 8.8, 2.5 Hz), 4.02 (t, 2H, *J* = 6.5 Hz), 2.74 (s, 3H), 1.79 (h, 2H, *J* = 7.2 Hz), 1.03 (t, 3H, *J* = 7.4 Hz); ^13^C NMR (DMSO-*d_6_*) *δ* 153.11, 142.60, 137.32, 135.68, 135.54, 127.22, 121.90, 118.71, 113.17, 113.16, 104.94, 70.11, 22.73, 20.85, 11.02; ESI-MS: *m*/*z* 241.2 (M + 1)^+^.

#### 3.1.4. General Procedure for the Synthesis of β-Carboline-Based Boc-Protected Amines **9**–**11**, **21**, **23**

To a stirred solution of compounds **6**–**8**, **18**, or **20** in anhydrous DMF (6 mL) at 90 °C under an argon atmosphere, Cs_2_CO_3_ (4.5 equivalents) was added. The resulting suspension was stirred at 90 °C for 20 min, followed by the addition of 2-(Boc-amino)ethyl bromide (4 equivalents). The reaction mixture was stirred at 90 °C for 18 h, cooled down, poured into H_2_O (60 mL), and extracted with ethyl acetate (3 × 60 mL). The collected organic layers were washed with H_2_O and brine, dried over anhydrous sodium sulfate, filtered, and evaporated under reduced pressure. The crude product was purified by column chromatography (DCM:MeOH = 8:1) and triturated with diethyl ether.

##### *tert*-Butyl (2-(7-ethoxy-1-methyl-9*H*-pyrido[3,4-*b*]indol-9-yl)ethyl)carbamate (**9**)

Compound **6**: 0.320 g, 1.41 mmol; Cs_2_CO_3_: 2.074 g, 6.36 mmol; 2-(Boc-amino)ethyl bromide: 1.268 g, 5.66 mmol; yield: 0.287 g (55%); mp 167–167.5 °C; IR (ATR, *ν*/cm^−1^) 3239, 2974, 1698, 1623, 1564, 1503, 1447, 1365, 1308, 1276, 1247, 1165, 1148, 1124, 1048, 977, 962, 874, 802, 733, 637; ^1^H NMR (DMSO-*d_6_*) *δ* 8.15 (d, 1H, *J* = 5.1 Hz), 8.06 (d, 1H, *J* = 8.5 Hz), 7.85 (d, 1H, *J* = 5.0 Hz), 7.19 (s, 1H), 7.04 (t, 1H, *J* = 5.7 Hz), 6.86 (d, 1H, *J* = 8.5 Hz), 4.54 (t, 2H, *J* = 6.4 Hz), 4.18 (q, 2H, *J* = 6.8 Hz), 3.33 (s, 3H), 2.94 (s, 3H), 1.41 (t, 3H, *J* = 6.9 Hz), 1.29 (s, 8H), 1.00 (s, 1H); ^13^C NMR (DMSO-*d_6_*) *δ* 160.17, 156.14, 143.38, 140.97, 138.17, 135.21, 128.92, 122.77, 114.78, 112.67, 109.66, 94.88, 78.30, 63.93, 44.48, 40.58, 28.54, 23.58, 15.17; ESI-MS: *m*/*z* 370.2325 (M + 1)^+^.

##### *tert*-Butyl (2-(1-methyl-7-propoxy-9*H*-pyrido[3,4-*b*]indol-9-yl)ethyl)carbamate (**10**)

Compound **7**: 0.340 g, 1.42 mmol; Cs_2_CO_3_: 2.075 g, 6.37 mmol; 2-(Boc-amino)ethyl bromide: 1.268 g, 5.66 mmol; yield: 0.261 g (48%); mp 178–179.5 °C; IR (ATR, *ν*/cm^−1^) 3190, 2970, 1698, 1622, 1567, 1447, 1391, 1364, 1276, 1245, 1164, 1147, 1124, 1042, 989, 968, 876, 802, 769, 682, 638; ^1^H NMR (DMSO-*d_6_*) *δ* 8.15 (d, 1H, *J* = 5.1 Hz), 8.06 (d, 1H, *J* = 8.5 Hz), 7.85 (d, 1H, *J* = 5.1 Hz), 7.22 (d, 1H, *J* = 2.2 Hz), 7.05 (t, 1H, *J* = 6.0 Hz), 6.86 (dd, 1H, *J* = 8.6, 2.1 Hz), 4.54 (t, 2H, *J* = 6.7 Hz), 4.09 (t, 2H, *J* = 6.6 Hz), 3.32 (d, 2H, *J* = 7.1 Hz), 2.94 (s, 3H), 1.82 (d, 2H, *J* = 7.1 Hz), 1.30 (s, 8H), 1.04 (d, 3H, *J* = 7.4 Hz); ^13^C NMR (DMSO-*d_6_*) *δ* 160.34, 156.16, 143.38, 140.96, 138.17, 135.19, 128.91, 122.74, 114.74, 112.66, 109.77, 94.81, 78.31, 69.86, 44.48, 40.59, 28.54, 23.57, 22.61, 10.96; ESI-MS: *m*/*z* 384.3 (M + 1)^+^.

##### *tert*-Butyl (2-(7-isopropoxy-1-methyl-9*H*-pyrid [3,4-*b*]indol-9-yl)ethyl)carbamate (**11**)

Compound **8**: 0.300 g, 1.25 mmol; Cs_2_CO_3_: 1.830 g, 5.62 mmol; 2-(Boc-amino)ethyl bromide: 1.119 g, 4.99 mmol; yield: 0.193 g (40%); mp 173.5–174.5 °C; IR (ATR, *ν*/cm^−1^) 3240, 2978, 2934, 1702, 1620, 1561, 1497, 1364, 1333, 1244, 1225, 1168, 1037, 967, 876, 833, 816, 779, 646; ^1^H NMR (DMSO-*d_6_*) *δ* 8.15 (d, 1H, *J* = 5.1 Hz), 8.05 (d, 1H, *J* = 8.5 Hz), 7.85 (d, 1H, *J* = 5.2 Hz), 7.20 (s, 1H), 7.04 (t, 1H, *J* = 5.7 Hz), 6.84 (dd, 1H, *J* = 8.5, 1.6 Hz), 4.84–4.79 (m, 1H), 4.53 (t, 2H, *J* = 6.5 Hz), 3.33 (s, 2H), 2.94 (s, 3H), 1.36 (d, 6H, *J* = 6.0 Hz), 1.29 (s, 8H), 0.99 (s, 1H); ^13^C NMR (DMSO-*d_6_*) *δ* 157.95, 155.08, 142.39, 139.85, 137.08, 134.15, 127.83, 121.72, 113.63, 111.57, 109.37, 94.99, 77.23, 68.85, 43.35, 39.56, 27.46, 22.49, 21.34; ESI-MS: *m*/*z* 284.3 (M + 1)^+^.

##### *tert*-Butyl (2-(1-methyl-6-propoxy-9*H*-pyrido[3,4-*b*]indol-9-yl)ethyl)carbamate (**21**)

Compound **20**: 0.249 g, 1.05 mmol; Cs_2_CO_3_: 1.531 g, 4.70 mmol; 2-(Boc-amino)ethyl bromide: 0.936 g, 4.18 mmol; anhydrous DMF: 5 mL; yield: 0.200 g (50%); mp 175–181.5 °C; IR (ATR, *ν*/cm^−1^) 3208, 2964, 1702, 1566, 1487, 1367, 1275, 1223, 1050, 840, 814, 743, 623; ^1^H NMR (DMSO-*d_6_*) *δ* 8.16 (d, 1H, *J* = 5.2 Hz), 7.96 (d, 1H, *J* = 5.2 Hz), 7.77 (d, 1H, *J* = 2.5 Hz), 7.57 (d, 1H, *J* = 9.0 Hz), 7.23 (dd, 1H, *J* = 8.9, 2.4 Hz), 7.03 (t, 1H, *J* = 5.9 Hz), 4.57 (t, 2H, *J* = 6.7 Hz), 4.03 (t, 2H, *J* = 6.5 Hz), 3.28 (q, 2H, *J* = 6.6 Hz), 2.96 (s, 3H), 1.79 (h, 2H, *J* = 7.2 Hz), 1.31 (s, 6H), 1.03 (t, 3H, *J* = 7.4 Hz); ^13^C NMR (DMSO-*d_6_*) *δ* 155.07, 152.34, 140.72, 136.28, 135.59, 134.40, 127.23, 120.42, 117.61, 112.41, 110.44, 103.81, 77.25, 69.12, 43.32, 39.64, 27.50, 22.58, 21.60, 9.91; ESI-MS: *m*/*z* 384.20 (M + 1)^+^.

##### *tert*-Butyl (2-(6-methoxy-1-methyl-9*H*-pyrido[3,4-*b*]indol-9-yl)ethyl)carbamate (**23**)

Compound **18**: 0.449 g, 2.12 mmol; Cs_2_CO_3_: 3.101 g, 9.52 mmol; 2-(Boc-amino)ethyl bromide: 1.896 g, 8.46 mmol; anhydrous DMF: 7 mL; yield: 0.200 g (50%); mp 161–162 °C; IR (ATR, *ν*/cm^−1^) 3205, 2990, 1702, 1570, 1489, 1475, 1389, 1355, 1284, 1248, 1227, 1202, 1187, 1122, 1048, 1024, 863, 815, 805, 757, 705, 670; ^1^H NMR (DMSO-*d_6_*) *δ* 8.17 (d, 1H, *J* = 5.2 Hz), 7.97 (d, 1H, *J* = 5.2 Hz), 7.78 (d, 1H, *J* = 2.5 Hz), 7.59 (d, 1H, *J* = 9.0 Hz), 7.23 (dd, 1H, *J* = 8.9, 2.5 Hz), 7.03 (t, 1H, *J* = 5.9 Hz), 4.58 (t, 2H, *J* = 6.7 Hz), 3.86 (s, 3H), 3.29 (q, 2H, *J* = 6.5 Hz), 2.96 (s, 3H), 1.31 (s, 8H), 1.04 (s, 1H); ^13^C NMR (DMSO-*d_6_*) *δ* 156.16, 154.10, 141.84, 137.34, 136.72, 135.48, 128.32, 121.46, 118.32, 113.47, 111.59, 103.90, 78.33, 56.16, 44.42, 40.73, 28.58, 23.65; ESI-MS: *m*/*z* 356.25 (M + 1)^+^.

#### 3.1.5. General Procedure for the Synthesis of β-Carboline-Based Amines **12**–**14**, **22**, **24**

A solution of the corresponding compound **9**–**11**, **21**, or **23** and 4 M HCl (10 equivalents) in MeOH was stirred at 50 °C for 18 h or 4 h (compound **13**). Upon completion, the solvent was removed under reduced pressure. The residue was dissolved in H2O (20 mL), basified to pH 12 with 5% NaOH, and extracted with ethyl acetate (5×40 mL). The collected organic layers were dried over anhydrous sodium sulfate, filtered, and evaporated under reduced pressure. The crude product was triturated with diethyl ether.

##### 2-(7-Ethoxy-1-methyl-9*H*-pyrido[3,4-*b*]indol-9-yl)ethan-1-amine (**12**)

Compound **9**: 0.345 g, 0.93 mmol; MeOH: 6 mL; yield: 0.181 g (72%); mp 156–157.5 °C; IR (ATR, *ν*/cm^−1^); ^1^H NMR (DMSO-*d_6_*) *δ* 8.15 (d, 1H, *J* = 5.1 Hz), 8.06 (d, 1H, *J* = 8.5 Hz), 7.85 (d, 1H, *J* = 5.1 Hz), 7.22 (s, 1H), 6.85 (dd, 1H, *J* = 8.5 Hz, 1.5 Hz), 4.51 (t, 2H, *J* = 7.2 Hz), 4.18 (q, 2H, *J* = 6.9 Hz), 2.96 (s, 3H), 2.90 (t, 2H, *J* = 7.2 Hz), 1.65 (s, 2H), 1.40 (t, 3H, *J* = 6.9 Hz); ^13^C NMR (DMSO-*d_6_*) *δ* 160.18, 143.41, 141.08, 138.08, 135.26, 128.69, 122.75, 114.58, 112.63, 109.76, 94.90, 63.98, 47.66, 42.61, 23.76, 15.16; ESI-MS: *m*/*z* 270.10 (M + 1)^+^.

##### 2-(1-Methyl-7-propoxy-9*H*-pyrido[3,4-*b*]indol-9-yl)ethan-1-amine (**13**)

Compound **10**: 0.245 g, 0.64 mmol; MeOH: 4 mL; yield: 0.145 g (80%); mp 167.5–173 °C; IR (ATR, *ν*/cm^−1^); ^1^H NMR (DMSO-*d_6_*) *δ* 8.16 (d, 1H, *J* = 5.2 Hz), 8.07 (d, 1H, *J* = 8.5 Hz), 7.86 (d, 1H, *J* = 5.1 Hz), 7.30 (d, 1H, *J* = 2.2 Hz), 6.87 (dd, 1H, *J* = 8.6, 2.1 Hz), 4.64 (t, 2H, *J* = 7.5 Hz), 4.10 (t, 2H, *J* = 6.5 Hz), 3.00 (t, 2H, *J* = 7.6 Hz), 2.97 (s, 3H), 2.51 (s, 2H), 1.81 (h, 2H, *J* = 7.1 Hz), 1.04 (t, 3H, *J* = 7.4 Hz); ^13^C NMR (DMSO-*d_6_*) *δ* 160.49, 143.29, 141.06, 138.28, 135.12, 128.87, 122.83, 114.65, 112.67, 109.98, 94.88, 69.98, 45.39, 41.08, 23.72, 22.62, 11.01; ESI-MS: *m*/*z* 284.3 (M + 1)^+^.

##### 2-(7-Isopropoxy-1-methyl-9*H*-pyrido[3,4-*b*]indol-9-yl)ethan-1-amine (**14**)

Compound **11**: 0.184 g, 0.48 mmol; MeOH: 3 mL; yield: 0.046 g (34%); mp 105–107.5 °C; IR (ATR, *ν*/cm^−1^) 1619, 1412, 1218, 1155, 1109, 980, 844, 812; ^1^H NMR (DMSO-*d_6_*) *δ* 8.14 (d, 1H, *J* = 5.1 Hz), 8.05 (d, 1H, *J* = 8.6 Hz), 7.85 (d, 1H, *J* = 5.1 Hz), 7.22 (d, 1H, *J* = 2.0 Hz), 6.84 (dd, 1H, *J* = 8.6, 2.1 Hz), 4.86–4.80 (m, 1H), 4.51 (t, 2H, *J* = 7.2 Hz), 2.99 (s, 1H), 2.96 (s, 2H), 2.90 (t, 2H, *J* = 7.3 Hz), 1.34 (d, 6H, *J* = 6.0 Hz); ^13^C NMR (DMSO-*d_6_*) *δ* 158.99, 143.46, 141.05, 138.07, 135.29, 128.69, 122.81, 114.59, 112.63, 110.60, 96.35, 69.96, 47.57, 42.61, 29.13, 23.76; ESI-MS: *m*/*z* 284.3 (M + 1)^+^.

##### 2-(1-Methyl-6-propoxy-9*H*-pyrido[3,4-*b*]indol-9-yl)ethan-1-amine (**22**)

Compound **21**: 0.186 g, 0.49 mmol; MeOH: 3 mL; yield: 0.098 g (71%); mp 106.5–108.5 °C; IR (ATR, *ν*/cm^−1^) 2964, 1655, 1582, 1449, 1374, 1287, 1218, 1052, 991, 850, 813, 616; ^1^H NMR (DMSO-*d_6_*) *δ* 8.16 (d, 1H, *J* = 5.1 Hz), 7.96 (d, 1H, *J* = 5.0 Hz), 7.77 (s, 1H), 7.66 (d, 1H, *J* = 8.9 Hz), 7.22 (dd, 1H, *J* = 8.8, 1.6 Hz), 4.53 (t, 2H, *J* = 7.0 Hz), 4.03 (t, 2H, *J* = 6.4 Hz), 2.97 (s, 3H), 2.89 (t, 2H, *J* = 7.0 Hz), 1.79 (h, 2H, *J* = 6.9 Hz), 1.03 (t, 3H, *J* = 7.4 Hz); ^13^C NMR (DMSO-*d_6_*) *δ* 153.35, 141.91, 137.25, 136.73, 135.57, 128.06, 121.32, 118.67, 113.48, 111.92, 104.75, 70.17, 47.87, 42.82, 23.88, 22.70, 10.99; ESI-MS: *m*/*z* 284.20 (M + 1)^+^.

##### 2-(6-Methoxy-1-methyl-9*H*-pyrido[3,4-*b*]indol-9-yl)ethan-1-amine (**24**)

Compound **23**: 0.160 g, 0.45 mmol; MeOH: 3 mL; yield: 0.072 g (62%); mp 111–113 °C; IR (ATR, *ν*/cm^−1^) 1561, 1489, 1446, 1290, 1221, 1049, 814, 616; ^1^H NMR (DMSO-*d_6_*) *δ* 8.16 (d, 1H, *J* = 5.2 Hz), 7.96 (d, 1H, *J* = 5.2 Hz), 7.77 (d, 1H, *J* = 2.5 Hz), 7.67 (d, 1H, *J* = 9.0 Hz), 7.22 (dd, 1H, *J* = 8.9, 2.5 Hz), 4.54 (t, 2H, *J* = 7.2 Hz), 3.87 (s, 3H), 2.98 (s, 3H), 2.90 (t, 2H, *J* = 7.2 Hz); ^13^C NMR (DMSO-*d_6_*) *δ* 154.00, 141.95, 137.26, 136.74, 135.55, 128.04, 121.28, 118.28, 113.45, 111.96, 103.81, 56.14, 47.76, 42.75, 23.87; ESI-MS: *m*/*z* 256.20 (M + 1)^+^.

#### 3.1.6. General Procedure for the Synthesis of β-Carboline-Based Alkynes **15**–**17**, **25**, **26**, **30**–**32**

An appropriate β-carboline **6**–**8**, **18**, **20**, or **27**–**29** was dissolved in dry DMF (3 mL). Under an argon atmosphere, 60% dispersion of sodium hydride in mineral oil (60% NaH) (2.66 equivalents) or Cs_2_CO_3_ (1.4 equivalents; compounds **31** and **32**) was added, followed by a dropwise addition of 80% solution of propargyl bromide in toluene (3 equivalents). The reaction was stirred at r.t. and under an argon atmosphere for 2 h. Upon completion, the reaction mixture was poured into 30 mL of water. The product was extracted with ethyl acetate (5 × 30 mL). Organic layers were collected and washed with water and brine, dried over anhydrous sodium sulfate, and evaporated under reduced pressure. The crude product was purified by column chromatography (DCM:MeOH = 95:5) or cyclohexane:ethyl acetate:MeOH = 3:1:0.25 (compound **25**) and triturated with diethyl ether.

##### 7-Ethoxy-1-methyl-9-(prop-2-yn-1-yl)-9H-pyrido[3,4-b]indole (**15**)

Compound **6**: 0.160 g, 0.71 mmol; 60% NaH: 0.075 g, 1.88 mmol; 80% solution of propargyl bromide in toluene: 0.315 g, 2.12 mmol; yield: 0.102 g (55%); mp 137.5–140 °C; IR (ATR, *ν*/cm^−1^) 3270, 2980, 1626, 1564, 1445, 1394, 1305, 1254, 1208, 1189, 1110, 1046, 950, 927, 842, 810, 798, 697, 671, 632; ^1^H NMR (DMSO-*d_6_*) *δ* 8.19 (d, 1H, *J* = 5.2 Hz), 8.08 (d, 1H, *J* = 8.6 Hz), 7.87 (d, 1H, *J* = 5.3 Hz), 7.31 (d, 1H, *J* = 2.0 Hz), 6.89 (dd, 1H, *J* = 8.6, 2.1 Hz), 5.43 (d, 2H, *J* = 2.3 Hz), 4.19 (q, 2H, *J* = 7.0 Hz), 3.04 (s, 3H), 2.96 (s, 1H), 1.41 (d, 3H, *J* = 6.9 Hz); ^13^C NMR (DMSO-*d_6_*) *δ* 159.90, 142.57, 141.02, 138.48, 134.38, 128.91, 122.45, 114.33, 112.23, 109.90, 94.40, 75.49, 63.57, 34.16, 22.46, 14.64; ESI-MS: *m*/*z* 265.10 (M + 1)^+^.

##### 1-Methyl-9-(prop-2-yn-1-yl)-7-propoxy-9*H*-pyrido [3,4-*b*]indole (**16**)

Compound **7**: 0.140 g, 0.58 mmol; 60% NaH: 0.062 g, 1.55 mmol; 80% solution of propargyl bromide in toluene: 0.260 g, 1.75 mmol; yield: 0.077 g (47%); mp 135–136 °C; IR (ATR, *ν*/cm^−1^) 2960, 2106, 1622, 1566, 1443, 1335, 1279, 1251, 1184, 1141, 1119, 1050, 979; 926, 811, 730, 648; ^1^H NMR (DMSO-*d_6_*) *δ* 8.19 (d, 1H, *J* = 5.2 Hz), 8.08 (d, 1H, *J* = 8.6 Hz), 7.87 (d, 1H, *J* = 5.2 Hz), 7.32 (d, 1H, *J* = 1.9 Hz), 6.90 (dd, 1H, *J* = 8.5, 2.1 Hz), 5.44 (d, 2H, *J* = 2.2 Hz), 4.09 (t, 2H, *J* = 6.6 Hz), 3.36 (t, 1H, *J* = 2.2 Hz), 3.04 (s, 3H), 1.81 (h, 2H, *J* = 7.2 Hz), 1.04 (t, 3H, *J* = 7.4 Hz); ^13^C NMR (DMSO-*d_6_*) *δ* 160.58, 143.08, 141.53, 138.99, 134.88, 129.41, 122.95, 114.82, 112.74, 110.44, 94.91, 76.00, 69.97, 34.67, 22.97, 22.60, 10.97; ESI-MS: *m*/*z* 279.05 (M + 1)^+^.

##### 7-Isopropoxy-1-methyl-9-(prop-2-yn-1-yl)-9*H*-pyrido[3,4-*b*]indole (**17**)

Compound **8**: 0.160 g, 0.67 mmol; 60% NaH: 0.071 g, 1.77 mmol; 80% solution of propargyl bromide in toluene: 0.297 g, 2.00 mmol; yield: 0.098 g (47%); mp 134–137 °C; IR (ATR, *ν*/cm^−1^) 3116, 2974, 2106, 1632, 1571, 1496, 1444, 1373, 1348, 1299, 1282, 1218, 1183, 1134, 1109, 979, 827, 803, 671, 648; ^1^H NMR (DMSO-*d_6_*) *δ* 8.19 (d, 1H, *J* = 5.2 Hz), 8.07 (d, 1H, *J* = 8.6 Hz), 7.87 (d, 1H, *J* = 5.2 Hz), 7.31 (d, 1H, *J* = 1.7 Hz), 6.87 (dd, 1H, *J* = 8.6, 1.9 Hz), 5.43 (d, 2H, *J* = 2.0 Hz), 4.84 (hept, 1H, *J* = 6.0 Hz), 3.35 (t, 1H, *J* = 2.0 Hz), 3.04 (s, 3H), 1.35 (d, 6H, *J* = 6.0 Hz); ^13^C NMR (DMSO-*d_6_*) *δ* 159.26, 143.13, 141.50, 138.99, 134.92, 129.42, 123.01, 114.83, 112.74, 111.21, 96.22, 75.95, 70.07, 34.66, 23.00, 22.36; ESI-MS: *m*/*z* 279.10 (M + 1)^+^.

##### 6-Methoxy-1-methyl-9-(prop-2-yn-1-yl)-9*H*-pyrido[3,4-*b*]indole (**25**)

Compound **18**: 0.354 g, 1.67 mmol; 60% NaH: 0.177 g, 4.44 mmol; 80% solution of propargyl bromide in toluene: 0.744 g, 5.00 mmol yield: 0.051 g (10%); mp 137.5–145.5 °C; IR (ATR, *ν*/cm^−1^) 3162, 3014, 2111, 1585, 1564, 1489, 1452, 1375, 1333, 1289, 1229, 1123, 1043, 980, 928, 832, 816, 720, 619; ^1^H NMR (DMSO-*d_6_*) *δ* 8.21 (d, 1H, *J* = 5.2 Hz), 7.99 (d, 1H, *J* = 5.2 Hz), 7.80 (d, 1H, *J* = 2.5 Hz), 7.72 (d, 1H, *J* = 9.0 Hz), 7.26 (dd, 1H, *J* = 8.9, 2.5 Hz), 5.43 (d, 2H, *J* = 2.3 Hz), 3.87 (s, 3H), 3.04 (s, 3H); ^13^C NMR (DMSO-*d_6_*) *δ* 154.48, 142.36, 138.29, 136.33, 135.28, 128.92, 121.83, 118.45, 113.58, 111.89, 104.15, 76.00, 56.17, 34.80, 23.07; ESI-MS: *m*/*z* 251.15 (M + 1)^+^.

##### 1-Methyl-9-(prop-2-yn-1-yl)-6-propoxy-9*H*-pyrido[3,4-*b*]indole (**26**)

Compound **20**: 0.095 g, 0.40 mmol; 60% NaH: 0.042 g, 1.05 mmol; 80% solution of propargyl bromide in toluene: 0.176 g, 1.19 mmol; yield: 0.057 g (52%); mp 137.5–140.5 °C; IR (ATR, *ν*/cm^−1^) 2940, 2109, 1582, 1450, 1374, 1288, 1211, 1197, 990, 909, 878, 830, 816, 745, 622; ^1^H NMR (DMSO-*d_6_*) *δ* 8.21 (d, 1H, *J* = 5.2 Hz), 7.99 (d, 1H, *J* = 5.3 Hz), 7.80 (d, 1H, *J* = 2.4 Hz), 7.70 (d, 1H, *J* = 8.9 Hz), 7.25 (dd, 1H, *J* = 8.9, 2.5 Hz), 5.42 (d, 3H, *J* = 2.2 Hz), 4.04 (t, 3H, *J* = 6.5 Hz), 3.04 (s, 3H), 2.98 (s, 1H), 1.79 (h, 3H, *J* = 7.1 Hz), 1.03 (t, 4H, *J* = 7.4 Hz); ^13^C NMR (DMSO-*d_6_*) *δ* 152.76, 141.23, 137.21, 135.22, 134.20, 127.86, 120.79, 117.72, 112.52, 110.74, 103.99, 95.85, 85.40, 69.10, 33.71, 21.99, 21.61, 9.91; ESI-MS: *m*/*z* 267.20 (M + 1)^+^.

##### 1-Ethyl-9-(prop-2-yn-1-yl)-9*H*-pyrido[3,4-*b*]indole (**30**)

Compound **27**: 0.150 g, 0.76 mmol; 60% NaH: 0.081 g, 2.04 mmol; 80% solution of propargyl bromide in toluene: 0.341 g, 2.30 mmol; yield: 0.071 g (40%); mp 126–128 °C; IR (ATR, *ν*/cm^−1^) 3276, 1656, 1581, 1450, 1374, 1197, 991, 806, 623; ^1^H NMR (DMSO-*d_6_*) *δ* 8.34 (t, 1H, *J* = 4.9 Hz), 8.26 (t, 1H, *J* = 7.0 Hz), 8.02 (d, 1H, *J* = 5.1 Hz), 7.79 (dd, 1H, *J* = 17.9, 8.4 Hz), 7.66–7.58 (m, 1H), 7.32 (dt, 1H, *J* = 10.3, 7.5 Hz), 5.76 (d, 1H, *J* = 6.2 Hz), 5.44 (d, 1H, *J* = 2.4 Hz), 3.45–3.38 (m, 2H), 3.34 (s, 1H), 1.39 (dt, 3H, *J* = 32.3, 7.5 Hz); ^13^C NMR (DMSO-*d_6_*) *δ* 146.87, 141.47, 138.94, 134.04, 129.43, 128.84, 121.98, 121.52, 120.67, 113.34, 110.99, 96.44, 86.05, 35.05, 28.14, 13.75; ESI-MS: *m*/*z* 235.15 (M + 1)^+^.

##### 9-(Prop-2-yn-1-yl)-1-propyl-9*H*-pyrido[3,4-*b*]indole (**31**)

Compound **28**: 0.200 g, 0.95 mmol; Cs_2_CO_3_: 0.433 g, 1.33 mmol; 80% solution of propargyl bromide in toluene: 0.136 g, 1.14 mmol; yield: 0.131 g (55%); mp 89.5–90.5 °C; IR (ATR, *ν*/cm^−1^) 2968, 1612, 1579, 1452, 1430, 1330, 1227, 1200, 1140, 1047, 875, 815, 746; ^1^H NMR (DMSO-*d_6_*) *δ* 8.33 (d, 1H, *J* = 5.1 Hz), 8.25 (d, 1H, *J* = 7.7 Hz), 8.02 (d1H, *J* = 5.0 Hz), 7.79 (d, 1H, *J* = 8.3 Hz), 7.63 (t, 1H, *J* = 7.6 Hz), 7.31 (t, 1H, *J* = 7.4 Hz), 5.41 (d, 2H, *J* = 1.62 Hz), 3.36 (t, 3H, *J* = 7.8 Hz), 1.90 (h, 2H, *J* = 7.4 Hz), 1.05 (t, 3H, *J* = 7.3 Hz); ^13^C NMR (DMSO-*d_6_*) *δ* 145.90, 141.51, 138.93, 134.18, 129.59, 128.87, 121.98, 121.52, 120.69, 113.33, 110.99, 76.10, 37.07, 35.04, 22.62, 14.49; ESI-MS: *m*/*z* 249.20 (M + 1)^+^.

##### 1-Isopropyl-9-(prop-2-yn-1-yl)-9*H*-pyrido[3,4-*b*]indole (**32**)

Compound **29**: 0.150 g, 0.71 mmol; Cs_2_CO_3_: 0.325 g, 1.00 mmol; 80% solution of propargyl bromide in toluene: 0.102 g, 0.86 mmol; yield: 0.052 g (29%); mp 89.5–91.5 °C; IR (ATR, *ν*/cm^−1^); ^1^H NMR (DMSO-*d_6_*) *δ* 8.38 (d, 1H, *J* = 5.0 Hz), 8.25 (d, 1H, *J* = 7.7 Hz), 8.02 (d, 1H, *J* = 5.1 Hz), 7.80 (d, 1H, *J* = 8.4 Hz), 7.63 (ddd, 1H, *J* = 8.4, 7.1, 1.3 Hz), 7.31 (ddd, 1H, *J* = 7.9, 7.0, 0.9 Hz), 5.43 (d, 2H, *J* = 2.4 Hz), 3.97 (hept, 1H, *J* = 6.6 Hz), 3.37 (t, 1H, *J* = 2.4 Hz), 1.42 (d, 6H, *J* = 6.6 Hz); ^13^C NMR (DMSO-*d_6_*) *δ* 150.40, 141.13, 138.47, 132.46, 129.37, 128.31, 121.32, 120.96, 120.10, 112.57, 110.40, 34.86, 30.73, 22.70; ESI-MS: *m*/*z* 249.20 (M + 1)^+^.

#### 3.1.7. General Procedure for the Synthesis of AT Harmiquins **33**–**37**

A suspension of a 7-chloroquinoline-based carboxylic acid **4**, amine **12**–**14, 22** or **24** (1.1 equivalents), and TEA (2 equivalents) in dry DMF (1 mL) was stirred at room temperature for 10 min, followed by the dropwise addition of T3P (≥50% in ethyl acetate, 1 equivalent). The reaction mixture was stirred at room temperature for 18 h. Afterwards, the reaction mixture was placed in an ultrasonic bath, and 5% NaOH was added dropwise until the formation of white precipitate was completed. The formed precipitate was filtered off and the crude product was purified by column chromatography (DCM/MeOH 85:15) and trituration with diethyl ether.

##### 2-((7-Chloroquinolin-4-yl)amino)-*N*-(2-(7-ethoxy-1-methyl-9*H*-pyrido[3,4-*b*]indol-9-yl)ethyl)acetamide (**33**)

Acid **4**: 0.050 g, 0.21 mmol; amine **12**: 0.063 g, 0.23 mmol; TEA: 0.043 g, 0.42 mmol; T3P: 0.067 g, 0.21 mmol; yield: 0.052 g (46%); mp 204–205 °C; IR (ATR, *ν*/cm^−1^) 3269, 2926, 1657, 1623, 1580, 1499, 1444, 1339, 1279, 1248, 1186, 1140, 1112, 1049, 975, 850, 804, 765, 732, 638; ^1^H NMR (DMSO-*d_6_*) *δ* 8.38 (t, 1H, *J* = 5.5 Hz), 8.32 (d, 1H, *J* = 5.3 Hz), 8.26 (d, 1H, *J* = 9.0 Hz), 8.16 (d, 1H, *J* = 5.1 Hz), 8.09 (d, 1H, *J* = 8.5 Hz), 7.87 (d, 1H, *J* = 5.0 Hz), 7.82 (d, 1H, *J* = 2.3 Hz), 7.80 (d, 1H, *J* = 6.4 Hz), 7.50 (dd, 1H, *J* = 8.9, 2.3 Hz), 7.25 (s, 1H), 6.88 (d, 1H, *J* = 8.4 Hz), 6.10 (d, 1H, *J* = 5.3 Hz), 4.56 (t, 2H, *J* = 6.8 Hz), 4.15 (q, 2H, *J* = 6.8 Hz), 3.87 (d, 1H, *J* = 5.5 Hz), 3.51 (q, 1H, *J* = 6.9 Hz), 2.96 (s, 1H), 1.37 (t, 2H, *J* = 6.9 Hz); ^13^C NMR (DMSO-*d_6_*) *δ* 169.96, 160.30, 152.05, 150.79, 149.06, 143.38, 140.99, 138.21, 135.08, 134.09, 128.96, 127.70, 124.88, 124.71, 122.91, 117.95, 114.70, 112.72, 109.90, 99.45, 94.70, 63.95, 46.41, 43.74, 39.25, 23.50, 15.14; ESI-MS: *m*/*z* 488.20 (M + 1)^+^.

##### 2-((7-Chloroquinolin-4-yl)amino)-*N*-(2-(1-methyl-7-propoxy-9*H*-pyrido[3,4-*b*]indol-9-yl)ethyl)acetamide (**34**)

Acid **4**: 0.050 g, 0.21 mmol; amine **13**: 0.066 g, 0.23 mmol; TEA: 0.043 g, 0.42 mmol; T3P: 0.067 g, 0.21 mmol; yield: 0.021 g (20%); mp 232.5–234.5 °C; IR (ATR, *ν*/cm^−1^) 2963, 1656, 1623, 1581, 1444, 1340, 1278, 1248, 1188, 1140, 1048, 988, 851, 798, 637; ^1^H NMR (DMSO-*d_6_*) *δ* 8.38 (t, 1H, *J* = 6.1 Hz), 8.33 (d, 1H, *J* = 5.4 Hz), 8.25 (d, 1H, *J* = 9.0 Hz), 8.16 (d, 1H, *J* = 5.2 Hz), 8.08 (d, 1H, *J* = 8.6 Hz), 7.86 (d, 1H, *J* = 5.2 Hz), 7.82 (d, 1H, *J* = 2.3 Hz), 7.79 (t, 1H, *J* = 6.1 Hz), 7.50 (dd, 1H, *J* = 9.0, 2.3 Hz), 7.26 (d, 1H, *J* = 2.1 Hz), 6.88 (dd, 1H, *J* = 8.6, 2.1 Hz), 6.12 (d, 1H, *J* = 5.4 Hz), 4.83–4.77 (m, 1H), 4.55 (t, 2H, *J* = 7.2 Hz), 3.88 (d, 2H, *J* = 5.9 Hz), 3.49 (q, 2H, J = 7.2 Hz), 2.96 (s, 3H), 1.32 (d, 6H, *J* = 6.0 Hz); ^13^C NMR (DMSO-*d_6_*) *δ* 169.97, 159.15, 152.15, 150.75, 149.17, 143.37, 140.99, 138.23, 135.14, 134.05, 128.92, 127.79, 124.87, 124.69, 122.98, 117.98, 114.74, 112.72, 110.61, 99.48, 96.17, 70.08, 46.39, 43.73, 39.15, 23.52, 22.39; ESI-MS: *m*/*z* 502.2 (M + 1)^+^.

##### 2-((7-Chloroquinolin-4-yl)amino)-*N*-(2-(7-isopropoxy-1-methyl-9*H*-pyrido[3,4-*b*]indol-9-yl)ethyl)acetamide (**35**)

Acid **4**: 0.028 g, 0.12 mmol; amine **14**: 0.037 g, 0.13 mmol; TEA: 0.024 g, 0.24 mmol; T3P: 0.038 g, 0.12 mmol; yield: 0.022 g (37%); mp 249.5–255.5 °C; IR (ATR, *ν*/cm^−1^) 3384, 3198, 2975, 1684, 1624, 1586, 1526, 1449, 1388, 1334, 1281, 1232, 1198, 1114, 1081, 1025, 986, 935, 857, 821, 810, 759, 739, 643; ^1^H NMR (DMSO-*d_6_*) *δ* 8.38 (t, 1H, *J* = 6.1 Hz), 8.33 (d, 1H, *J* = 5.4 Hz), 8.25 (d, 1H, *J* = 9.0 Hz), 8.16 (d, 1H, *J* = 5.2 Hz), 8.08 (d, 1H, *J* = 8.6 Hz), 7.86 (d, 1H, *J* = 5.2 Hz), 7.82 (d, 1H, *J* = 2.3 Hz), 7.79 (t, 1H, *J* = 6.1 Hz), 7.50 (dd, 1H, *J* = 9.0, 2.3 Hz), 7.26 (d, 1H, *J* = 2.1 Hz), 6.88 (dd, 1H, *J* = 8.6, 2.1 Hz), 6.12 (d, 1H, *J* = 5.4 Hz), 4.83–4.77 (m, 1H), 4.55 (t, 2H, *J* = 7.2 Hz), 3.88 (d, 2H, *J* = 5.9 Hz), 3.49 (q, 2H, J = 7.2 Hz), 2.96 (s, 3H), 1.32 (d, 6H, *J* = 6.0 Hz); ^13^C NMR (DMSO-*d_6_*) *δ* 169.97, 159.15, 152.15, 150.75, 149.17, 143.37, 140.99, 138.23, 135.14, 134.05, 128.92, 127.79, 124.87, 124.69, 122.98, 117.98, 114.74, 112.72, 110.61, 99.48, 96.17, 70.08, 46.39, 43.73, 39.15, 23.52, 22.39; ESI-MS: *m*/*z* 502.2 (M + 1)^+^.

##### 2-((7-Chloroquinolin-4-yl)amino)-*N*-(2-(6-methoxy-1-methyl-9*H*-pyrido[3,4-*b*]indol-9-yl)ethyl)acetamide (**36**)

Acid **4**: 0.046 g, 0.20 mmol; amine **24**: 0.055 g, 0.22 mmol; TEA: 0.040 g, 0.40 mmol; T3P: 0.062 g, 0.20 mmol; yield: 0.037 g (40%); mp 219.5–222.5 °C; IR (ATR, *ν*/cm^−1^) 3272, 2920, 1666, 1611, 1489, 1408, 1374, 1226, 1200, 1178, 1126, 1048, 1022, 983, 899, 850, 806, 703, 670, 619; ^1^H NMR (DMSO-*d_6_*) *δ* 8.35 (d, 1H, *J* = 6.0 Hz), 8.33 (d, 1H, *J* = 5.5 Hz), 8.25 (d, 1H, *J* = 9.0 Hz), 8.18 (d, 1H, *J* = 5.2 Hz), 7.98 (d, 1H, *J* = 5.2 Hz), 7.82 (d, 1H, *J* = 2.3 Hz), 7.80 (d, 1H, *J* = 2.7 Hz), 7.65 (d, 1H, *J* = 9.0 Hz), 7.50 (dd, 1H, *J* = 9.0, 2.3 Hz), 7.23 (dd, 1H, *J* = 9.0, 2.5 Hz), 6.09 (d, 1H, *J* = 5.5 Hz), 4.60 (t, 2H, *J* = 7.0 Hz), 3.87 (s, 3H), 3.85 (d, 2H, *J* = 5.9 Hz), 3.48 (q, 2H, *J* = 6.6 Hz), 2.97 (s, 3H); ^13^C NMR (DMSO-*d_6_*) *δ* 169.84, 154.15, 151.97, 150.83, 148.98, 141.87, 137.46, 136.67, 135.37, 134.12, 128.34, 127.63, 124.90, 124.71, 121.45, 118.40, 117.93, 113.53, 111.61, 103.97, 99.46, 56.15, 46.29, 43.92, 40.41, 23.64; ESI-MS: *m*/*z* 474.30 (M + 1)^+^.

##### 2-((7-Chloroquinolin-4-yl)amino)-*N*-(2-(1-methyl-6-propoxy-9*H*-pyrido[3,4-*b*]indol-9-yl)ethyl)acetamide (**37**)

Acid **4**: 0.049 g, 0.21 mmol; amine **22**: 0.065 g, 0.23 mmol; TEA: 0.042 g, 0.42 mmol; T3P: 0.066 g, 0.21 mmol; yield: 0.017 g (16%); mp 238.5–245.5 °C; IR (ATR, *ν*/cm^−1^) 3273, 2964, 1656, 1611, 1582, 1488, 1450, 1374, 1225, 1196, 991, 850, 802, 766, 618; ^1^H NMR (DMSO-*d_6_*) *δ* 8.33 (d, 2H, *J* = 4.7 Hz), 8.24 (d, 1H, *J* = 9.0 Hz), 8.17 (d, 1H, *J* = 5.2 Hz), 7.97 (d, 1H, *J* = 5.1 Hz), 7.81 (d, 1H, *J* = 1.9 Hz), 7.79 (d, 1H, *J* = 2.2 Hz), 7.73 (t, 1H, *J* = 5.7 Hz), 7.63 (d, 1H, *J* = 9.0 Hz), 7.49 (dd, 1H, *J* = 9.0, 2.0 Hz), 7.23 (dd, 1H, *J* = 8.9, 2.3 Hz), 6.08 (d, 1H, *J* = 5.4 Hz), 4.59 (t, 2H, *J* = 6.9 Hz), 4.03 (t, 2H, *J* = 6.5 Hz), 3.84 (d, 2H, *J* = 5.8 Hz), 3.48 (q, 3H, *J* = 6.5 Hz), 2.97 (s, 3H), 1.79 (h, 2H, *J* = 7.1 Hz), 1.03 (t, 3H, *J* = 7.4 Hz); ^13^C NMR (DMSO-*d_6_*) *δ* 169.91, 153.50, 152.25, 150.65, 149.30, 141.84, 137.50, 136.65, 135.38, 134.00, 128.35, 127.89, 124.83, 124.66, 121.50, 118.75, 117.99, 113.55, 111.55, 104.89, 99.47, 70.17, 46.31, 43.91, 40.42, 23.66, 22.71, 11.01; ESI-MS: *m*/*z* 502.20 (M + 1)^+^.

#### 3.1.8. General Procedure for the Synthesis of TT Harmiquins **38**–**45**

To a solution of an alkyne and the 7-chloroquinoline-based azide **3** (1.1 equivalents) in MeOH (5 mL), a catalytic amount of Cu(OAc)_2_ was added. The reaction mixture was stirred at rt for 24 h. Upon completion of the reaction, the solvent was removed under reduced pressure. The residue was purified by column chromatography (with an additional Al_2_O_3_ layer in order to remove Cu salts) with DCM:MeOH=85:15 as a mobile phase. The crude product was triturated with diethyl ether or MeOH (compounds **44** and **45**).

##### 7-Chloro-*N*-(2-(4-((7-ethoxy-1-methyl-9*H*-pyrido[3,4-*b*]indol-9-yl)methyl)-1*H*-1,2,3-triazol-1-yl)ethyl)quinolin-4-amine (**38**)

Alkyne **15**: 0.060 g, 0.23 mmol; azide **3**: 0.062 g, 0.25 mmol; yield: 0.067 g (58%); mp 227.5–230 °C; IR (ATR, *ν*/cm^−1^) 2972, 1622, 1579, 1444, 1408, 1324, 1250, 1181, 1140, 1047, 954, 910, 876, 814, 765, 641; ^1^H NMR (DMSO-*d_6_*) *δ* 8.31 (d, 1H, *J* = 5.4 Hz), 8.16 (d, 1H, *J* = 5.2 Hz), 8.06 (d, 1H, *J* = 8.6 Hz), 8.03 (d, 1H, *J* = 9.1 Hz), 7.97 (s, 1H), 7.86 (d, 1H, *J* = 5.2 Hz), 7.79 (d, 1H, *J* = 2.2 Hz), 7.39 (dd, 1H, *J* = 9.0, 2.2 Hz), 7.37 (d, 1H, *J* = 6.0 Hz), 7.24 (d, 1H, *J* = 2.1 Hz), 6.85 (dd, 1H, *J* = 8.6, 2.1 Hz), 6.45 (d, 1H, *J* = 5.4 Hz), 5.82 (s, 2H), 4.57 (t, 2H, *J* = 6.0 Hz), 4.08 (q, 2H, *J* = 7.0 Hz), 3.69 (q, 2H, *J* = 5.8 Hz), 2.98 (s, 3H), 1.35 (t, 3H, *J* = 6.9 Hz); ^13^C NMR (DMSO-*d_6_*) *δ* 159.74, 151.59, 149.70, 148.70, 143.85, 142.62, 141.00, 137.92, 134.56, 133.52, 128.48, 127.30, 124.31, 123.72, 123.41, 122.29, 117.29, 114.27, 112.17, 109.60, 98.74, 94.43, 63.42, 47.84, 42.32, 23.07, 14.58; ESI-MS: *m*/*z* 512.20 (M + 1)^+^.

##### 7-Chloro-*N*-(2-(4-((1-methyl-7-propoxy-9*H*-pyrido[3,4-*b*]indol-9-yl)methyl)-1*H*-1,2,3-triazol-1-yl)ethyl)quinolin-4-amine (**39**)

Alkyne **16**: 0.060 g, 0.22 mmol; azide **3**: 0.059 g, 0.24 mmol; yield: 0.082 g (72%); mp 245.5–249 °C; IR (ATR, *ν*/cm^−1^) 3204, 2964, 1622, 1578, 1498, 1445, 1429, 1366, 1341, 1323, 1181, 1140, 1081, 1048, 982, 935, 910, 877, 815, 766, 732, 640; ^1^H NMR (DMSO-*d_6_*) *δ* 8.31 (d, 1H, *J* = 5.4 Hz), 8.17 (d, 1H, *J* = 5.2 Hz), 8.07 (d, 1H, *J* = 8.6 Hz), 8.03 (d, 1H, *J* = 9.1 Hz), 7.96 (s, 1H), 7.86 (d, 1H, *J* = 5.2 Hz), 7.78 (d, 1H, *J* = 2.1 Hz), 7.39 (dd, 1H, *J* = 9.0, 2.2 Hz), 7.36 (d, 1H, *J* = 5.6 Hz), 7.25 (d, 1H, *J* = 1.8 Hz), 6.87 (dd, 1H, *J* = 8.6, 2.0 Hz), 6.45 (d, 1H, *J* = 5.5 Hz), 5.83 (s, 2H), 4.57 (t, 2H, *J* = 6.0 Hz), 3.99 (t, 2H, *J* = 6.6 Hz), 3.69 (q, 2H, *J* = 5.8 Hz), 2.97 (s, 3H), 1.76 (h, 2H, *J* = 7.1 Hz), 0.99 (t, 3H, *J* = 7.4 Hz); ^13^C NMR (DMSO-*d_6_*) *δ* 159.33, 151.06, 149.10, 148.17, 143.36, 142.07, 140.42, 137.36, 134.00, 132.93, 127.92, 126.77, 123.72, 123.14, 122.78, 121.71, 116.73, 113.70, 111.60, 109.05, 98.17, 93.89, 68.73, 47.27, 41.75, 22.49, 21.47, 9.87; ESI-MS: *m*/*z* 526.15 (M + 1)^+^.

##### 7-Chloro-*N*-(2-(4-((7-isopropoxy-1-methyl-9*H*-pyrido[3,4-*b*]indol-9-yl)methyl)-1*H*-1,2,3-triazol-1-yl)ethyl)quinolin-4-amine (**40**)

Alkyne **17**: 0.060 g, 0.22 mmol; azide **3**: 0.059 g, 0.24 mmol; yield: 0.054 g (47%); mp 234–236 °C; IR (ATR, *ν*/cm^−1^) 2974, 1622, 1578, 1445, 1408, 1324, 1182, 1139, 1111, 1047, 909, 875, 803; ^1^H NMR (DMSO-*d_6_*) *δ* 8.31 (d, J = 5.4 Hz, 1H), 8.16 (d, J = 5.2 Hz, 1H), 8.06 (d, J = 4.8 Hz, 1H), 8.05 (d, J = 5.1 Hz, 1H), 7.97 (s, 1H), 7.86 (d, J = 5.1 Hz, 1H), 7.79 (d, J = 1.7 Hz, 1H), 7.41 (d, J = 8.8 Hz, 1H), 7.25 (d, J = 1.9 Hz, 1H), 5.82–5.80 (m, 2H), 6.84 (dd, J = 8.6, 2.0 Hz, 1H), 6.45 (d, J = 5.3 Hz, 1H), 5.81 (s, 2H), 4.75 (h, J = 6.0 Hz, 1H), 4.57 (t, J = 6.0 Hz, 2H), 3.70 (q, J = 5.8 Hz, 2H), 2.96 (s, 3H), 1.28 (d, J = 6.0 Hz, 6H); ^13^C NMR (DMSO-*d_6_*) *δ* 158.58, 151.38, 149.84, 148.46, 143.94, 142.72, 140.90, 137.85, 134.60, 133.63, 128.52, 127.10, 124.39, 123.80, 123.31, 122.36, 117.26, 114.27, 112.18, 110.39, 98.72, 95.88, 69.48, 47.83, 42.33, 23.03, 21.80; ESI-MS: *m*/*z* 526.25 (M + 1)^+^.

##### 7-Chloro-*N*-(2-(4-((6-methoxy-1-methyl-9*H*-pyrido[3,4-*b*]indol-9-yl)methyl)-1*H*-1,2,3-triazol-1-yl)ethyl)quinolin-4-amine (**41**)

Alkyne **25**: 0.030 g, 0.12 mmol; azide **3**: 0.033 g, 0.13 mmol; yield: 0.028 g (46%); mp 245.5–250.5 °C; IR (ATR, *ν*/cm^−1^) 3143, 3061, 2958, 1611, 1579, 1487, 1448, 1431, 1402, 1370, 1324, 1225, 1195, 1140, 1083, 1083, 1046, 1022, 980, 909, 876, 818, 806, 734, 725, 690, 609; ^1^H NMR (DMSO-*d_6_*) *δ* 8.31 (d, 1H, *J* = 5.4 Hz), 8.18 (d, 1H, *J* = 5.2 Hz), 8.04 (d, 1H, *J* = 9.1 Hz), 7.97 (d, 1H, *J* = 5.2 Hz), 7.93 (s, 1H), 7.79 (d, 1H, *J* = 2.2 Hz), 7.77 (d, 1H, *J* = 2.5 Hz), 7.64 (d, 1H, *J* = 9.0 Hz), 7.42 (dd, 1H, *J* = 9.0, 2.2 Hz), 7.40 (d, 1H, *J* = 5.5 Hz), 7.14 (dd, 1H, *J* = 9.0, 2.5 Hz), 6.44 (d, 1H, *J* = 5.5 Hz), 5.82 (s, 2H), 4.55 (t, 2H, *J* = 6.0 Hz), 3.86 (s, 3H), 3.69 (q, 2H, *J* = 5.9 Hz), 2.99 (s, 3H); ^13^C NMR (DMSO-*d_6_*) *δ* 154.25, 152.03, 150.24, 149.13, 144.47, 142.30, 137.69, 136.38, 135.39, 134.08, 128.37, 127.74, 124.86, 124.32, 123.86, 121.64, 118.27, 117.79, 113.50, 111.98, 103.96, 99.22, 56.14, 48.35, 42.76, 23.68; ESI-MS: *m*/*z* 498.20 (M + 1)^+^.

##### 7-Chloro-*N*-(2-(4-((1-methyl-6-propoxy-9*H*-pyrido[3,4-*b*]indol-9-yl)methyl)-1*H*-1,2,3-triazol-1-yl)ethyl)quinolin-4-amine (**42**)

Alkyne **26**: 0.045 g, 0.16 mmol; azide **3**: 0.044 g, 0.18 mmol; yield: 0.034 g (40%); mp 205–206 °C; IR (ATR, *ν*/cm^−1^) 3207, 2969, 1610, 1577, 1488, 1448, 1404, 1281, 1241, 1194, 1139, 1047, 990, 908, 880, 820, 807, 666, 639, 622; ^1^H NMR (DMSO-*d_6_*) *δ* 8.31 (d, *J* = 5.4 Hz, 1H), 8.17 (d, *J* = 5.2 Hz, 1H), 8.04 (d, *J* = 9.1 Hz, 1H), 7.96 (d, *J* = 5.2 Hz, 1H), 7.93 (s, 1H), 7.79 (d, *J* = 2.2 Hz, 1H), 7.77 (d, *J* = 2.5 Hz, 1H), 7.61 (d, *J* = 9.0 Hz, 1H), 7.43 (dd, *J* = 9.0, 2.2 Hz, 1H), 7.40 (d, *J* = 5.7 Hz, 1H), 7.14 (dd, *J* = 8.9, 2.5 Hz, 1H), 6.44 (d, *J* = 5.5 Hz, 1H), 5.81 (s, 2H), 4.55 (t, *J* = 6.0 Hz, 2H), 4.02 (t, *J* = 6.5 Hz, 2H), 3.69 (q, *J* = 5.9 Hz, 3H), 2.99 (s, 3H), 1.79 (h, *J* = 7.3 Hz, 2H), 1.03 (t, *J* = 7.4 Hz, 3H); ^13^C NMR (DMSO-*d_6_*) *δ* 153.61, 150.26, 149.11, 144.47, 142.25, 137.68, 136.35, 135.40, 134.09, 128.40, 127.73, 124.86, 124.33, 123.87, 121.69, 118.64, 117.79, 113.53, 111.91, 104.91, 99.22, 70.17, 48.37, 42.77, 23.67, 22.71, 11.01; ESI-MS: *m*/*z* 526.20 (M + 1)^+^.

##### 7-Chloro-*N*-(2-(4-((1-ethyl-9*H*-pyrido[3,4-*b*]indol-9-yl)methyl)-1*H*-1,2,3-triazol-1-yl)ethyl)quinolin-4-amine (**43**)

Alkyne **30**: 0.060 g, 0.26 mmol; azide **3**: 0.069 g, 0.28 mmol; yield: 0.054 g (44%); mp 192.5–194.5 °C; IR (ATR, *ν*/cm^−1^) 2969, 1612, 1579, 1450, 1370, 1326, 1197, 1141, 1047, 875, 806, 744; ^1^H NMR (DMSO-*d_6_*) *δ* 8.32 (d, 1H, *J* = 6.96 Hz), 8.30 (d, 1H, *J* = 5.1 Hz), 8.23 (d, 1H, *J* = 7.8 Hz), 8.05 (d, 1H, *J* = 9.0 Hz), 8.00 (d, 1H, *J* = 5.1 Hz), 7.93 (s, 1H), 7.80 (d, 1H, *J* = 1.7 Hz), 7.70 (d, 1H, *J* = 8.4 Hz), 7.55–7.49 (m, 1H), 7.42 (dd, 2H, *J* = 8.9, 2.1 Hz), 7.32–7.24 (m, 1H), 6.44 (d, 1H, *J* = 5.4 Hz), 5.82 (s, 2H), 4.55 (t, 2H, *J* = 6.0 Hz), 3.70 (q, 2H, *J* = 5.9 Hz), 3.35 (q, 2H, *J* = 7.4 Hz), 1.30 (t, 2H, *J* = 7.4 Hz); ^13^C NMR (DMSO-*d_6_*) *δ* 151.96, 150.28, 149.07, 146.77, 144.26, 141.52, 138.39, 134.29, 134.10, 128.95, 128.61, 127.69, 124.88, 124.33, 123.87, 121.84, 121.39, 120.28, 117.79, 113.25, 111.03, 99.22, 48.37, 42.76, 40.67, 28.29, 13.60; ESI-MS: *m*/*z* 482.20 (M + 1)^+^.

##### 7-Chloro-N-(2-(4-((1-propyl-9H-pyrido[3,4-b]indol-9-yl)methyl)-1H-1,2,3-triazol-1-yl)ethyl)quinolin-4-amine (**44**)

Alkyne **31**: 0.070 g, 0.28 mmol; azide **3**: 0.077 g, 0.31 mmol; yield: 0.063 g (45%); mp 205–209 °C; IR (ATR, *ν*/cm^−1^) 2964, 1611, 1581, 1452, 1368, 1324, 1196, 1142, 1047, 875, 738; ^1^H NMR (DMSO-*d_6_*) *δ* 8.31 (d, 1H, *J* = 4.8 Hz), 8.29 (d, 1H, *J* = 5.0 Hz), 8.23 (d, 1H, *J* = 7.7 Hz), 8.04 (d, 1H, *J* = 9.0 Hz), 8.00 (d, 1H, *J* = 5.0 Hz), 7.91 (s, 1H), 7.79 (d, 1H, *J* = 2.3 Hz), 7.70 (d, 1H, *J* = 8.3 Hz), 7.52 (t, 1H, *J* = 7.6 Hz), 7.45–7.39 (m, 1H), 7.38 (t, 1H, *J* = 5.8 Hz), 7.27 (t, 1H, *J* = 7.4 Hz), 6.44 (d, 1H, *J* = 5.3 Hz), 5.80 (s, 1H), 4.55 (t, 2H, *J* = 5.7 Hz), 3.69 (q, 1H, *J* = 5.9 Hz), 3.26 (t, 2H, *J* = 7.62 Hz), 1.77 (h, 2H, *J* = 7.2 Hz), 0.93 (t, 2H, *J* = 7.3 Hz); ^13^C NMR (DMSO-*d_6_*) *δ* 151.51, 149.62, 148.63, 145.25, 143.66, 141.00, 137.82, 133.80, 133.45, 128.57, 128.07, 127.23, 124.25, 123.70, 123.24, 121.26, 120.80, 119.71, 117.22, 112.65, 110.44, 98.64, 47.77, 42.19, 40.09, 36.65, 22.05, 13.79; ESI-MS: *m*/*z* 496.20 M + 1)^+^.

##### 7-Chloro-N-(2-(4-((1-isopropyl-9H-pyrido[3,4-b]indol-9-yl)methyl)-1H-1,2,3-triazol-1-yl)ethyl)quinolin-4-amine (**45**)

Alkyne **32**: 0.040 g, 0.16 mmol; azide **3**: 0.044 g, 0.18 mmol; yield: 0.033 g (41%); mp 189–192 °C; IR (ATR, *ν*/cm^−1^) 2963, 1582, 1450, 1328, 1219, 1198, 1086, 1039, 872, 798, 739; ^1^H NMR (DMSO-*d_6_*) *δ* 8.33 (d, 1H, *J* = 5.0 Hz), 8.24 (d, 1H, *J* = 7.8 Hz), 8.03 (d, 1H, *J* = 9.0 Hz), 8.00 (d, 1H, *J* = 5.1 Hz), 7.90 (s, 1H), 7.79 (s, 1H), 7.70 (d, 1H, *J* = 8.4 Hz), 7.53 (ddd, 1H, *J* = 8.3, 7.0, 1.2 Hz), 7.40 (dd, 1H, *J* = 8.9, 1.3 Hz), 7.33 (t, 1H, *J* = 5.5 Hz), 7.27 (t, 1H, *J* = 7.4 Hz), 6.44 (d, 1H, *J* = 4.9 Hz), 5.82 (s, 2H), 4.56 (t, 2H, *J* = 6.0 Hz), 4.09 (q, 1H, *J* = 5.3 Hz), 3.96–3.91 (m, 1H), 3.68 (q, 2H, *J* = 5.9 Hz), 3.17 (d, 1H, *J* = 5.3 Hz), 1.25 (d, 5H, *J* = 6.6 Hz).; ^13^C NMR (DMSO-*d_6_*) *δ* 150.23, 149.45, 143.64, 141.21, 137.91, 133.32, 132.73, 128.87, 128.10, 124.18, 123.72, 123.19, 121.18, 120.80, 119.70, 112.46, 110.37, 47.74, 42.22, 40.53, 30.48, 22.45; ESI-MS: *m*/*z* 496.25 M + 1)^+^.

### 3.2. SwissADME Prediction

SwissADME, an open-access software was used to predict some physicochemical properties of the title compounds [52].

### 3.3. In Vitro Drug Sensitivity Assay Against Erythrocytic Stages of P. falciparum

Antiplasmodial activity of harmiquins 18–32 was evaluated against two laboratory P. falciparum strains (3D7–CQ-sensitive and Dd2—multi-drug resistant), as previously described, using the histidine-rich protein 2 (HRP2) assay [40,41]. Briefly, 96-well plates were pre-coated with the tested compounds in a three-fold dilution before ring-stage parasites were added to the complete culture medium at a haematocrit of 1.5% and a parasitaemia of 0.05%. After three days of incubation at 37 °C, 5% CO_2_, and 5% oxygen, plates were frozen until analysed by HRP2-ELISA. All compounds were evaluated in duplicate in at least two independent experiments. The IC_50_ was determined by non-linear regression analysis of log concentration-response curves using the drc-package v0.9.0 of R v2.6.1 [42].

### 3.4. In Vitro Cytotoxicity Assay

Cytotoxicity against a human cell line (HepG2) was evaluated using the neutral red assay [53] with minor modifications [54]. In brief, human cells were seeded to a 96-well plate in a complete culture medium; on the following day, a serial dilution of the respective compound was added. After one day of incubation, cytotoxicity was evaluated by the addition of neutral red, subsequent lysis of cells, and measurement of absorbance (540 nm) in a plate reader (CLARIOstar, BMG Labtech). The IC_50_ was determined similarly to the in vitro drug assay against *P. falciparum*. To assess the safety of a compound, SI was calculated as the fractional ratio between the IC_50_s for HepG2 and *P. falciparum* 3D7 strain.

### 3.5. Spectroscopic Experiments

The electronic absorption (UV/vis) spectra were recorded on a Varian Cary 100 Bio spectrophotometer (Varian, Inc., Palo Alto, CA, USA). Fluorescence measurements were performed on a Varian Cary Eclipse fluorimeter (Varian, Inc., CA, USA), taking care that the absorbance of a sample at the excitation wavelength was > 0.05 to avoid any impact of the inner filter effect. CD spectra were recorded on a JASCO J815 spectropolarimeter (JASCO, Tokyo, Japan). All spectrophotometric measurements were performed using an appropriate quartz cuvette (Hellma Suprasil QX, path length: 1 cm) in aqueous buffer solution (sodium cacodylate buffer, pH = 7.0, *I* = 0.05 M) at room temperature (25 °C).

### 3.6. Study of DNA/RNA and HSA Interactions

Polynucleotides were purchased as noted: calf thymus (ct) DNA, poly dAdT–poly dAdT, poly dGdC–poly dGdC, and poly A–poly U (Sigma Aldrich, St. Louis, MO, USA), and dissolved in sodium cacodylate buffer (*I* = 0.05 M, pH = 7.0).

Human serum albumin was purchased from Sigma-Aldrich (Sigma-Aldrich, St. Louis, MO, USA) supplier, and dissolved in milli Q (MQ) water.

The presence of a double-stranded helix was confirmed by a single, well-defined transition in the thermal denaturation experiment, giving a Tm value agreeing well with the literature, as well as by collecting CD spectra of free ds-DNA or ds-RNA, which also agreed well with the literature [48,49]. The ct-DNA was additionally sonicated and filtered through a 0.45 mm filter to obtain mostly short (ca. 100 base pairs) rod-like B-helical DNA fragments. Polynucleotide concentration was determined spectroscopically according to producer data as the concentration of phosphates (corresponds to c(nucleobase)) [55].

Fluorimetric titration experiments were performed by adding aliquots of the polynucleotide stock solution into the solution of the studied dye. Titration data were processed by the Scatchard equation, yielding values for *Ks* and *n*, all fitting data having satisfactory correlation coefficients (>0.999). CD experiments were performed by adding the aliquots of the compound stock solution into the solution of polynucleotide (*c* = 2 × 10^–5^ M), and spectra were recorded with a scanning speed of 200 nm/min (an average of three accumulations). In both sets of experiments (fluorimetric and CD titrations), a buffer background was subtracted from each spectrum. Every reported spectrophotometric titration was the average of at least two measurements.

Thermal denaturation curves for ds-DNA, ds-RNA, and their complexes with studied compounds were determined as previously described [50] by following the absorption change at 260 nm as a function of temperature. The absorbance of the ligands was subtracted from every curve, and the absorbance scale was normalised. The *T*_m_ values are the midpoints of the transition curves determined from the maximum of the first derivative and checked graphically by the tangent method. The ∆*T*_m_ values were calculated by subtracting the *T*_m_ of the free nucleic acid from the Tm of the complex. Every ∆*T*_m_ value reported herein was the average of at least two measurements. The error in ∆*T*_m_ is ±0.5 °C. All spectrophotometric data are processed by using the Origin 7.0 program.

## 4. Conclusions

In this paper, we present the synthesis of a novel series of β-carboline/7-chloroquinoline hybrids **33**–**45**, evaluation of their in vitro antiplasmodial activity against CQ-sensitive 3D7 and multi-drug-resistant Dd2 *P. falciparum* strains, and cytotoxicity against the human cell line HepG2. Their antiplasmodial activity against the erythrocytic stage of *P. falciparum* demonstrated strong effectiveness against both CQ-sensitive and resistant *Plasmodium* strains.

Notably, the most active compound against *Pf*3D7, AT harmiquine **37**, displayed a 1.8-fold higher activity against *Pf*3D7 than CQ (IC_50_ = 4.6 ± 0.3 nM) and an 11.4-fold higher activity than CQ against *Pf*Dd2 (IC_50_ = 10.5 ± 0.4 nM). On the other hand, the most active compound against *Pf*Dd2, AT harmiquine **33**, displayed a 1.8-fold higher activity against *Pf*3D7 than CQ (IC_50_ = 4.7 ± 1.3 nM) and 18.4-fold higher activity than CQ against *Pf*Dd2 (IC_50_ = 6.5 ± 2.5 nM).

The study on interactions of representative compounds **34** and **39** with biomacromolecules (DNA, RNA, and HSA) revealed that the shorter and more flexible **34** showed at least two orders of magnitude higher affinity toward DNA/RNA than **39** and much better fluorescence and circular dichroism sensing of a fine difference between various DNA or RNA structures.

Very intriguingly, the binding to the HSA results in the strong hypsochromic shift of **34** and **39** emissions for almost Δ*λ* = -90 nm, which is distinguishable from the effect of DNA/RNA binding (no shift of the emission); thus, the presented compounds can be considered very efficient fluorimetric probes for differentiation between DNA/RNA and proteins. Again, the emission band at 360 nm was much more pronounced for flexible **34** in comparison to rigid **39**.

High SIs of all title compounds, ranging from 711.0 to 8081.8, and low Ris ranging from 0.9 to 5.3, together with significant nanomolar potency against *Plasmodium* strains, additionally confirm their remarkable antiplasmodial activity and pave the way for further optimisation towards developing less cytotoxic and more potent antiplasmodial agents.

## Figures and Tables

**Table 1:** Reaction conditions and yields in the synthesis of the β-carboline-based intermediates (**6**–**8**, and **20**).

Compd.	Base Equivalent	Alkyl Bromide Equivalent	Reaction Time/h	Temp/°C	Yield/%
**6**	1.4	1.2	2.75 h	r.t. and 40	67
**7**	1.4	4.2	3.5 h	r.t. and 40	73
**8**	4.2	8	3 h	r.t. and 60	47
**20**	1.4	1.2	2 h	r.t.	53

r.t., room temperature.

**Table 2:** Drug-like parameters estimated according to Lipinski’s and Veber’s rules.

Compd.	Formula	MW	HA	RB	HBA	HBD	TPSA	cLogP	LV	VV
**33**	C_27_H_2_6ClN_5_O_2_	487.98	35	9	4	2	81.07	4.1	0	0
**34**	C_28_H_28_ClN_5_O_2_	502.01	36	10	4	2	81.07	4.59	1	0
**35**	C_28_H_28_ClN_5_O_2_	502.01	36	9	4	2	81.07	4.43	1	0
**36**	C_26_H_24_ClN_5_O_2_	473.95	34	8	4	2	81.07	3.88	0	0
**37**	C_28_H_28_ClN_5_O_2_	502.01	36	10	4	2	81.07	4.56	1	0
**38**	C_28_H_26_ClN_7_O	512.01	37	8	5	1	82.68	4.26	1	0
**39**	C_29_H_28_ClN_7_O	526.03	38	9	5	1	82.68	4.66	1	0
**40**	C_29_H_28_ClN_7_O	526.03	38	8	5	1	82.68	4.61	1	0
**41**	C_27_H_24_ClN_7_O	497.98	36	7	5	1	82.68	4.09	0	0
**42**	C_29_H_28_ClN_7_O	526.03	38	9	5	1	82.68	4.81	1	0
**43**	C_27_H_24_ClN_7_	481.98	35	7	4	1	73.45	4.5	0	0
**44**	C_28_H_26_ClN_7_	496.01	36	8	4	1	73.45	4.64	0	0
**45**	C_28_H_26_ClN_7_	496.01	36	7	4	1	73.45	4.64	0	0

MW, molecular weight; HA, number of heavy atoms; RB, number of rotatable bonds; HBA, number of H-bond acceptors; HBD, number of H-bond donors; TPSA, polar surface area; clog P, partition coefficient; LV, number of violations of the Lipiski rules; VV, number of the violations of the Veber rules.

**Table 3:** In vitro antiplasmodial activity of harmiquins **33**–**45** against *P. falciparum* erythrocytic stage (*Pf*3D7 and *Pf*Dd2 strains) and resistance index (RI) calculated as the ratio between multi-drug resistant (*Pf*Dd2) and CQ-sensitive (*Pf*3D7) *P. falciparum* strains.

Compd.	IC_50_ ^a^/nM	IC_50_/nM	RI ^e^
	*Pf*3D7	*Pf*Dd2	
**33**	4.7 ± 1.3 ^b^	6.5 ± 2.5	1.4
**34**	11.0 ± 1.5	17.7 ± 8.7	1.6
**35**	28.1 ± 0.5	26.5 ± 4.5	0.9
**36**	16.6 ± 2.1	36.7 ± 4.0	2.2
**37**	4.6 ± 0.6	10.5 ± 0.4	2.3
**38**	18.7 ± 4.7	82.2 ± 2.2	4.4
**39**	6.8 ± 4.0	20.1 ± 2.2	3
**40**	25.0 ± 3.3	33.3 ± 1.6	1.3
**41**	18.5 ± 6.1	98.7 ± 36.3	5.3
**42**	7.6 ± 0.2	15.5 ± 1.7	2
**43**	88.4 ± 0.5	203.5 ± 73.2	2.3
**44**	96.9 ± 1.5	213.8 ± 71.8	2.2
**45**	15.9 ± 11.5	18.8 ± 9.1	1.2
CQ ^c^	8.3 ± 1.4	119.4 ± 39.2	14.4
harmine	8250 ± 2830 ^d^	>27777.8	

^a^ IC_50_, the concentration of the tested compound that is necessary for 50% growth inhibition; ^b^ the results are expressed as mean ± SD (n ≥ 2); ^c^ CQ, chloroquine; ^d^ [28]; ^e^ RI, resistance index expressed as ratio between IC_50_ obtained for multi-drug resistant (Dd2) and CQ-sensitive (3D7) *P. falciparum* strain; IC_50_(*Pf*Dd2)/IC_50_(*Pf*3D7).

**Table 4:** In vitro cytotoxicity screening of the second-generation harmiquins **33**–**35** against HepG2 and calculated selectivity indices.

Compd.	IC_50_ ^a^/μM	SI ^d^
**33**	17.1 ^b^ ± 7.9	3638.3
**34**	18.2 ± 1.8	1654.5
**35**	126.7 ± 31.5	4508.9
**36**	24.3 ± 3.4	1463.9
**37**	35.8 ± 4.3	7782.6
**38**	29.6 ± 6.0	1582.9
**39**	14.4 ± 1.7	2117.6
**40**	26.9 ± 0.9	1076.0
**41**	32.1 ± 1.1	1735.1
**42**	20.3 ± 1.6	2671.1
**43**	68.2 ± 6.1	771.5
**44**	68.9 ± 4.3	711.0
**45**	128.5 ± 2.7	8081.8
harmine	>250 ^c^	>30.3 ^e^

^a^ IC_50_, the concentration of the tested compound that is necessary for 50% growth inhibition; ^b^ the results are expressed as mean ± SD (n ≥ 2); ^c^ the precise IC_50_ value could not be determined as the activity was only detectable at the highest concentration tested [28]; ^d^ SI (selectivity index) = IC_50_ (HepG2)/IC_50_ (Pf3D7); ^e^ the precise selectivity Index (SI) could not be obtained due to the inability to determine the exact IC_50_ value.

**Table 5:** Spectrophotometric data of **34** and **39** measured in sodium cacodylate buffer, pH 7, *I* = 0.05 M.

Compound	^a max^*λ*_abs_/[nm] (*ε* × 10^3^/[M^−1^ cm^−1^])	^b max^*λ*_em_ [nm]	^c^ Δ*λ* [nm]
**34**	325 (13.9)	428	103
**39**	329 (21.8)	422	93

^a max^*λ*_abs_ = absorption maximum; ^b max^*λ*_em_ = emission maximum; ^c^ Δ*λ* = ^max^*λ*_Em_ − ^max^*λ*_Abs._

**Table 6:** Binding constants (^a^log *K_s_*) and emission quenching efficiency (^b^Δ*Int*) of complexes of **34** and **39** with ds-polynucleotides and HSA calculated by processing fluorimetric titrations at pH = 7.0, sodium cacodylate buffer, *I* = 0.05 M.

Compound	^a^ log *K_s_* (^b^ Δ*Int*)				
	ctDNA	pApU	p(dA-dT)_2_	p(dG-dC)_2_	HSA
**34**	7.3	>4 ^c^	5.2	5.9	6.1
	(0.57)	-	(2.28)	(0.08)	(62.8)
**39**	5.5	- ^d^	- ^d^	4.3	5.2 ^e^/5.1 ^f^
	(0.59)	-	-	(0.29)	(43.7 ^e^, 0.37 ^f^)

^a^ The fluorimetric titration data were processed by employing the Scatchard equation. All log *K*_s_ values were re-calculated for fixed *n* [bounded compound]/[polynucleotide] = 0.2 for easier comparison, the correlation coefficients were >0.999 for all calculated *K*_s_; ^b^ Δ*Int* = *Int*_(100% complex)*/*_*Int*_0_; ^c^ Almost linear emission change in the experimental range used allowed only estimation of the binding constant; ^d^ no significant change in the emission of compound was observed; ^e^ binding constant calculated from the results obtained at *λ*_em_ = 360 nm; ^f^ binding constant calculated from the results obtained at *λ*_em_ = 422 nm.

**Table 7:** Δ*T_m_* values ^a^ (°C) for ds-DNA or ds-RNA upon addition of compounds **34** and **39** at ratio ^b^ *r*_[compound]/[polynucleotide]_ = 0.3. Measured in sodium cacodylate buffer, pH 7, *I* = 0.05 M.

Compound	ctDNA	pApU
**34**	−1.1	+0.2
**39**	−0.3	+0.9

^a^ Error in Δ*T*_m_: ±0.5 °C; ^b^ *r =* [compound]/[polynucleotide].

## Data Availability

The original contributions presented in this study are included in the article/Supplementary Materials. Further inquiries can be directed to the corresponding author(s).

## Supplementary Materials

The following supporting information can be downloaded at: https://www.mdpi.com/article/10.3390/molecules29245991/s1, Table S1. Analytical and MS data for β-carbolines **6**–**8**, **20**; Table S2. IR, ^1^H and ^13^C NMR spectroscopic data for β-carbolines **6**–**8**, **20**; Table S3. Analytical and MS data for β-carboline-based Boc-protected amines **9**–**11**, **21**, **23**; Table S4. ^1^H and ^13^C NMR spectroscopic data for β-carboline-based Boc-protected amines **9**–**11**, **21**, **23**; Table S5. Analytical and MS data for β-carboline-based amines **12**–**14**, **22**, **24**; Table S6. ^1^H and ^13^C NMR spectroscopic data for β-carboline-based amines **12**–**14**, **22**, **24**; Table S7. Analytical and MS data for β-carboline-based alkynes **15**–**17**, **25**, **26**, **30**–**32**; Table S8. ^1^H and ^13^C NMR spectroscopic data for β-carboline-based alkynes **15**–**17**, **25**, **26**, **30**–**32**; Table S9. Analytical and MS data for AT harmiquins **33**–**37**; Table S10. ^1^H and ^13^C NMR spectroscopic data for AT harmiquins **33**–**37**; Table S11. Analytical and MS data for TT harmiquins **38**–**45**; Table S12. ^1^H and ^13^C NMR spectroscopic data for TT harmiquins **38**–**45**; Table S13. Structural properties of studied ds-DNA and ds-RNA.; Figure S1. Dependence of the absorbance on the concentration of compound **34** (concentration range 5 × 10^−6^–2.5 × 10^−5^ M). Measured in sodium cacodylate buffer, pH 7, *I* = 0.05 M; Figure S2. Dependence of the absorbance on the concentration of compound **39** (concentration range 5 × 10^−6^–2.5 × 10^−5^ M). Measured in sodium cacodylate buffer, pH 7, *I* = 0.05 M.; Figure S3. Dependence of the emission on the concentration of compound **34** (concentration range 5 × 10^−7^–2 × 10^−6^ M). Measured in sodium cacodylate buffer, pH 7, *I* = 0.05 M, *λ*_exc_ = 325 nm.; Figure S4. Dependence of the emission on the concentration of compound **39** (concentration range 5 × 10^−7^–2 × 10^−6^ M). Measured in sodium cacodylate buffer, pH 7, *I* = 0.05 M, *λ*_exc_ = 329 nm.; Figure S5. (a) Changes in the fluorescence spectrum of **34** (*c* = 5 × 10^−6^ M, *λ*_exc_ = 325 nm) upon titration with **ctDNA** (*c* = 5 × 10^−6^–4.1 × 10^−5^ M), slit: 5–10; (b) Dependence of **34** emission at *λ*_max_ = 428 nm on *c*(ctDNA), at pH 7.0, sodium cacodylate buffer, *I* = 0.05 M. Figure S6. (a) Changes in the fluorescence spectrum of **39** (*c* = 5 × 10^−6^ M, *λ*_exc_ = 329 nm) upon titration with **ctDNA** (*c* = 5 × 10^−6^–1.5 × 10^−4^ M), slit: 5–5; (b) Dependence of **39** emission at *λ*_max_ = 422 nm on *c*(ctDNA), at pH 7.0, sodium cacodylate buffer, *I* = 0.05 M.; Figure S7. (a) Changes in the fluorescence spectrum of **34** (*c* = 5 × 10^−6^ M, *λ*_exc_ = 325 nm) upon titration with **poly A—poly U** (*c* = 5 × 10^−6^–2 × 10^−4^ M), slit: 5–5; (b) Dependence of **34** emission at *λ*_max_ = 428 nm on *c*(pApU), at pH 7.0, sodium cacodylate buffer, *I* = 0.05 M.; Figure S8. (a) Changes in the fluorescence spectrum of **34** (*c* = 5 × 10^−6^ M, *λ*_exc_ = 325 nm) upon titration with **poly A—poly U** (*c* = 5 × 10^−6^–2 × 10^−4^ M), slit: 5–5; (b) Dependence of **34** emission at *λ*_max_ = 428 nm on *c*(pApU), at pH 7.0, sodium cacodylate buffer, *I* = 0.05 M.; Figure S9. (a) Changes in the fluorescence spectrum of **34** (*c* = 5 × 10^−6^ M, *λ*_exc_ = 325 nm) upon titration with **poly (dAdT)—poly (dAdT)** (*c* = 5 × 10^−6^–1.8 × 10^−4^ M), slit: 5–5; (b) Dependence of **34** emission at *λ*_max_ = 428 nm on *c*(p(dA-dT)_2_), at pH 7.0, sodium cacodylate buffer, *I* = 0.05 M.; Figure S10. (a) Changes in the fluorescence spectrum of **39** (*c* = 5 × 10^−6^ M, *λ*_exc_ = 329 nm) upon titration with **poly (dAdT)—poly (dAdT)** (*c* = 5 × 10^−6^–9.2 × 10^−5^ M), slit: 5–5; (b) Dependence of **39** emission at *λ*_max_ = 422 nm on *c*(p(dA-dT)_2_), at pH 7.0, sodium cacodylate buffer, *I* = 0.05 M.; Figure S11. (a) Changes in the fluorescence spectrum of **34** (*c* = 5 × 10^−6^ M, *λ*_exc_ = 325 nm) upon titration with **poly (dGdC)—poly (dGdC)** (*c* = 5 × 10^−6^–2.5 × 10^−4^ M), slit: 5–10; (b) Dependence of **34** emission at *λ*_max_ = 428 nm on *c*(p(dG-dC)_2_), at pH 7.0, sodium cacodylate buffer, *I* = 0.05 M.; Figure S12. (a) Changes in the fluorescence spectrum of **39** (*c* = 5 × 10^−6^ M, *λ*_exc_ = 329 nm) upon titration with **poly (dGdC)—poly (dGdC)** (*c* = 5 × 10^−6^–2.4 × 10^−4^ M), slit: 5–5; (b) Dependence of **39** emission at *λ*_max_ = 422 nm on *c*(p(dG-dC)_2_), at pH 7.0, sodium cacodylate buffer, *I* = 0.05 M. Figure S13. (a) Changes in the fluorescence spectrum of **34** (*c* = 5 × 10−^6^ M, *λ*_exc_ = 325 nm) upon titration with **HSA** (*c* = 5 × 10^−6^–1.5 × 10^−4^ M), slit: 5–5; (b) Dependence of **34** emission at max = 360 nm on c(HSA); (c) Changes in the fluorescence spectrum of 39 (*c* = 5 × 10^−6^ M, *λ*_exc_ = 329 nm) upon titration with **HSA** (*c* = 5 × 10^−6^–1.8 × 10^−4^ M), slit: 5–5; (d) Dependence of **39** emission at max = 360 and 422 nm on c(HSA), at pH 7.0, sodium cacodylate buffer, *I* = 0.05 M. Figure S14. CD titration of **ctDNA** (*c* = 2 × 10^−5^ M), **poly A—poly U** (c = 2 × 10^−5^ M) with **34** at molar ratio *r* = [compound]/[polynucleotide] (pH 7.0, buffer sodium cacodylate, *I* = 0.05 M). Figure S15. CD titration of **ctDNA** (*c* = 3 × 10^−5^ M) and **poly A—poly U** (*c* = 2 × 10^−5^ M), with **39** at molar ratio *r* = [compound]/[polynucleotide] (pH 7.0, buffer sodium cacodylate, *I* = 0.05 M). Figure S16. (**a**) Denaturation of **ctDNA** upon addition of *r* = 0.3 ([compound/[polynucleotide]) of **34** at pH 7.0 (buffer sodium cacodylate, *I* = 0.05 M), red lines denote fitting of experimental data to sigmoidal eq. by Origin 7.5; (b) The first derivation of absorbance (fitted to sigmoidal eq.) on temperature dependence. Figure S17. (a) Denaturation of **pApU** upon addition of *r* = 0.3 ([compound/[polynucleotide]) of **34** at pH 7.0 (buffer sodium cacodylate, *I* = 0.05 M), red lines denote fitting of experimental data to sigmoidal eq. by Origin 7.5; (b) The first derivation of absorbance (fitted to sigmoidal eq.) on temperature dependence. Figure S18. (a) Denaturation of **ctDNA** upon addition of *r* = 0.3 ([compound/[polynucleotide]) of **39** at pH 7.0 (buffer sodium cacodylate, *I* = 0.05 M), red lines denote fitting of experimental data to sigmoidal eq. by Origin 7.5; (b) The first derivation of absorbance (fitted to sigmoidal eq.) on temperature dependence. Figure S19. (**a**) Denaturation of **pApU** upon addition of *r* = 0.3 ([compound/[polynucleotide]) of **39** at pH 7.0 (buffer sodium cacodylate, *I* = 0.05 M), (b) The first derivation of absorbance on temperature dependence. Refs. [56,57] are cited in Supplementary Materials.
