# Supplementary material for: Towards Novel Antiplasmodial Agents—Design, Synthesis and Antimalarial Activity of Second-Generation β-Carboline/Chloroquine Hybrids
Source: Molecules. 2024 Dec 19;29(24):5991. doi: 10.3390/molecules29245991 (PMC11678116; doi:10.3390/molecules29245991)
Supplement: Supplementary file 1 [file molecules-29-05991-s001.zip › molecules-3325084-supplementary.pdf]

Supplementary materials

Towards novel antiplasmodial agents - design, synthesis and antimalarial activity of second generation β-carboline/chloroquine hybrids

Ana Penava [1], Marina Marinović [1], Lais Pesanha de Carvallo [2], Jana Held [2,3], Ivo Piantanida [4], Dijana Pavlović Saftić [4], Zrinka Rajić [1*], Ivana Perković [1*]

[1] University of Zagreb Faculty of Pharmacy and Biochemistry, A. Kovačića 1, 10000 Zagreb, Croatia; ana.penava@pharma.unizg.hr (A.P.), marina.marinovic@pharma.unizg.hr (M.M.)
[2] University of Tübingen, Institute of Tropical Medicine, Wilhelmstraße 27, 72074 Tübingen, Germany lais.pessanha-de-carvalho@uni-tuebingen.de (L.P.C.)
[3] German Center for Infection Research (DZIF), Partner Site Tübingen, 72074 Tübingen, Germany; jana.held@uni-tuebingen.de (J.H.)
[4] Rudjer Bošković Institute, Bijenička cesta 54, 10000 Zagreb, Croatia; ivo.piantanida@irb.hr (I.Pi.), dijana.pavlovic.saftic@irb.hr (D.P.S.)
* Correspondence: ivana.perkovic@pharma.unizg.hr (I.Pe.), zrinka.rajic@pharma.unizg.hr (Z.R.)

1. Analytical, MS, IR and NMR data for the compounds 6-17, 20-26 and 30-45.

Table S1. Analytical and MS data for β-carbolines **6-8**, **20**

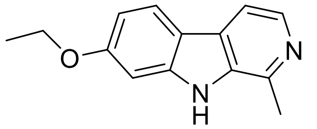

6

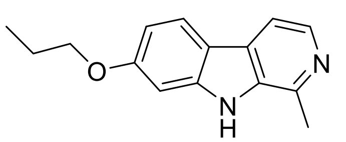

7

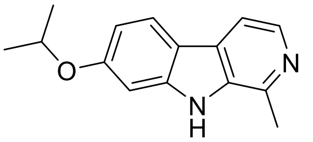

8

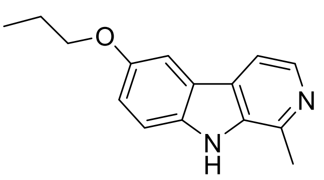

20

Compd.	Yield (%)	t_t (°C)	Molecular formula	M_r	MS (m/z)
**6**	67	193-194.5	$C_{14}H_{14}N_2O$	226.28	227.0 $(M + 1)^+$
**7**	73	203-205.5	$C_{15}H_{16}N_2O$	240.31	241.1 $(M + 1)^+$
**8**	47	178-180	$C_{15}H_{16}N_2O$	240.31	241.1 $(M + 1)^+$
**20**	53	190.5-191	$C_{15}H_{16}N_2O$	240.31	241.2 $(M + 1)^+$

Table S2. IR, ^{1}H and ^{13}C NMR spectroscopic data for β-carbolines **6-8**, **20**

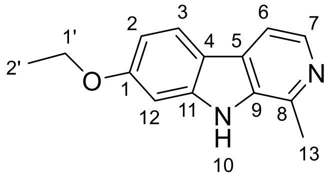

6

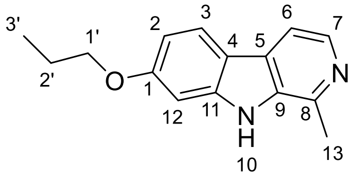

7

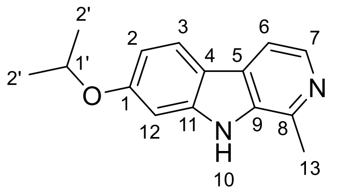

8

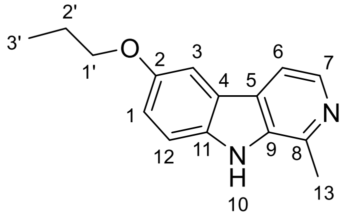

20

Compd.	IR (ATR, ν/cm^{-1})	^{1}H NMR (DMSO-d_6, δ ppm)	^{13}C NMR (DMSO-d_6, δ ppm)
**6**	2977, 2867, 1628, 1568, 1444, 1328, 1277, 1171, 1107, 1041, 966, 871, 832, 813, 769, 686, 653	11.43 (s, 1H, 10), 8.15 (d, 1H, 7, *J* = 5.2 Hz), 8.04 (d, 1H, 3, *J* = 8.6 Hz), 7.81 (d, 1H, 6, *J* = 5.3 Hz), 7.00 (d, 1H, 12, *J* = 2.1 Hz), 6.83 (dd, 1H, 2, *J* = 8.6, 2.2 Hz), 4.13 (q, 2H, 1', *J* = 7.0 Hz), 2.51 (s, 2H, 13), 1.40 (t, 3H, 2', *J* = 6.9 Hz)	158.78 (1), 141.44 (8), 140.52 (9), 136.88 (7), 133.90 (11), 126.76 (5), 122.04 (3), 114.13 (4), 111.35 (6), 108.85 (2), 94.54 (12), 62.68 (1'), 19.61 (13), 14.10 (2')
**7**	2963, 2871, 2765, 1626, 1569, 1486, 1447, 1328, 1297, 1281, 1235, 1176, 1136, 1105, 1071, 985, 878, 837, 819, 801, 686, 655, 633	11.43 (s, 1H, 10), 8.15 (d, 1H, 7, *J* = 5.3 Hz), 8.04 (d, 1H, 3, *J* = 8.6 Hz), 7.80 (d, 1H, 6, *J* = 5.2 Hz), 7.00 (d, 1H, 12, *J* = 1.9 Hz), 6.84 (dd, 1H, 2, *J* = 8.6, 2.1 Hz), 4.03 (t, 2H, 1', *J* = 6.5 Hz), 2.73 (s, 3H, 13), 1.80 (h, 2H, 2', *J* = 7.1 Hz), 1.03 (t, 3H, 3', *J* = 7.4 Hz)	158.91 (1), 141.41 (8), 140.56 (9), 136.96 (7), 133.92 (11), 126.71 (5), 122.01 (3), 114.15 (4), 111.33 (6), 108.84 (2), 94.61 (12), 68.57 (1'), 21.50 (2'), 19.67 (13), 9.89 (3')
**8**	3051, 2923, 2854, 1626, 1568, 1449, 1380, 1275, 1183, 1167, 978, 878, 805, 640	11.44 (s, 1H, 10), 8.15 (d, 1H, 7, *J* = 4.8 Hz), 8.04 (d, 1H, 3, *J* = 8.5 Hz), 7.82 (d, 1H, 6, *J* = 4.7 Hz), 7.00 (s, 1H, 12), 6.82 (d, 1H, 2, *J* = 8.1 Hz),	157.66 (1), 141.56 (8), 140.38 (9), 136.61 (7), 133.91 (11), 126.87 (5), 122.16 (3), 114.10 (4), 111.40 (6), 109.77 (2), 96.00 (12), 68.98 (1'), 21.24 (2'), 19.46 (13)

		4.76 – 4.70 (m, 2H, 1'), 2.73 (s, 2H, 13), 1.34 (d, 6H, *J* = 5.7 Hz, 2')	
**20**	2954, 1580, 1486, 1448, 1324, 1287, 1212, 1047, 1022, 909, 877, 844, 815, 766, 622	11.35 (s, 1H, 10), 8.15 (d, 1H, 7, *J* = 5.3 Hz), 7.90 (d, 1H, 6, *J* = 5.3 Hz), 7.73 (d, 1H, 3, *J* = 2.4 Hz), 7.49 (d, 1H, 12, *J* = 8.8 Hz), 7.17 (dd, 1H, 1, *J* = 8.8, 2.5 Hz), 4.02 (t, 2H, 1', *J* = 6.5 Hz), 2.74 (s, 3H, 13), 1.79 (h, 2H, 2', *J* = 7.2 Hz), 1.03 (t, 3H, 3', *J* = 7.4 Hz)	153.11 (2), 142.60 (8), 137.32 (7), 135.68 (11), 135.54 (9), 127.22 (4), 121.90 (5), 118.71 (1), 113.17 (12), 113.16 (6), 104.94 (3), 70.11 (1'), 22.73 (2'), 20.85 (13), 11.02 (3')

Table S3. Analytical and MS data for β-carboline based Boc-protected amines **9-11**, **21**, **23**

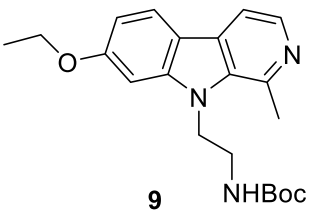

9

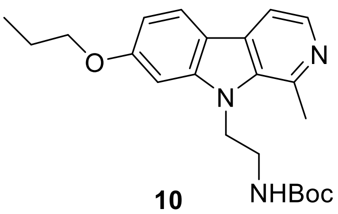

10

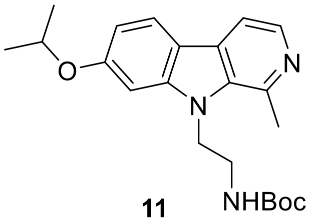

11

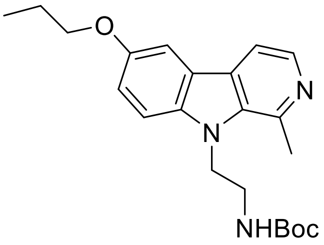

21

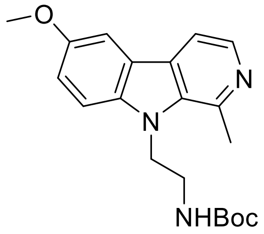

23

**Compd.**	**Yield (%)**	t_t **(°C)**	**Molecular formula**	M_r	**MS (*m*/*z*)**
**9**	55	167-167.5	$C_{21}H_{27}N_3O_3$	369.47	370.2325 $(M + 1)^+$
**10**	48	178-179.5	$C_{22}H_{29}N_3O_3$	383.49	384.3 $(M + 1)^+$
**11**	40	173.5-174.5	$C_{15}H_{16}N_2O$	383.49	284.3 $(M + 1)^+$
**21**	50	175-181.5	$C_{22}H_{29}N_3O_3$	383.49	384.20 $(M + 1)^+$
**23**	50	161-162	$C_{20}H_{25}N_3O_3$	355.44	356.25 $(M + 1)^+$

Table S4. ^{1}H and ^{13}C NMR spectroscopic data for β-carboline based Boc-protected amines **9-11**, **21**, **23**

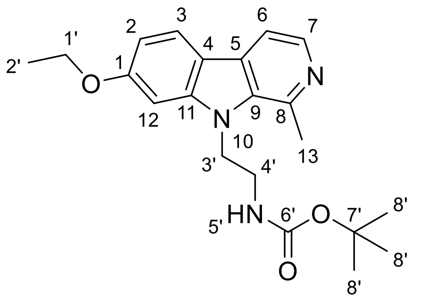

9

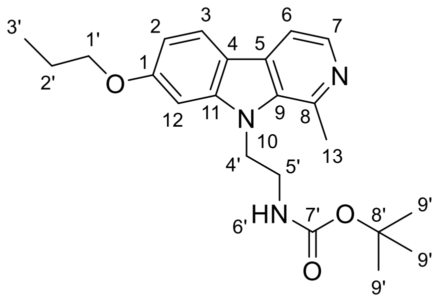

10

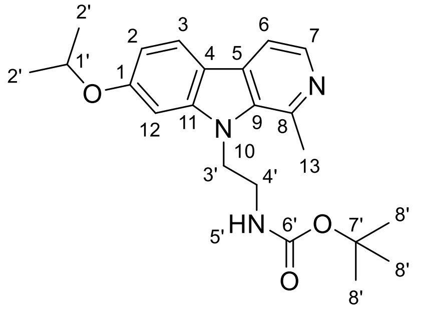

11

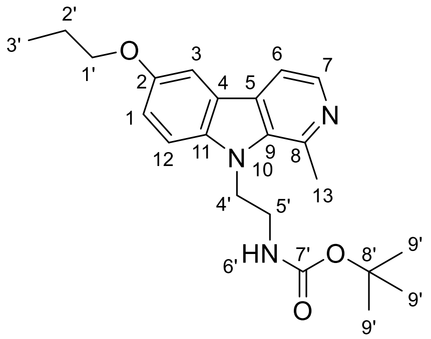

21

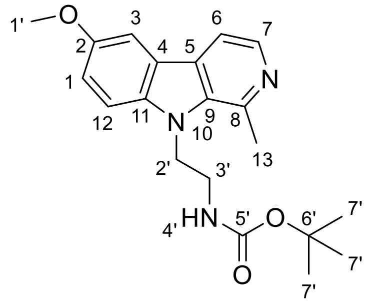

23

Compd.	IR (ATR, ν/cm^{-1})	^{1}H NMR (DMSO-d_6, δ ppm)	^{13}C NMR (DMSO-d_6, δ ppm)
**9**	3239, 2974, 1698, 1623, 1564, 1503, 1447, 1365, 1308, 1276, 1247, 1165, 1148, 1124, 1048, 977, 962, 874, 802, 733, 637	8.15 (d, 1H, 7, *J* = 5.1 Hz), 8.06 (d, 1H, 3, *J* = 8.5 Hz), 7.85 (d, 1H, 6, *J* = 5.0 Hz), 7.19 (s, 1H, 12), 7.04 (t, 1H, 5', *J* = 5.7 Hz), 6.86 (d, 1H, 2, *J* = 8.5 Hz), 4.54 (t, 2H, 3', *J* = 6.4 Hz), 4.18 (q, 2H, 1', *J* = 6.8 Hz), 3.33 (s, 3H, 4'), 2.94 (s, 3H, 13), 1.41 (t, 3H, 2', *J* = 6.9 Hz), 1.29 (s, 8H, 8', 1.00 (s, 1H, 8')	160.17 (6'), 156.14 (1), 143.38 (8), 140.97 (9), 138.17 (7), 135.21 (11), 128.92 (4), 122.77 (3), 114.78 (5), 112.67 (6), 109.66 (2), 94.88 (12), 78.30 (7'), 63.93 (1'), 44.48 (3'), 40.58 (4'), 28.54 (8'), 23.58 (13), 15.17 (2')
**10**	3190, 2970, 1698, 1622, 1567, 1447, 1391, 1364, 1276, 1245, 1164, 1147, 1124, 1042, 989, 968, 876, 802, 769, 682, 638	8.15 (d, 1H, 7, *J* = 5.1 Hz), 8.06 (d, 1H, 3, *J* = 8.5 Hz), 7.85 (d, 1H, 6, *J* = 5.1 Hz), 7.22 (d, 1H, 12, *J* = 2.2 Hz), 7.05 (t, 1H, 6', *J* = 6.0 Hz), 6.86 (dd, 1H, 2, *J* = 8.6, 2.1 Hz), 4.54 (t, 2H, 1', *J* = 6.7 Hz), 4.09 (t, 2H, 4', *J* = 6.6 Hz), 3.32 (d, 2H, 5', *J* = 7.1 Hz), 2.94 (s, 3H, 13), 1.82 (d, 2H, 2' *J* = 7.1 Hz), 1.30 (s, 8H, 9'), 1.04 (d, 3H, 3', *J* = 7.4 Hz)	160.34 (7'), 156.16 (1), 143.38 (10), 140.96 (9), 138.17 (7), 135.19 (11), 128.91 (4), 122.74 (3), 114.74 (5), 112.66 (6), 109.77 (2), 94.81 (12), 78.31 (8'), 69.86 (1'), 44.48 (4'), 40.59 (5'), 28.54 (9'), 23.57 (13), 22.61 (2'), 10.96 (3')
**11**	3240, 2978, 2934, 1702, 1620, 1561, 1497, 1364, 1333, 1244, 1225, 1168, 1037, 967, 876, 833, 816, 779, 646	8.15 (d, 1H, 7, *J* = 5.1 Hz), 8.05 (d, 1H, 3, *J* = 8.5 Hz), 7.85 (d, 1H, 6, *J* = 5.2 Hz), 7.20 (s, 1H, 12), 7.04 (t, 1H, 5', *J* = 5.7 Hz), 6.84 (dd, 1H, 2, *J* = 8.5, 1.6 Hz), 4.84 – 4.79 (m, 1H, 1'), 4.53 (t, 2H, 3', *J* = 6.5 Hz), 3.33 (s, 2H, 4'), 2.94 (s, 3H, 13), 1.36 (d, 6H, 2', *J* = 6.0 Hz), 1.29 (s, 8H, 8'), 0.99 (s, 1H, 8')	157.95 (6'), 155.08 (1), 142.39 (8), 139.85 (9), 137.08 (7), 134.15 (11), 127.83 (4), 121.72 (3), 113.63 (5), 111.57 (6), 109.37 (2), 94.99 (12), 77.23 (7'), 68.85 (1'), 43.35 (3'), 39.56 (4'), 27.46 (8'), 22.49 (2'), 21.34 (13)
**21**	3208, 2964, 1702, 1566, 1487, 1367, 1275, 1223, 1050, 840, 814, 743, 623	8.16 (d, 1H, 7, *J* = 5.2 Hz), 7.96 (d, 1H, 6, *J* = 5.2 Hz), 7.77 (d, 1H, 3, *J* = 2.5 Hz), 7.57 (d, 1H, 12, *J* = 9.0 Hz), 7.23 (dd, 1H, 1, *J* = 8.9, 2.4 Hz), 7.03 (t, 1H, 6', *J* = 5.9 Hz), 4.57 (t, 2H, 4', *J* = 6.7 Hz), 4.03 (t, 2H, 1', *J* = 6.5 Hz),	155.07 (7'), 152.34 (2'), 140.72 (8), 136.28 (7), 135.59 (9), 134.40 (11), 127.23 (4), 120.42 (5), 117.61 (6), 112.41 (1), 110.44 (12), 103.81 (3), 77.25 (2), 69.12 (1'), 43.32 (4'), 39.64

		3.28 (q, 2H, 5', *J* = 6.6 Hz), 2.96 (s, 3H, 13), 1.79 (h, 2H, 2', *J* = 7.2 Hz), 1.31 (s, 6H, 9'), 1.03 (t, 3H, 3'/9', *J* = 7.4 Hz)	(5'), 27.50 (9'), 22.58 (13), 21.60 (2'), 9.91 (3')
**23**	3205, 2990, 1702, 1570, 1489, 1475, 1389, 1355, 1284, 1248, 1227, 1202, 1187, 1122, 1048, 1024, 863, 815, 805, 757, 705, 670	8.17 (d, 1H, 7, *J* = 5.2 Hz), 7.97 (d, 1H, 6, *J* = 5.2 Hz), 7.78 (d, 1H, 3, *J* = 2.5 Hz), 7.59 (d, 1H, 12, *J* = 9.0 Hz), 7.23 (dd, 1H, 1, *J* = 8.9, 2.5 Hz), 7.03 (t, 1H, 4', *J* = 5.9 Hz), 4.58 (t, 2H, 2', *J* = 6.7 Hz), 3.86 (s, 3H, 1'), 3.29 (q, 2H, 3', *J* = 6.5 Hz), 2.96 (s, 3H, 13), 1.31 (s, 8H, 7'), 1.04 (s, 1H, 7')	156.16 (5'), 154.10 (2), 141.84 (8), 137.34 (7), 136.72 (9), 135.48 (11), 128.32 (4), 121.46 (5), 118.32 (6), 113.47 (1), 111.59 (12), 103.90 (3), 78.33 (6'), 56.16 (1'), 44.42 (2'), 40.73 (3'), 28.58 (7'), 23.65 (13)

Table S5. Analytical and MS data for β-carboline based amines **12-14**, **22**, **24**

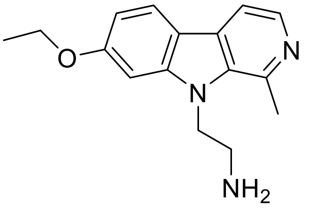

12

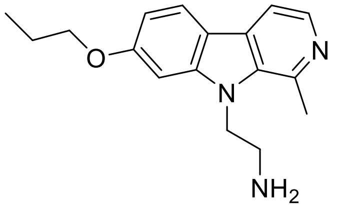

13

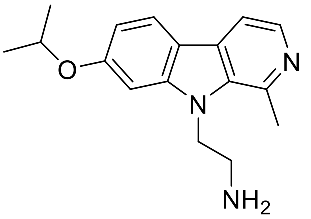

14

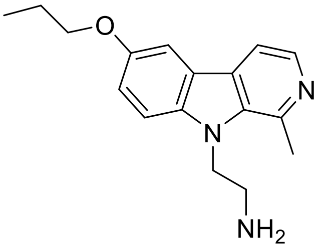

22

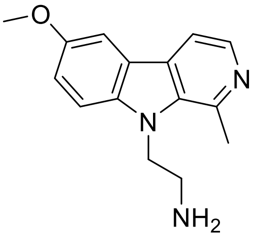

24

**Compd.**	**Yield (%)**	t_t **(°C)**	**Molecular formula**	M_r	**MS (*m*/*z*)**
**12**	72	156-157.5	$C_{16}H_{19}N_3O$	269.35	270.10 $(M + 1)^+$
**13**	80	167.5-173	$C_{17}H_{21}N_3O$	283.38	284.3 $(M + 1)^+$
**14**	34	105-107.5	$C_{17}H_{21}N_3O$	283.38	284.3 $(M + 1)^+$
**22**	71	106.5-108.5	$C_{17}H_{21}N_3O$	283.38	284.20 $(M + 1)^+$
**24**	62	111-113	$C_{15}H_{17}N_3O$	255.32	256.20 $(M + 1)^+$.

Table S6. ^{1}H and ^{13}C NMR spectroscopic data for β-carboline based amines **12-14**, **22**, **24**

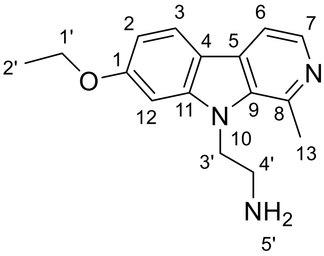

12

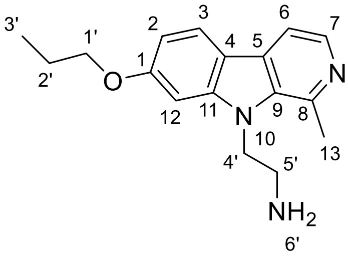

13

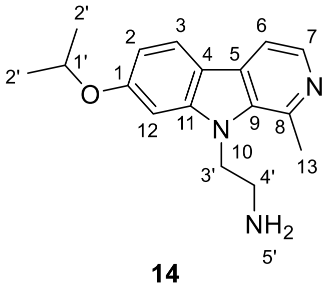

14

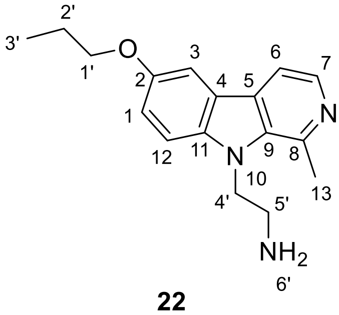

22

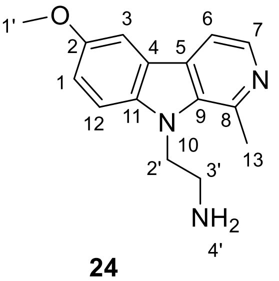

24

**Compd.**	**IR (ATR, *ν*/cm⁻¹)**	**^{1}H NMR (DMSO-*d*₆, *δ* ppm)**	**^{13}C NMR (DMSO-*d*₆, *δ* ppm)**
**12**		8.15 (d, 1H, 7, *J* = 5.1 Hz), 8.06 (d, 1H, 6, *J* = 8.5 Hz), 7.85 (d, 1H, 3, *J* = 5.1 Hz), 7.22 (s, 1H, 12), 6.85 (dd, 1H, 2, *J* = 8.5 Hz, 1.5 Hz), 4.51 (t, 2H, 3', *J* = 7.2 Hz), 4.18 (q, 2H, 1', *J* = 6.9 Hz), 2.96 (s, 3H, 13), 2.90 (t, 2H, 5', *J* = 7.2 Hz), 1.65 (s, 2H, 6'), 1.40 (t, 3H, 3', *J* = 6.9 Hz)	160.18 (1), 143.41 (8), 141.08 (9), 138.08 (7), 135.26 (11), 128.69 (4), 122.75 (1), 114.58 (5), 112.63 (6), 109.76 (2), 94.90 (12), 63.98 (1'), 47.66 (3'), 42.61 (4'), 23.76 (13), 15.16 (2')
**13**		8.16 (d, 1H, 7, *J* = 5.2 Hz), 8.07 (d, 1H, 3, *J* = 8.5 Hz), 7.86 (d, 1H, 6, *J* =	160.49 (1), 143.29 (8), 141.06 (9), 138.28 (7), 135.12 (11), 128.87 (4),

		5.1 Hz), 7.30 (d, 1H, 12, *J* = 2.2 Hz), 6.87 (dd, 1H, 2, *J* = 8.6, 2.1 Hz), 4.64 (t, 2H, 1', *J* = 7.5 Hz), 4.10 (t, 2H, 4', *J* = 6.5 Hz), 3.00 (t, 2H, 5', *J* = 7.6 Hz), 2.97 (s, 3H, 13), 2.51 (s, 2H, 6'), 1.81 (h, 2H, 2', *J* = 7.1 Hz), 1.04 (t, 3H, 3', *J* = 7.4 Hz)	122.83 (3), 114.65 (5), 112.67 (6), 109.98 (2), 94.88 (12), 69.98 (1'), 45.39 (4), 41.08 (5), 23.72 (13), 22.62 (2'), 11.01 (3')
**14**	1619, 1412, 1218, 1155, 1109, 980, 844, 812	8.14 (d, 1H, 7, *J* = 5.1 Hz), 8.05 (d, 1H, 3, *J* = 8.6 Hz), 7.85 (d, 1H, 6, *J* = 5.1 Hz), 7.22 (d, 1H, 12, *J* = 2.0 Hz), 6.84 (dd, 1H, 2, *J* = 8.6, 2.1 Hz), 4.86 – 4.80 (m, 1H, 1'), 4.51 (t, 2H, 3', *J* = 7.2 Hz), 2.99 (s, 1H, 13), 2.96 (s, 2H, 13), 2.90 (t, 2H, 5', *J* = 7.3 Hz), 1.34 (d, 6H, 2', *J* = 6.0 Hz)	158.99 (1), 143.46 (8), 141.05 (9), 138.07 (7), 135.29 (11), 128.69 (4), 122.81 (3), 114.59 (5), 112.63 (6), 110.60 (2), 96.35 (12), 69.96 (1'), 47.57 (3'), 42.61 (4'), 29.13 (13), 23.76 (2')
**22**	2964, 1655, 1582, 1449, 1374, 1287, 1218, 1052, 991, 850, 813, 616	8.16 (d, 1H, 7, *J* = 5.1 Hz), 7.96 (d, 1H, 6, *J* = 5.0 Hz), 7.77 (s, 1H, 3), 7.66 (d, 1H, 12, *J* = 8.9 Hz), 7.22 (dd, 1H, 1, *J* = 8.8, 1.6 Hz), 4.53 (t, 2H, 4', *J* = 7.0 Hz), 4.03 (t, 2H, 1', *J* = 6.4 Hz), 2.97 (s, 3H, 13), 2.89 (t, 2H, 5', *J* = 7.0 Hz), 1.79 (h, 2H, 2', *J* = 6.9 Hz), 1.03 (t, 3H, 3', *J* = 7.4 Hz)	153.35 (2), 141.91 (8), 137.25 (7), 136.73 (9), 135.57 (11), 128.06 (4), 121.32 (5), 118.67 (6), 113.48 (1), 111.92 (12), 104.75 (3), 70.17 (1'), 47.87 (4'), 42.82 (5'), 23.88 (13), 22.70 (2'), 10.99 (3')
**24**	1561, 1489, 1446, 1290, 1221, 1049, 814, 616	8.16 (d, 1H, 7, *J* = 5.2 Hz), 7.96 (d, 1H, 6, *J* = 5.2 Hz), 7.77 (d, 1H, 3, *J* = 2.5 Hz), 7.67 (d, 1H, 12, *J* = 9.0 Hz), 7.22 (dd, 1H, 1, *J* = 8.9, 2.5 Hz), 4.54 (t, 2H, 2', *J* = 7.2 Hz), 3.87 (s, 3H, 1'), 2.98 (s, 3H, 13), 2.90 (t, 2H, 3', *J* = 7.2 Hz)	154.00 (2), 141.95 (8), 137.26 (7), 136.74 (9), 135.55 (11), 128.04 (4), 121.28 (5), 118.28 (6), 113.45 (1), 111.96 (12), 103.81 (3), 56.14 (1'), 47.76 (2'), 42.75 (3'), 23.87 (13)

Table S7. Analytical and MS data for β-carboline based alkynes **15-17**, **25**, **26**, **30-32**

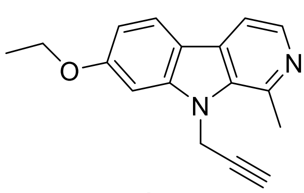

15

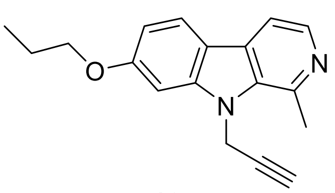

16

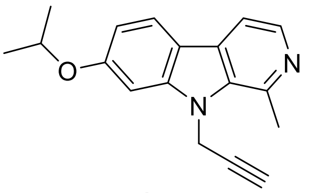

17

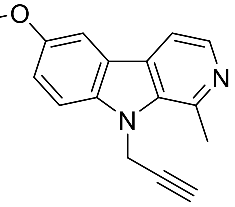

25

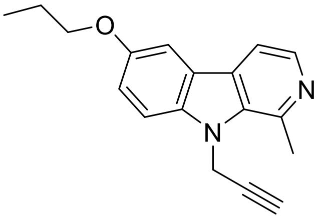

26

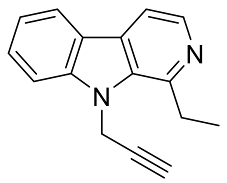

30

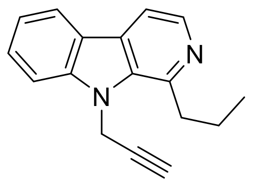

31

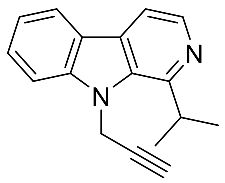

32

Compd.	Yield (%)	t_t (°C)	Molecular formula	M_r	MS (*m/z*)
**15**	55	137.5-140	$C_{17}H_{16}N_2O$	264.33	265.10 $(M + 1)^+$
**16**	47	135-136	$C_{18}H_{18}N_2O$	278.36	279.05 $(M + 1)^+$
**17**	47	134-137	$C_{18}H_{18}N_2O$	278.36	279.10 $(M + 1)^+$
**25**	10	137.5-145.5	$C_{16}H_{14}N_2O$	250.30	251.15 $(M + 1)^+$
**26**	52	137.5-140.5	$C_{18}H_{18}N_2O$	278.36	267.20 $(M + 1)^+$
**30**	40	126-128	$C_{16}H_{14}N_2$	234.30	235.15 $(M + 1)^+$
**31**	55	89.5-90.5	$C_{17}H_{16}N_2$	248.33	249.20 $(M + 1)^+$
**32**	29	89.5-91.5	$C_{17}H_{16}N_2$	248.33	249.20 $(M + 1)^+$

Table S8. ^{1}H and ^{13}C NMR spectroscopic data for β-carboline based alkynes **15-17**, **25**, **26**, **30-32**

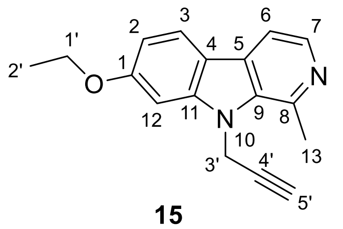

15

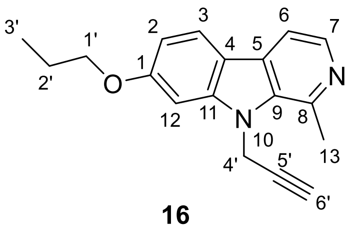

16

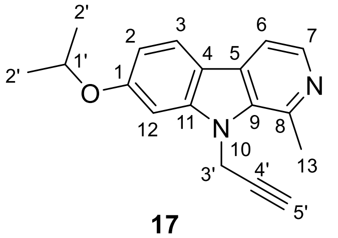

17

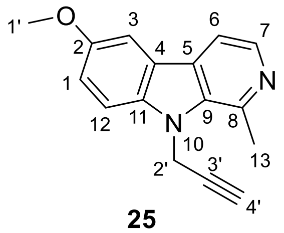

25

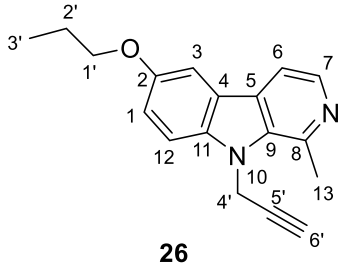

26

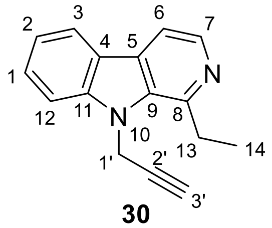

30

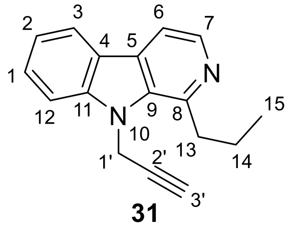

31

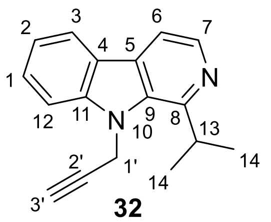

32

Compd.	IR (ATR, ν/cm^{-1})	^{1}H NMR (DMSO-d_6, δ ppm)	^{13}C NMR (DMSO-d_6, δ ppm)
**15**	3270, 2980, 1626, 1564, 1445, 1394, 1305, 1254, 1208, 1189, 1110, 1046, 950, 927, 842, 810, 798, 697, 671, 632	8.19 (d, 1H, 7, *J* = 5.2 Hz), 8.08 (d, 1H, 3, *J* = 8.6 Hz), 7.87 (d, 1H, 6, *J* = 5.3 Hz), 7.31 (d, 1H, 12, *J* = 2.0 Hz), 6.89 (dd, 1H, 2, *J* = 8.6, 2.1 Hz), 5.43 (d, 2H, 3', *J* = 2.3 Hz), 4.19 (q, 2H, 1', *J* = 7.0 Hz), 3.04 (s, 3H, 13), 2.96 (s, 1H, 5'), 1.41 (d, 3H, 2', *J* = 6.9 Hz)	159.90 (1), 142.57 (8), 141.02 (9), 138.48 (7), 134.38 (11), 128.91 (5), 122.45 (3), 114.33 (4), 112.23 (6), 109.90 (2), 94.40 (12), 75.49 (4'), 63.57 (1'), 34.16 (3'), 22.46 (13'), 14.64 (2')
**16**	2960, 2106, 1622, 1566, 1443, 1335, 1279, 1251, 1184, 1141, 1119, 1050, 979; 926, 811, 730, 648	8.19 (d, 1H, 7, *J* = 5.2 Hz), 8.08 (d, 1H, 3, *J* = 8.6 Hz), 7.87 (d, 1H, 6, *J* = 5.2 Hz), 7.32 (d, 1H, 12, *J* = 1.9 Hz), 6.90 (dd, 1H, 2, *J* = 8.5, 2.1 Hz), 5.44 (d, 2H, 4', *J* = 2.2 Hz), 4.09 (t, 2H, 1', *J* = 6.6 Hz), 3.36 (t, 1H, 6', *J* = 2.2 Hz), 3.04 (s, 3H, 13), 1.81 (h, 2H, 2', *J* = 7.2 Hz), 1.04 (t, 3H, 3', *J* = 7.4 Hz)	160.58 (1), 143.08 (8), 141.53 (9), 138.99 (7), 134.88 (11), 129.41 (5), 122.95 (3), 114.82 (4), 112.74 (6), 110.44 (2), 94.91 (12), 76.00 (5'), 69.97 (1'), 34.67 (4'), 22.97 (2'), 22.60 (13), 10.97 (3')
**17**	3116, 2974, 2106, 1632, 1571, 1496, 1444, 1373, 1348, 1299, 1282, 1218, 1183, 1134, 1109, 979, 827, 803, 671, 648	8.19 (d, 1H, 7, *J* = 5.2 Hz), 8.07 (d, 1H, 3, *J* = 8.6 Hz), 7.87 (d, 1H, 6, *J* = 5.2 Hz), 7.31 (d, 1H, 12, *J* = 1.7 Hz), 6.87 (dd, 1H, 2, *J* = 8.6, 1.9 Hz), 5.43 (d, 2H, 4', *J* = 2.0 Hz), 4.84 (hept, 1H, 1', *J* = 6.0 Hz), 3.35 (t, 1H, 5', *J* = 2.0 Hz), 3.04 (s, 3H, 13), 1.35 (d, 6H, 2', *J* = 6.0 Hz)	159.26 (1), 143.13 (8), 141.50 (9), 138.99 (7), 134.92 (11), 129.42 (5), 123.01 (3), 114.83 (4), 112.74 (6), 111.21 (2), 96.22 (12), 75.95 (4'), 70.07 (1'), 34.66 (3'), 23.00 (13), 22.36 (2')
**25**	3162, 3014, 2111, 1585, 1564, 1489, 1452, 1375, 1333, 1289, 1229, 1123, 1043, 980, 928, 832, 816, 720, 619	8.21 (d, 1H, 7, *J* = 5.2 Hz), 7.99 (d, 1H, 6, *J* = 5.2 Hz), 7.80 (d, 1H, 3, *J* = 2.5 Hz), 7.72 (d, 1H, 12, *J* = 9.0 Hz), 7.26 (dd, 1H, 1, *J* = 8.9, 2.5 Hz), 5.43 (d, 2H, 2', *J* = 2.3 Hz), 3.87 (s, 3H, 1'), 3.04 (s, 3H, 13)	154.48 (2), 142.36 (8), 138.29 (7), 136.33 (9), 135.28 (11), 128.92 (4), 121.83 (5), 118.45 (6), 113.58 (1), 111.89 (12), 104.15 (3), 76.00 (3'), 56.17 (1'), 34.80 (2'), 23.07 (13)

**26**	2940, 2109, 1582, 1450, 1374, 1288, 1211, 1197, 990, 909, 878, 830, 816, 745, 622	8.21 (d, 1H, 7, *J* = 5.2 Hz), 7.99 (d, 1H, 6, *J* = 5.3 Hz), 7.80 (d, 1H, 3, *J* = 2.4 Hz), 7.70 (d, 1H, 12, *J* = 8.9 Hz), 7.25 (dd, 1H, 1, *J* = 8.9, 2.5 Hz), 5.42 (d, 3H, 4', *J* = 2.2 Hz), 4.04 (t, 3H, 1', *J* = 6.5 Hz), 3.04 (s, 3H, 13), 2.98 (s, 1H, 6'), 1.79 (h, 3H, 2', *J* = 7.1 Hz), 1.03 (t, 4H, 3', *J* = 7.4 Hz)	152.76 (2), 141.23 (8), 137.21 (7), 135.22 (9), 134.20 (11), 127.86 (4), 120.79 (5), 117.72 (6), 112.52 (1), 110.74 (12), 103.99 (3), 95.85 (6'), 85.40 (5'), 69.10 (1'), 33.71 (4'), 21.99 (2'), 21.61 (13), 9.91 (3')
**30**	3276, 1656, 1581, 1450, 1374, 1197, 991, 806, 623	8.34 (t, 1H, 7, *J* = 4.9 Hz), 8.26 (t, 1H, 3, *J* = 7.0 Hz), 8.02 (d, 1H, 6, *J* = 5.1 Hz), 7.79 (dd, 1H, 12, *J* = 17.9, 8.4 Hz), 7.66 – 7.58 (m, 1H, 1), 7.32 (dt, 1H, 2, *J* = 10.3, 7.5 Hz), 5.76 (d, 1H, 1', *J* = 6.2 Hz), 5.44 (d, 1H, 1', *J* = 2.4 Hz), 3.45 – 3.38 (m, 2H, 13), 3.34 (s, 1H, 3'), 1.39 (dt, 3H, *J* = 32.3, 7.5 Hz, 14)	146.87 (8), 141.47 (9), 138.94 (7), 134.04 (11), 129.43 (4), 128.84 (3), 121.98 (12), 121.52 (2), 120.67 (6), 113.34 (12), 110.99 (5), 96.44 (2'), 86.05 (3'), 35.05 (1'), 28.14 (13), 13.75 (14)
**31**	2968, 1612, 1579, 1452, 1430, 1330, 1227, 1200, 1140, 1047, 875, 815, 746	8.33 (d, 1H, 7, *J* = 5.1 Hz), 8.25 (d, 1H, 3, *J* = 7.7 Hz), 8.02 (d, 1H, 6, *J* = 5.0 Hz,), 7.79 (d, 1H, 12, *J* = 8.3 Hz), 7.63 (t, 1H, 1, *J* = 7.6 Hz), 7.31 (t, 1H, 2, *J* = 7.4 Hz), 5.41 (d, 2H, 1', *J* = 1.62 Hz), 3.36 (t, 3H, 13'/2', *J* = 7.8 Hz), 1.90 (h, 2H, 14, *J* = 7.4 Hz), 1.05 (t, 3H, 15, *J* = 7.3 Hz)	145.90 (8), 141.51 (9), 138.93 (7), 134.18 (11), 129.59 (4), 128.87 (1), 121.98 (3), 121.52 (5), 120.69 (2), 113.33 (6), 110.99 (12), 76.10 (2'), 37.07 (1'), 35.04 (13), 22.62 (14), 14.49 (15)
**32**		8.38 (d, 1H, 7, *J* = 5.0 Hz), 8.25 (d, 1H, 3, *J* = 7.7 Hz), 8.02 (d, 1H, 6, *J* = 5.1 Hz), 7.80 (d, 1H, 12, *J* = 8.4 Hz), 7.63 (ddd, 1H, 1, *J* = 8.4, 7.1, 1.3 Hz), 7.31 (ddd, 1H, 2, *J* = 7.9, 7.0, 0.9 Hz), 5.43 (d, 2H, 1', *J* = 2.4 Hz), 3.97 (hept, 1H, 13, *J* = 6.6 Hz), 3.37 (t, 1H, 3', *J* = 2.4 Hz), 1.42 (d, 6H, 14, *J* = 6.6 Hz)	150.40 (8), 141.13 (9), 138.47 (7), 132.46 (11), 129.37 (4), 128.31 (3), 121.32 (1), 120.96 (5), 120.10 (2), 112.57 (6), 110.40 (12), 75.54 (2'), 34.86 (1'), 30.73 (13), 22.70 (14)

Table S9. Analytical and MS data for AT harmiquins **33-37**

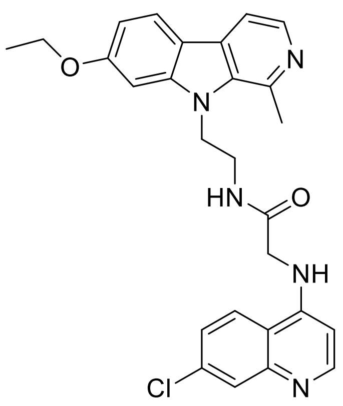

33

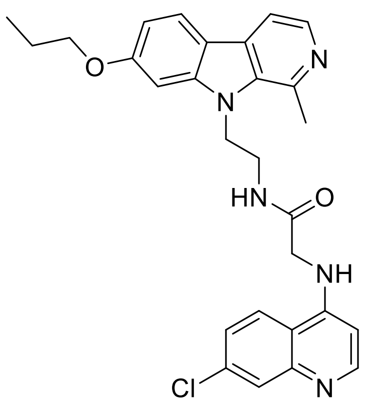

34

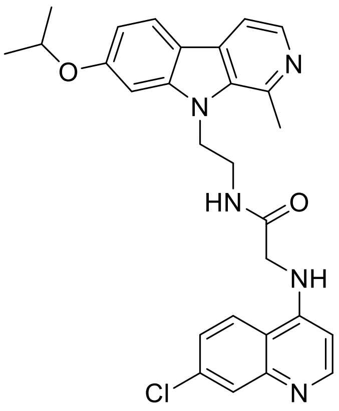

35

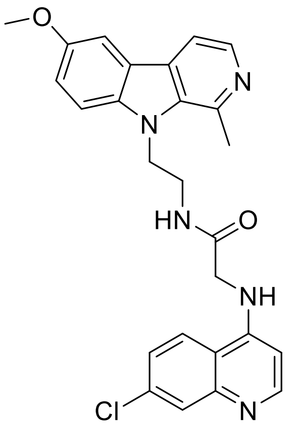

36

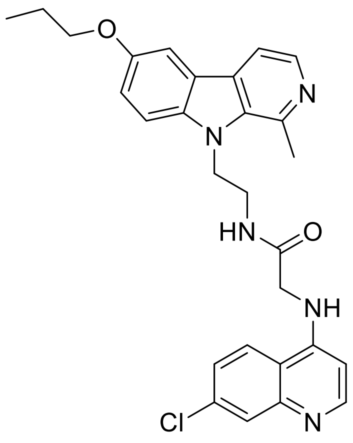

37

Compd.	Yield (%)	t_t (°C)	Molecular formula	M_r	MS (*m*/*z*)
**33**	46	204-205	$C_{27}H_{26}ClN_5O_2$	487.99	488.20 $(M + 1)^+$
**34**	20	232.5-234.5	$C_{28}H_{28}ClN_5O_2$	502.02	502.2 $(M + 1)^+$
**35**	37	249.5-255.5	$C_{28}H_{28}ClN_5O_2$	502.02	502.2 $(M + 1)^+$
**36**	40	219.5-222.5	$C_{26}H_{24}ClN_5O_2$	473.96	474.30 $(M + 1)^+$
**37**	16	238.5-245.5	$C_{28}H_{28}ClN_5O_2$	502.02	502.20 $(M + 1)^+$

Table S10. ^{1}H and ^{13}C NMR spectroscopic data for AT harmiquins **33-37**

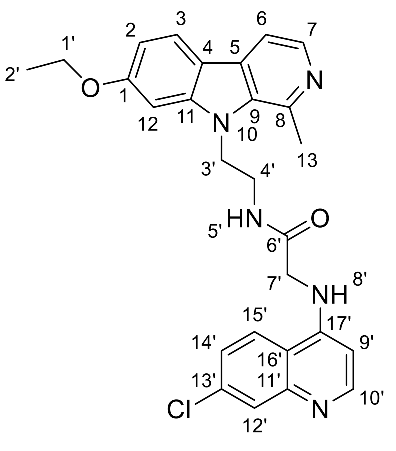

33

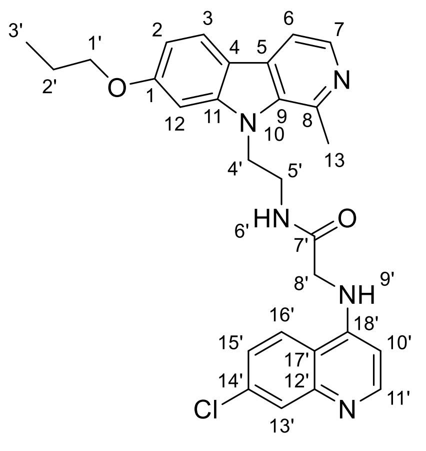

34

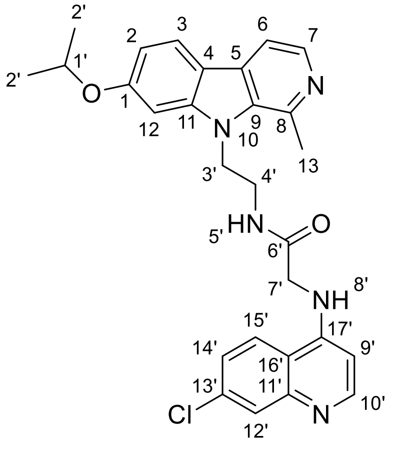

35

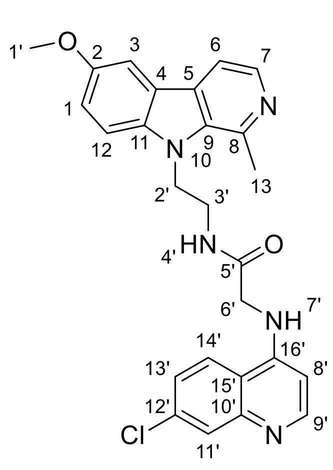

36

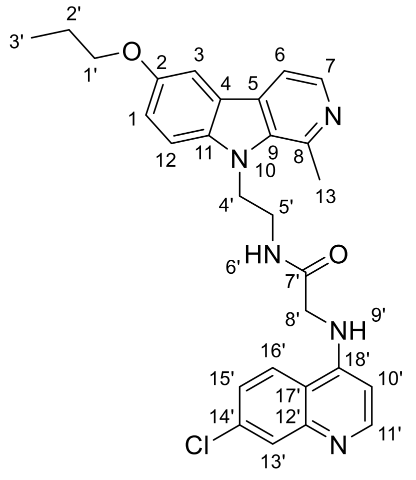

37

Compd.	IR (ATR, ν/cm⁻¹)	^{1}H NMR (DMSO-d_6, δ ppm)	^{13}C NMR (DMSO-d_6, δ ppm)
**33**	3269, 2926, 1657, 1623, 1580, 1499, 1444, 1339, 1279, 1248, 1186, 1140, 1112, 1049, 975, 850, 804, 765, 732, 638	8.38 (t, 1H, 5', *J* = 5.5 Hz), 8.32 (d, 1H, 10', *J* = 5.3 Hz), 8.26 (d, 1H, 15', *J* = 9.0 Hz), 8.16 (d, 1H, 7, *J* = 5.1 Hz), 8.09 (d, 1H, 3, *J* = 8.5 Hz), 7.87 (d, 1H, 6, *J* = 5.0 Hz), 7.82 (d, 1H, 12', *J* = 2.3 Hz), 7.80 (d, 1H, 8', *J* = 6.4 Hz), 7.50 (dd, 1H, 14', *J* = 8.9, 2.3 Hz), 7.25 (s, 1H, 12), 6.88 (d, 1H, 2, *J* = 8.4 Hz), 6.10 (d, 1H, 9', *J* = 5.3 Hz), 4.56 (t, 2H, 3', *J* = 6.8 Hz), 4.15 (q, 2H, 1', *J* = 6.8 Hz), 3.87 (d, 1H, 7', *J* = 5.5 Hz), 3.51 (q, 1H, 4', *J* = 6.9 Hz), 2.96 (s, 1H, 13), 1.37 (t, 2H, 2', *J* = 6.9 Hz)	169.96 (6'), 160.30 (17), 152.05 (1), 150.79 (10'), 149.06 (11'), 143.38 (8), 140.99 (11), 138.21 (9), 135.08 (7), 134.09 (12'), 128.96 (13'), 127.70 (5), 124.88 (14'), 124.71 (1'), 122.91 (3), 117.95 (16'), 114.70 (4), 112.72 (6), 109.90 (2), 99.45 (9'), 94.70 (12), 63.95 (1'), 46.41 (7'), 43.74 (3'), 39.25 (4'), 23.50 (13), 15.14 (2')
**34**	2963, 1656, 1623, 1581, 1444, 1340, 1278, 1248, 1188, 1140, 1048, 988, 851, 798, 637	8.36 (t, 1H, 6', *J* = 6.0 Hz), 8.32 (d, 1H, 11', *J* = 5.4 Hz), 8.25 (d, 1H, 16', *J* = 9.0 Hz), 8.16 (d, 1H, 7, *J* = 5.2 Hz), 8.09 (d, 1H, 3, *J* = 8.6 Hz), 7.87 (d, 1H, 6, *J* = 5.2 Hz), 7.82 (d, 1H, 13', *J* = 2.2 Hz), 7.79 (t, 1H, 9', *J* = 6.2 Hz), 7.50 (dd, 1H, 15', *J* = 9.0, 2.3 Hz), 7.25 (d, 1H, 12, *J* = 2.1 Hz), 6.89 (dd, 1H, 2, *J* = 8.6, 2.1 Hz), 6.09 (d, 1H, 10', *J* = 5.4 Hz), 4.57 (t, 2H, 1', *J* = 7.1 Hz), 4.05 (t, 2H, 4', *J* = 6.5 Hz), 3.87 (d, 2H, 8', *J* = 5.8 Hz), 3.51 (q, 2H, 5', *J* = 6.8 Hz), 2.96 (s, 3H, 13), 1.80 – 1.74 (m, 2H, 2'), 0.99 (t, 3H, 3', *J* = 7.4 Hz)	169.94 (7'), 160.46 (18'), 152.01 (11'), 150.81 (1), 149.01 (12'), 143.38 (8), 140.99 (11), 138.20 (7), 135.09 (9), 134.11 (14'), 128.97 (5), 127.67 (13'), 124.90 (15'), 124.68 (16'), 122.91 (3), 117.93 (17'), 114.70 (4), 112.73 (6), 109.93 (2), 99.45 (10'), 94.71 (12), 69.83 (1'), 46.39 (8'), 43.74 (4'), 39.26 (5'), 23.50 (13), 22.57 (2'), 10.95 (3')
**35**	3384, 3198, 2975, 1684, 1624, 1586, 1526, 1449, 1388, 1334, 1281, 1232, 1198, 1114, 1081, 1025, 986, 935, 857, 821, 810, 759, 739, 643	8.38 (t, 1H, 5', *J* = 6.1 Hz), 8.33 (d, 1H, 10', *J* = 5.4 Hz), 8.25 (d, 1H, 15', *J* = 9.0 Hz), 8.16 (d, 1H, 7, *J* = 5.2 Hz), 8.08 (d, 1H, 3, *J* = 8.6 Hz), 7.86 (d, 1H, 6, *J* = 5.2 Hz), 7.82 (d, 1H, 12', *J* = 2.3 Hz), 7.79 (t, 1H, 8', *J* = 6.1 Hz), 7.50 (dd, 1H, 14', *J* = 9.0, 2.3 Hz), 7.26 (d, 1H, 12, *J* = 2.1 Hz), 6.88 (dd, 1H, 2, *J* = 8.6, 2.1 Hz), 6.12 (d, 1H, 9', *J* = 5.4 Hz), 4.83 – 4.77 (m, 1H, 1'), 4.55 (t, 2H, 3', *J* = 7.2 Hz), 3.88 (d, 2H, 7', *J* = 5.9 Hz), 3.49 (q, 2H, 4', *J* = 7.2 Hz), 2.96 (s, 3H, 13), 1.32 (d, 6H, 2', *J* = 6.0 Hz)	169.97 (6'), 159.15 (17'), 152.15 (10'), 150.75 (1), 149.17 (11'), 143.37 (8), 140.99 (11), 138.23 (7), 135.14 (9), 134.05 (13'), 128.92 (5), 127.79 (12'), 124.87 (15'), 124.69 (14'), 122.98 (3), 117.98 (16'), 114.74 (4), 112.72 (6), 110.61 (2), 99.48 (9'), 96.17 (12), 70.08 (1'), 46.39 (7'), 43.73 (3'), 39.15 (4'), 23.52 (13), 22.39 (2')
**36**	3272, 2920, 1666, 1611, 1489, 1408, 1374, 1226, 1200, 1178, 1126, 1048, 1022, 983, 899, 850, 806, 703, 670, 619	8.35 (d, 1H, 4', *J* = 6.0 Hz), 8.33 (d, 1H, 9', *J* = 5.5 Hz), 8.25 (d, 1H, 14', *J* = 9.0 Hz), 8.18 (d, 1H, 7, *J* = 5.2 Hz), 7.98 (d, 1H, 6, *J* = 5.2 Hz), 7.82 (d, 1H, 11', *J* = 2.3 Hz), 7.82-7.79 (m, 1H, 7'), 7.80 (d, 1H, 3, *J* = 2.7 Hz), 7.65 (d, 1H, 12, *J* = 9.0 Hz), 7.50 (dd, 1H, 13', *J* = 9.0, 2.3 Hz), 7.23 (dd, 1H, 1, *J* = 9.0, 2.5 Hz), 6.09 (d, 1H, 8', *J* = 5.5 Hz), 4.60 (t, 2H, 2', *J* = 7.0 Hz), 3.87 (s, 3H, 1'), 3.85 (d, 2H, 6', *J* = 5.9	169.84 (5'), 154.15 (16'), 151.97 (9'), 150.83 (2), 148.98 (10'), 141.87 (8), 137.46 (7), 136.67 (9), 135.37 (12'), 134.12 (11), 128.34 (4), 127.63 (11'), 124.90 (14'), 124.71 (13'), 121.45 (15'), 118.40 (6), 117.93 (5), 113.53 (1), 111.61 (12), 103.97 (3), 99.46 (8'), 56.15 (1'), 46.29 (6'), 43.92 (2'), 40.41 (3'), 23.64 (13)

		Hz), 3.48 (q, 2H, 3', *J* = 6.6 Hz), 2.97 (s, 3H, 13)	
**37**	3273, 2964, 1656, 1611, 1582, 1488, 1450, 1374, 1225, 1196, 991, 850, 802, 766, 618	8.33 (d, 2H, 6'/11', *J* = 4.7 Hz), 8.24 (d, 1H, 16', *J* = 9.0 Hz), 8.17 (d, 1H, 7, *J* = 5.2 Hz), 7.97 (d, 1H, 6, *J* = 5.1 Hz), 7.81 (d, 1H, 13', *J* = 1.9 Hz), 7.79 (d, 1H, 3, *J* = 2.2 Hz), 7.73 (t, 1H, 9', *J* = 5.7 Hz), 7.63 (d, 1H, 12, *J* = 9.0 Hz), 7.49 (dd, 1H, 15', *J* = 9.0, 2.0 Hz), 7.23 (dd, 1H, 1, *J* = 8.9, 2.3 Hz), 6.08 (d, 1H, 10', *J* = 5.4 Hz), 4.59 (t, 2H, 4', *J* = 6.9 Hz), 4.03 (t, 2H, 1', *J* = 6.5 Hz), 3.84 (d, 2H, 8', *J* = 5.8 Hz), 3.48 (q, 3H, 5', *J* = 6.5 Hz), 2.97 (s, 3H, 13), 1.79 (h, 2H, 2', *J* = 7.1 Hz), 1.03 (t, 3H, 3', *J* = 7.4 Hz)	169.91 (7'), 153.50 (18'), 152.25 (11'), 150.65 (2), 149.30 (12'), 141.84 (8), 137.50, (7) 136.65 (9), 135.38 (14'), 134.00 (11), 128.35 (4), 127.89 (13'), 124.83 (16'), 124.66 (15'), 121.50 (17'), 118.75 (6), 117.99 (5), 113.55 (1), 111.55 (12), 104.89 (3), 99.47 (18'), 70.17 (1'), 46.31 (8'), 43.91 (4'), 40.42 (5'), 23.66 (13), 22.71 (2'), 11.01 (3')

Table S11. Analytical and MS data for TT harmiquins **38-45**

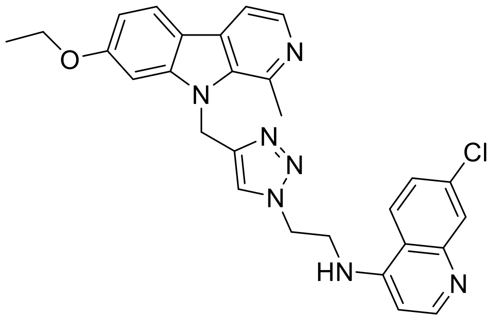

38

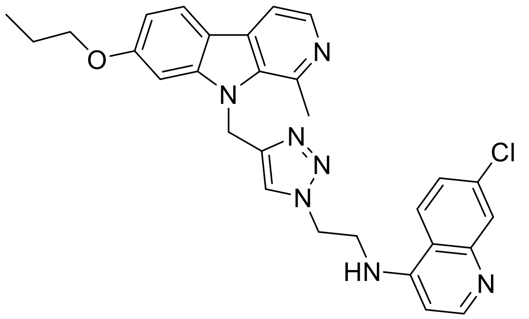

39

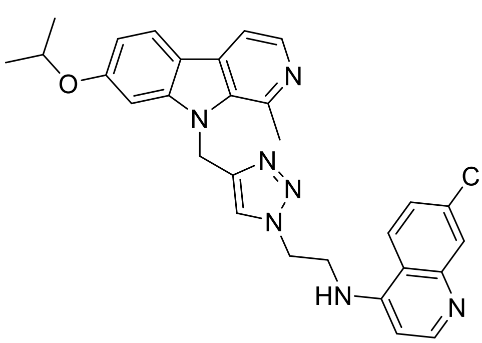

40

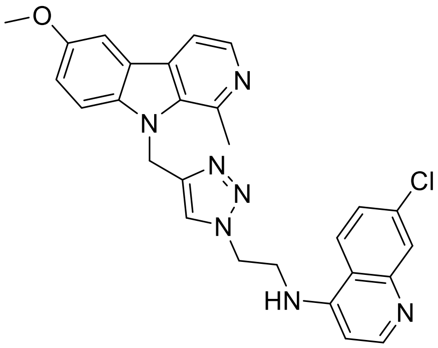

41

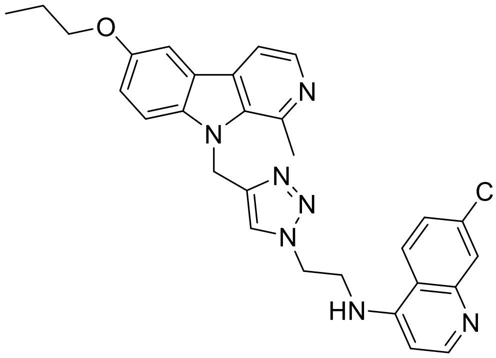

42

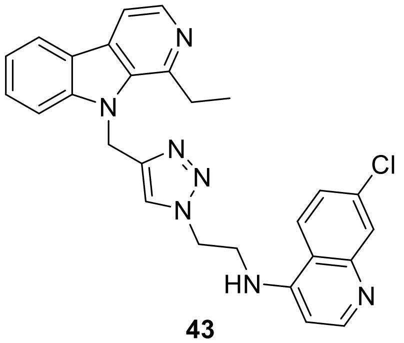

43

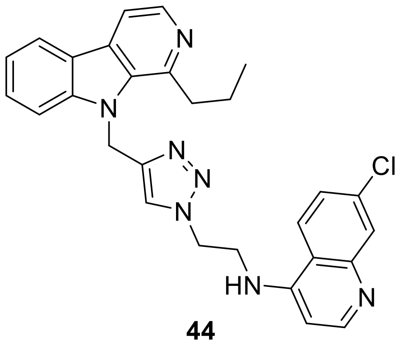

44

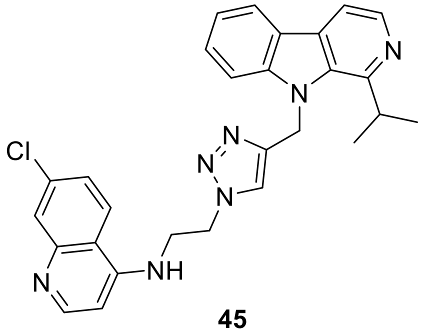

45

Compd.	Yield (%)	t_t (°C)	Molecular formula	M_r	MS (*m*/*z*)
**38**	58	227.5-230	$C_{28}H_{26}ClN_7O$	512.01	512.20 (M + 1)
**39**	72	245.5-249	$C_{29}H_{28}ClN_7O$	526.04	526.15 $(M + 1)^+$
**40**	47	234-236	$C_{29}H_{28}ClN_7O$	526.04	526.25 $(M + 1)^+$
**41**	46	245.5-250.5	$C_{27}H_{24}ClN_7O$	497.99	498.20 $(M + 1)^+$
**42**	40	205-206	$C_{29}H_{28}ClN_7O$	526.04	526.20 $(M + 1)^+$
**43**	44	192.5-194.5	$C_{27}H_{24}ClN_7$	481.99	482.20 $(M + 1)^+$
**44**	45	205-209 °C	$C_{28}H_{26}ClN_7$	496.02	496.20 $(M + 1)^+$
**45**	41	189-192	$C_{28}H_{26}ClN_7$	496.02	496.25 $(M + 1)^+$

Table S12. ^{1}H and ^{13}C NMR spectroscopic data for TT harmiquins **38-45**

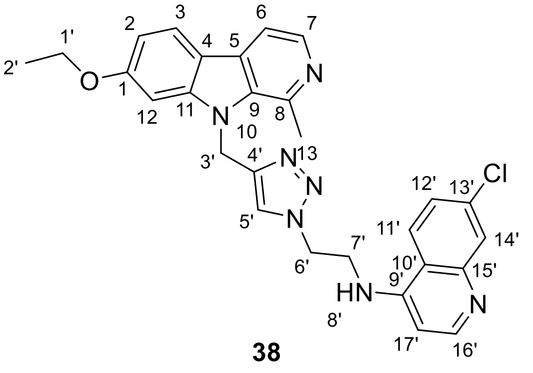

38

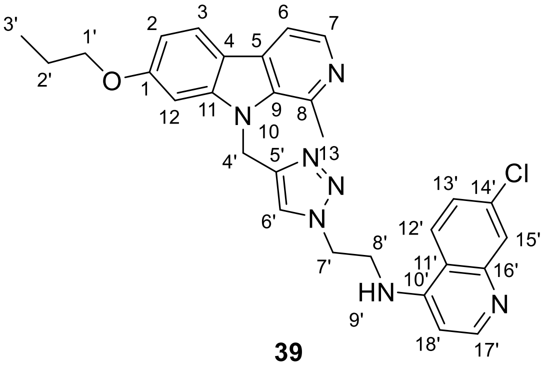

39

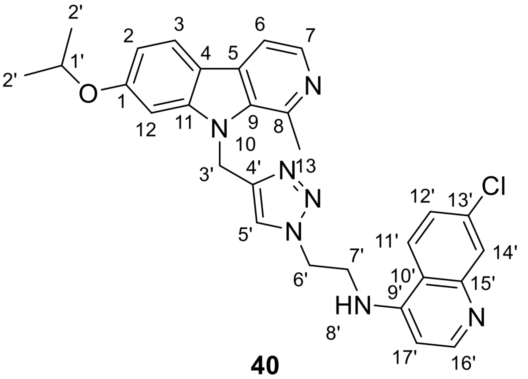

40

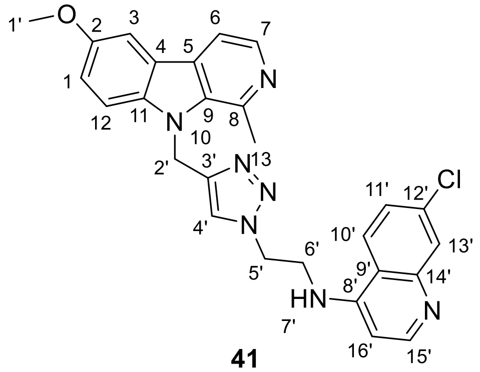

41

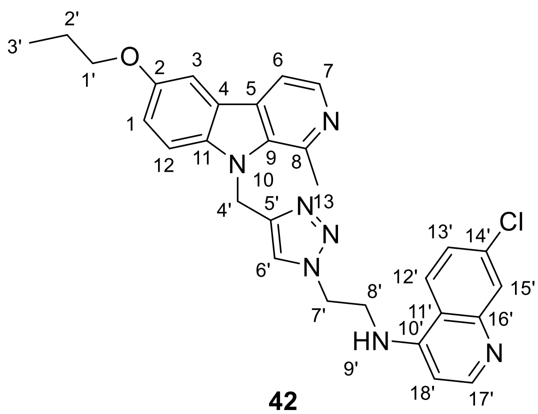

42

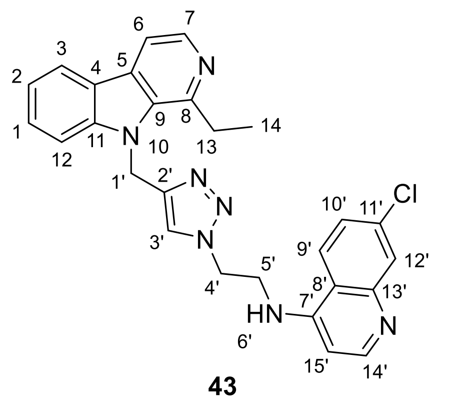

43

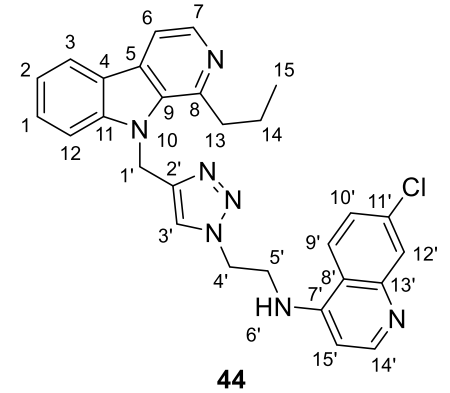

44

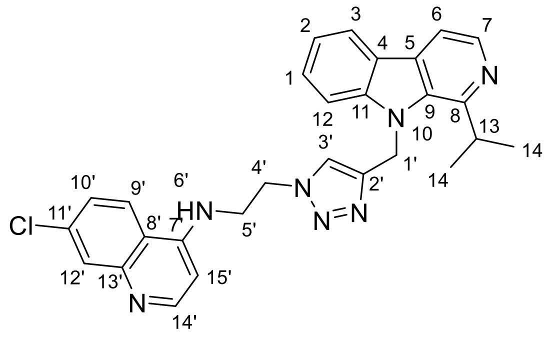

45

Compd.	IR (ATR, *ν*/cm⁻¹)	^{1}H NMR (DMSO-*d*$_6$, *δ* ppm)	^{13}C NMR (DMSO-*d*$_6$, *δ* ppm)
**38**	2972, 1622, 1579, 1444, 1408, 1324, 1250, 1181, 1140, 1047, 954, 910, 876, 814, 765, 641	8.31 (d, 1H, 16', *J* = 5.4 Hz), 8.16 (d, 1H, 7, *J* = 5.2 Hz), 8.06 (d, 1H, 11', *J* = 8.6 Hz), 8.03 (d, 1H, 3, *J* = 9.1 Hz), 7.97 (s, 1H, 6'), 7.86 (d, 1H, 6, *J* = 5.2 Hz), 7.79 (d, 1H, 14', *J* = 2.2 Hz), 7.39 (dd, 1H, 2, *J* = 9.0, 2.2 Hz), 7.37 (d, 1H, 8', *J* = 6.0 Hz), 7.24 (d, 1H, 12, *J* = 2.1 Hz), 6.85 (dd, 1H, 13', *J* = 8.6, 2.1 Hz), 6.45 (d, 1H, 17', *J* = 5.4 Hz), 5.82 (s, 2H, 3'), 4.57 (t, 2H, 6', *J* = 6.0 Hz), 4.08 (q, 2H, 1', *J* = 7.0 Hz), 3.69 (q, 2H, 7', *J* = 5.8 Hz), 2.98 (s, 3H, 13), 1.35 (t, 3H, 2', *J* = 6.9 Hz)	159.74 (9'), 151.59 (16'), 149.70 (15'), 148.70 (1), 143.85 (8), 142.62 (9), 141.00 (11), 137.92 (7), 134.56 (13'), 133.52 (4'), 128.48 (10'), 127.30 (5'), 124.31 (11'), 123.72 (5'), 123.41 (3), 122.29 (12'), 117.29 (4), 114.27 (5), 112.17 (6), 109.60 (2), 98.74 (12), 94.43 (17'), 63.42 (1'), 47.84 (3'), 42.32 (6'/7'), 23.07 (13), 14.58 (2')
**39**	3204, 2964, 1622, 1578, 1498, 1445, 1429, 1366, 1341, 1323, 1181, 1140, 1081, 1048, 982, 935, 910, 877, 815, 766, 732, 640	^{1}H NMR (600 MHz, DMSO-*d*$_6$) δ 8.31 (d, 1H, 17', *J* = 5.4 Hz), 8.17 (d, 1H, 7, *J* = 5.2 Hz), 8.07 (d, 1H, 12', *J* = 8.6 Hz), 8.03 (d, 1H, 3, *J* = 9.1 Hz), 7.96 (s, 1H, 6'), 7.86 (d, 1H, 6, *J* = 5.2 Hz), 7.78 (d, 1H, 15', *J* = 2.1 Hz), 7.39 (dd, 1H, 2, *J* = 9.0, 2.2 Hz), 7.36 (d, 1H, 9', *J* = 5.6 Hz), 7.25 (d, 1H, 12, *J* = 1.8 Hz), 6.87 (dd, 1H, 13', *J* = 8.6, 2.0 Hz), 6.45 (d, 1H, 18', *J* = 5.5 Hz), 5.83 (s, 2H, 4'), 4.57 (t, 2H, 7', *J* = 6.0 Hz), 3.99 (t, 2H, 1', *J* = 6.6 Hz), 3.69 (q, 2H, 8', *J* = 5.8 Hz), 2.97 (s, 3H, 13), 1.76 (h, 2H, 2', *J* = 7.1 Hz), 0.99 (t, 3H, 3', *J* = 7.4 Hz)	159.33 (10'), 151.06 (17'), 149.10 (1'), 148.17 (16'), 143.36 (8), 142.07 (9), 140.42 (11), 137.36 (7), 134.00 (14'), 132.93 (5'), 127.92 (11'), 126.77 (15'), 123.72 (12'), 123.14 (6'), 122.78 (3), 121.71 (13'), 116.73 (4), 113.70 (5), 111.60 (6), 109.05 (2), 98.17 (12), 93.89 (18'), 68.73 (1'), 47.27 (4'), 41.75 (7'/8'), 22.49 (13), 21.47 (2'), 9.87 (3')

**40**	2974, 1622, 1578, 1445, 1408, 1324, 1182, 1139, 1111, 1047, 909, 875, 803	8.31 (d, 1H, 16', *J* = 5.4 Hz), 8.16 (d, 1H, 7, *J* = 5.2 Hz), 8.06 (d, 1H, 2, *J* = 4.8 Hz), 8.05 (d, 1H, 3, *J* = 5.1 Hz), 7.97 (s, 1H, 5'), 7.86 (d, 1H, 6, *J* = 5.1 Hz), 7.79 (d, 1H, 14', *J* = 1.7 Hz), 7.41 (d, 1H, 11', *J* = 8.8 Hz), 7.25 (d, 1H, 12, *J* = 1.9 Hz), 6.84 (dd, 12', 1H, *J* = 8.6, 2.0 Hz), 6.45 (d, 1H, 17', *J* = 5.3 Hz), 5.81 (s, 2H, 3'), 4.75 (h, 1H, 1', *J* = 6.0 Hz), 4.57 (t, 2H, 6', *J* = 6.0 Hz), 3.70 (q, 2H, 7', *J* = 5.8 Hz), 2.96 (s, 3H, 13), 1.28 (d, 6H, 2', *J* = 6.0 Hz)	158.58 (9'), 151.38 (16'), 149.84 (1), 148.46 (15'), 143.94 (8), 142.72 (9), 140.90 (11), 137.85 (7), 134.60 (13'), 133.63 (4'), 128.52 (10'), 127.10 (14'), 124.39 (11'), 123.80 (5'), 123.31 (3), 122.36 (12'), 117.26 (4), 114.27 (11), 112.18 (6), 110.39 (2), 98.72 (12), 95.88 (17'), 69.48 (1'), 47.83 (3'), 42.33 (6'/7'), 23.03 (13), 21.80 (2')
**41**	3143, 3061, 2958, 1611, 1579, 1487, 1448, 1431, 1402, 1370, 1324, 1225, 1195, 1140, 1083, 1083, 1046, 1022, 980, 909, 876, 818, 806, 734, 725, 690, 609	8.31 (d, 1H, 7, *J* = 5.4 Hz), 8.18 (d, , 1H, 15', *J* = 5.2 Hz), 8.04 (d, 1H, 10', *J* = 9.1 Hz), 7.97 (d, 1H, 6, *J* = 5.2 Hz), 7.93 (s, 1H, 2'), 7.79 (d, 1H, 13', *J* = 2.2 Hz), 7.77 (d, 1H, 3, *J* = 2.5 Hz), 7.64 (d, 1H, 12, *J* = 9.0 Hz), 7.42 (dd, 1H, 11', *J* = 9.0, 2.2 Hz), 7.40 (d, 1H, 7', *J* = 5.5 Hz), 7.14 (dd, 1H, 1, *J* = 9.0, 2.5 Hz), 6.44 (d, 1H, 16', *J* = 5.5 Hz), 5.82 (s, 2H, 2'), 4.55 (t, 2H, 5', *J* = 6.0 Hz), 3.86 (s, 3H, 1'), 3.69 (q, 2H, 6', *J* = 5.9 Hz), 2.99 (s, 3H, 13)	154.25 (8'), 152.03 (15'), 150.24 (2), 149.13 (14'), 144.47 (8), 142.30 (9), 137.69 (7), 136.38 (12'), 135.39 (3'), 134.08 (11), 128.37 (4), 127.74 (13'), 124.86 (10'), 124.32 (4'), 123.86 (11'), 121.64 (5), 118.27 (6), 117.79 (9'), 113.50 (1), 111.98 (12), 103.96 (3), 99.22 (16'), 56.14 (1'), 48.35 (2'), 42.76 (5'/6'), 23.68 (13)
**42**	3207, 2969, 1610, 1577, 1488, 1448, 1404, 1281, 1241, 1194, 1139, 1047, 990, 908, 880, 820, 807, 666, 639, 622	8.31 (d, 1H, 7, *J* = 5.4 Hz), 8.17 (d, 1H, 17', *J* = 5.2 Hz), 8.04 (d, 1H, 12', *J* = 9.1 Hz), 7.96 (d, 1H, 6, *J* = 5.2 Hz), 7.93 (s, 1H, 6'), 7.79 (d, 1H, 3, *J* = 2.2 Hz), 7.77 (d, 1H, 15', *J* = 2.5 Hz), 7.61 (d, 1H, 12, *J* = 9.0 Hz), 7.43 (dd, 1H, 13', *J* = 9.0, 2.2 Hz), 7.40 (d, 1H, 18', *J* = 5.7 Hz), 7.14 (dd, 1H, 3, *J* = 8.9, 2.5 Hz), 6.44 (d, 1H, 9', *J* = 5.5 Hz), 5.81 (s, 2H, 4'), 4.55 (t, 2H, 7', *J* = 6.0 Hz), 4.02 (t, 2H, 1', *J* = 6.5 Hz), 3.69 (q, 3H, 8', *J* = 5.9 Hz), 2.99 (s, 3H, 13), 1.79 (h, 2H, 2', *J* = 7.3 Hz), 1.03 (t, 3H, 3', *J* = 7.4 Hz)	153.61 (10'), 150.26 (17'), 149.11 (2), 144.47 (16'), 142.25 (8), 137.68 (9), 136.35 (7), 135.40 (14'), 134.09 (1), 128.40 (5'), 127.73 (4), 124.86 (15'), 124.33 (12'), 123.87 (6'), 121.69 (13'), 118.64 (5), 117.79 (6), 113.53 (1), 111.91 (12), 104.91 (3), 99.22 (18'), 70.17 (1'), 48.37 (4'), 42.77 (7'/8'), 23.67 (2'), 22.71 (13), 11.01 (3')
**43**	2969, 1612, 1579, 1450, 1370, 1326, 1197, 1141, 1047, 875, 806, 744	δ 8.32 (d, 1H, 7, *J* = 6.96 Hz), 8.30 (d, 1H, 14', *J* = 5.1 Hz), 8.23 (d, 1H, 9', *J* = 7.8 Hz), 8.05 (d, 1H, 3, *J* = 9.0 Hz), 8.00 (d, 1H, 6, *J* = 5.1 Hz), 7.93 (s, 1H, 3'), 7.80 (d, 1H, 12', *J* = 1.7 Hz), 7.70 (d, 1H, 12, *J* = 8.4 Hz), 7.55 – 7.49 (m, 1H, 1), 7.42 (dd, 2H, 10', *J* = 8.9, 2.1 Hz), 7.32 – 7.24 (m, 1H, 2), 6.44 (d, 1H, 15', *J* = 5.4 Hz), 5.82 (s, 2H, 1'), 4.55 (t, 2H, 4', *J* = 6.0 Hz), 3.70 (q, 2H, 13, *J* = 5.9 Hz), 3.35 (q, 2H, 5', *J* = 7.4 Hz), 1.30 (t, 2H, 14, *J* = 7.4 Hz)	151.96 (14'), 150.28 (7'), 149.07 (13'), 146.77 (8), 144.26 (7), 141.52 (11'), 138.39 (2'), 134.29 (4), 134.10 (12'), 128.95 (9), 128.61 (12'), 127.69 (9), 124.88 (3'), 124.33 (3), 123.87 (2), 121.84 (1), 121.39 (8'), 120.28 (10'), 117.79 (5), 113.25 (6), 111.03 (12), 99.22 (15'), 48.37 (1'), 42.76 (4'), 40.67 (5'), 28.29 (13), 13.60 (14)
**44**	2964, 1611, 1581, 1452, 1368, 1324, 1196, 1142, 1047, 875, 738	8.31 (d, 1H, 14', *J* = 4.8 Hz), 8.29 (d, 1H, 7, *J* = 5.0 Hz), 8.23 (d, 1H, 9', *J* = 7.7 Hz), 8.04 (d, 1H, 3, *J* = 9.0 Hz), 8.00 (d, 1H, 6, *J* = 5.0 Hz), 7.91 (s, 1H, 3'), 7.79 (d, 1H, 12', *J* = 2.3 Hz),	151.51 (14'), 149.62 (7'), 148.63 (13'), 145.25 (8), 143.66 (11), 141.00 (9), 137.82 (7), 133.80 (11'), 133.45 (2'), 128.57 (4), 128.07 (12'), 127.23 (9'), 124.25 (3'), 123.70 (10'), 123.24 (1),

		7.70 (d, 1H, 12, *J* = 8.3 Hz), 7.52 (t, 1H, *J* = 7.6 Hz, 10'), 7.45 – 7.39 (m, 1H, 2), 7.38 (t, 1H, 6', *J* = 5.8 Hz), 7.27 (t, 1H, 1, *J* = 7.4 Hz), 6.44 (d, 1H, 15', *J* = 5.3 Hz), 5.80 (s, 1H, 1'), 4.55 (t, 2H, 4', *J* = 5.7 Hz), 3.69 (q, 1H, 5', *J* = 5.9 Hz), 3.26 (t, 2H, 13, *J* = 7.62 Hz), 1.77 (h, 2H, 14, *J* = 7.2 Hz), 0.93 (t, 2H, 15, *J* = 7.3 Hz)	121.26 (3'), 120.80 (8'), 119.71 (2), 117.22 (5), 112.65 (6), 110.44 (12), 98.64 (15'), 47.77 (1'), 42.19 (4'), 40.09 (5'), 36.65 (13), 22.05 (14), 13.79 (15)
**45**	2963, 1582, 1450, 1328, 1219, 1198, 1086, 1039, 872, 798, 739	8.33 (d, 1H, 14', *J* = 5.0 Hz), 8.24 (d, 1H, 7'/9', *J* = 7.8 Hz), 8.03 (d, 1H, 3, *J* = 9.0 Hz), 8.00 (d, 1H, 6, *J* = 5.1 Hz), 7.90 (s, 1H, 3'), 7.79 (s, 1H, 12'), 7.70 (d, 1H, 12, *J* = 8.4 Hz), 7.53 (ddd, 1H, 1, *J* = 8.3, 7.0, 1.2 Hz), 7.40 (dd, 1H, 2, *J* = 8.9, 1.3 Hz), 7.33 (t, 1H, 7', *J* = 5.5 Hz), 7.27 (t, 1H, 10', *J* = 7.4 Hz), 6.44 (d, 1H, 15', *J* = 4.9 Hz), 5.82 (s, 2H, 1'), 4.56 (t, 2H, 4', *J* = 6.0 Hz), 3.96 – 3.91 (m, 1H, 13), 3.68 (q, 2H, 5', 14, *J* = 5.9 Hz), 3.17 (d, 1H, 14, *J* = 5.3 Hz), 1.25 (d, 5H, 14, *J* = 6.6 Hz)	150.23 (8), 149.45 (7'), 143.64 (13'), 141.21 (9/11), 137.91 (7), 133.32 (11'), 132.73 (2'), 128.87 (4), 128.10 (12'), 124.18 (9'), 123.72 (3'), 123.19 (10), 121.18 (10'), 120.80 (8'), 121.18 (1/3), 119.70 (2), 112.46 (12), 110.37 (6), 47.74 (1'), 42.22 (4'), 40.53 (5'), 30.48 (13), 22.45 (14)

2. IR, MS and NMR spectra of compounds 6-17, 20-26 and 30-45

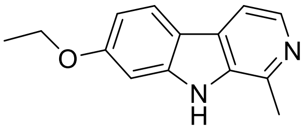

6

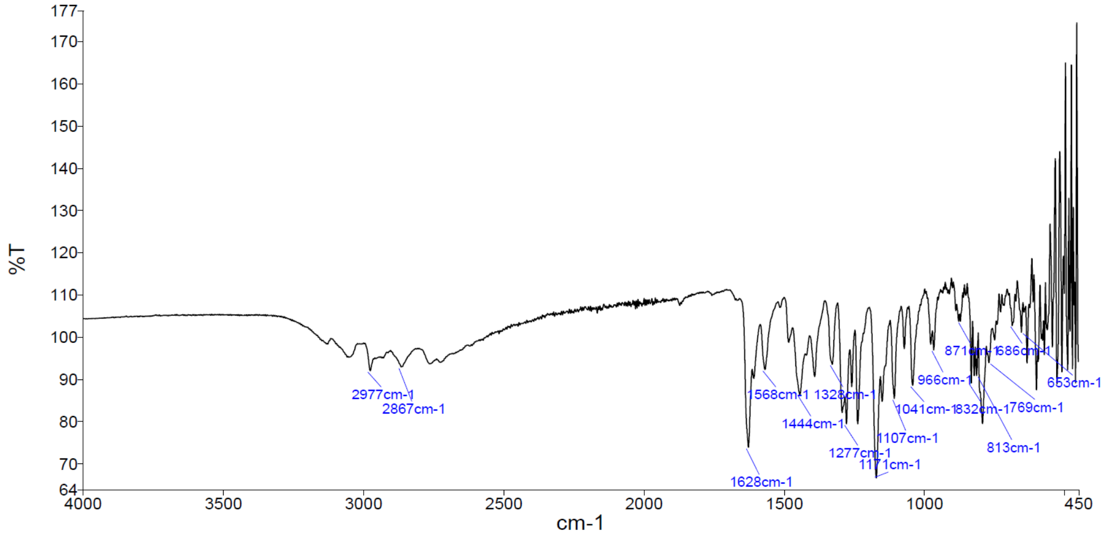

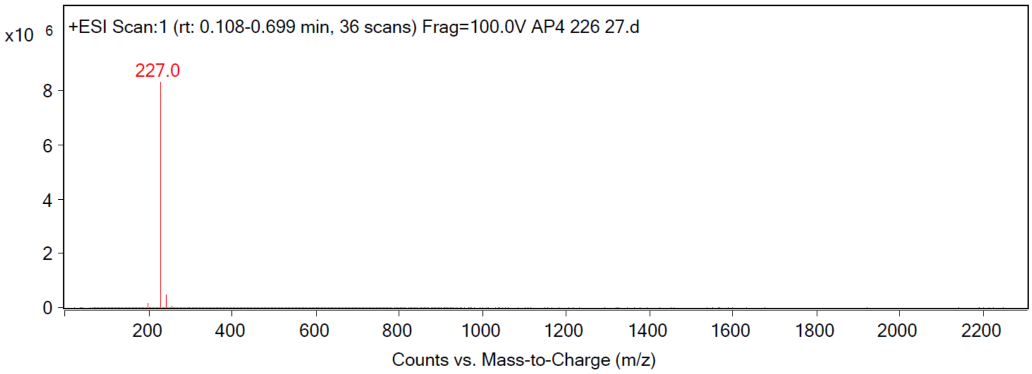

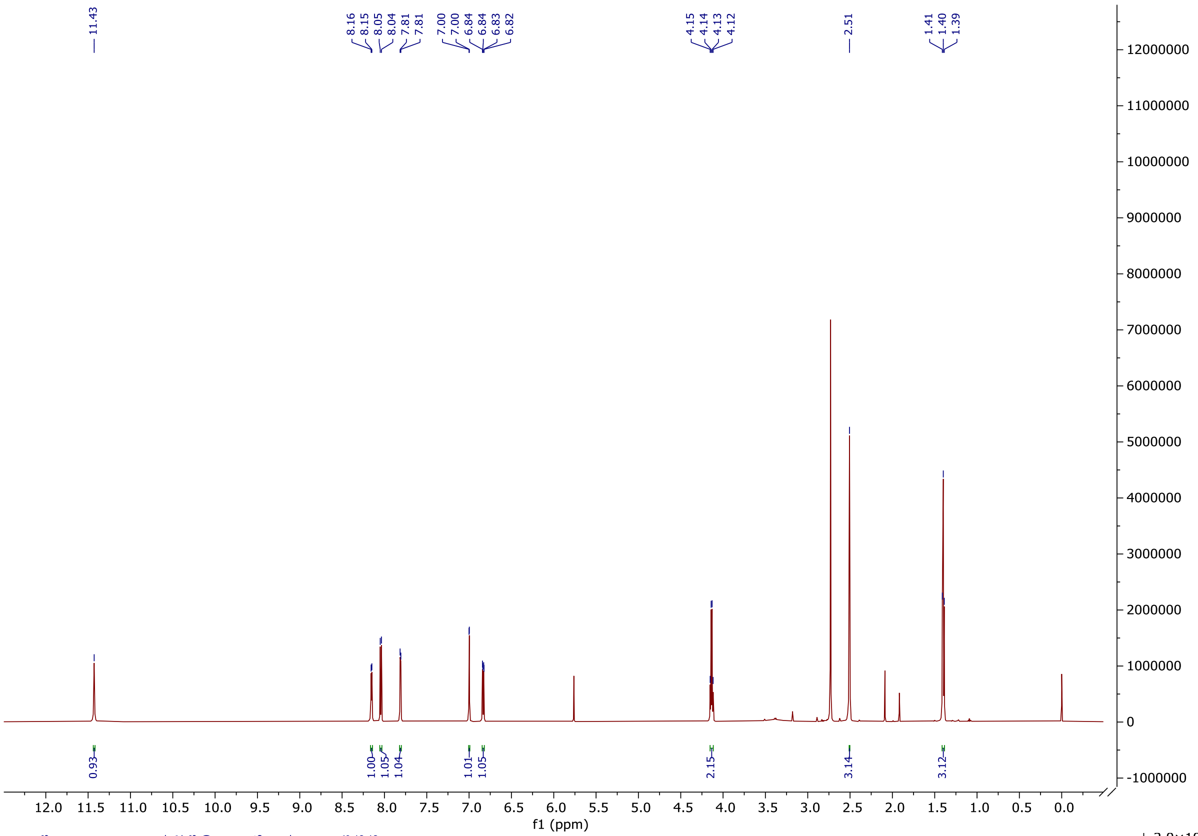
11.43
8.16
8.15
8.05
8.04
7.81
7.81
7.00
7.00
6.84
6.84
6.83
6.82
4.15
4.14
4.13
4.12
2.51
1.41
1.40
1.39
0.93
1.00
1.05
1.04
1.01
1.05
2.15
3.14
3.12
f1 (ppm)

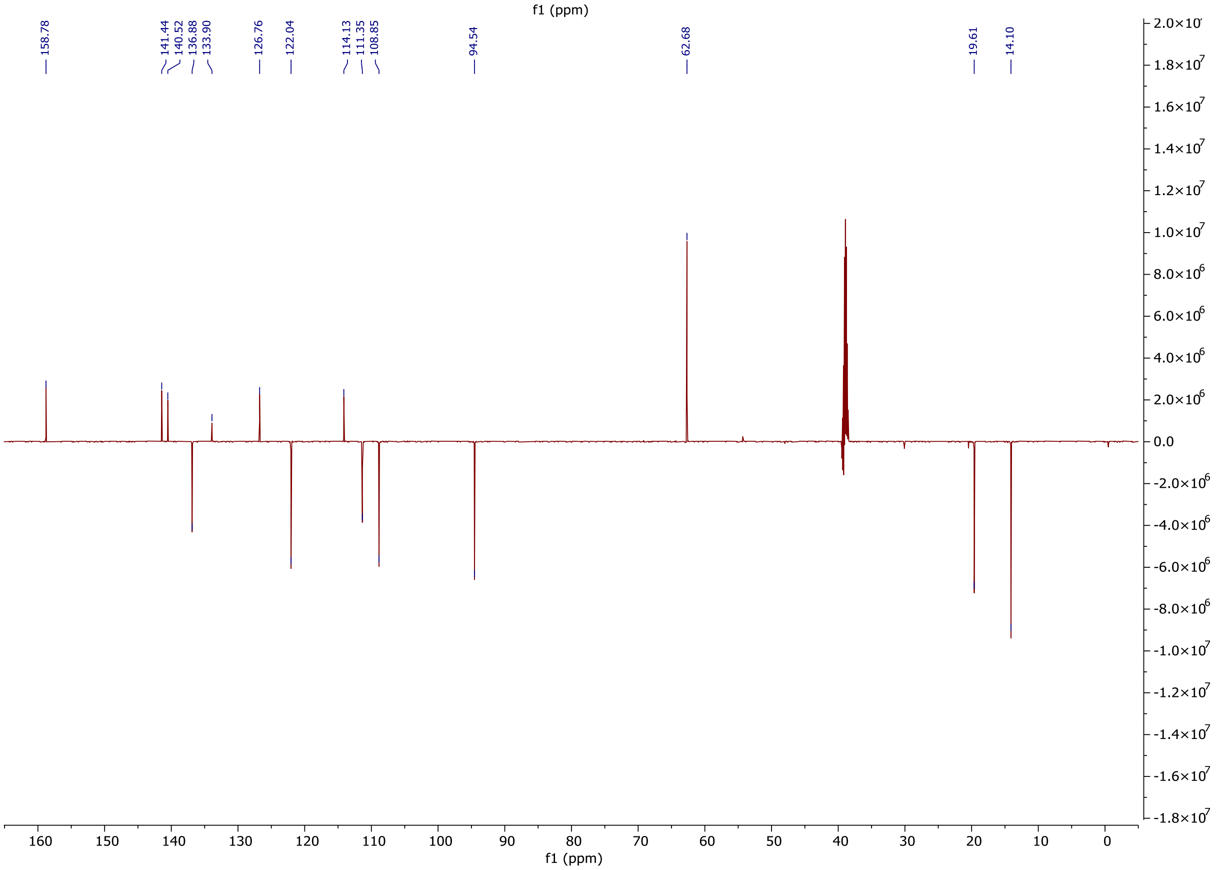
158.78
141.44
140.52
136.88
133.90
126.76
122.04
114.13
111.35
108.85
94.54
62.68
19.61
14.10
f1 (ppm)

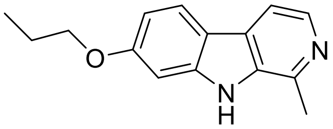
O
N
N
H

7

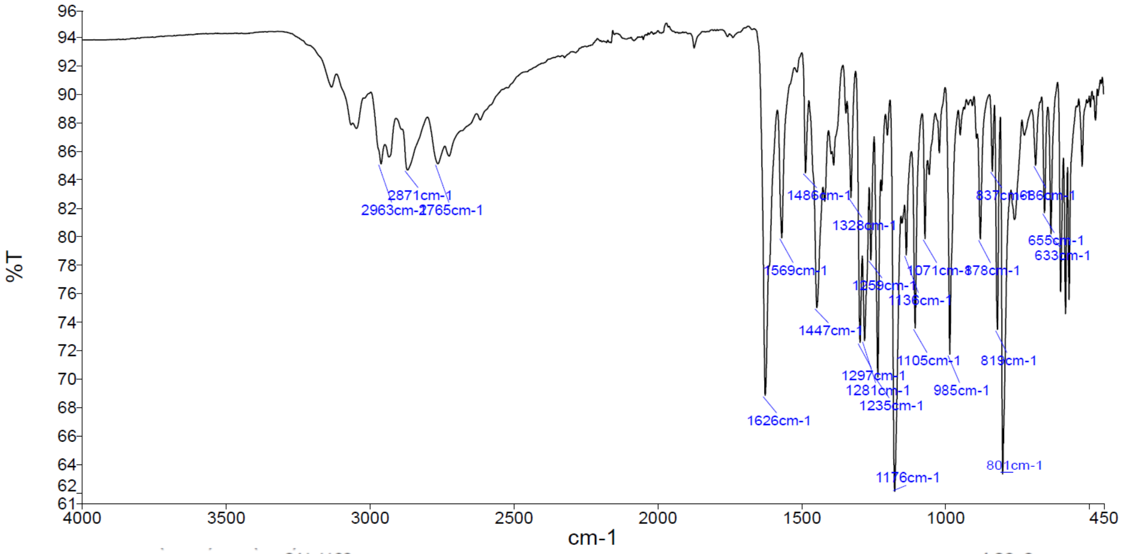
%T
cm-1
4000
3500
3000
2500
2000
1500
1000
450
2963cm-1
2871cm-1
2765cm-1
1626cm-1
1569cm-1
1486cm-1
1447cm-1
1328cm-1
1297cm-1
1281cm-1
1259cm-1
1235cm-1
1176cm-1
1136cm-1
1105cm-1
1071cm-1
985cm-1
878cm-1
837cm-1
819cm-1
801cm-1
686cm-1
655cm-1
633cm-1

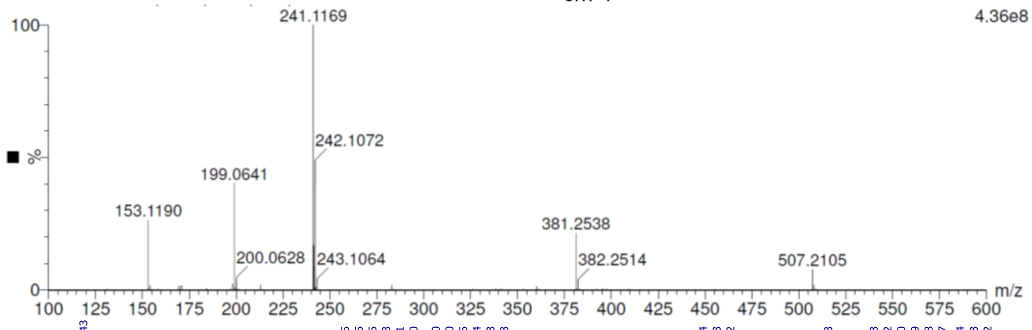
241.1169
4.36e8
242.1072
199.0641
153.1190
381.2538
200.0628
243.1064
382.2514
507.2105
%
m/z

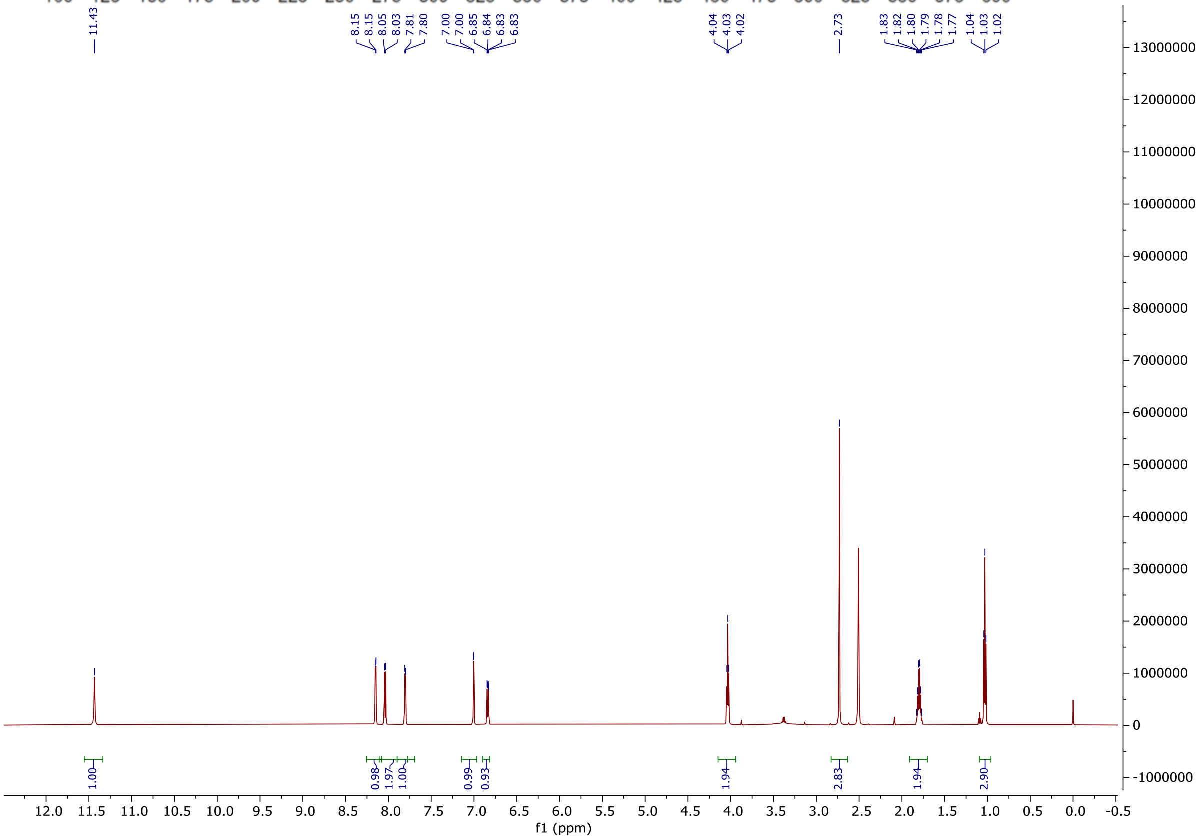
11.43
8.15
8.15
8.05
8.03
7.81
7.80
7.00
7.00
6.85
6.84
6.83
6.83
4.04
4.03
4.02
2.73
1.83
1.82
1.80
1.79
1.78
1.77
1.04
1.03
1.02
1.00
0.98
1.97
1.00
0.99
0.93
1.94
2.83
1.94
2.90
f1 (ppm)

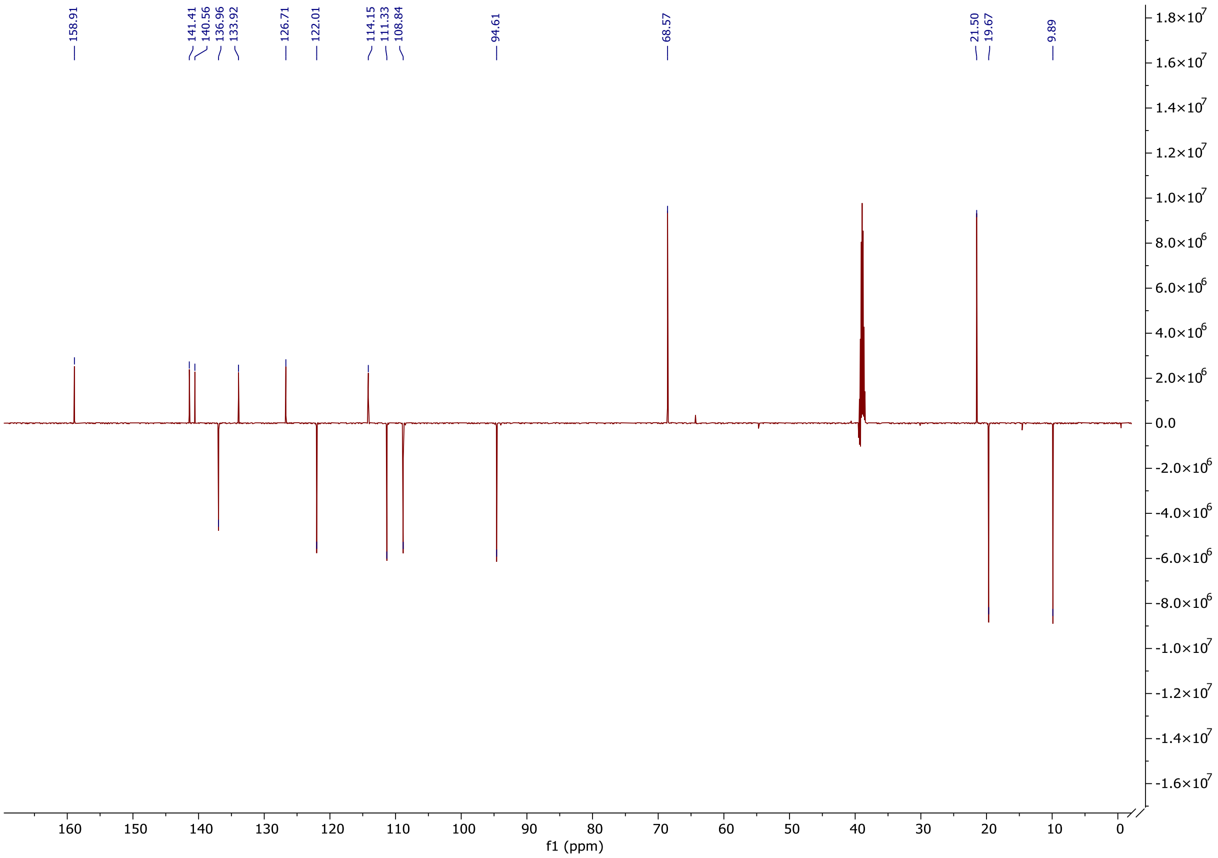

158.91
141.41
140.56
136.96
133.92
126.71
122.01
114.15
111.33
108.84
94.61
68.57
21.50
19.67
9.89
f1 (ppm)

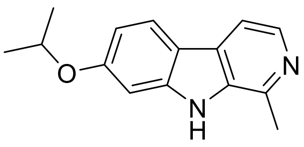

8

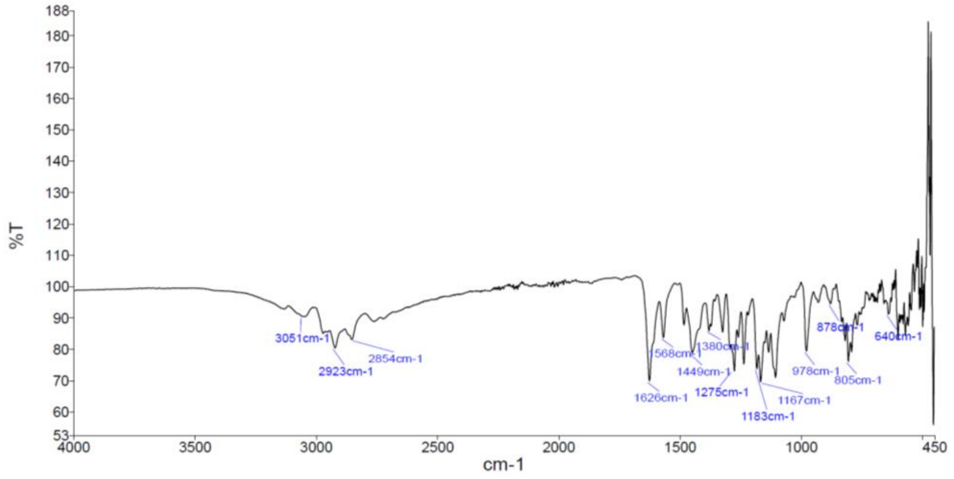

%T
cm-1
3051cm-1
2923cm-1
2854cm-1
1626cm-1
1568cm-1
1449cm-1
1380cm-1
1275cm-1
1183cm-1
1167cm-1
978cm-1
878cm-1
805cm-1
640cm-1

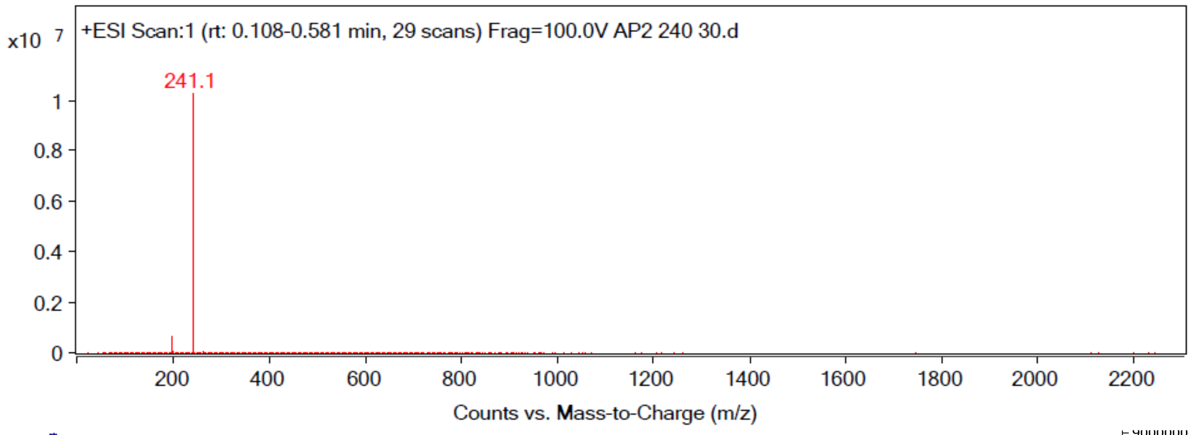

+ESI Scan:1 (rt: 0.108-0.581 min, 29 scans) Frag=100.0V AP2 240 30.d
x10 7
241.1
Counts vs. Mass-to-Charge (m/z)

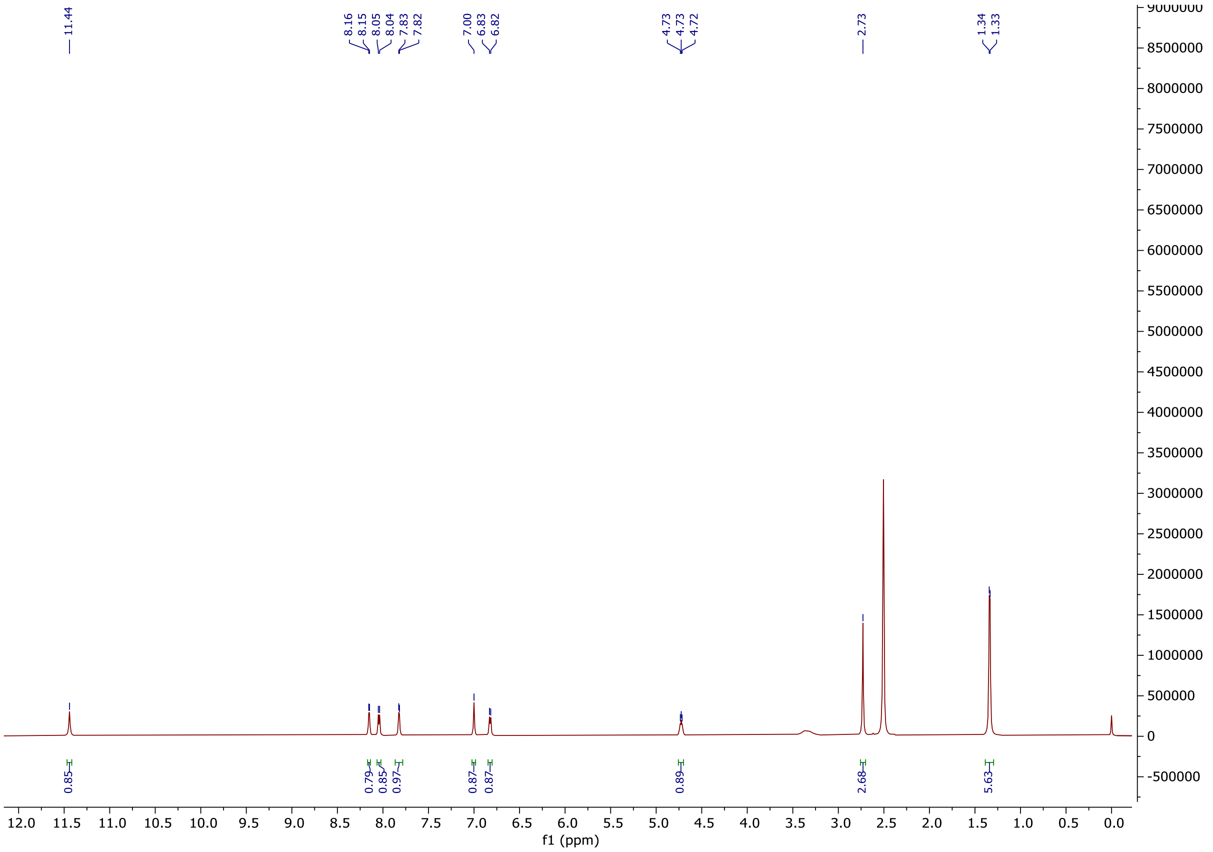

11.44
8.16
8.15
8.05
8.04
7.83
7.82
7.00
6.83
6.82
4.73
4.73
4.72
2.73
1.34
1.33
0.85
0.79
0.85
0.97
0.87
0.87
0.89
2.68
5.63
f1 (ppm)

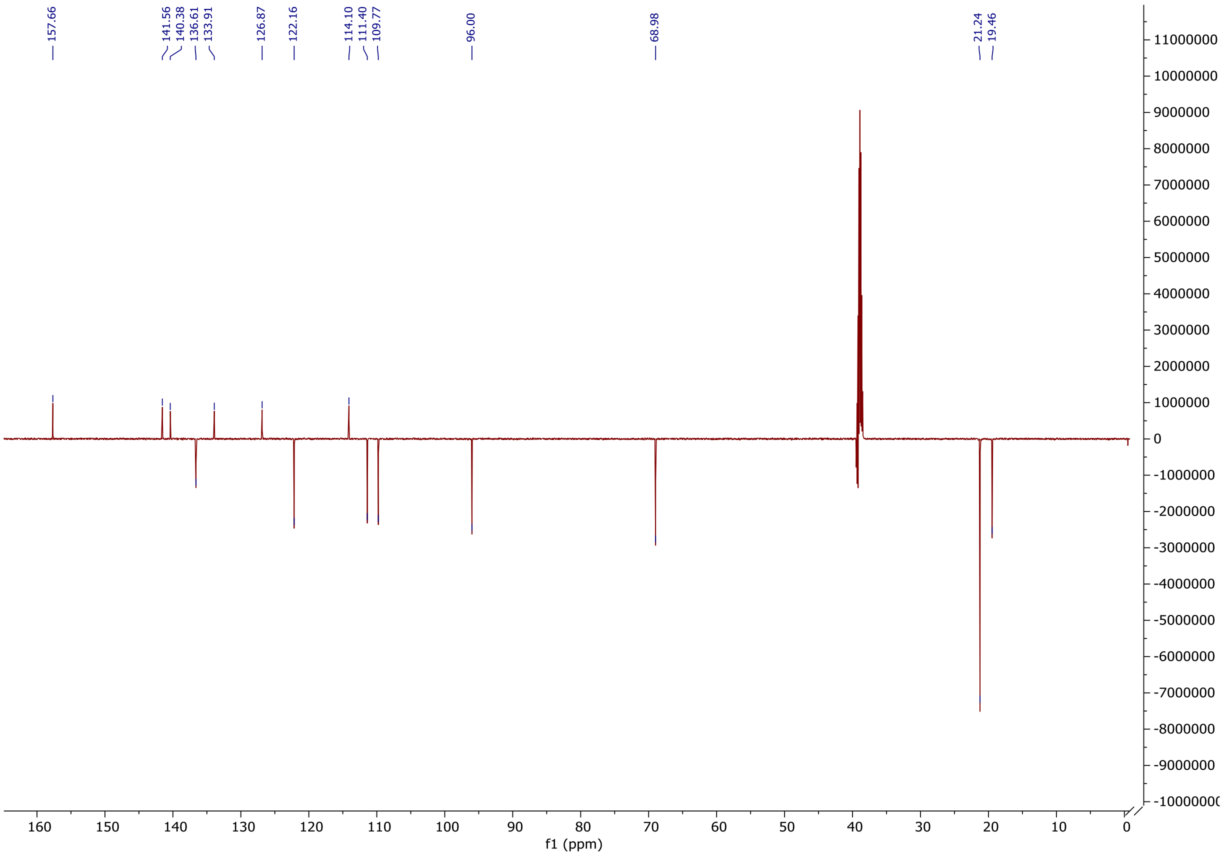
157.66
141.56
140.38
136.61
133.91
126.87
122.16
114.10
111.40
109.77
96.00
68.98
21.24
19.46
f1 (ppm)

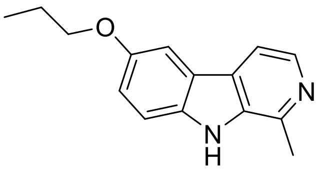

20

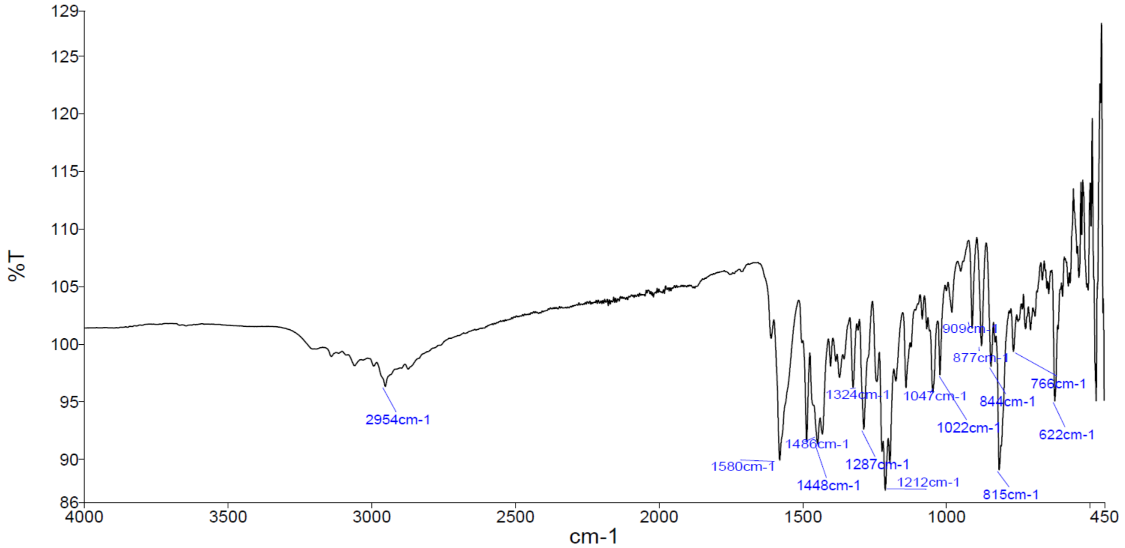
%T
cm-1
2954cm-1
1580cm-1
1486cm-1
1448cm-1
1324cm-1
1287cm-1
1212cm-1
1047cm-1
1022cm-1
909cm-1
877cm-1
844cm-1
815cm-1
766cm-1
622cm-1

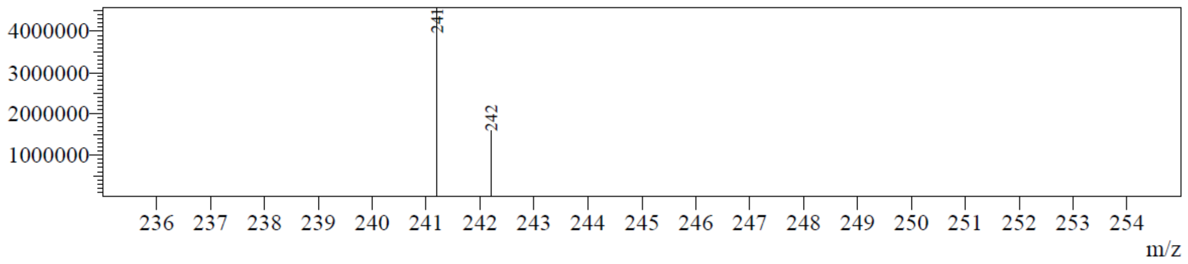
241
242
m/z

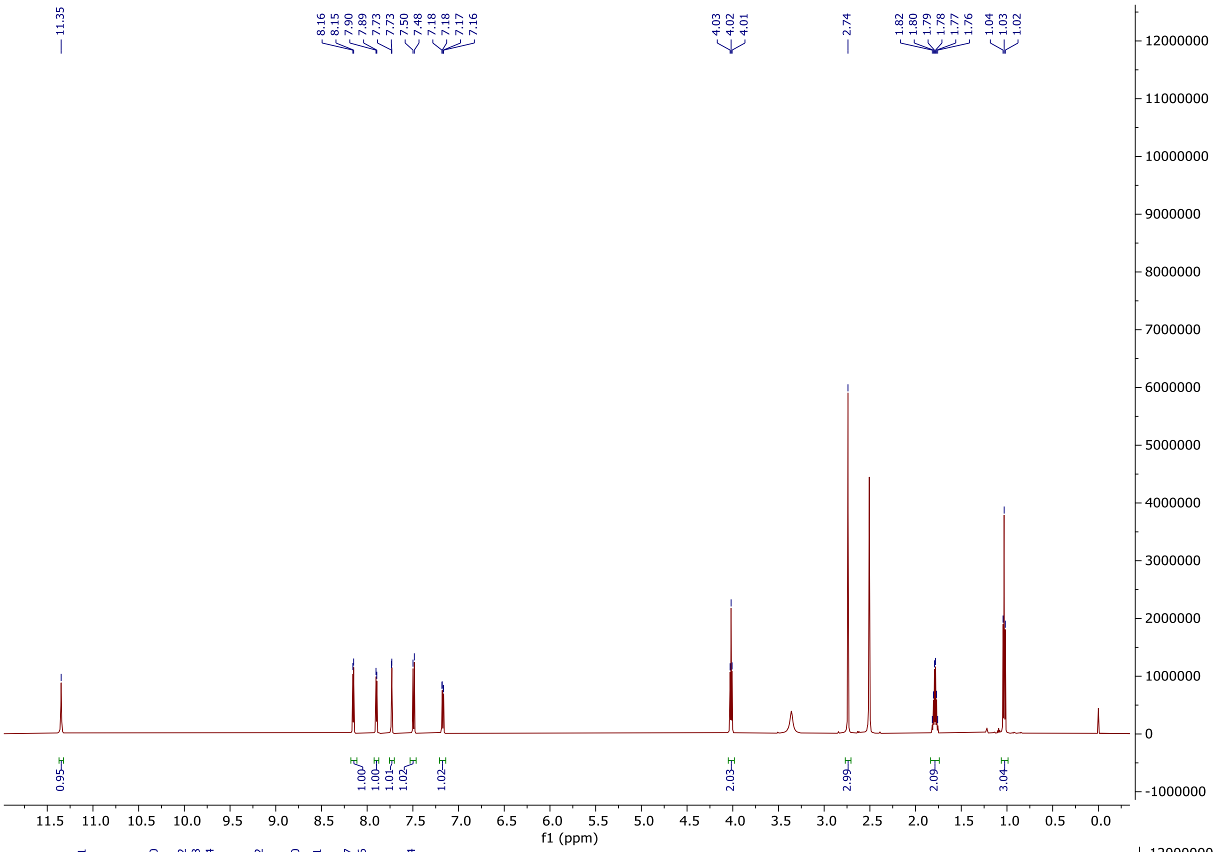

11.35
8.16
8.15
7.90
7.89
7.73
7.73
7.50
7.48
7.18
7.18
7.17
7.16
4.03
4.02
4.01
2.74
1.82
1.80
1.79
1.78
1.77
1.76
1.04
1.03
1.02
0.95
1.00
1.00
1.01
1.02
1.02
2.03
2.99
2.09
3.04
f1 (ppm)

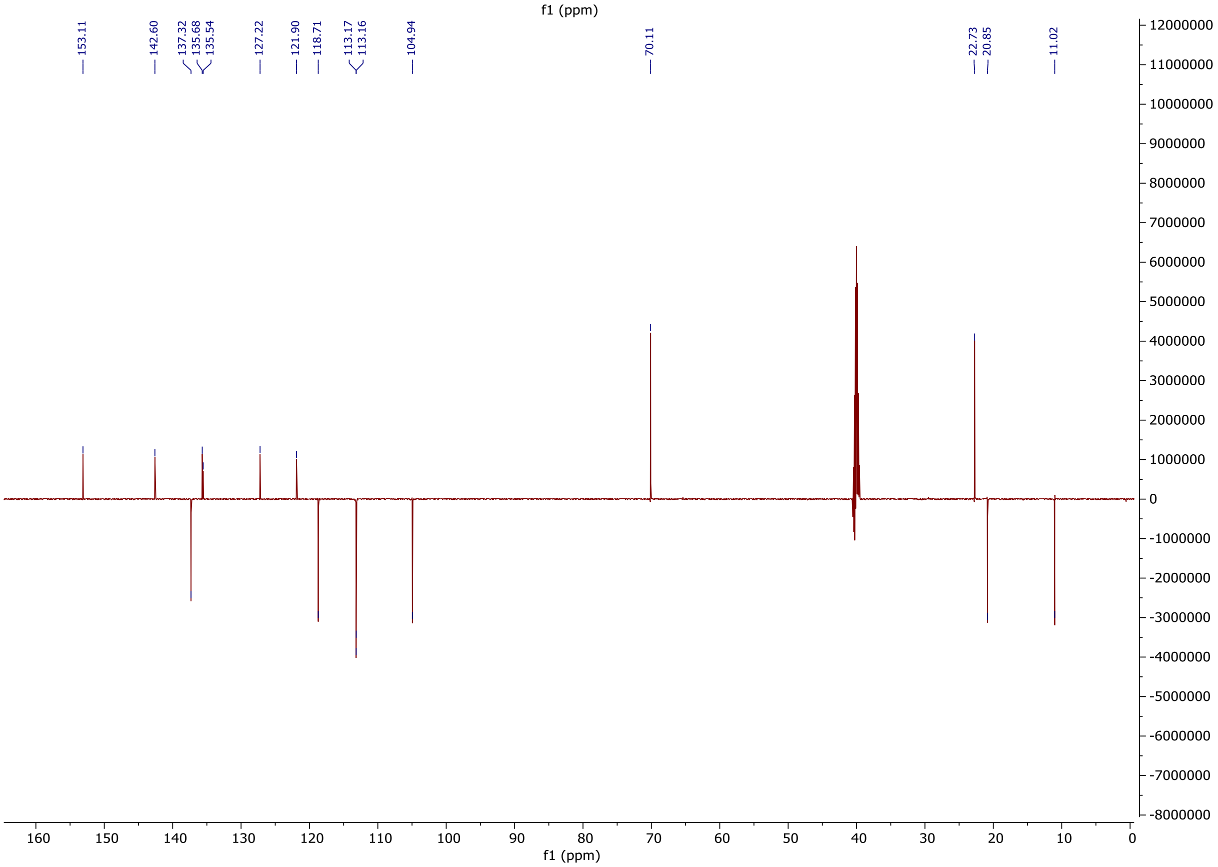

153.11
142.60
137.32
135.68
135.54
127.22
121.90
118.71
113.17
113.16
104.94
70.11
22.73
20.85
11.02
f1 (ppm)

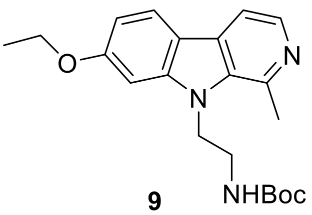
O
N
N
NHBoc

9

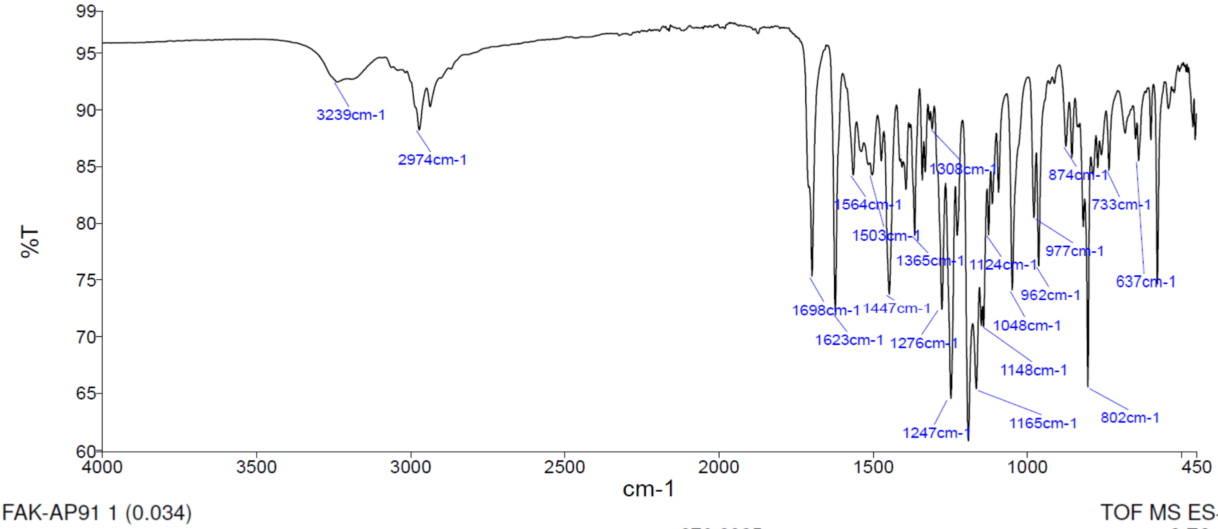
%T
cm-1
3239cm-1
2974cm-1
1698cm-1
1623cm-1
1564cm-1
1503cm-1
1447cm-1
1365cm-1
1308cm-1
1276cm-1
1247cm-1
1165cm-1
1148cm-1
1124cm-1
1048cm-1
977cm-1
962cm-1
874cm-1
802cm-1
733cm-1
637cm-1

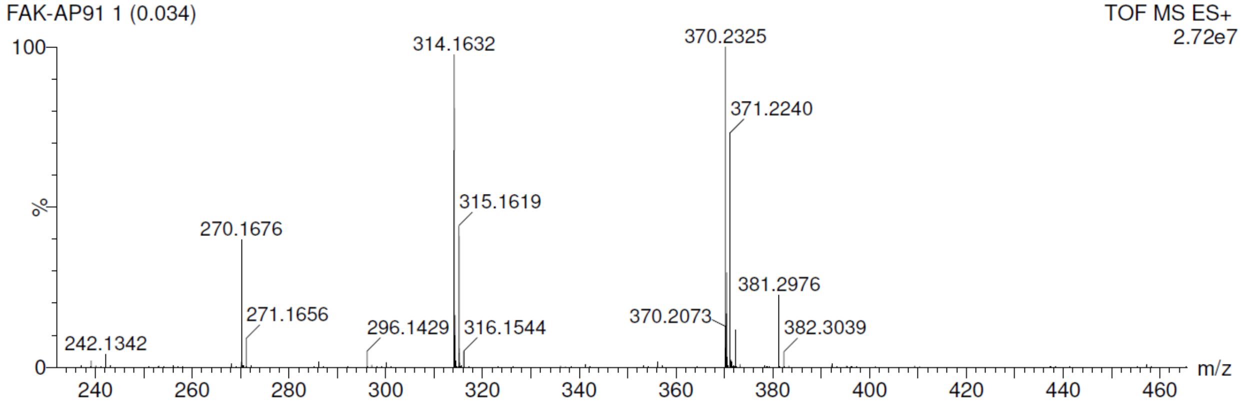
FAK-AP91 1 (0.034)
TOF MS ES+
2.72e7
314.1632
370.2325
371.2240
315.1619
270.1676
381.2976
271.1656
370.2073
296.1429
316.1544
382.3039
242.1342
%
m/z

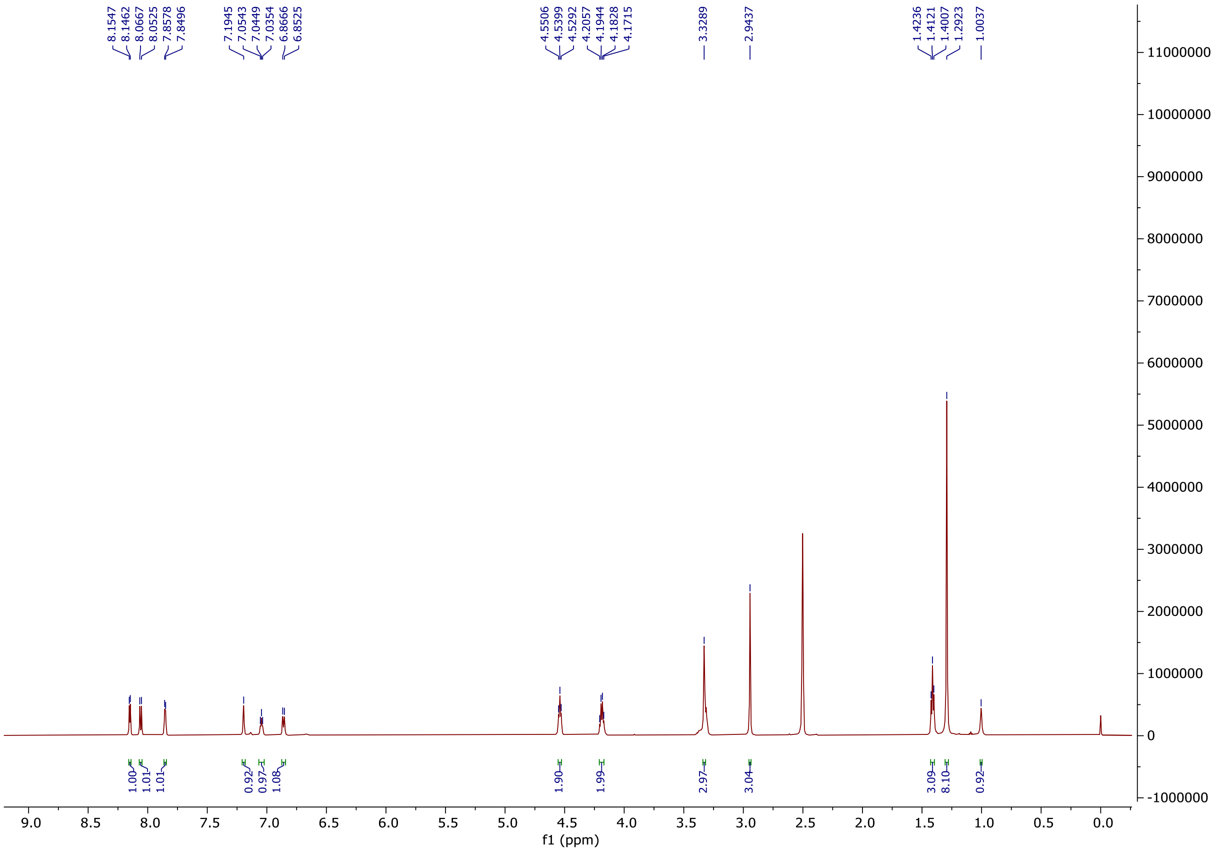
8.1547
8.1462
8.0667
8.0525
7.8578
7.8496
7.1945
7.0543
7.0449
7.0354
6.8666
6.8525
4.5506
4.5399
4.5292
4.2057
4.1944
4.1828
4.1715
3.3289
2.9437
1.4236
1.4121
1.4007
1.2923
1.0037
1.00
1.01
1.01
0.92
0.97
1.08
1.90
1.99
2.97
3.04
3.09
8.10
0.92
f1 (ppm)

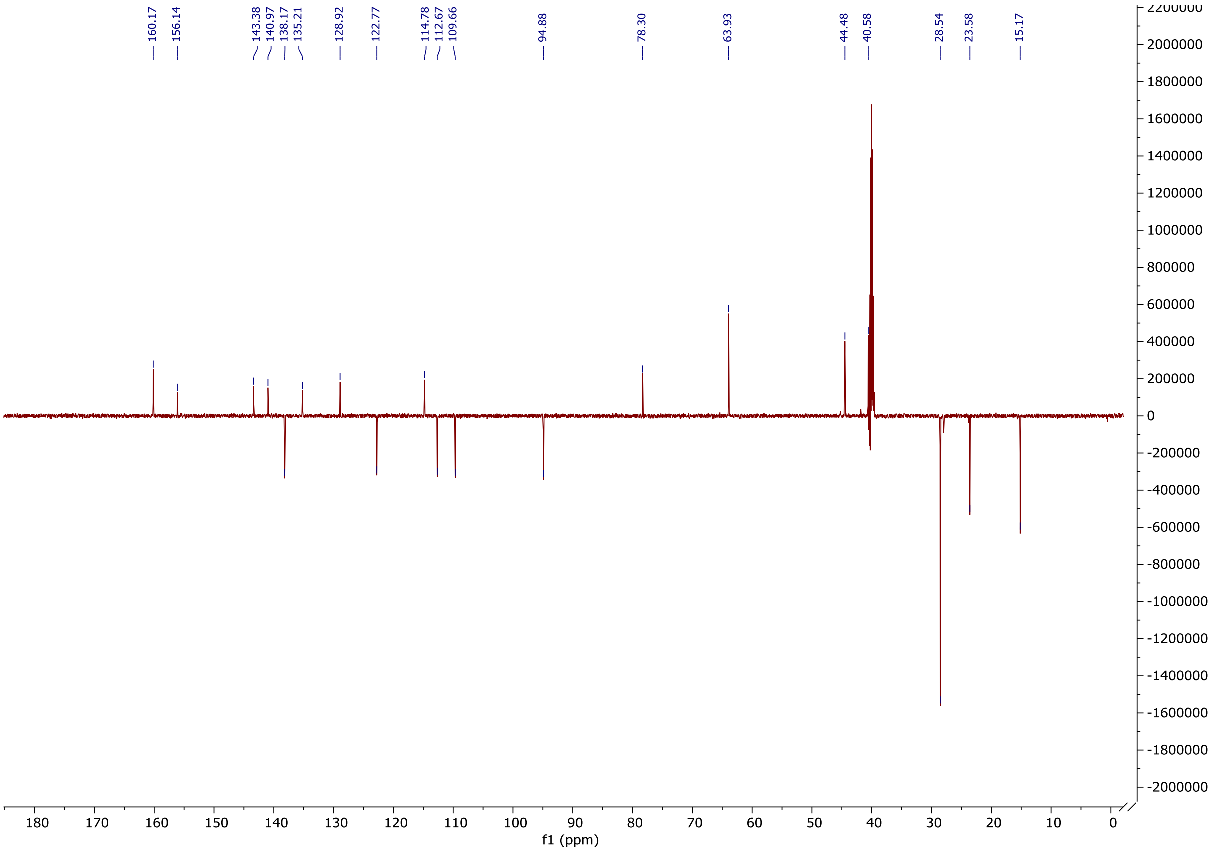
160.17
156.14
143.38
140.97
138.17
135.21
128.92
122.77
114.78
112.67
109.66
94.88
78.30
63.93
44.48
40.58
28.54
23.58
15.17
f1 (ppm)

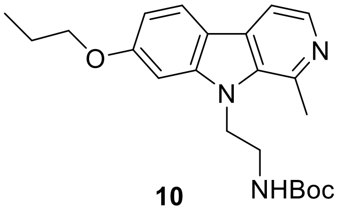
O
N
N
NHBoc

10

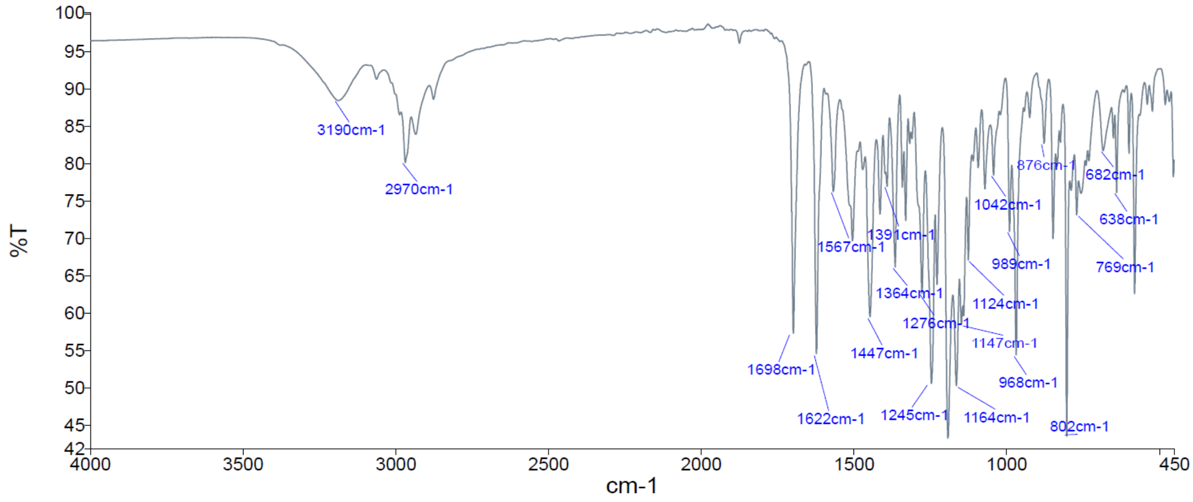
%T
cm-1
3190cm-1
2970cm-1
1698cm-1
1622cm-1
1567cm-1
1447cm-1
1391cm-1
1364cm-1
1276cm-1
1245cm-1
1164cm-1
1147cm-1
1124cm-1
1042cm-1
989cm-1
968cm-1
876cm-1
802cm-1
769cm-1
682cm-1
638cm-1

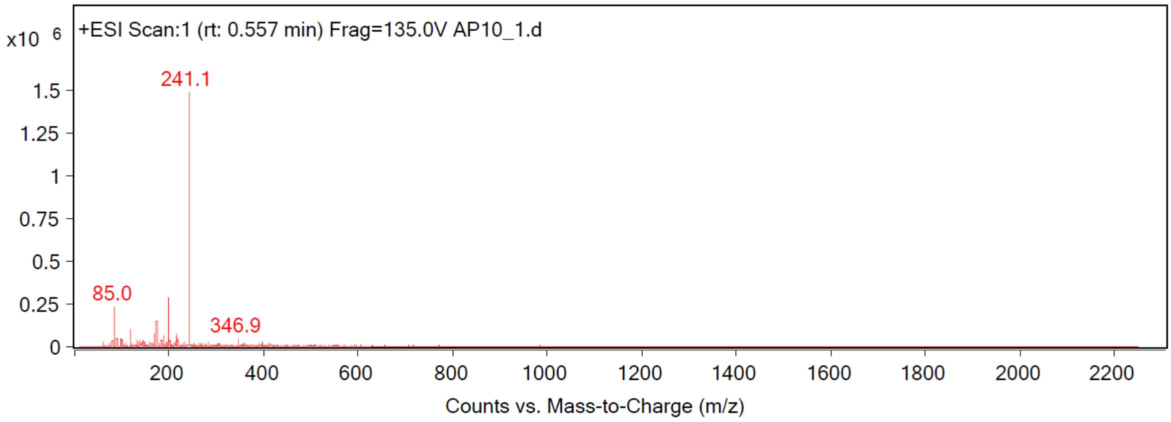

+ESI Scan:1 (rt: 0.557 min) Frag=135.0V AP10_1.d
x10 6
241.1
85.0
346.9
Counts vs. Mass-to-Charge (m/z)

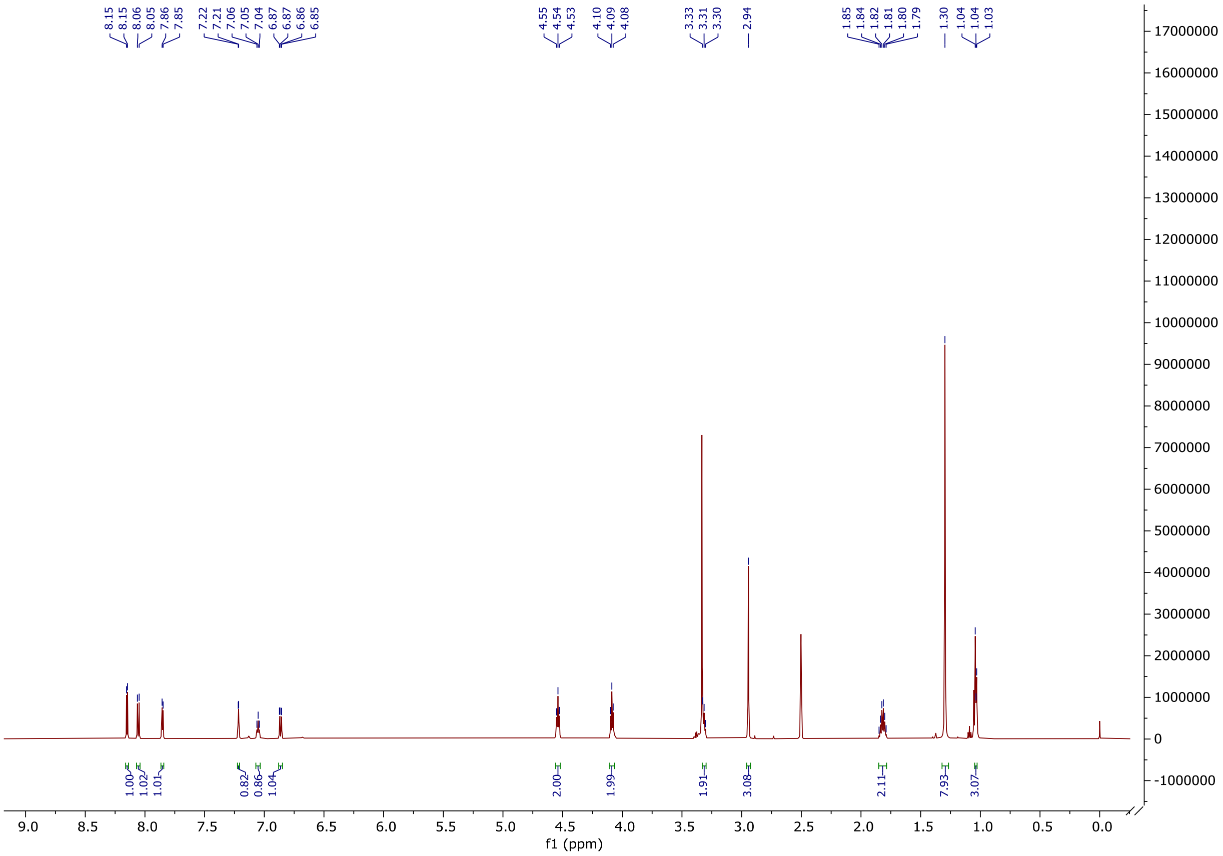

8.15 8.15 8.06 8.05 7.86 7.85 7.22 7.21 7.06 7.05 7.04 6.87 6.87 6.86 6.85 4.55 4.54 4.53 4.10 4.09 4.08 3.33 3.31 3.31 3.30 2.94 1.85 1.84 1.82 1.81 1.80 1.79 1.30 1.04 1.04 1.03
1.00 1.02 1.01 0.82 0.86 1.04 2.00 1.99 1.91 3.08 2.11 7.93 3.07
f1 (ppm)

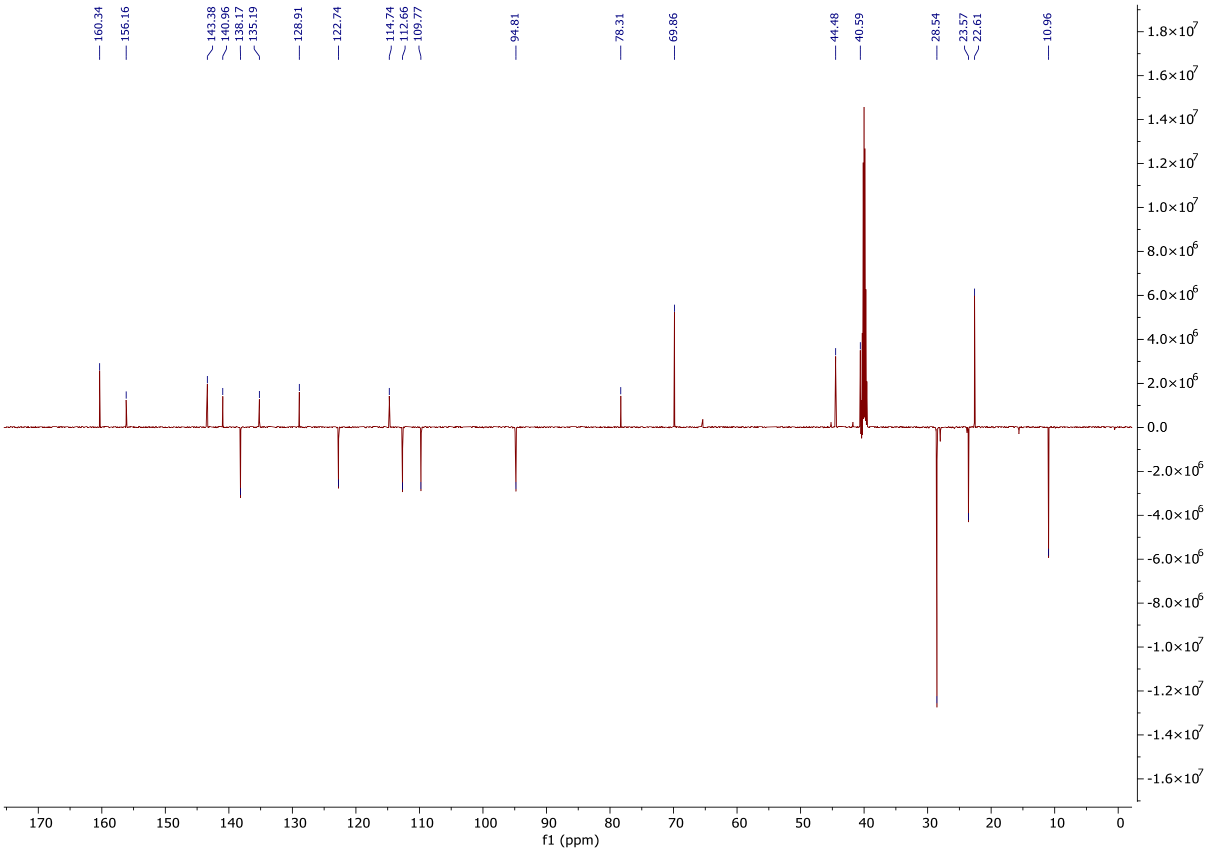

160.34
156.16
143.38
140.96
138.17
135.19
128.91
122.74
114.74
112.66
109.77
94.81
78.31
69.86
44.48
40.59
28.54
23.57
22.61
10.96
f1 (ppm)

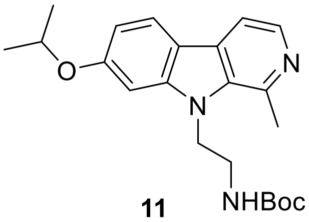

O
N
N
NHBoc

11

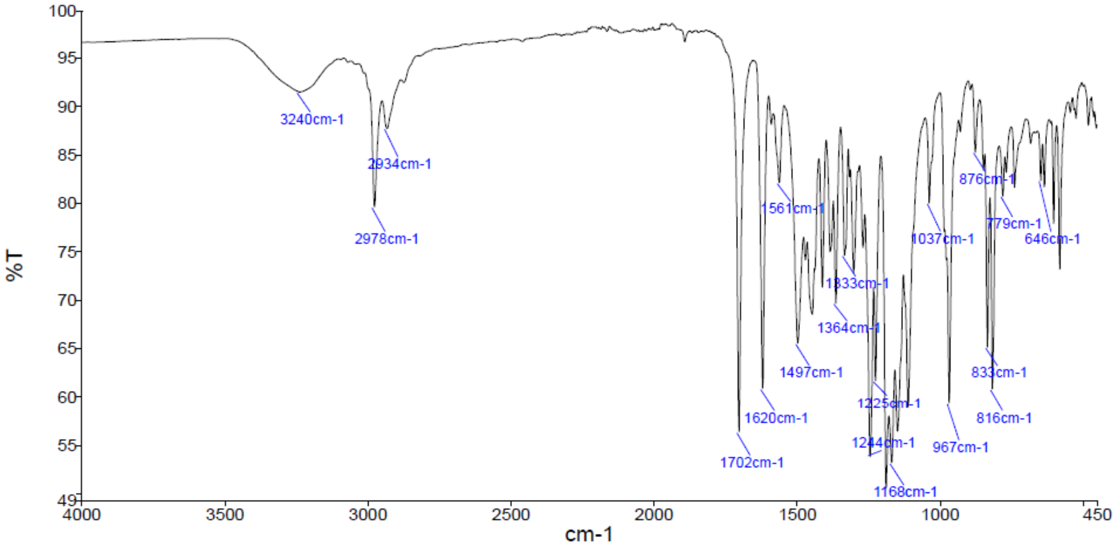

%T
cm-1
3240cm-1
2934cm-1
2978cm-1
1561cm-1
1333cm-1
1364cm-1
1497cm-1
1620cm-1
1702cm-1
1225cm-1
1244cm-1
1168cm-1
1037cm-1
967cm-1
876cm-1
779cm-1
646cm-1
833cm-1
816cm-1

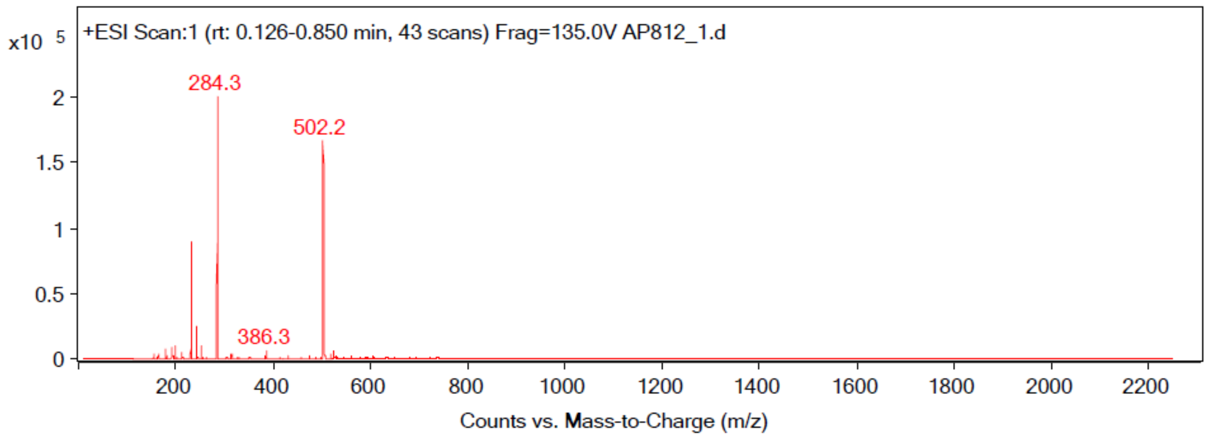

x10 5
+ESI Scan:1 (rt: 0.126-0.850 min, 43 scans) Frag=135.0V AP812_1.d
284.3
502.2
386.3
Counts vs. Mass-to-Charge (m/z)

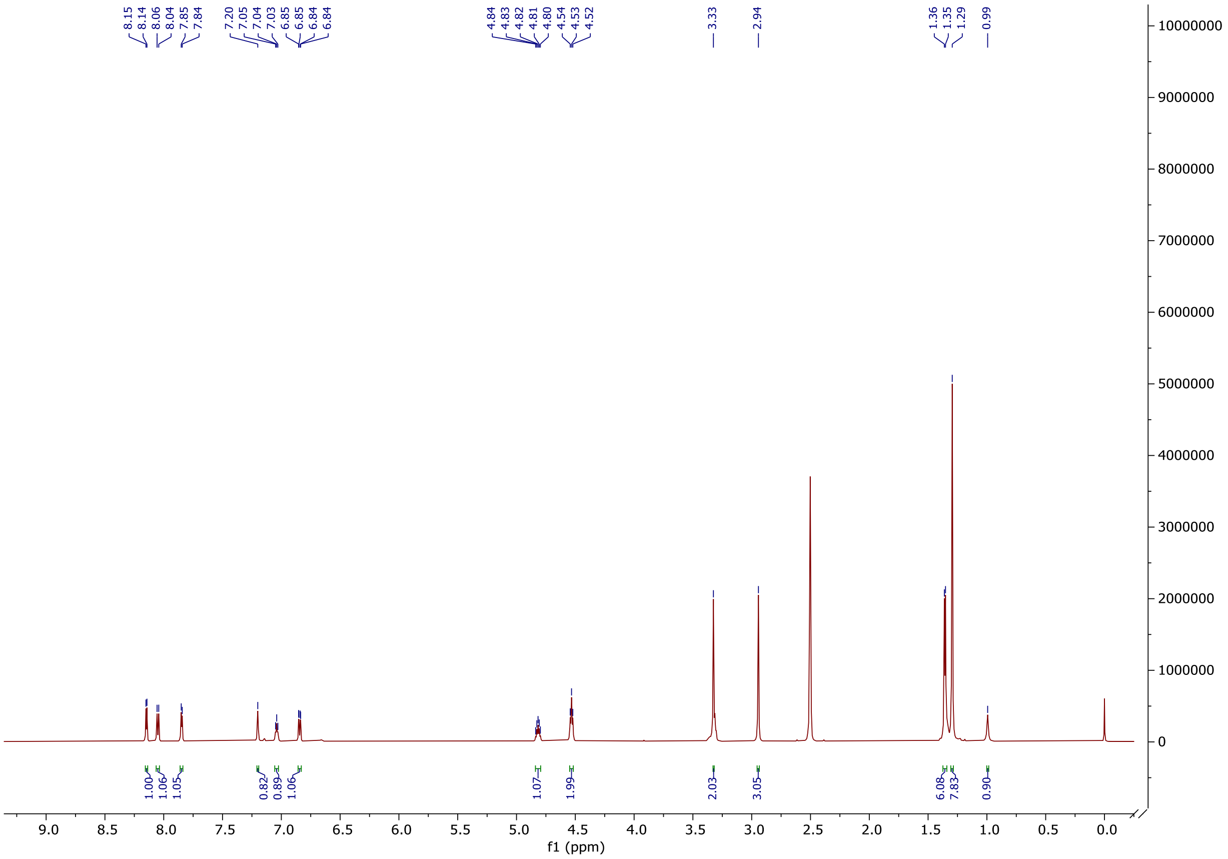

8.15
8.14
8.06
8.04
7.85
7.84
7.20
7.05
7.04
7.03
6.85
6.84
6.84
4.84
4.83
4.82
4.81
4.80
4.54
4.53
4.52
3.33
2.94
1.36
1.35
1.29
0.99
1.00
1.06
1.05
0.82
0.89
1.06
1.07
1.99
2.03
3.05
6.08
7.83
0.90
f1 (ppm)

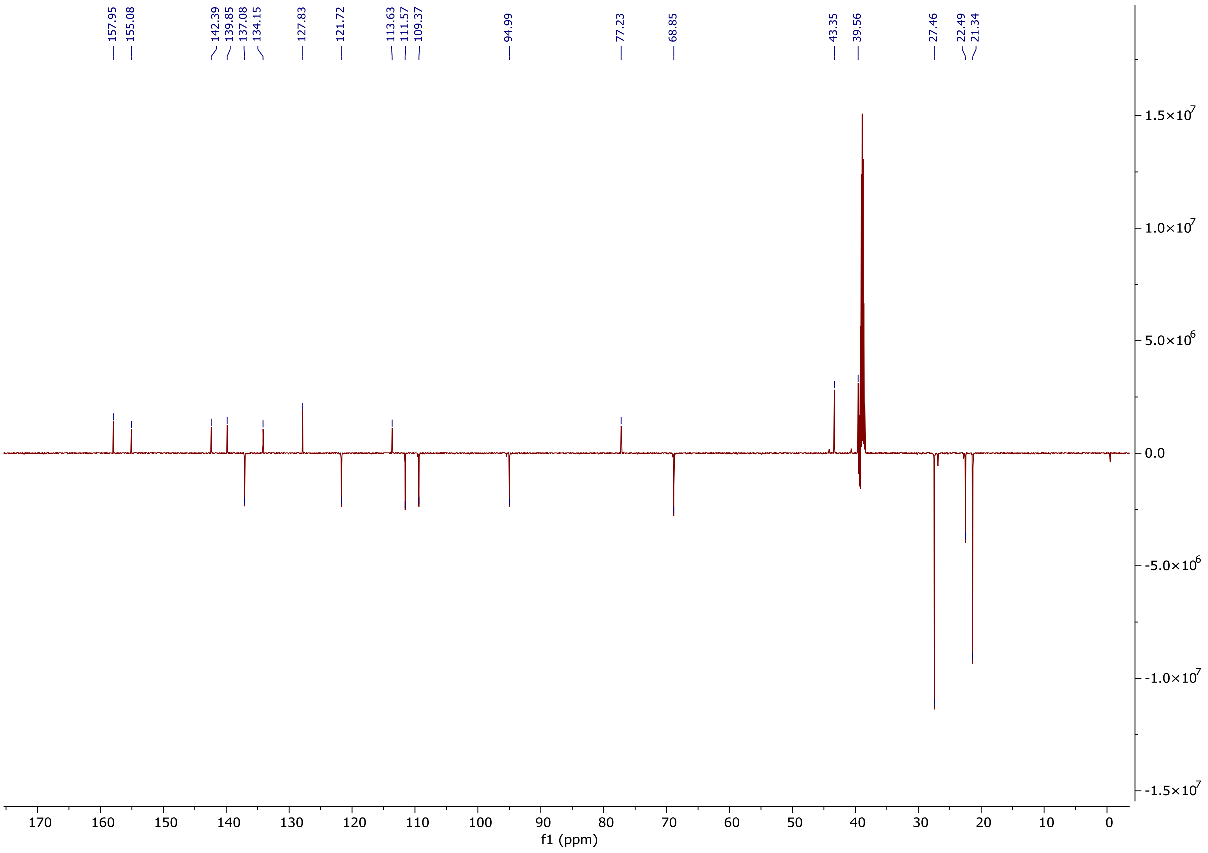
157.95
155.08
142.39
139.85
137.08
134.15
127.83
121.72
113.63
111.57
109.37
94.99
77.23
68.85
43.35
39.56
27.46
22.49
21.34
f1 (ppm)

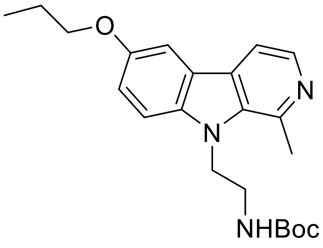
O
N
N
NHBoc

21

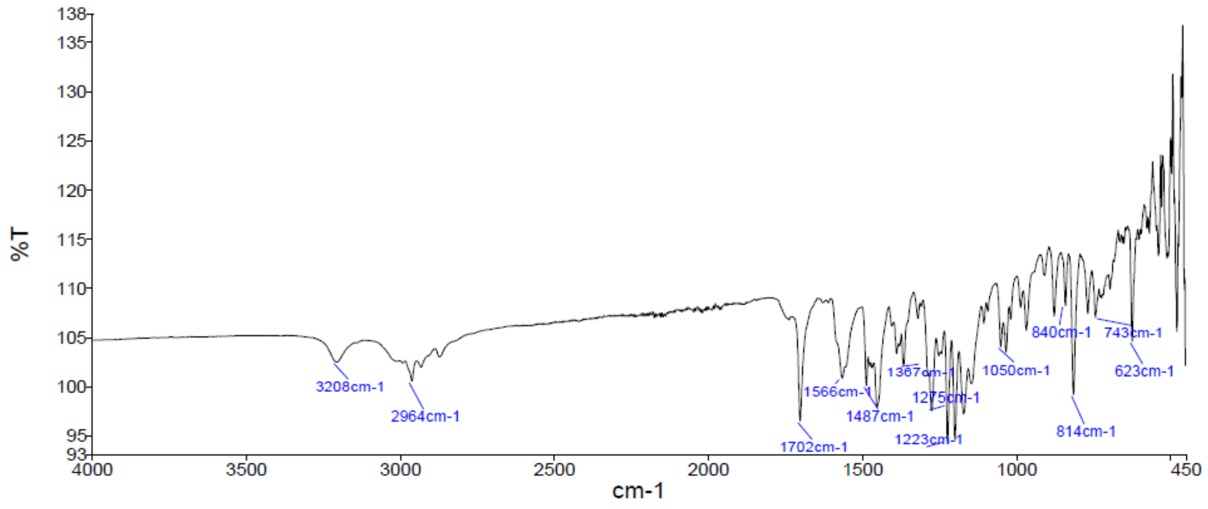
%T
cm-1
3208cm-1
2964cm-1
1702cm-1
1566cm-1
1487cm-1
1367cm-1
1275cm-1
1223cm-1
1050cm-1
840cm-1
814cm-1
743cm-1
623cm-1

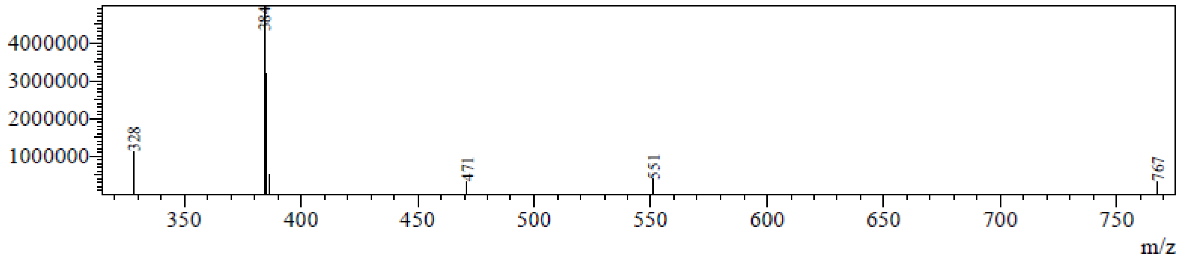
384
328
471
551
767
m/z

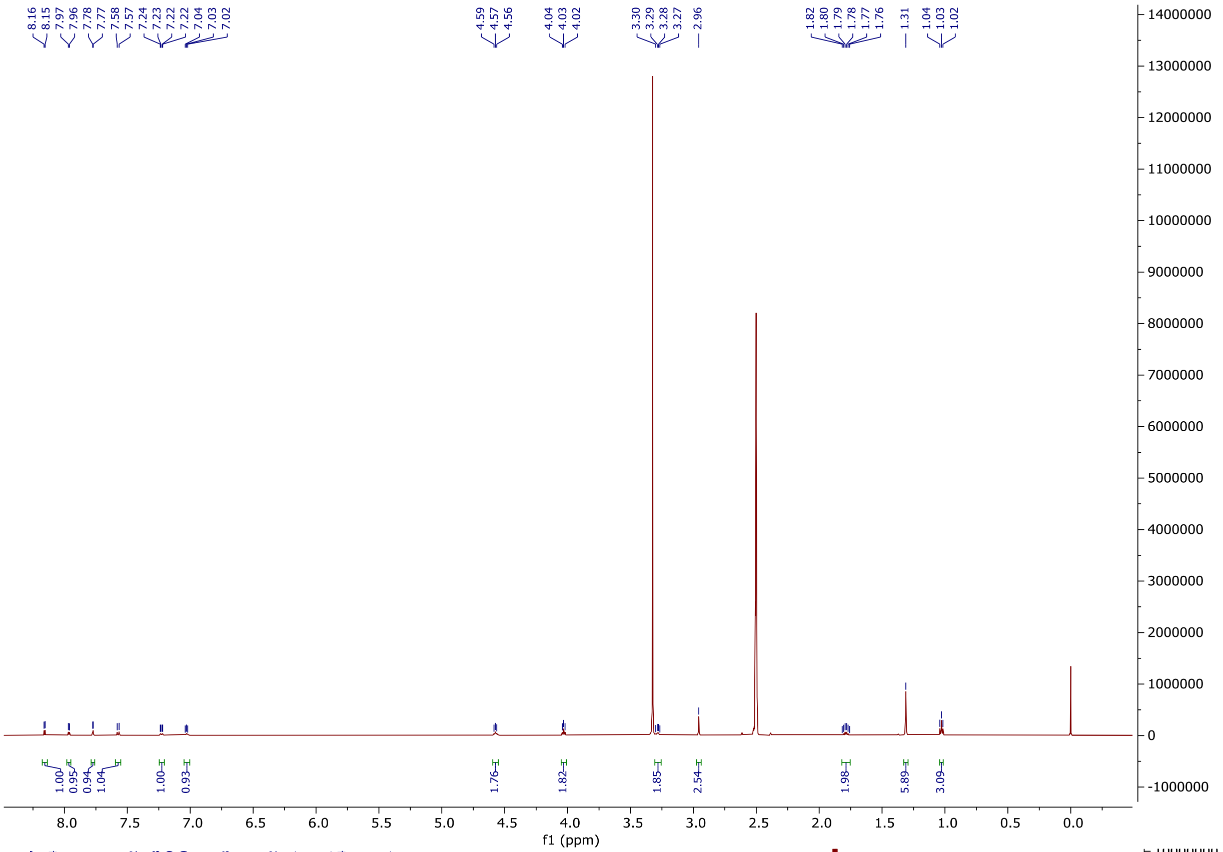
8.16 8.15 7.97 7.96 7.78 7.77 7.58 7.57 7.24 7.23 7.22 7.22 7.04 7.03 7.02
4.59 4.57 4.56
4.04 4.03 4.02
3.30 3.29 3.28 3.27
2.96
1.82 1.80 1.79 1.78 1.77 1.76
1.31
1.04 1.03 1.02
1.00 0.95 0.94 1.04 1.00 0.93 1.76 1.82 1.85 2.54 1.98 5.89 3.09
f1 (ppm)

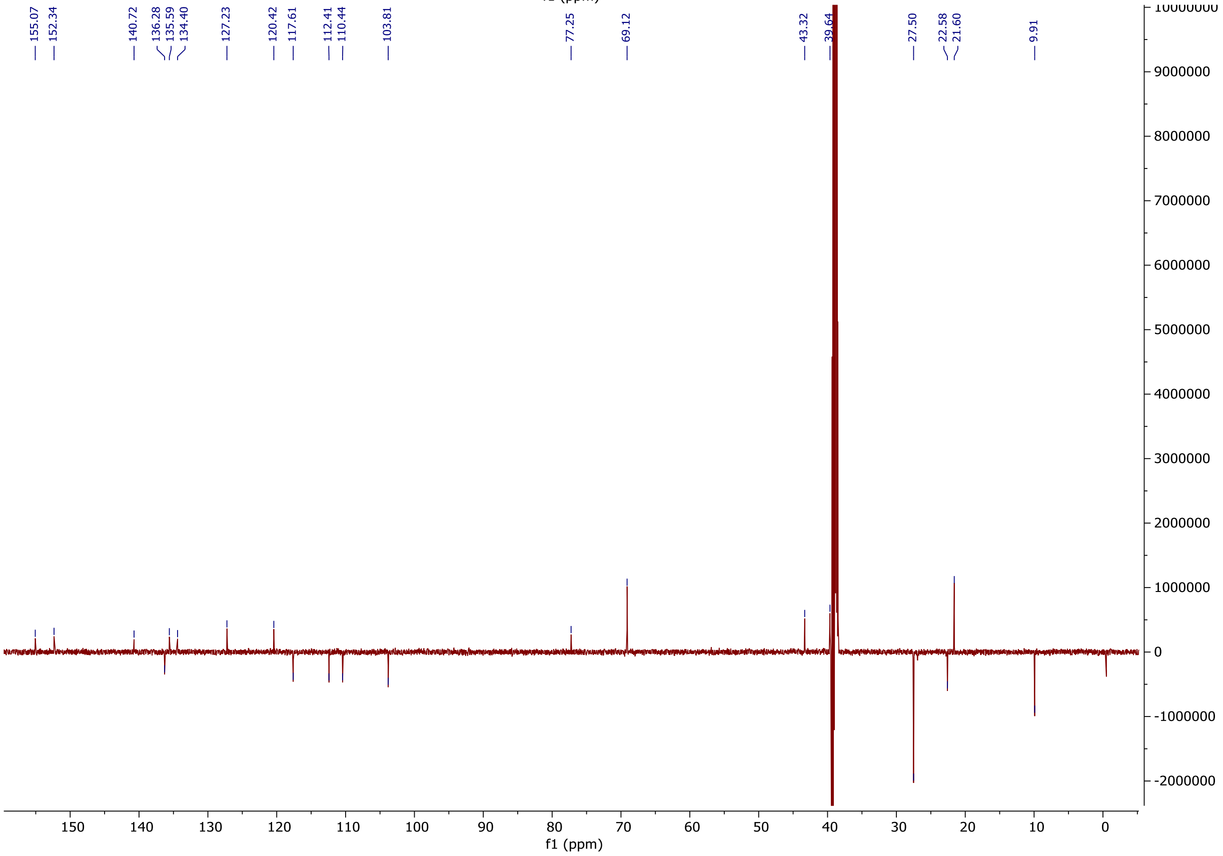
155.07 152.34 140.72 136.28 135.59 134.40 127.23 120.42 117.61 112.41 110.44 103.81 77.25 69.12 43.32 39.64 27.50 22.58 21.60 9.91
f1 (ppm)

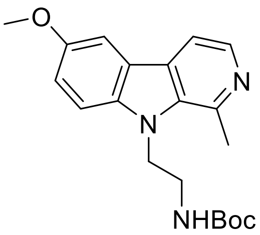
O
N
N
NHBoc

23

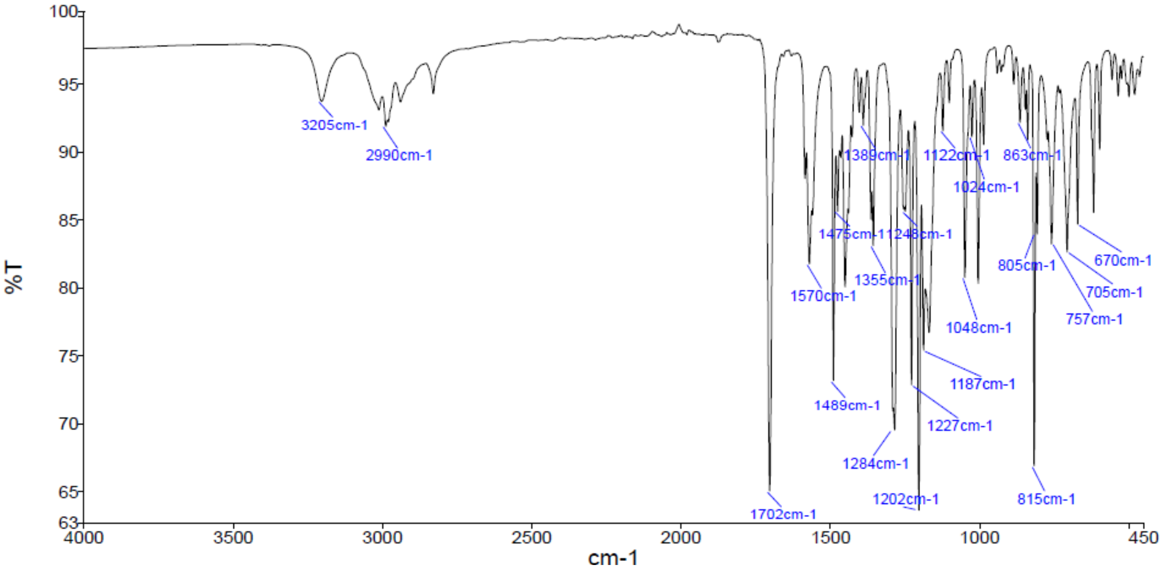
%T
cm-1
3205cm-1
2990cm-1
1702cm-1
1570cm-1
1489cm-1
1475cm-1
1389cm-1
1355cm-1
1284cm-1
1248cm-1
1227cm-1
1202cm-1
1187cm-1
1122cm-1
1048cm-1
1024cm-1
863cm-1
815cm-1
805cm-1
757cm-1
705cm-1
670cm-1

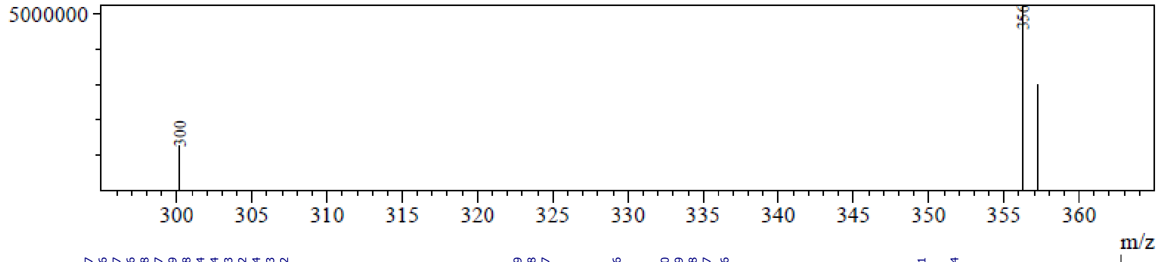
5000000
300
356
m/z

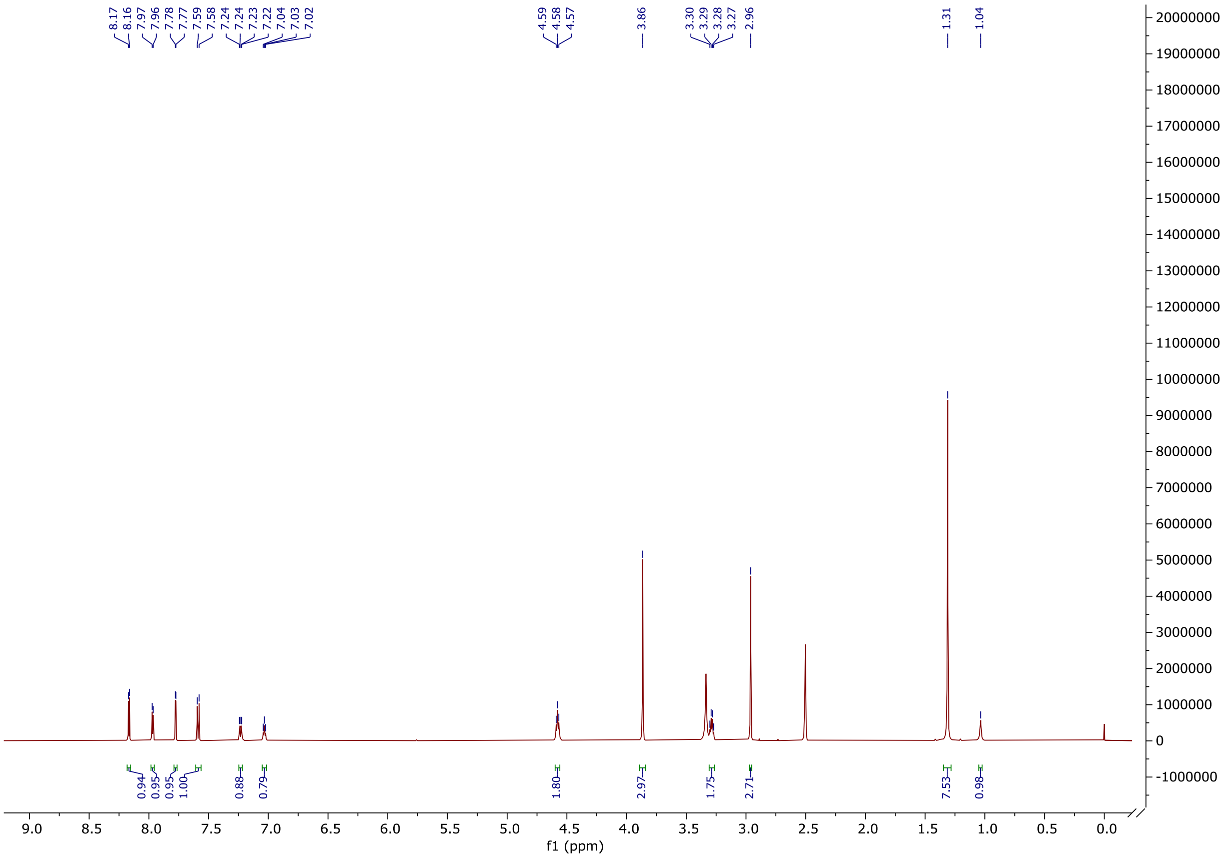
8.17 8.16 7.97 7.96 7.78 7.77 7.59 7.58 7.24 7.24 7.23 7.22 7.04 7.03 7.02
4.59 4.58 4.57
3.86
3.30 3.29 3.28 3.27
2.96
1.31
1.04
0.94 0.95 0.95 1.00 0.88 0.79 1.80 2.97 1.75 2.71 7.53 0.98
f1 (ppm)

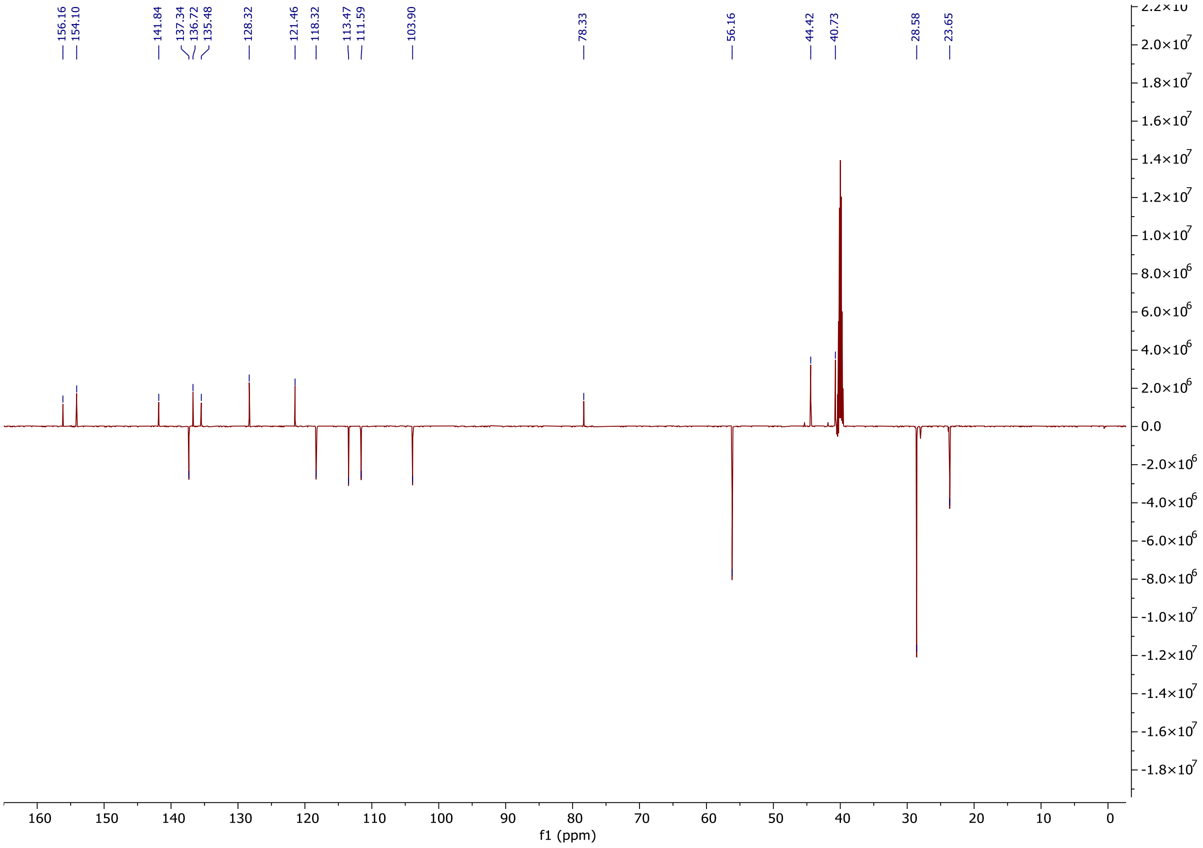

156.16
154.10
141.84
137.34
136.72
135.48
128.32
121.46
118.32
113.47
111.59
103.90
78.33
56.16
44.42
40.73
28.58
23.65
f1 (ppm)

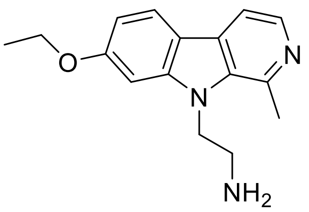
O
N
N
NH2

12

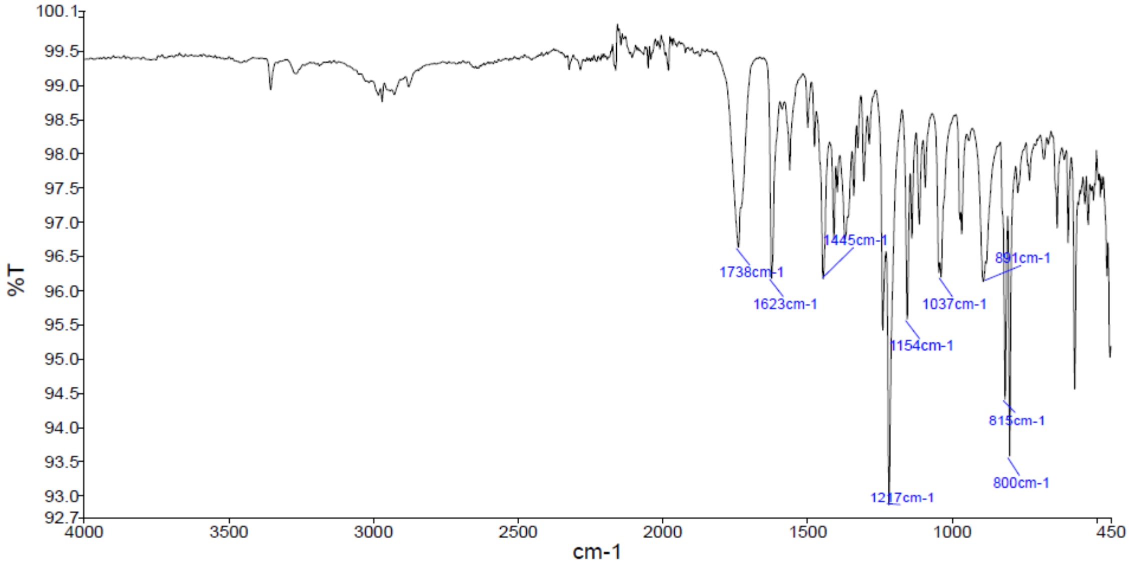
100.1
99.5
99.0
98.5
98.0
97.5
97.0
96.5
96.0
95.5
95.0
94.5
94.0
93.5
93.0
92.7
%T
4000
3500
3000
2500
2000
1500
1000
450
cm-1
1738cm-1
1623cm-1
1445cm-1
1154cm-1
1037cm-1
891cm-1
815cm-1
800cm-1
1217cm-1

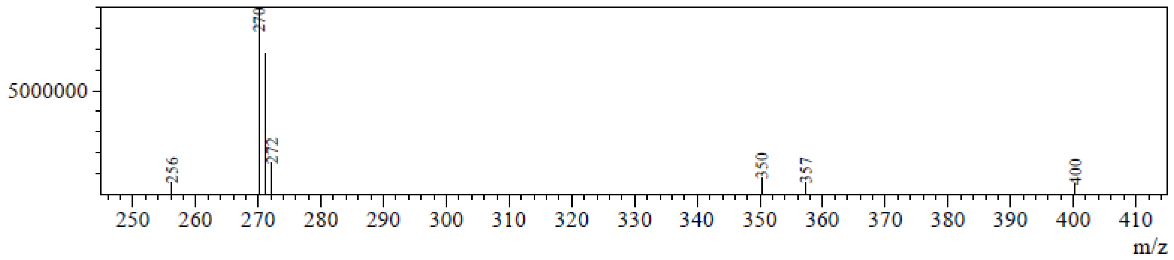
5000000
256
270
272
350
357
400
250
260
270
280
290
300
310
320
330
340
350
360
370
380
390
400
410
m/z

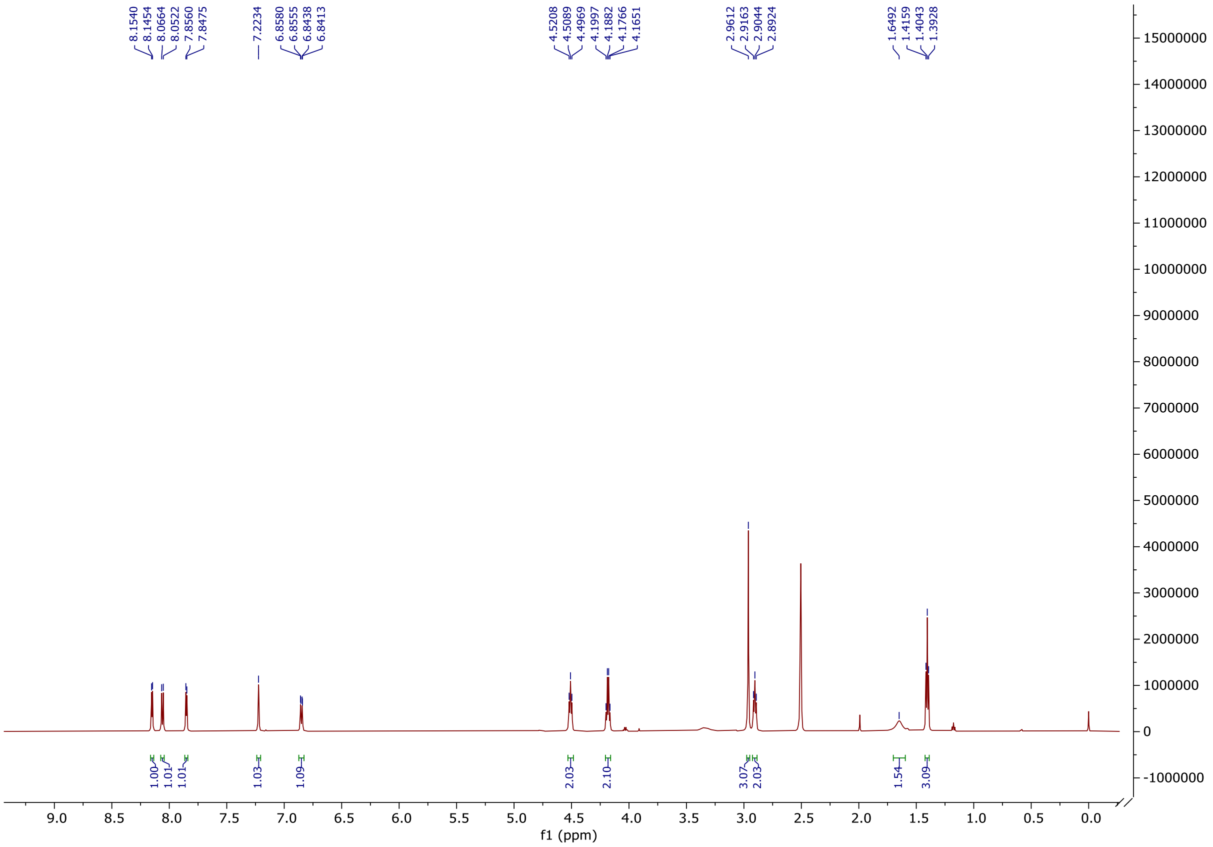
8.1540
8.1454
8.0664
8.0522
7.8560
7.8475
7.2234
6.8580
6.8555
6.8438
6.8413
4.5208
4.5089
4.4969
4.1997
4.1882
4.1766
4.1651
2.9612
2.9163
2.9044
2.8924
1.6492
1.4159
1.4043
1.3928
1.00
1.01
1.01
1.03
1.09
2.03
2.10
3.07
2.03
1.54
3.09
f1 (ppm)

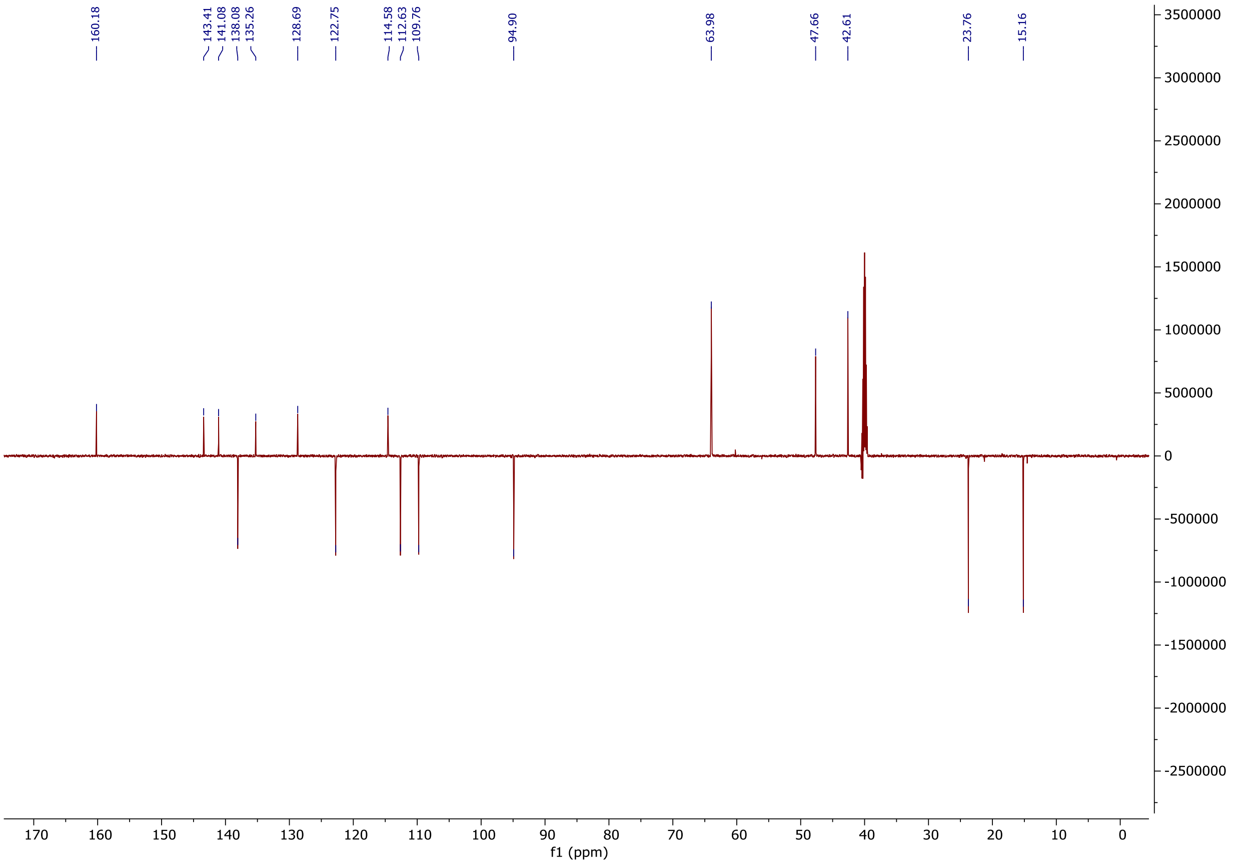
160.18
143.41
141.08
138.08
135.26
128.69
122.75
114.58
112.63
109.76
94.90
63.98
47.66
42.61
23.76
15.16
f1 (ppm)

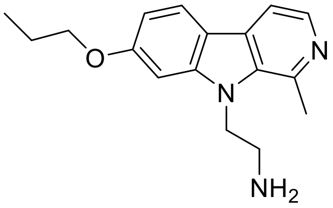

13

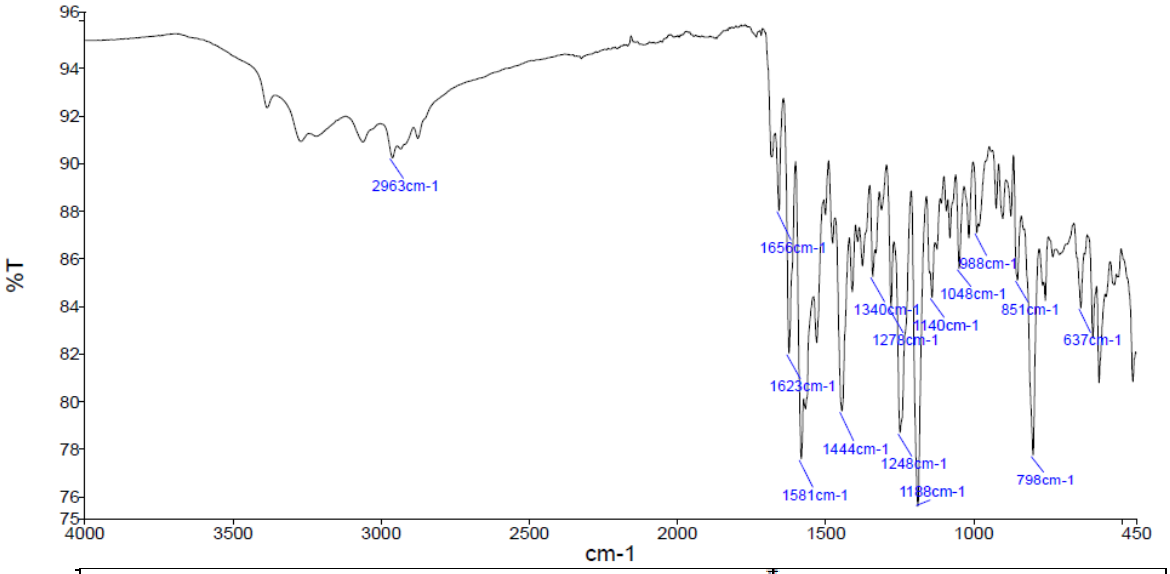
%T
cm-1
2963cm-1
1656cm-1
1623cm-1
1581cm-1
1444cm-1
1340cm-1
1278cm-1
1248cm-1
1188cm-1
1140cm-1
1048cm-1
988cm-1
851cm-1
798cm-1
637cm-1

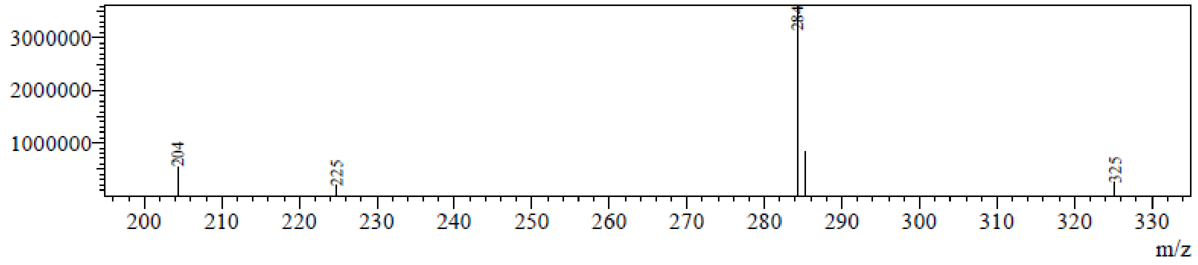
204
225
284
325
m/z

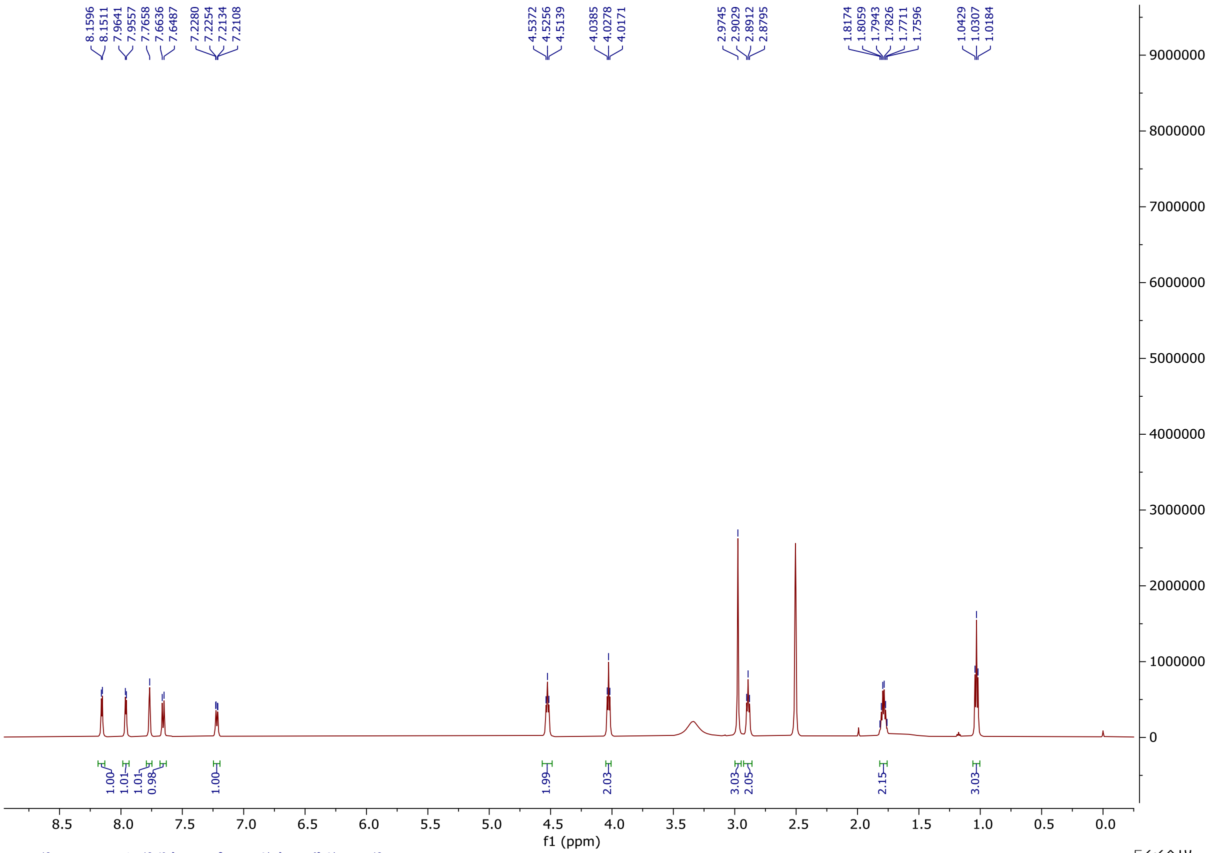

8.1596
8.1511
7.9641
7.9557
7.7658
7.6636
7.6487
7.2280
7.2254
7.2134
7.2108
4.5372
4.5256
4.5139
4.0385
4.0278
4.0171
2.9745
2.9029
2.8912
2.8795
1.8174
1.8059
1.7943
1.7826
1.7711
1.7596
1.0429
1.0307
1.0184
1.00
1.01
1.01
0.98
1.00
1.99
2.03
3.03
2.05
2.15
3.03
f1 (ppm)

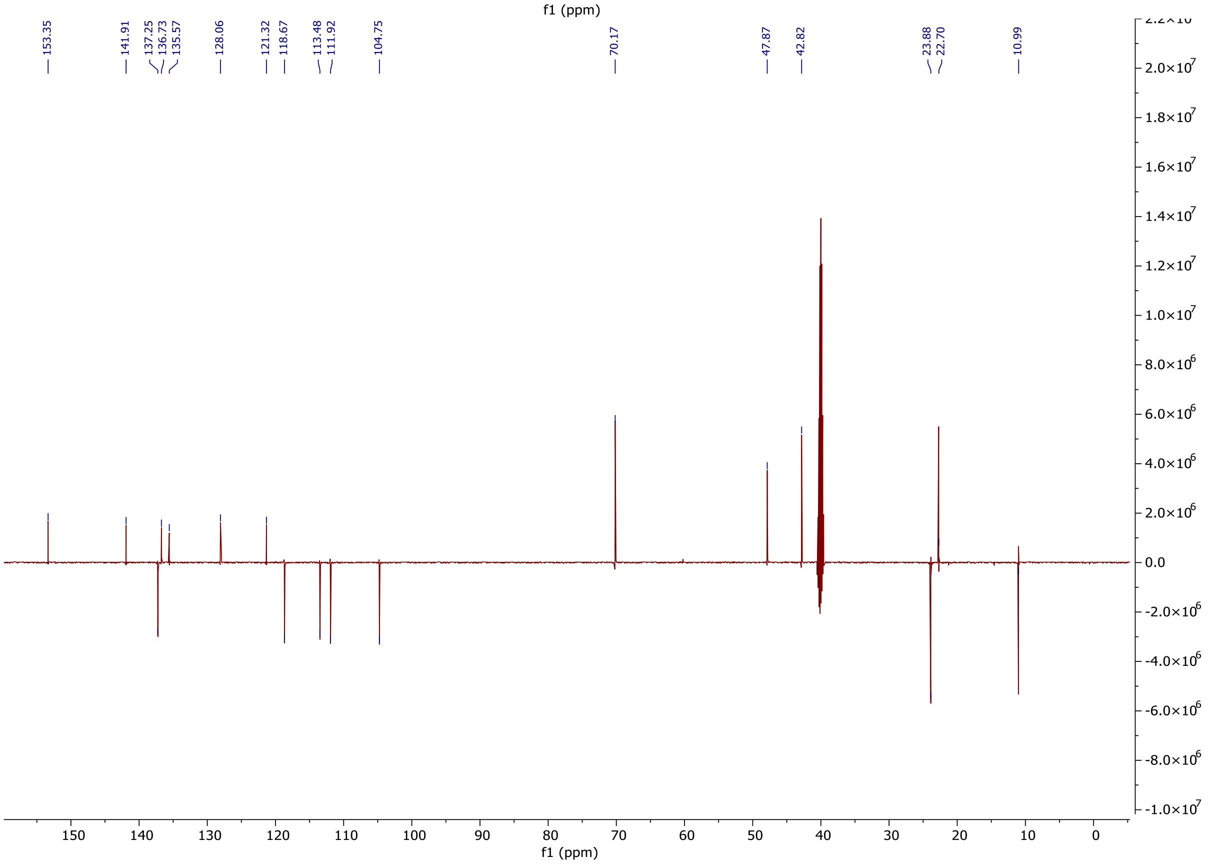

153.35
141.91
137.25
136.73
135.57
128.06
121.32
118.67
113.48
111.92
104.75
70.17
47.87
42.82
23.88
22.70
10.99
f1 (ppm)

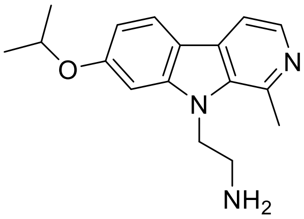

O
N
N
NH2

14

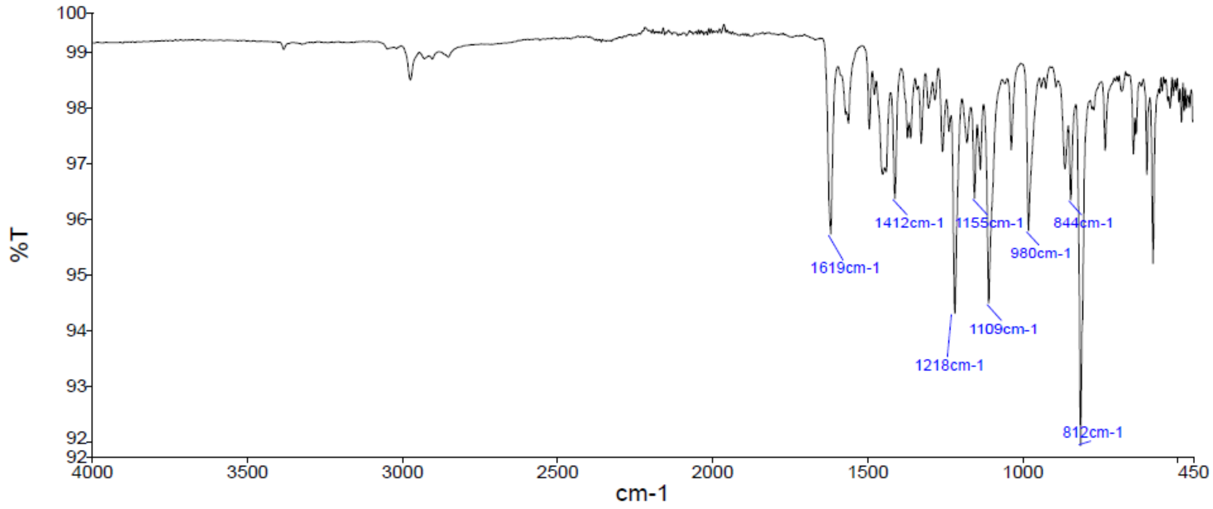

%T
cm-1
1619cm-1
1412cm-1
1218cm-1
1155cm-1
1109cm-1
980cm-1
844cm-1
812cm-1

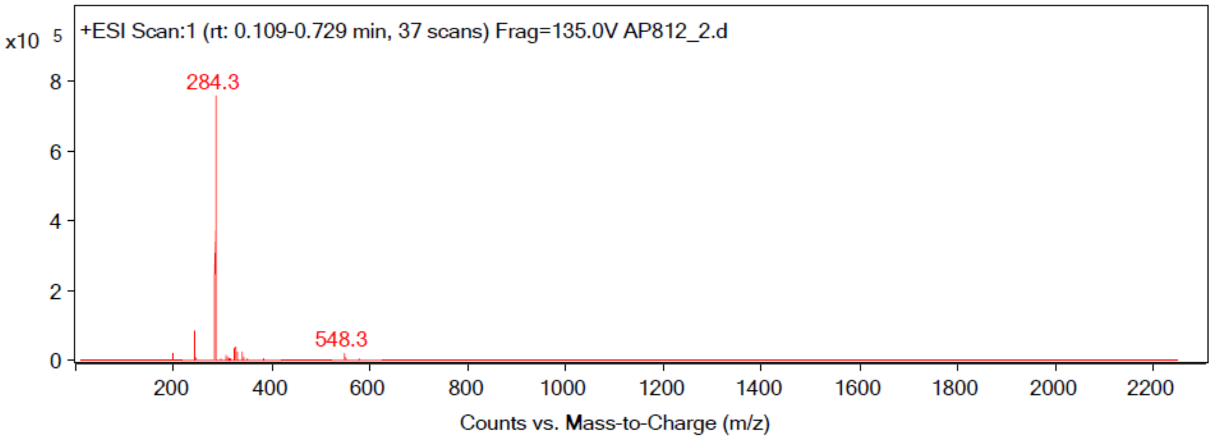

+ESI Scan:1 (rt: 0.109-0.729 min, 37 scans) Frag=135.0V AP812_2.d
x10 5
284.3
548.3
Counts vs. Mass-to-Charge (m/z)

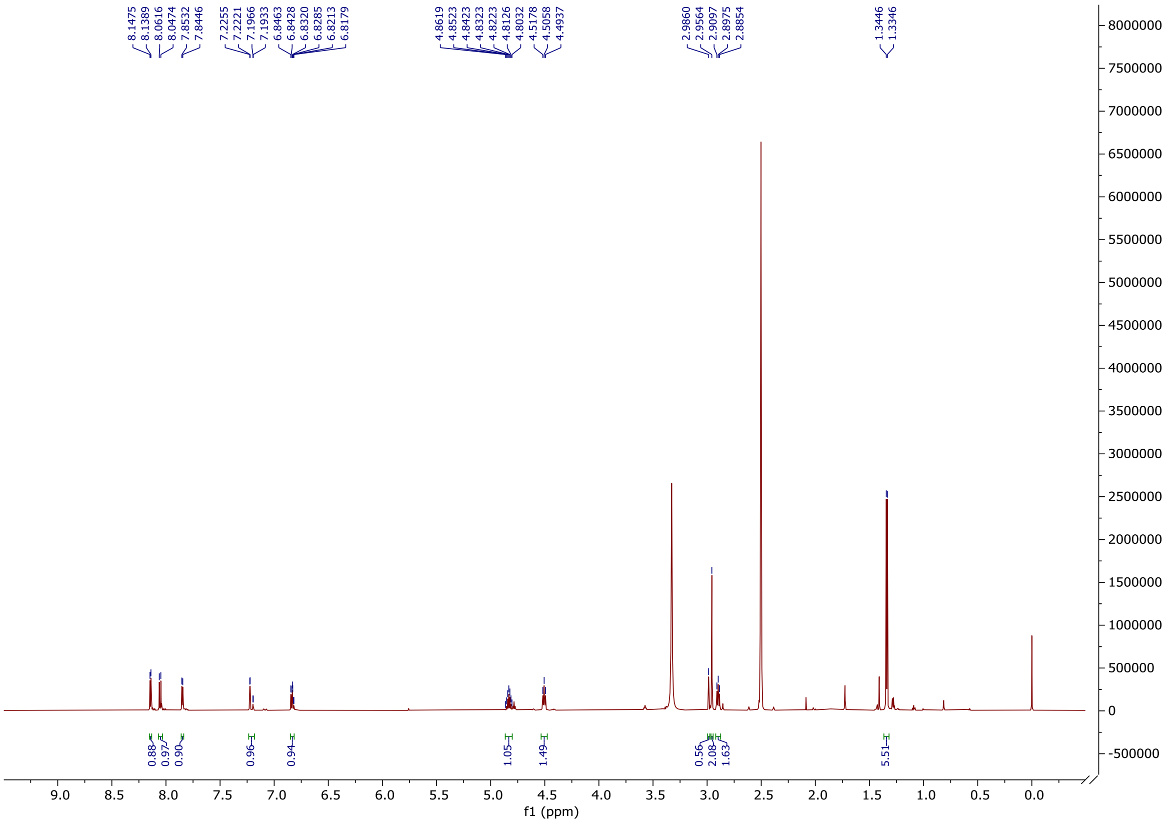

8.1475 8.1389 8.0616 8.0474 7.8532 7.8446
7.2255 7.2221 7.1966 7.1933 6.8463 6.8428 6.8320 6.8285 6.8213 6.8179
4.8619 4.8523 4.8423 4.8323 4.8223 4.8126 4.8032 4.5178 4.5058 4.4937
2.9860 2.9564 2.9097 2.8975 2.8854
1.3446 1.3346
0.88 0.97 0.90 0.96 0.94 1.05 1.49 0.56 2.08 1.63 5.51
f1 (ppm)

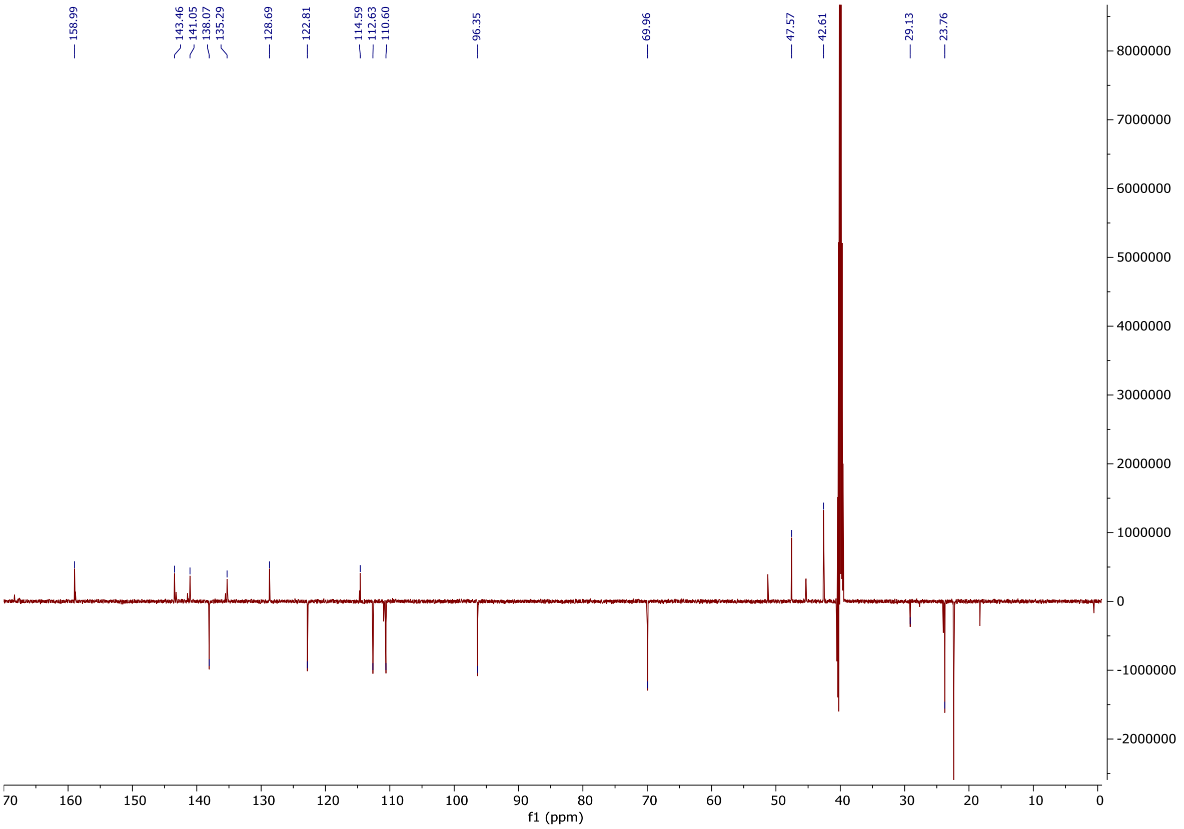
158.99
143.46
141.05
138.07
135.29
128.69
122.81
114.59
112.63
110.60
96.35
69.96
47.57
42.61
29.13
23.76
8000000
7000000
6000000
5000000
4000000
3000000
2000000
1000000
0
-1000000
-2000000
f1 (ppm)

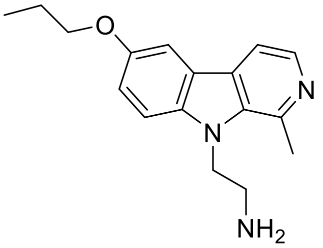
O
N
N
NH2

22

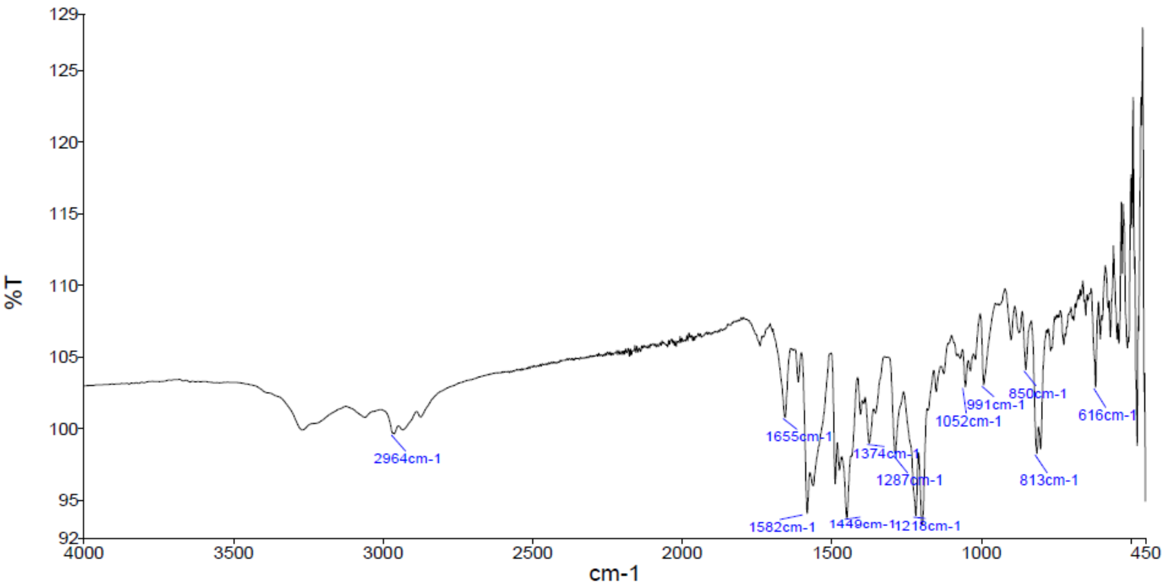
%T
cm-1
2964cm-1
1655cm-1
1582cm-1
1449cm-1
1374cm-1
1287cm-1
1218cm-1
1052cm-1
991cm-1
850cm-1
813cm-1
616cm-1

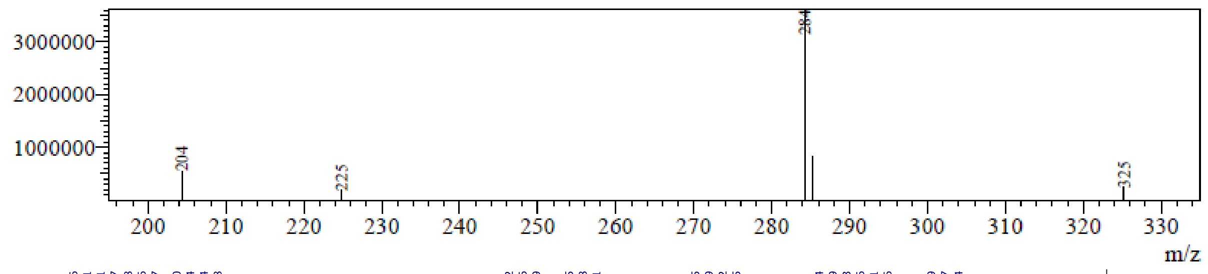

3000000
2000000
1000000
204
225
284
325
200 210 220 230 240 250 260 270 280 290 300 310 320 330
m/z

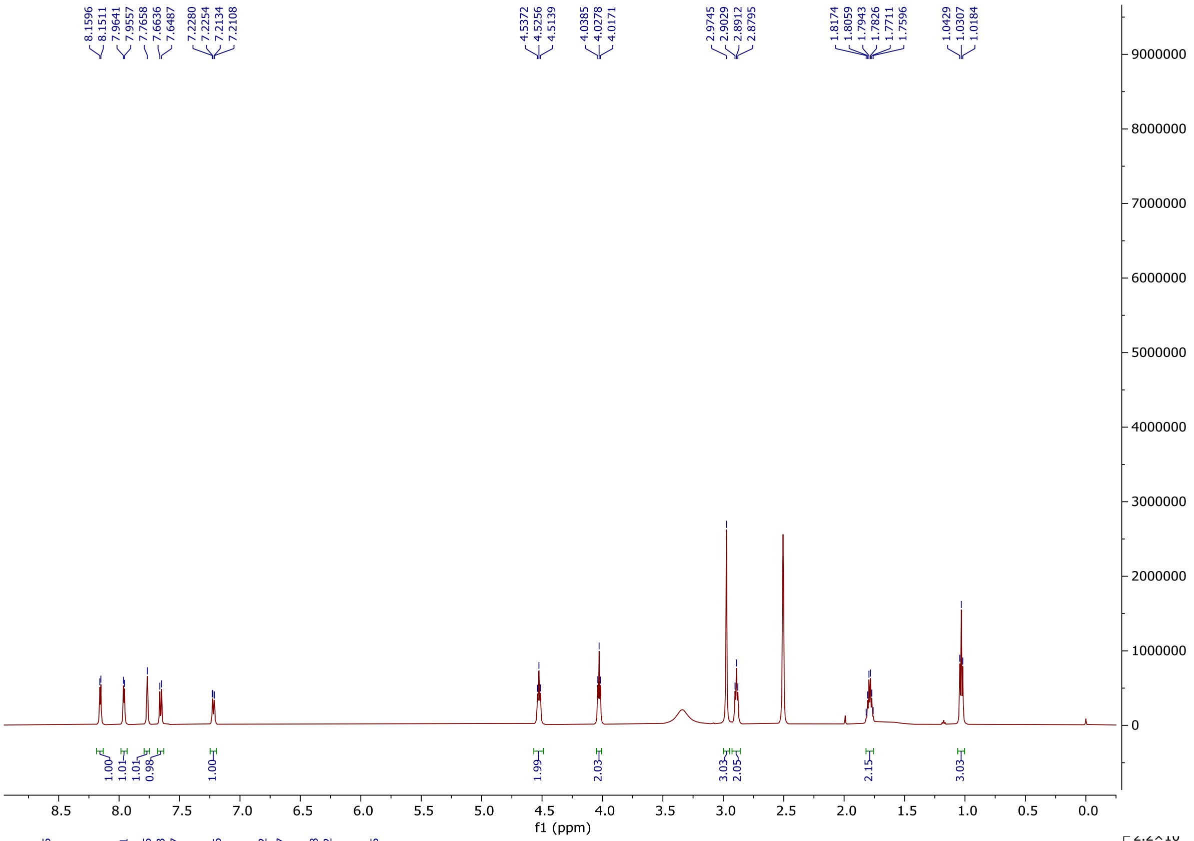

8.1596 8.1511 7.9641 7.9557 7.7658 7.6636 7.6487 7.2280 7.2254 7.2134 7.2108
4.5372 4.5256 4.5139
4.0385 4.0278 4.0171
2.9745 2.9029 2.8912 2.8795
1.8174 1.8059 1.7943 1.7826 1.7711 1.7596
1.0429 1.0307 1.0184
1.00 1.01 1.01 0.98 1.00 1.99 2.03 3.03 2.05 2.15 3.03
f1 (ppm)

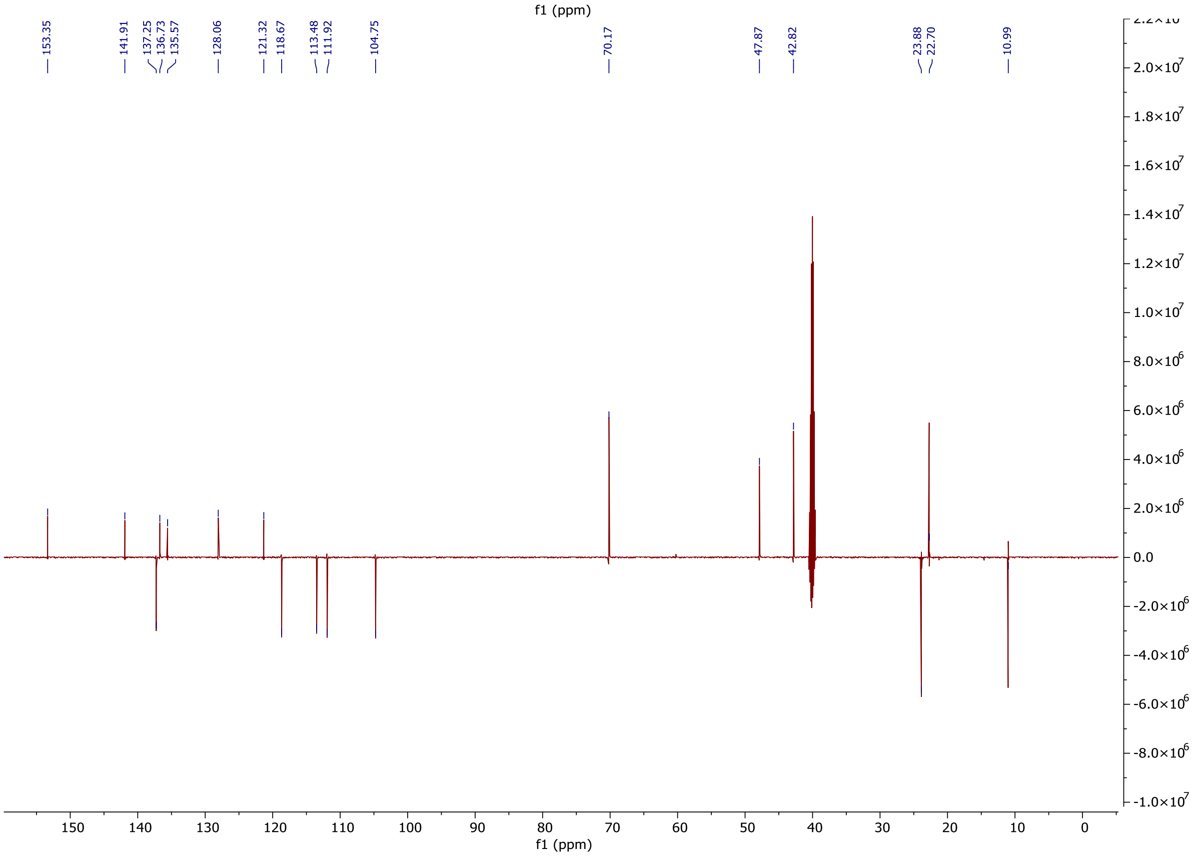

153.35
141.91
137.25 136.73 135.57
128.06
121.32
118.67
113.48 111.92
104.75
70.17
47.87
42.82
23.88 22.70
10.99
f1 (ppm)

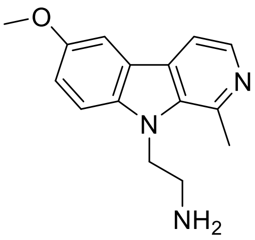

24

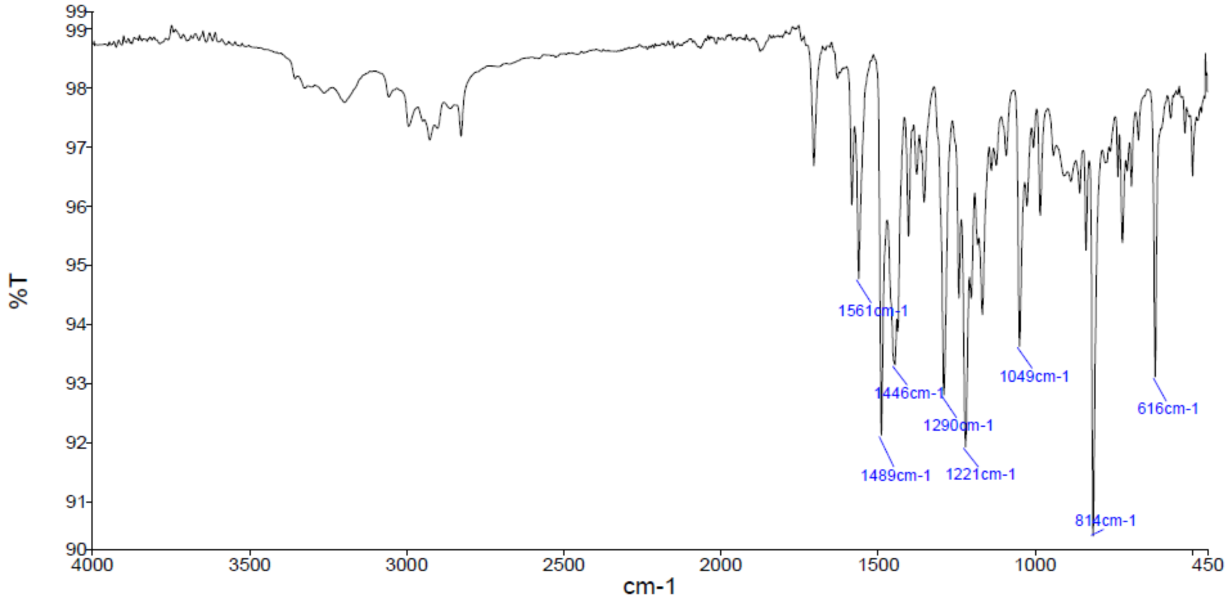

%T
cm-1
1561cm-1
1446cm-1
1290cm-1
1489cm-1
1221cm-1
1049cm-1
814cm-1
616cm-1

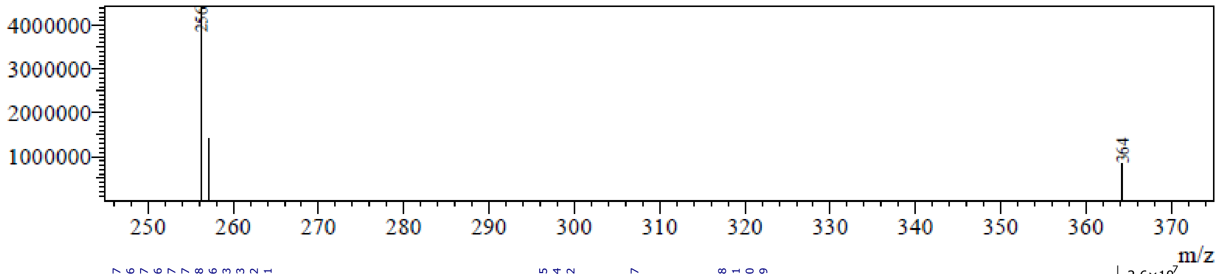

256
364
m/z

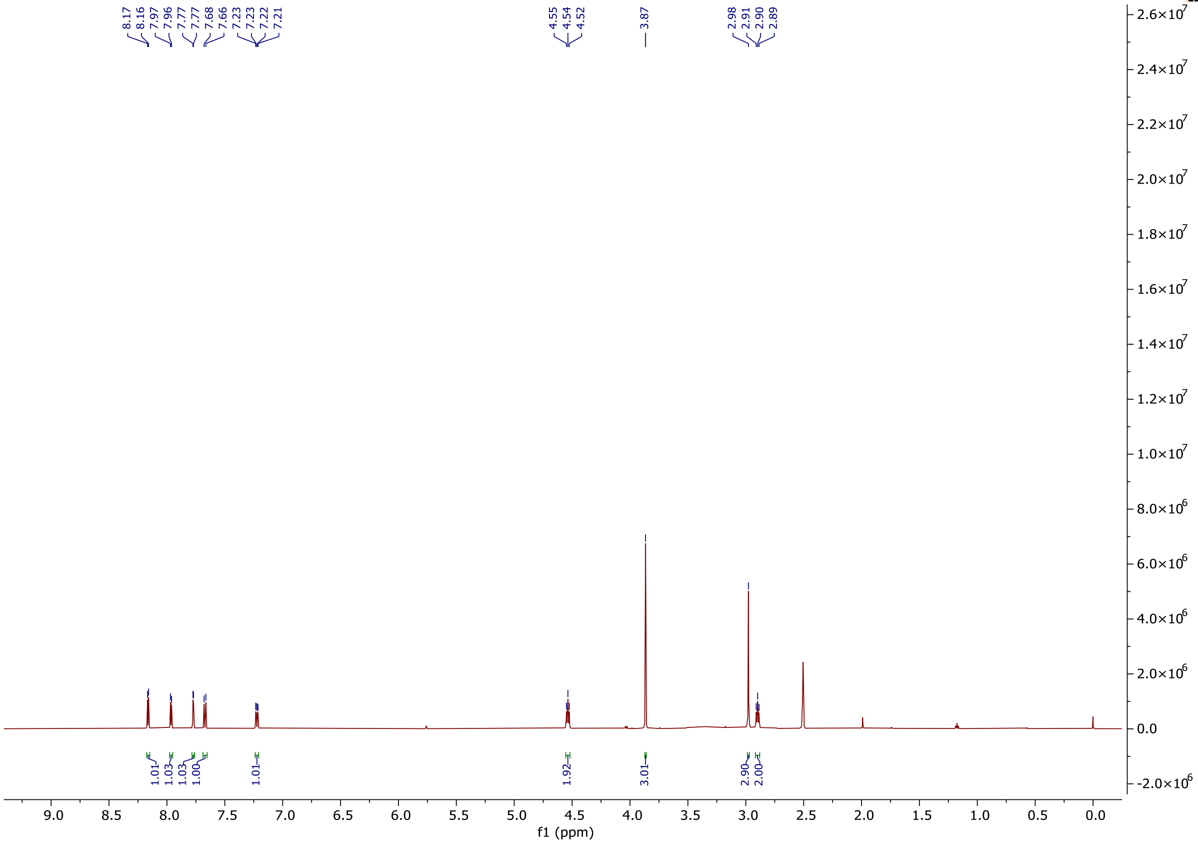

f1 (ppm)

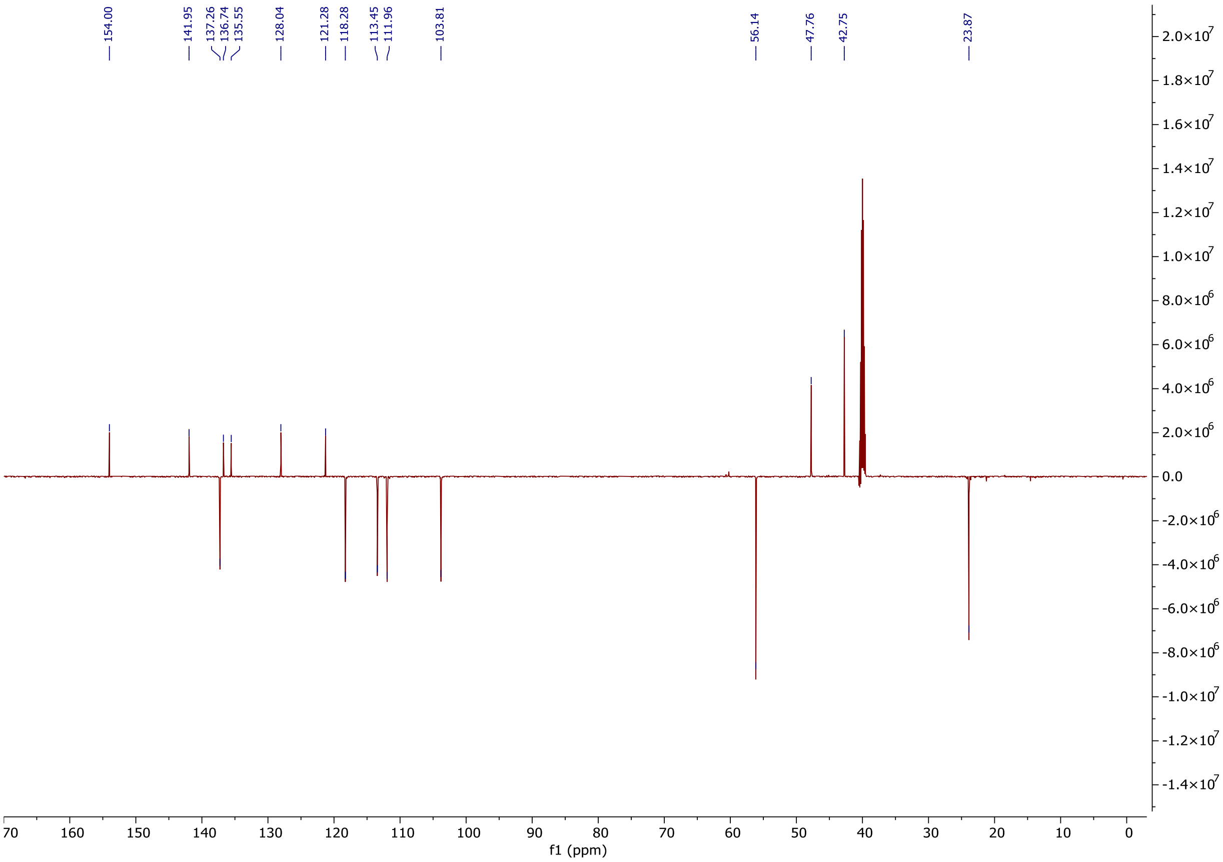
154.00
141.95
137.26
136.74
135.55
128.04
121.28
118.28
113.45
111.96
103.81
56.14
47.76
42.75
23.87
f1 (ppm)

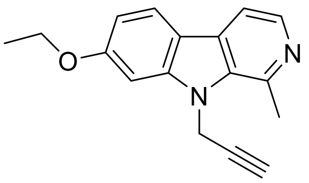
O
N
N

15

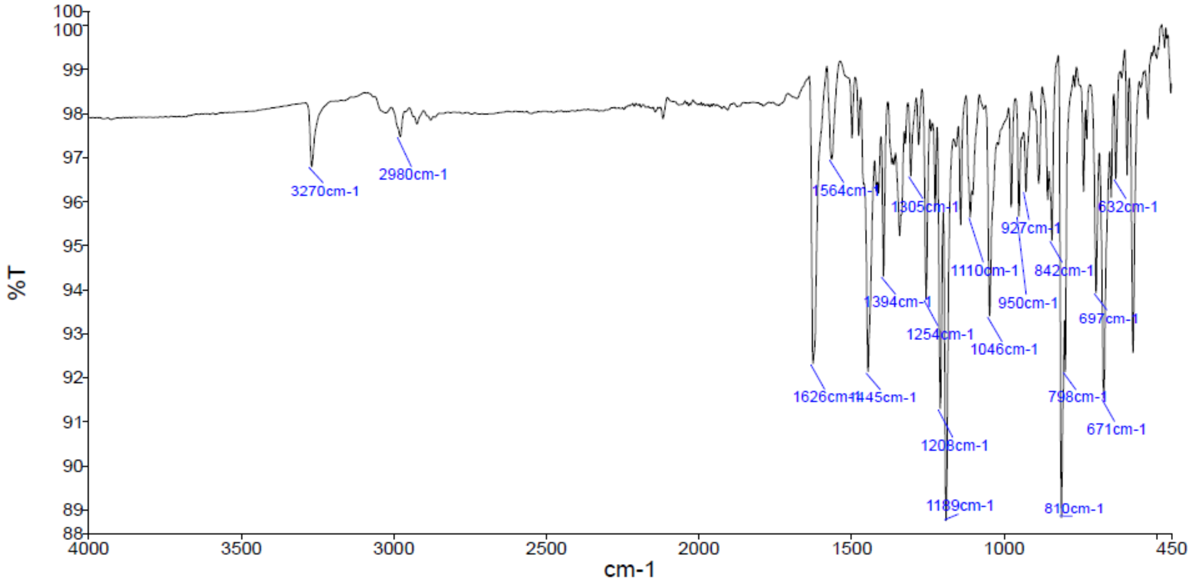
%T
cm-1
3270cm-1
2980cm-1
1626cm-1
1564cm-1
1445cm-1
1394cm-1
1305cm-1
1254cm-1
1208cm-1
1189cm-1
1110cm-1
1046cm-1
950cm-1
927cm-1
842cm-1
810cm-1
798cm-1
697cm-1
671cm-1
632cm-1

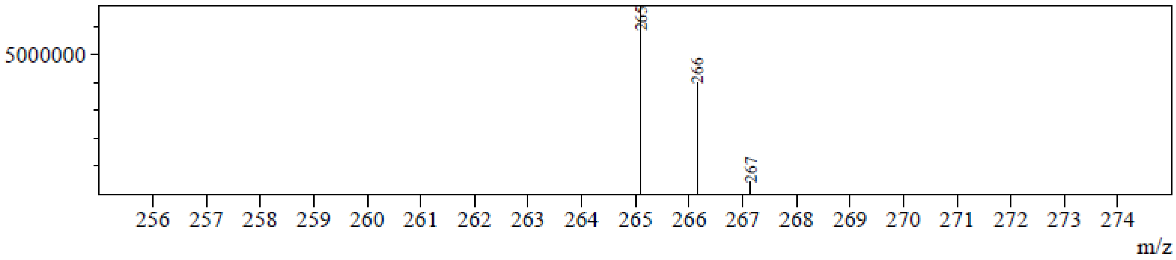
5000000
266
267
m/z

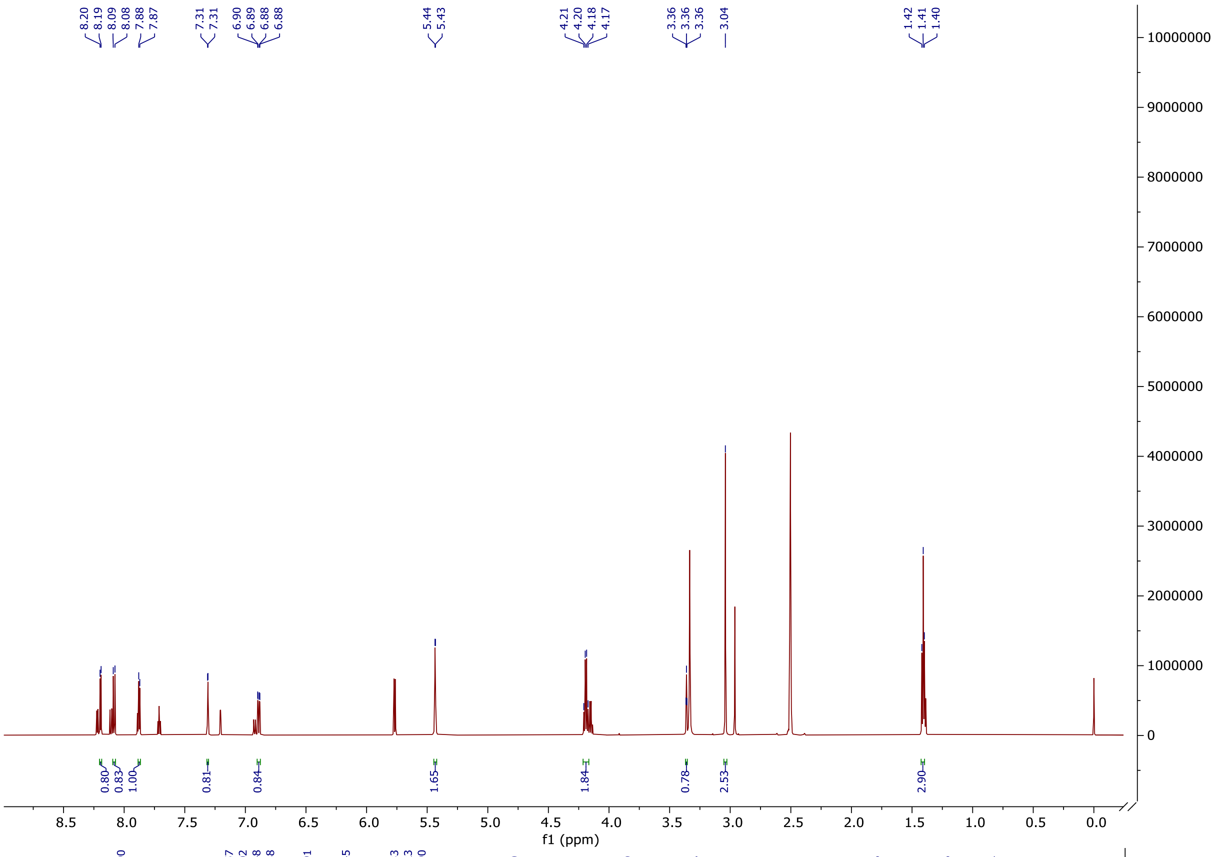
8.20
8.19
8.09
8.08
7.88
7.87
7.31
7.31
6.90
6.89
6.88
6.88
5.44
5.43
4.21
4.20
4.18
4.17
3.36
3.36
3.36
3.04
1.42
1.41
1.40
0.80
0.83
1.00
0.81
0.84
1.65
1.84
0.78
2.53
2.90
f1 (ppm)

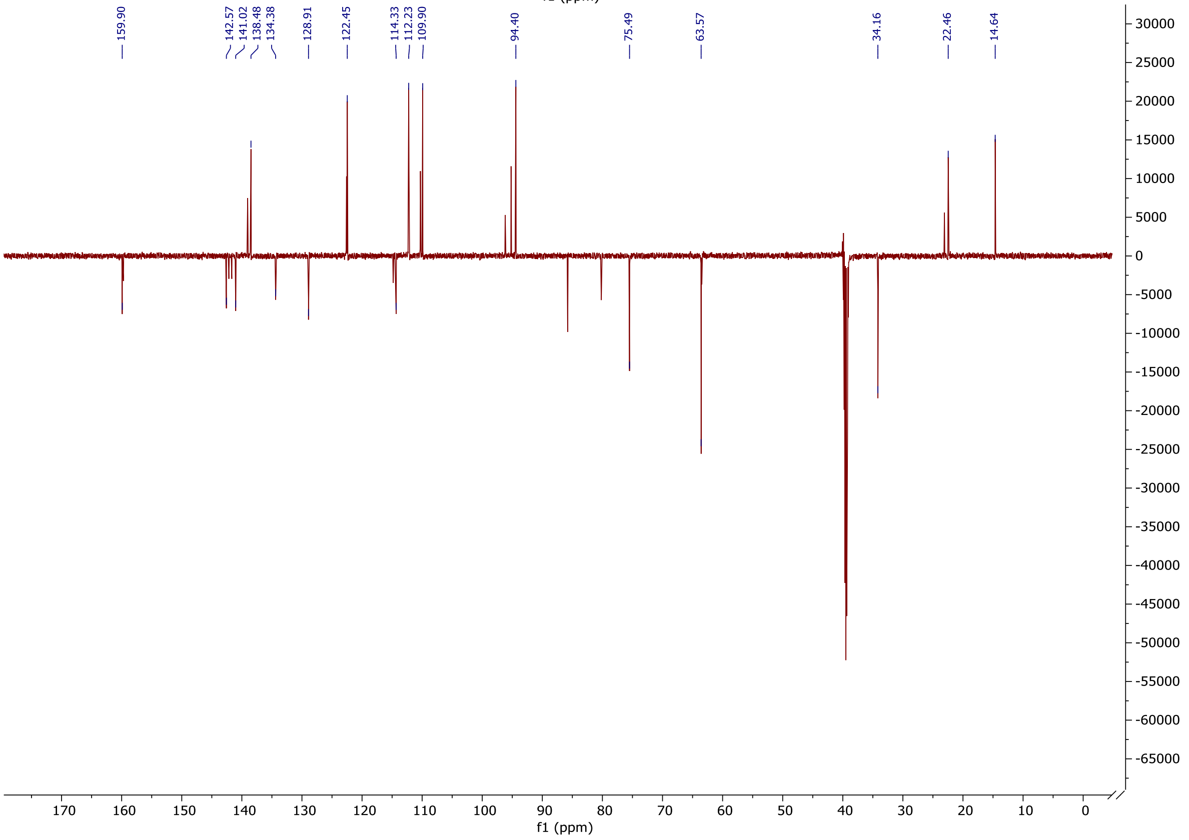
159.90
142.57
141.02
138.48
134.38
128.91
122.45
114.33
112.23
109.90
94.40
75.49
63.57
34.16
22.46
14.64
f1 (ppm)

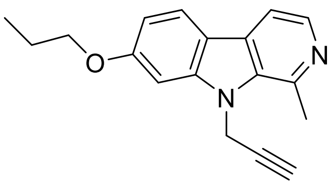

16

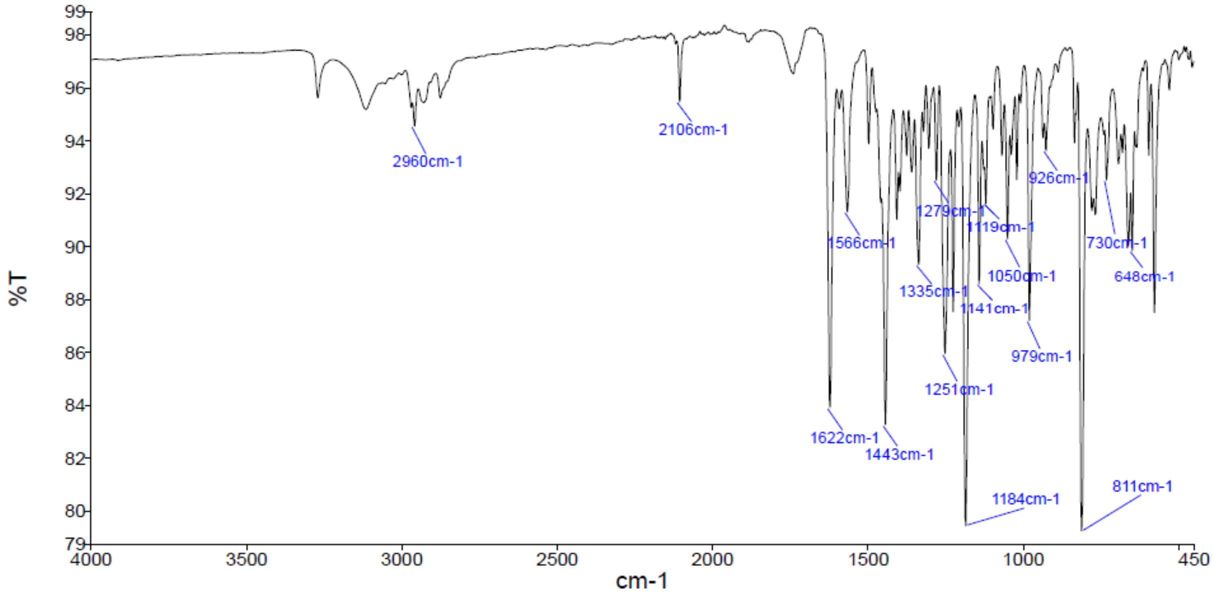
%T
cm-1
2960cm-1
2106cm-1
1622cm-1
1566cm-1
1443cm-1
1335cm-1
1279cm-1
1251cm-1
1184cm-1
1141cm-1
1119cm-1
1050cm-1
979cm-1
926cm-1
811cm-1
730cm-1
648cm-1

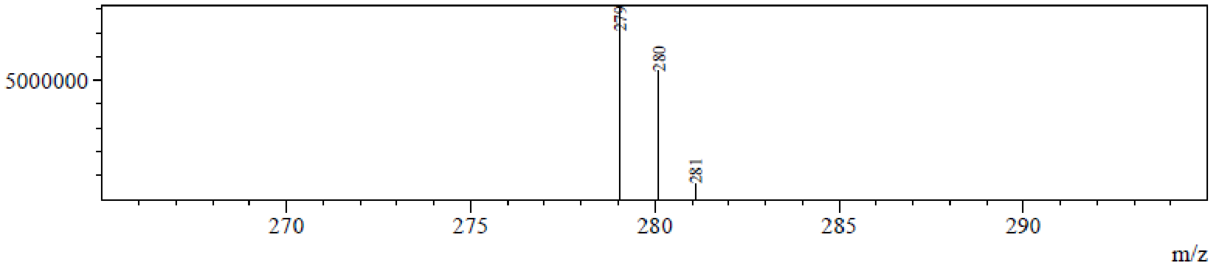
279
280
281
m/z

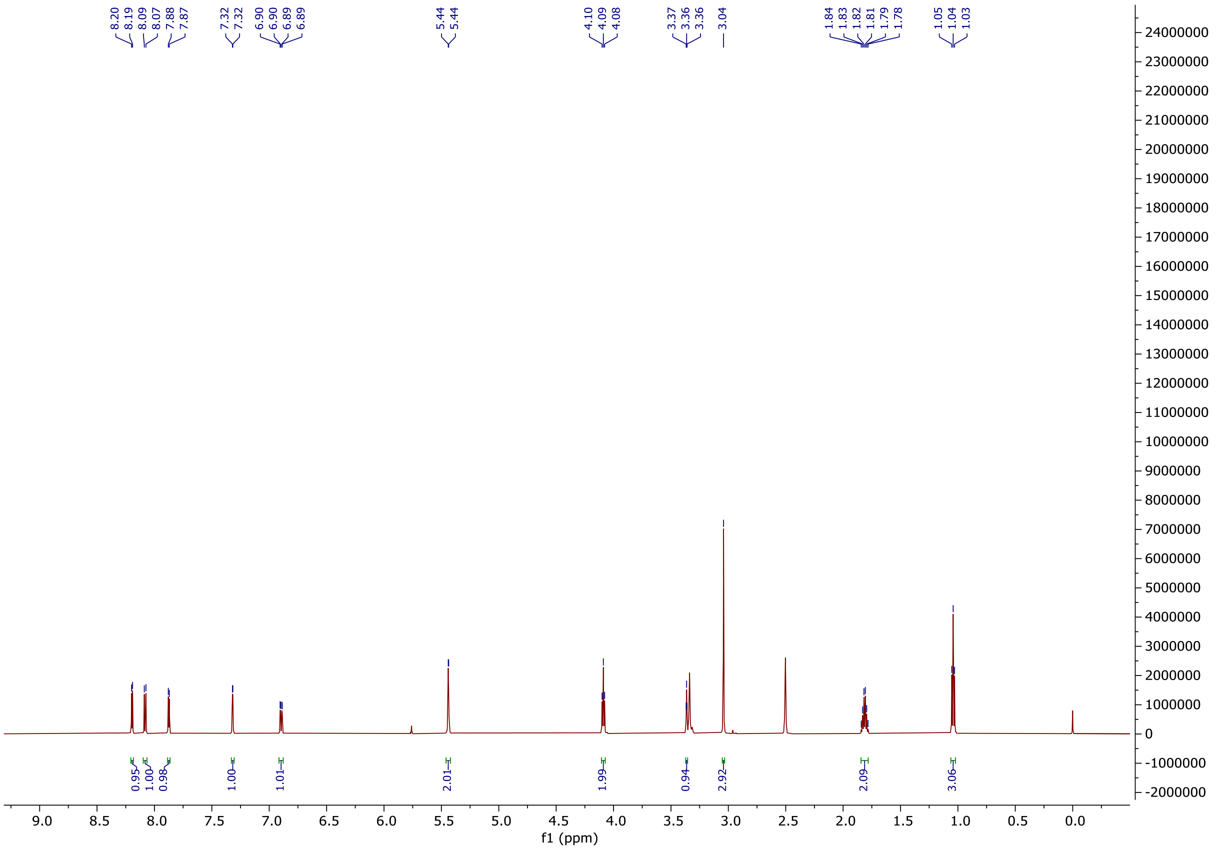
f1 (ppm)

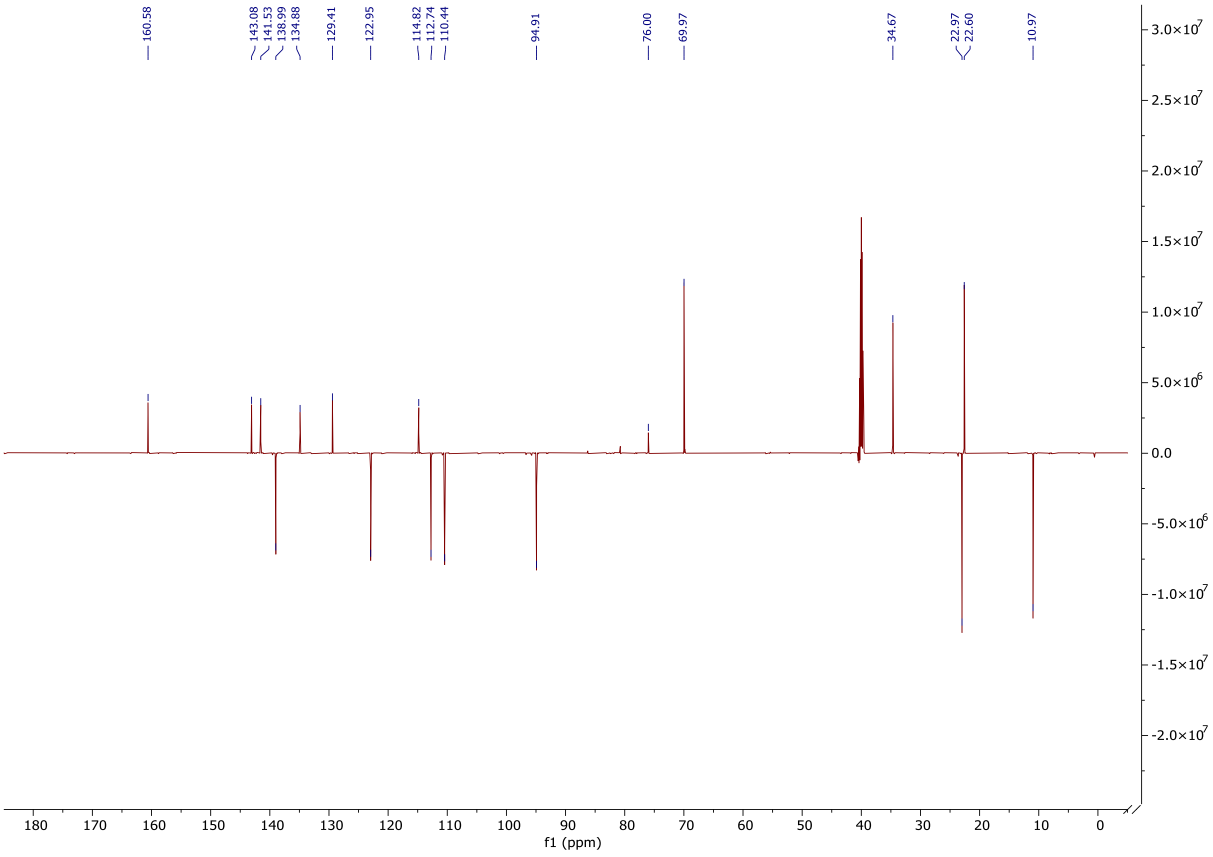
160.58
143.08
141.53
138.99
134.88
129.41
122.95
114.82
112.74
110.44
94.91
76.00
69.97
34.67
22.97
22.60
10.97
f1 (ppm)

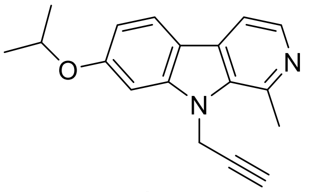
O
N
N

17

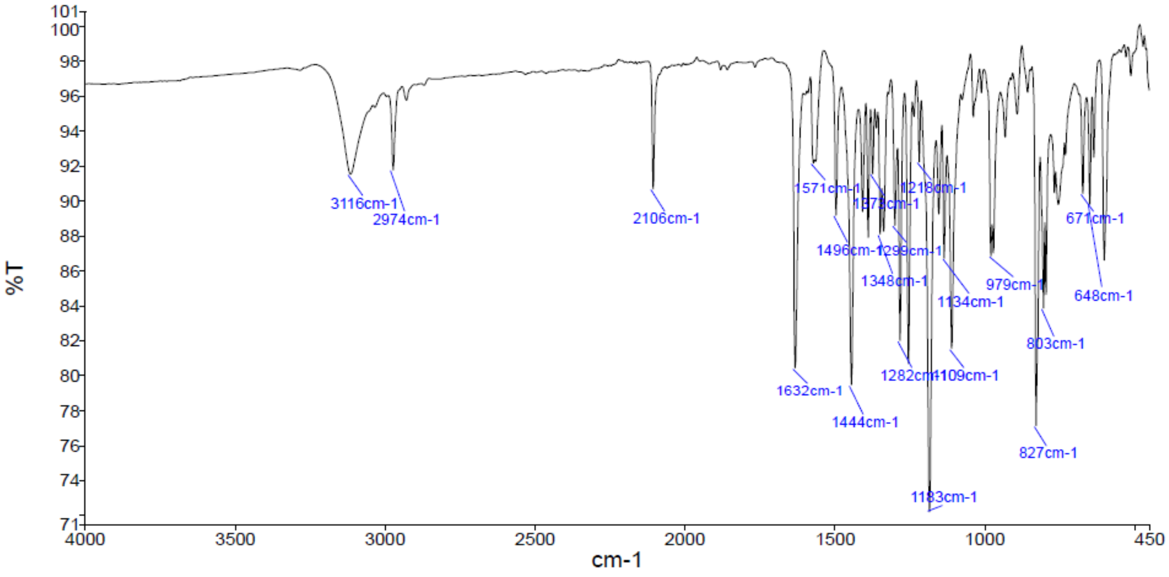
%T
cm-1
3116cm-1
2974cm-1
2106cm-1
1632cm-1
1571cm-1
1496cm-1
1444cm-1
1348cm-1
1299cm-1
1282cm-1
1218cm-1
1183cm-1
1134cm-1
1109cm-1
979cm-1
827cm-1
803cm-1
671cm-1
648cm-1

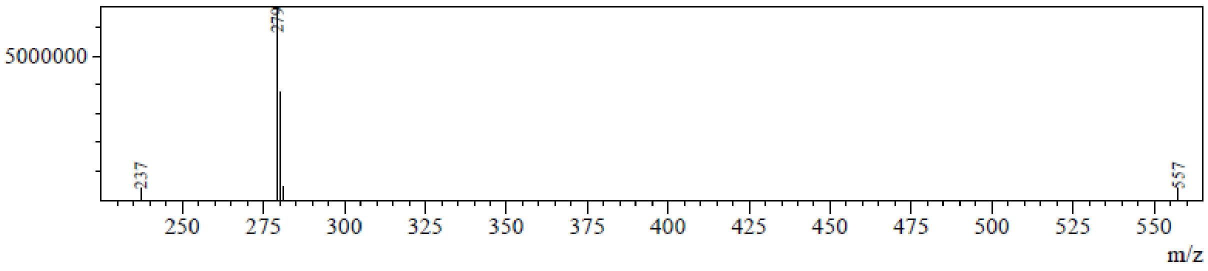
279
237
557
m/z

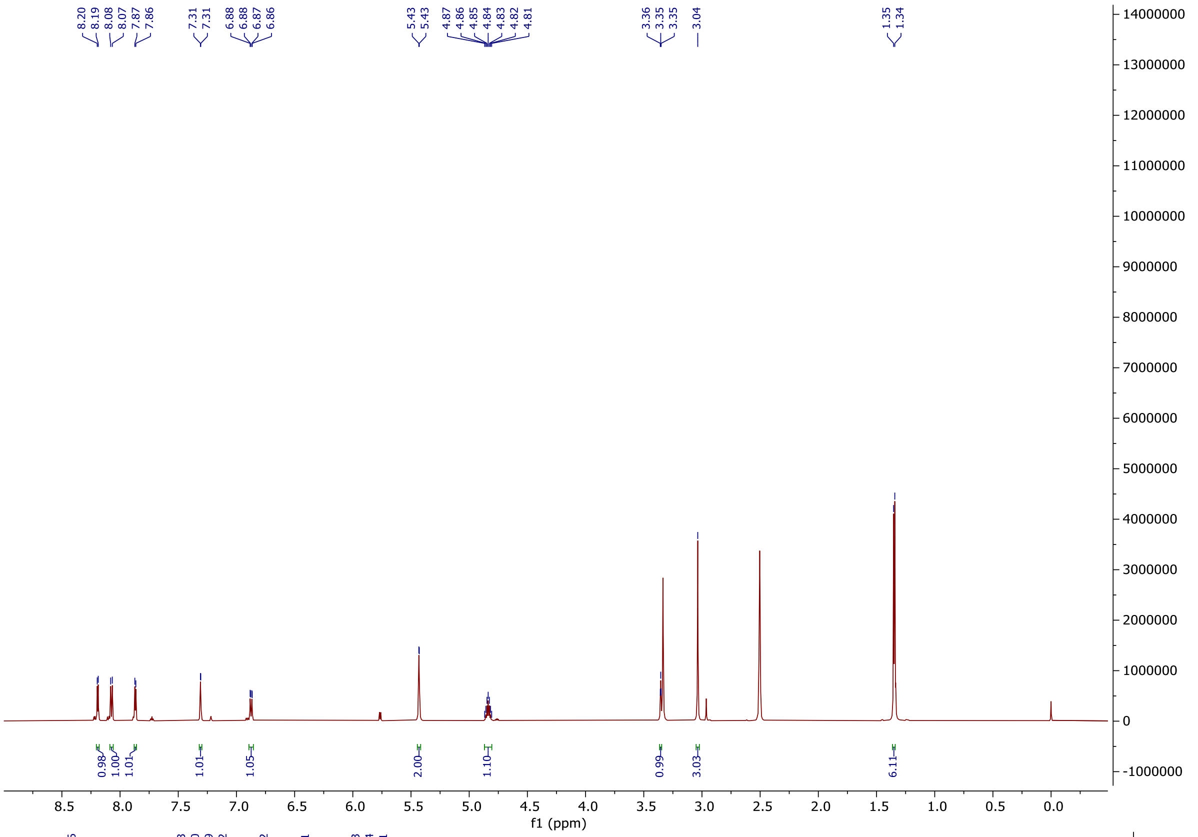
8.20
8.19
8.08
8.07
7.87
7.86
7.31
7.31
6.88
6.88
6.87
6.86
5.43
5.43
4.87
4.86
4.85
4.84
4.83
4.82
4.81
3.36
3.35
3.35
3.04
1.35
1.34
0.98
1.00
1.01
1.01
1.05
2.00
1.10
0.99
3.03
6.11
f1 (ppm)

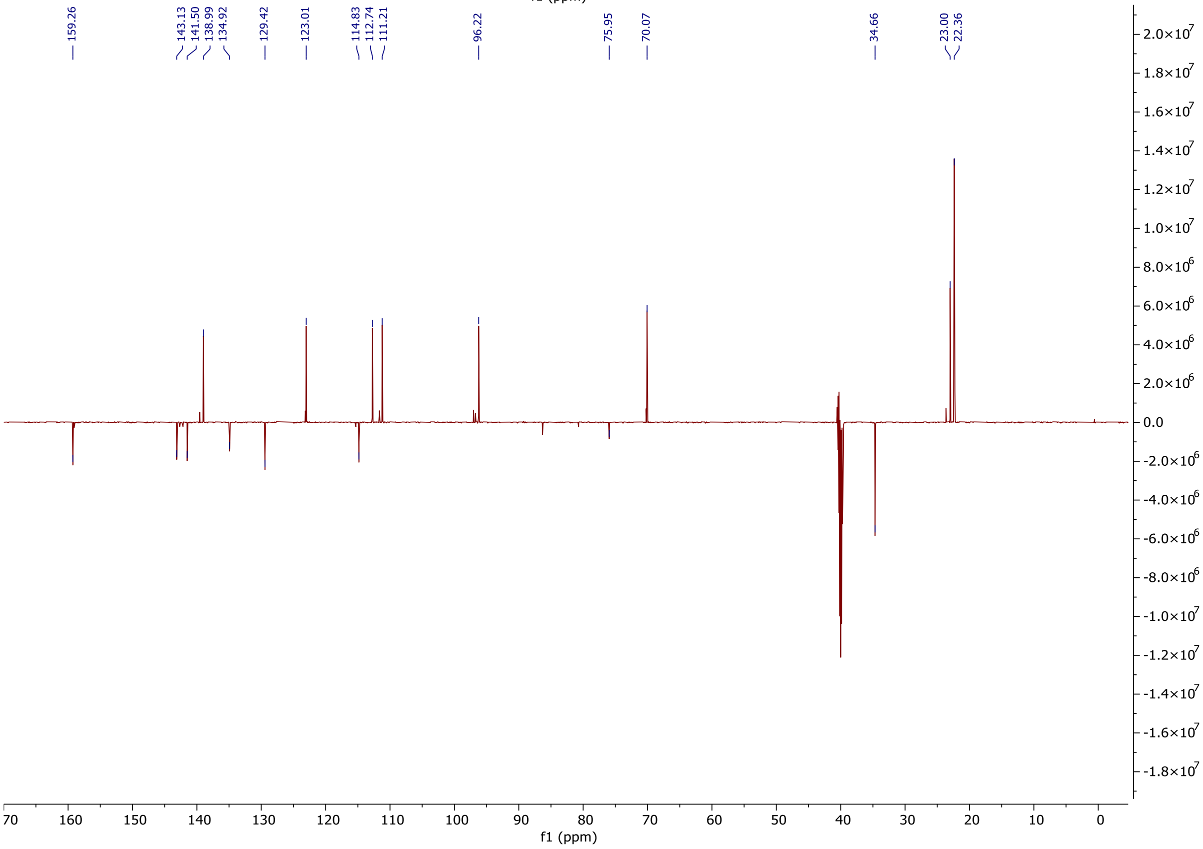
159.26
143.13
141.50
138.99
134.92
129.42
123.01
114.83
112.74
111.21
96.22
75.95
70.07
34.66
23.00
22.36
f1 (ppm)

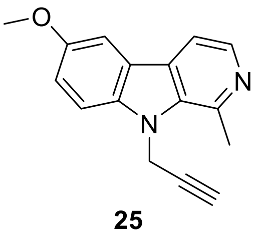

25

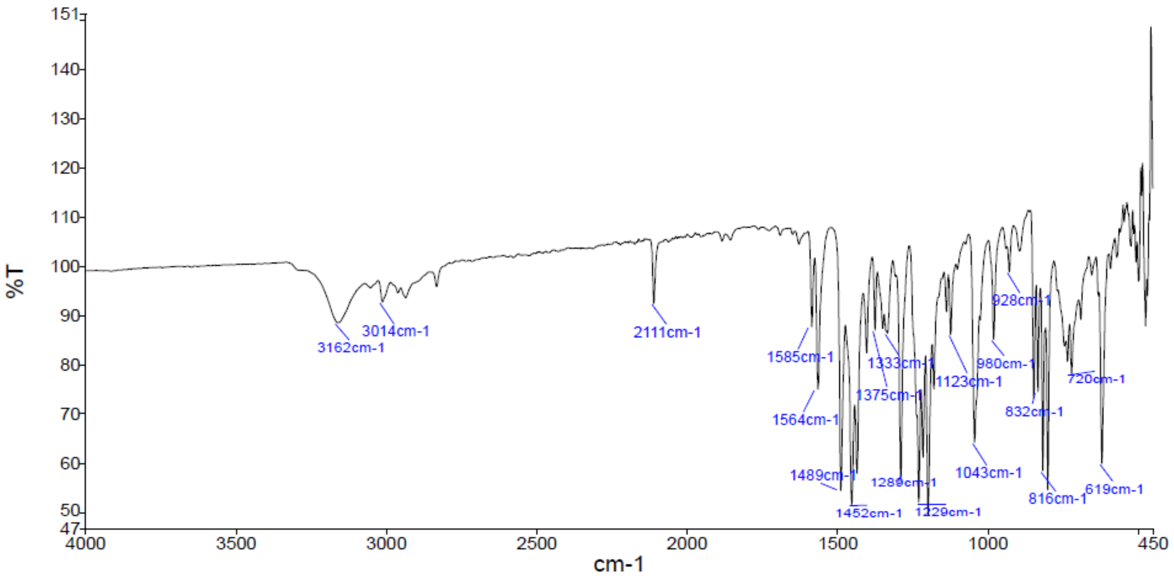
%T
cm-1
3162cm-1
3014cm-1
2111cm-1
1585cm-1
1564cm-1
1489cm-1
1452cm-1
1375cm-1
1333cm-1
1289cm-1
1229cm-1
1123cm-1
1043cm-1
980cm-1
928cm-1
832cm-1
816cm-1
720cm-1
619cm-1

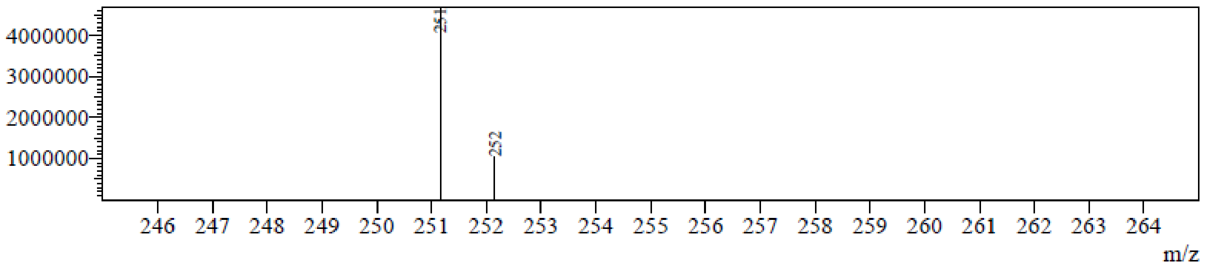
251
252
m/z

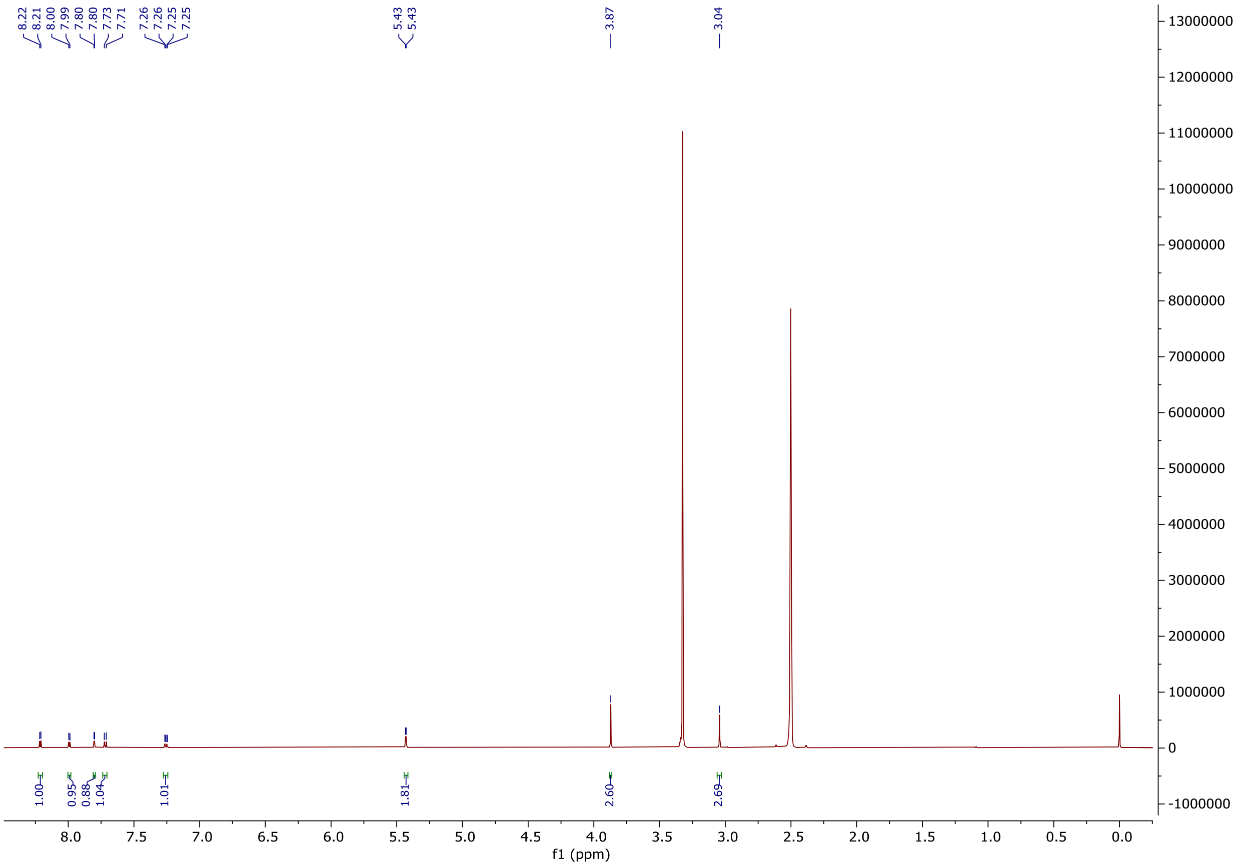
8.22
8.21
8.00
7.99
7.80
7.80
7.73
7.71
7.26
7.26
7.25
7.25
5.43
5.43
3.87
3.04
1.00
0.95
0.88
1.04
1.01
1.81
2.60
2.69
f1 (ppm)

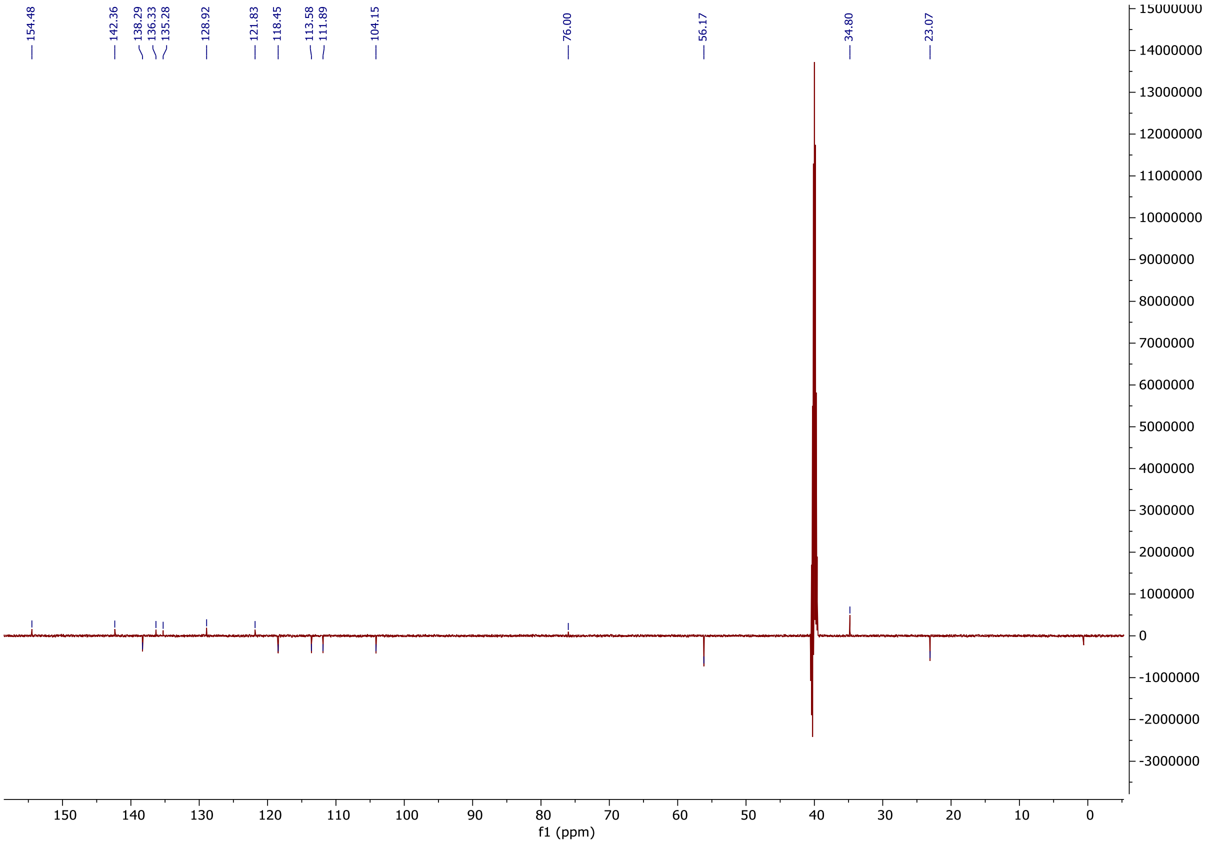
154.48
142.36
138.29
136.33
135.28
128.92
121.83
118.45
113.58
111.89
104.15
76.00
56.17
34.80
23.07
f1 (ppm)

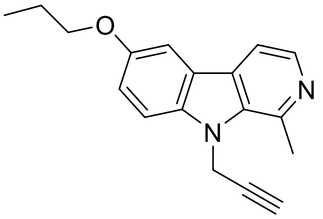

26

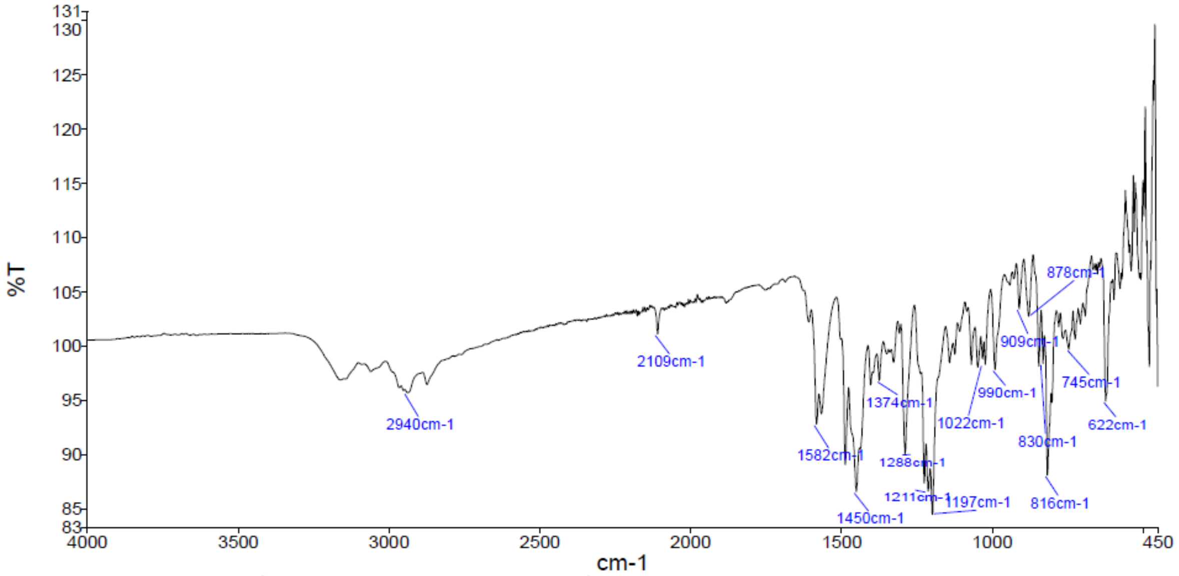
%T
cm-1
2940cm-1
2109cm-1
1582cm-1
1450cm-1
1374cm-1
1288cm-1
1211cm-1
1197cm-1
1022cm-1
990cm-1
909cm-1
878cm-1
830cm-1
816cm-1
745cm-1
622cm-1

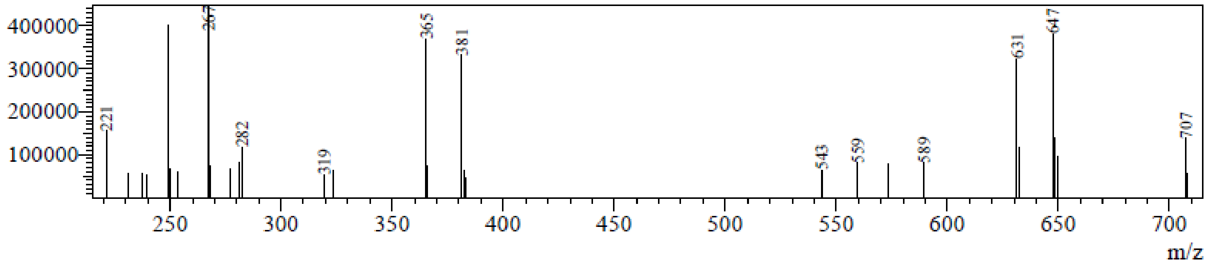
221
267
282
319
365
381
543
559
589
631
647
707
m/z

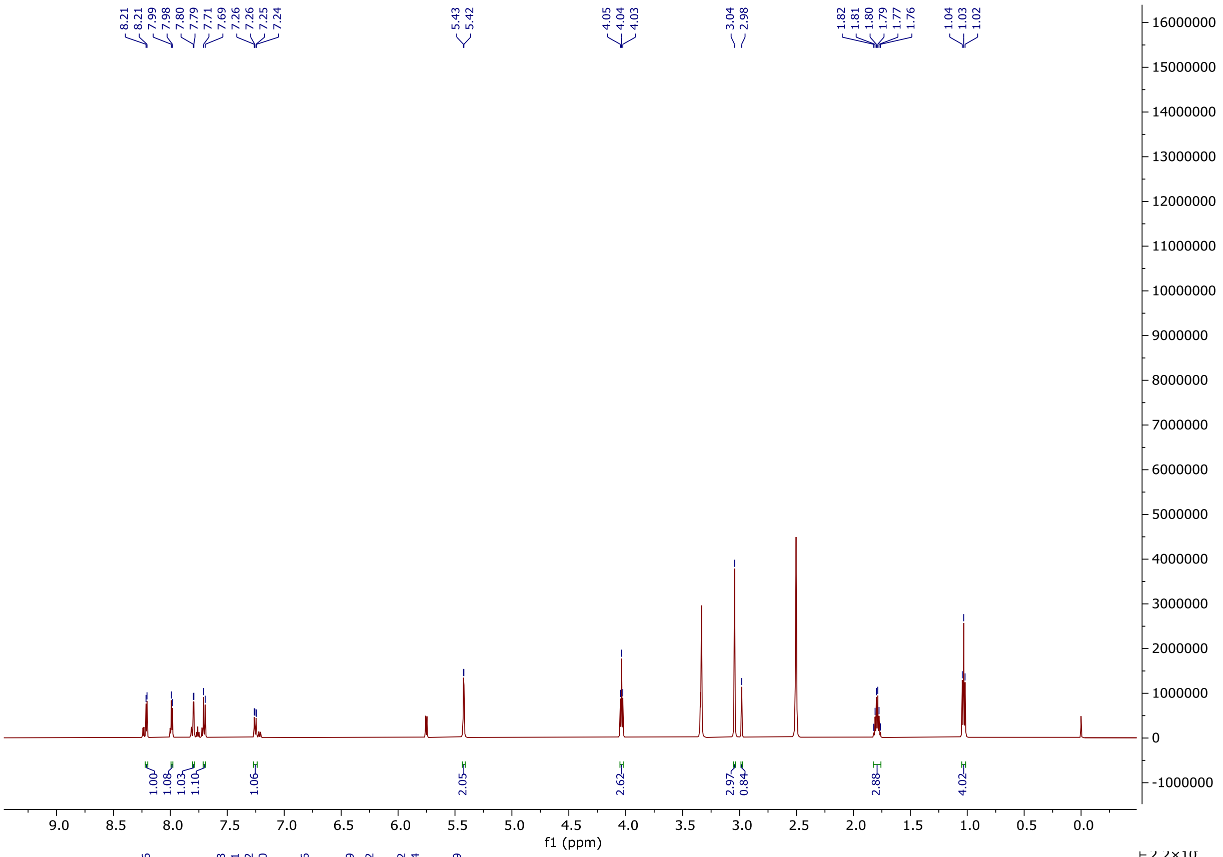
8.21
8.21
7.99
7.98
7.80
7.79
7.71
7.69
7.26
7.26
7.25
7.24
5.43
5.42
4.05
4.04
4.03
3.04
2.98
1.82
1.81
1.80
1.79
1.77
1.76
1.04
1.03
1.02
1.00
1.08
1.03
1.10
1.06
2.05
2.62
2.97
0.84
2.88
4.02
f1 (ppm)

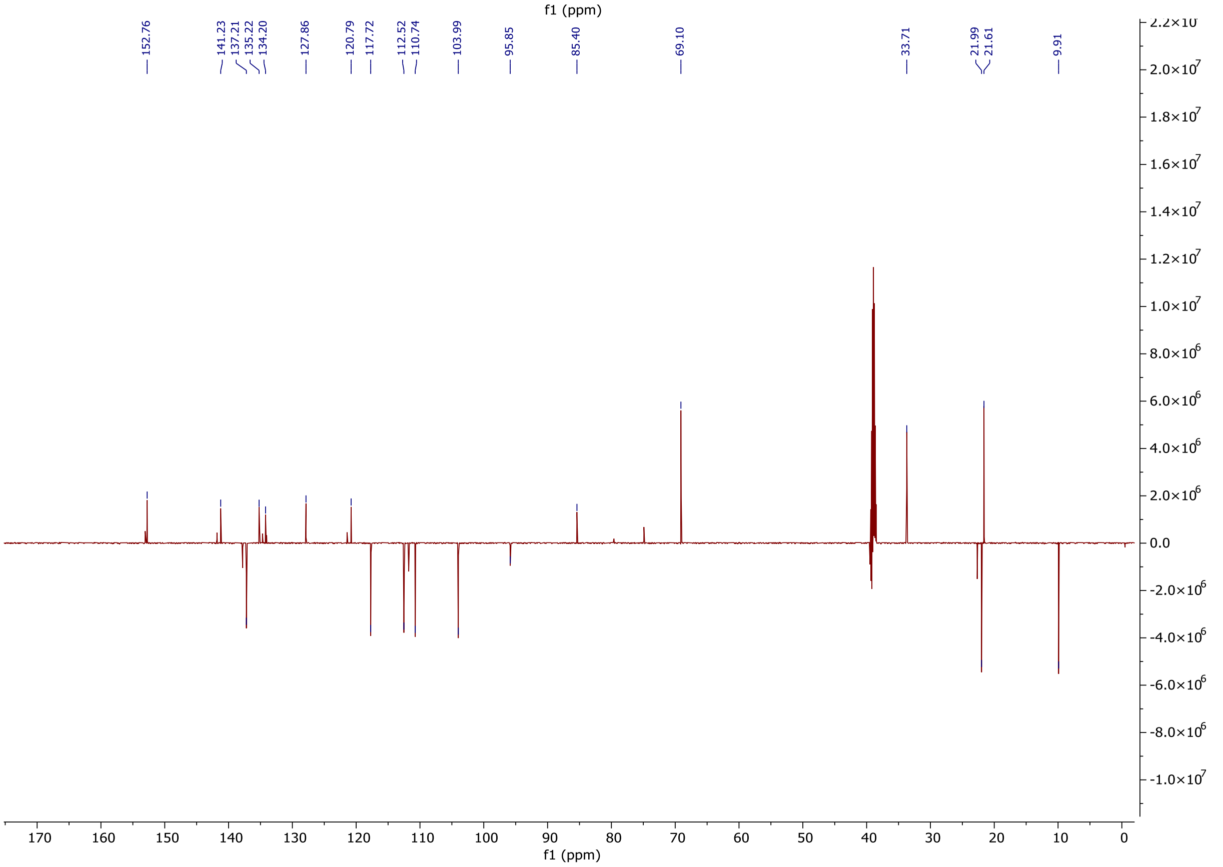
152.76
141.23
137.21
135.22
134.20
127.86
120.79
117.72
112.52
110.74
103.99
95.85
85.40
69.10
33.71
21.99
21.61
9.91
f1 (ppm)

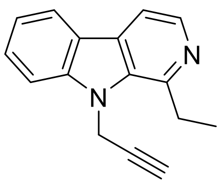

30

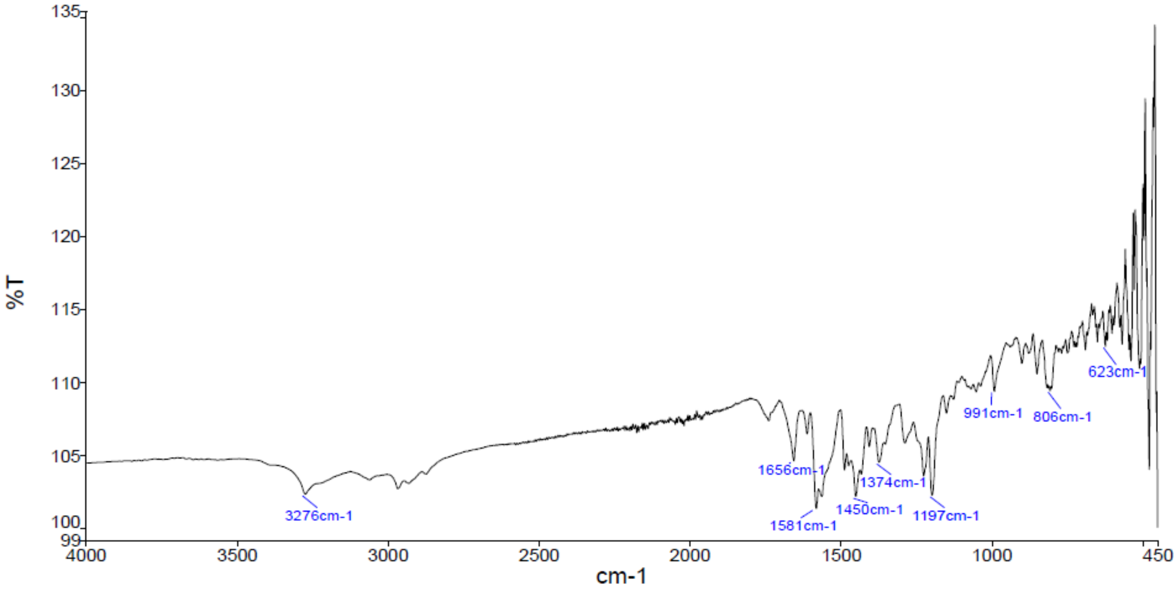
%T
cm-1
3276cm-1
1656cm-1
1581cm-1
1450cm-1
1374cm-1
1197cm-1
991cm-1
806cm-1
623cm-1

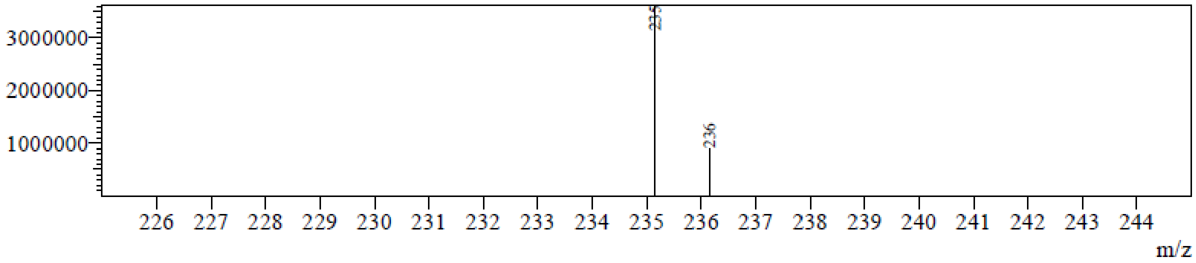
235
236
m/z

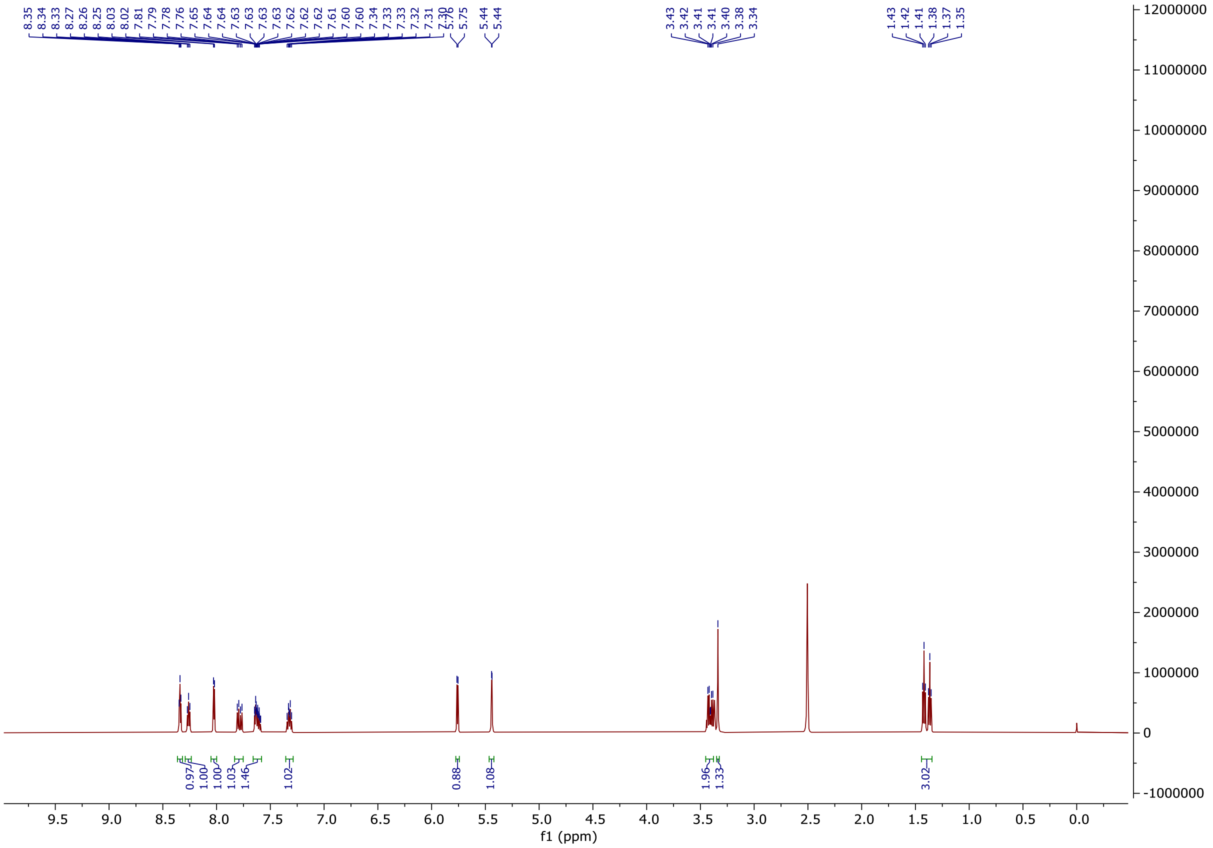
8.35 8.34 8.33 8.27 8.26 8.25 8.03 8.02 7.81 7.79 7.78 7.76 7.65 7.64 7.64 7.63 7.63 7.63 7.62 7.62 7.62 7.61 7.60 7.34 7.33 7.33 7.32 7.31 7.30 5.76 5.75 5.44 5.44
3.43 3.42 3.41 3.41 3.40 3.38 3.34
1.43 1.42 1.41 1.38 1.37 1.35
0.97 1.00 1.00 1.03 1.46 1.02 0.88 1.08 1.96 1.33 3.02
f1 (ppm)

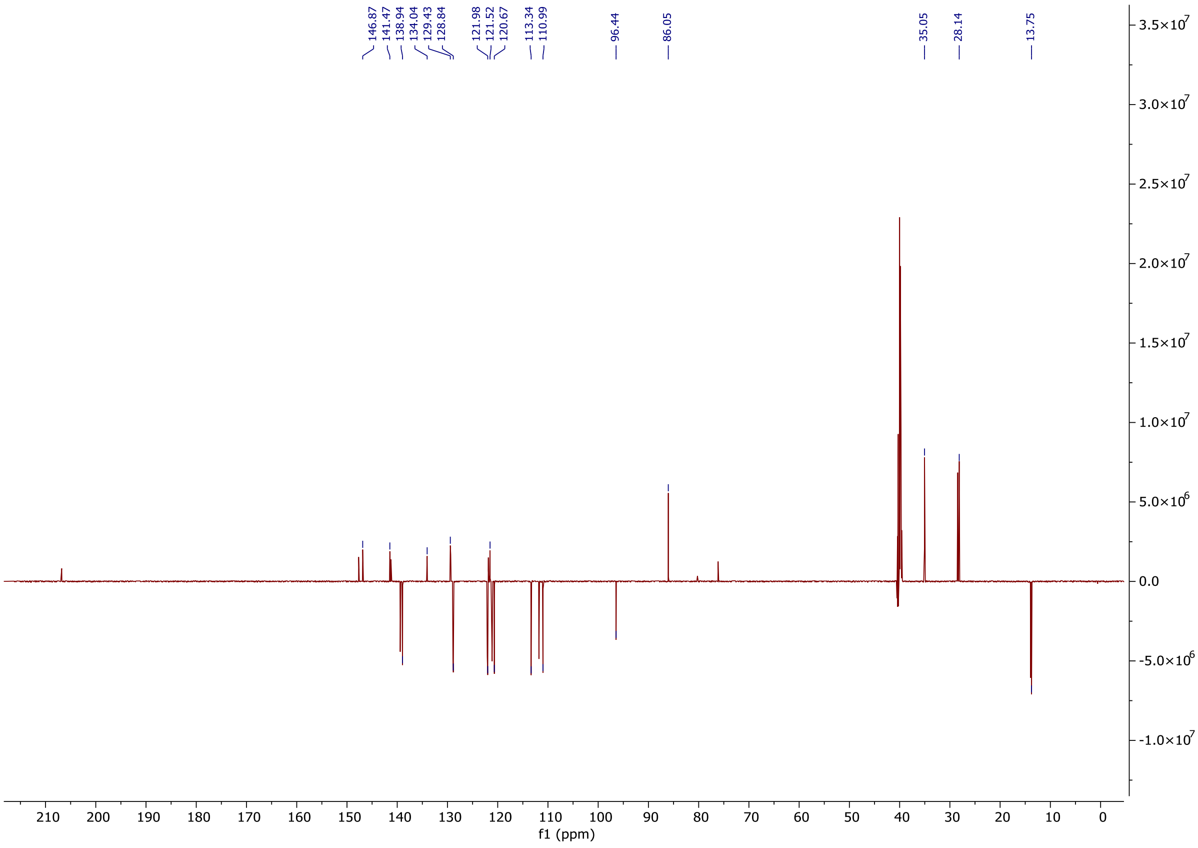
146.87
141.47
138.94
134.04
129.43
128.84
121.98
121.52
120.67
113.34
110.99
96.44
86.05
35.05
28.14
13.75
f1 (ppm)

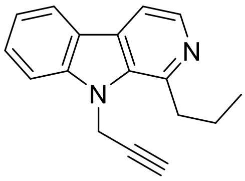

31

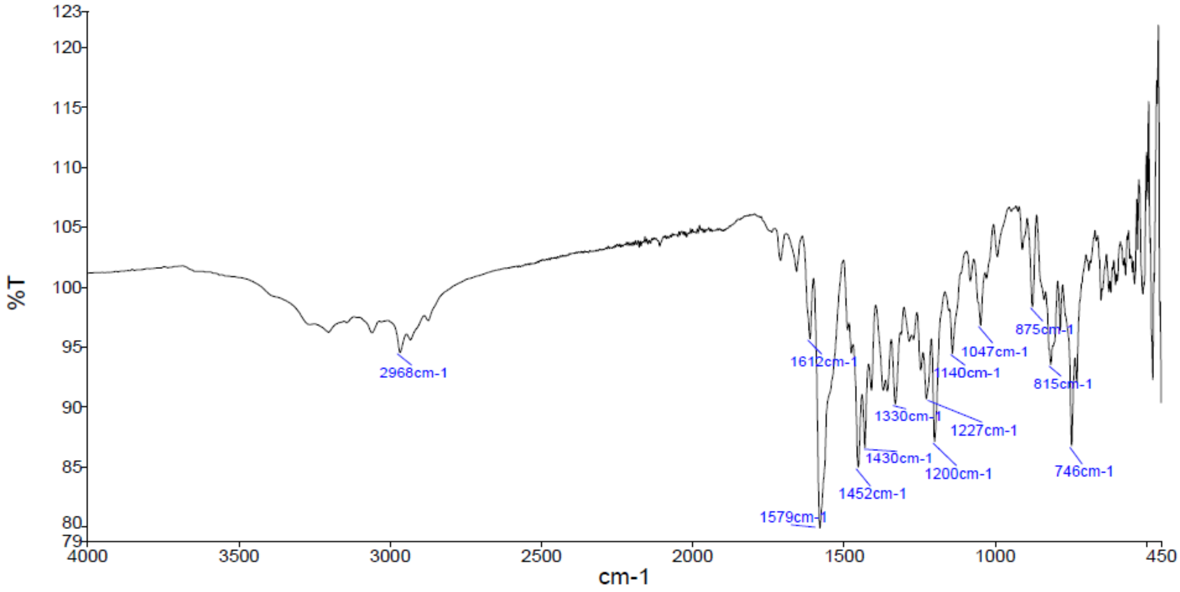
%T
cm-1
2968cm-1
1612cm-1
1579cm-1
1452cm-1
1430cm-1
1330cm-1
1227cm-1
1200cm-1
1140cm-1
1047cm-1
875cm-1
815cm-1
746cm-1

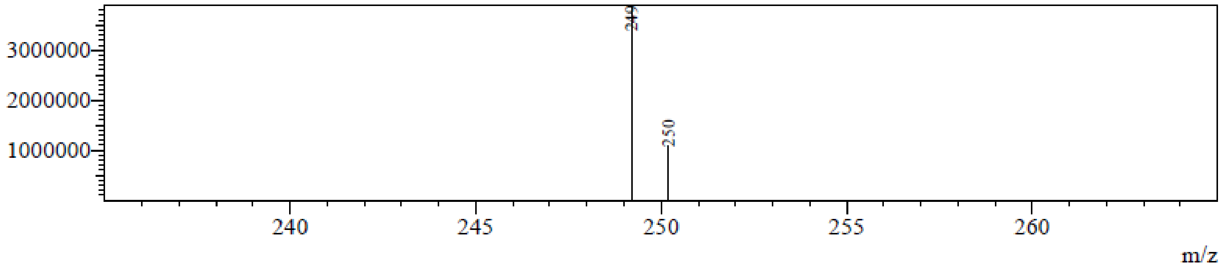
249
250
m/z

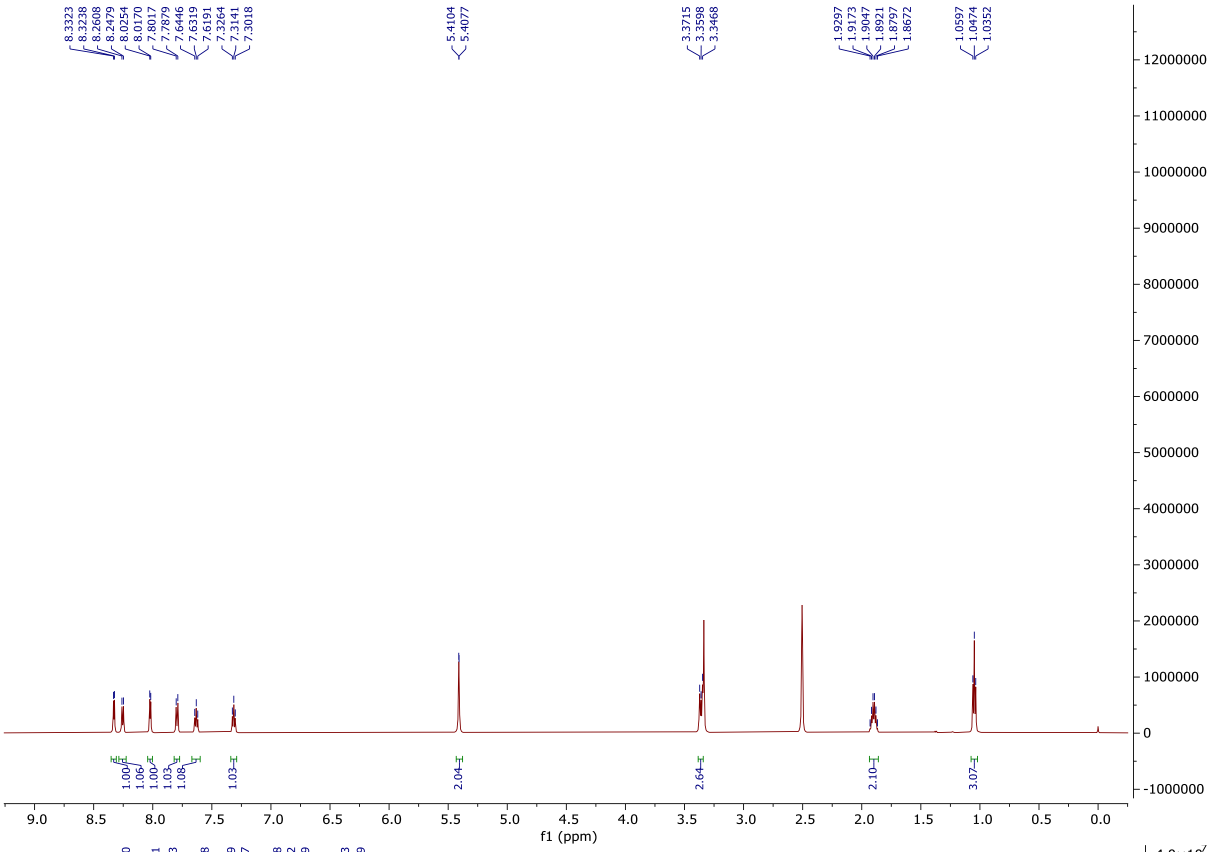
8.3323
8.3238
8.2608
8.2479
8.0254
8.0170
7.8017
7.7879
7.6446
7.6319
7.6191
7.3264
7.3141
7.3018
5.4104
5.4077
3.3715
3.3598
3.3468
1.9297
1.9173
1.9047
1.8921
1.8797
1.8672
1.0597
1.0474
1.0352
1.00
1.06
1.00
1.03
1.08
1.03
2.04
2.64
2.10
3.07
f1 (ppm)

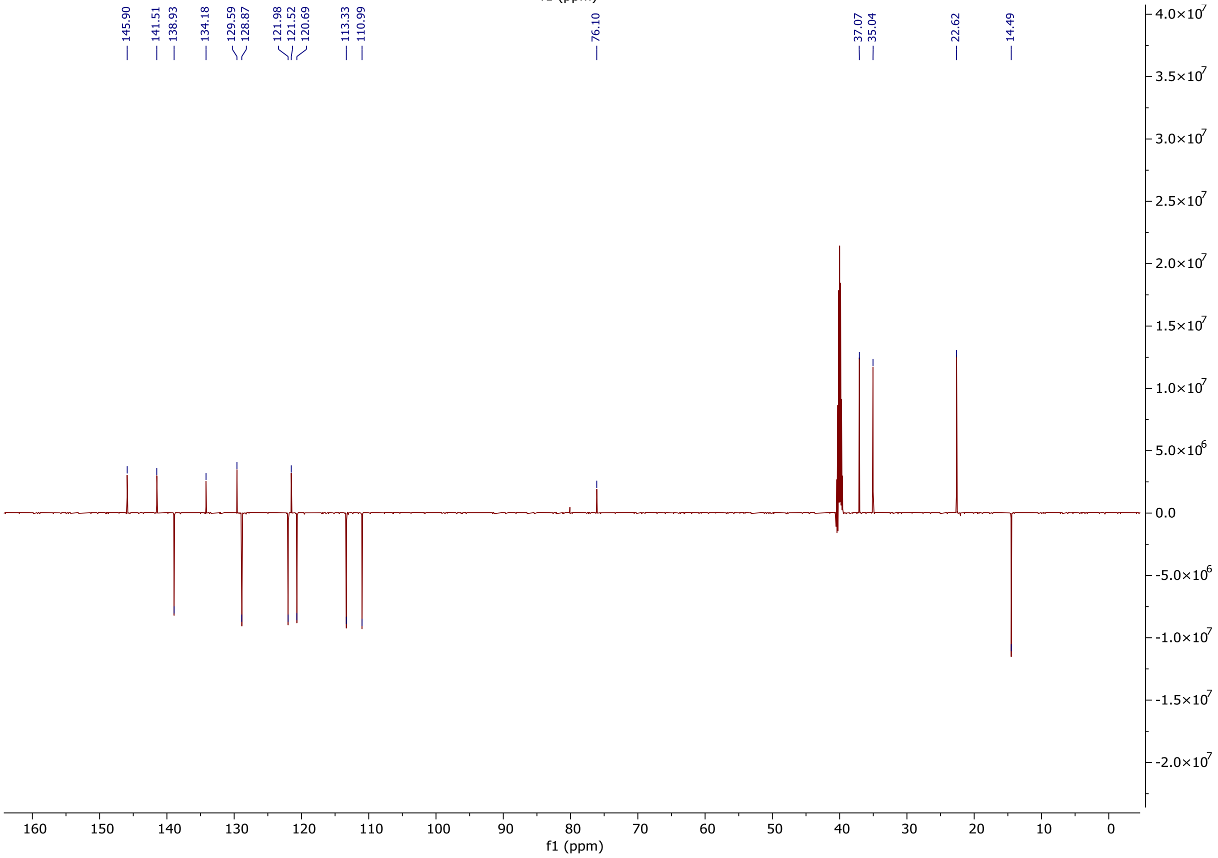
145.90
141.51
138.93
134.18
129.59
128.87
121.98
121.52
120.69
113.33
110.99
76.10
37.07
35.04
22.62
14.49
f1 (ppm)

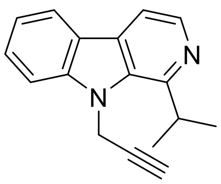

32

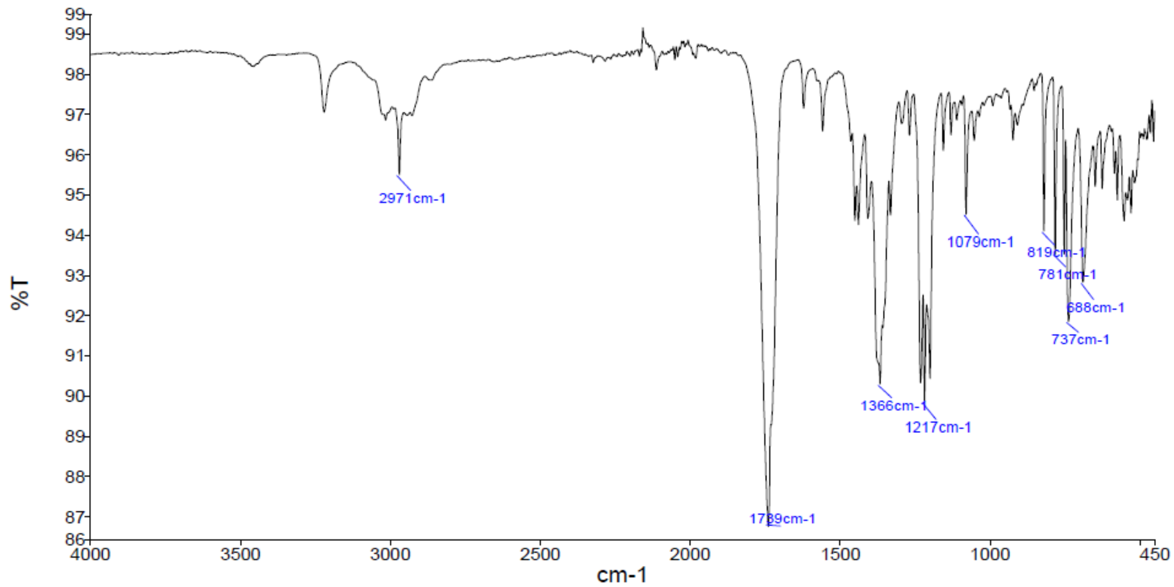

%T
cm-1
2971cm-1
1739cm-1
1366cm-1
1217cm-1
1079cm-1
819cm-1
781cm-1
737cm-1
688cm-1

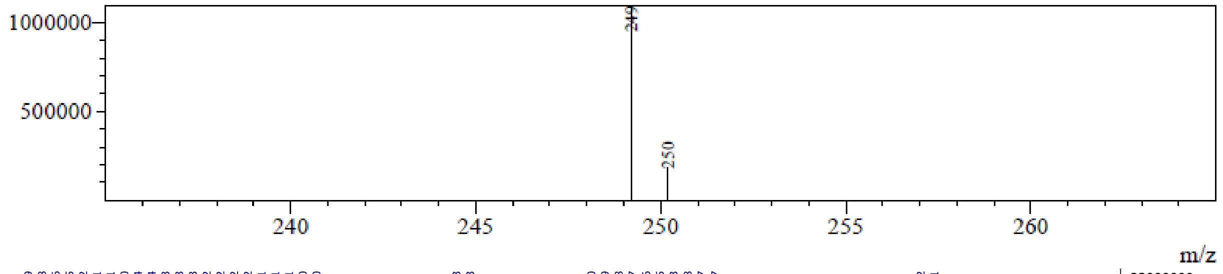

249
250
m/z

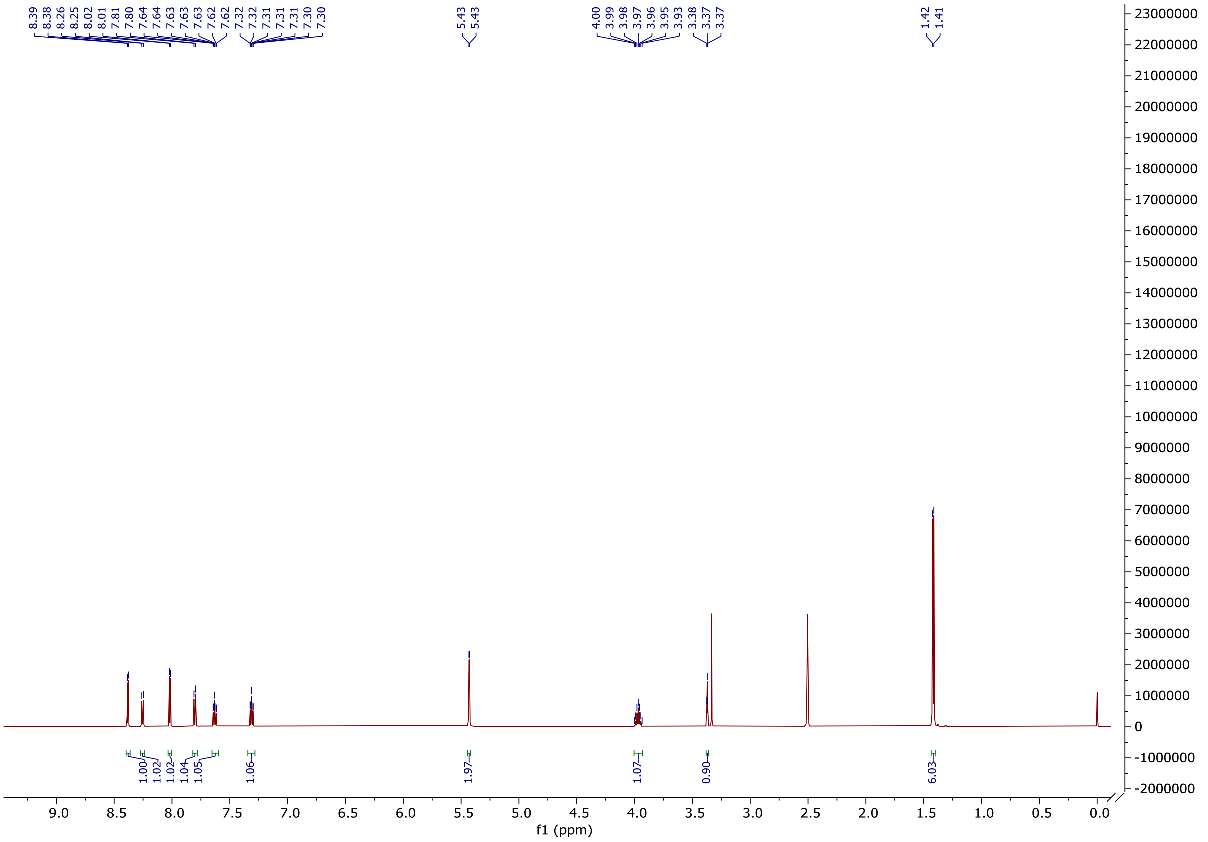

f1 (ppm)

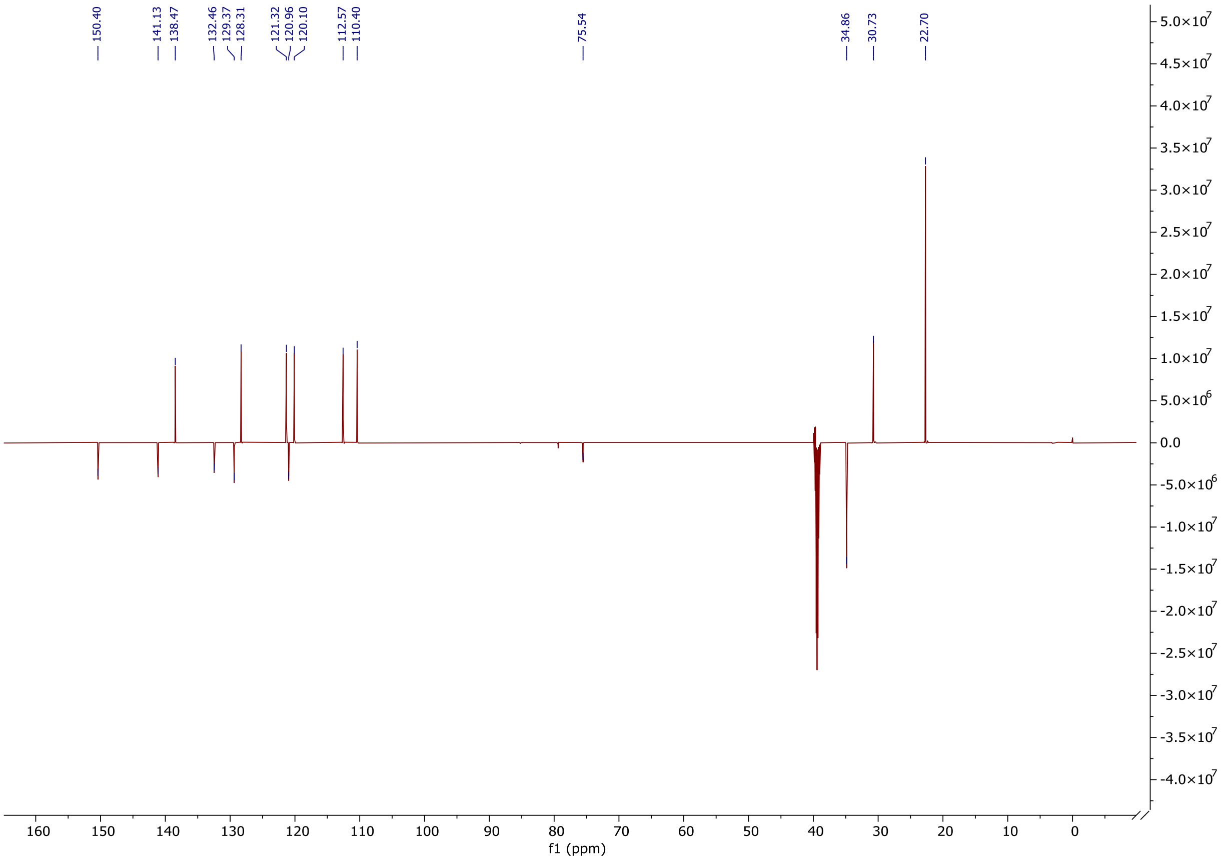

150.40
141.13
138.47
132.46
129.37
128.31
121.32
120.96
120.10
112.57
110.40
75.54
34.86
30.73
22.70
f1 (ppm)

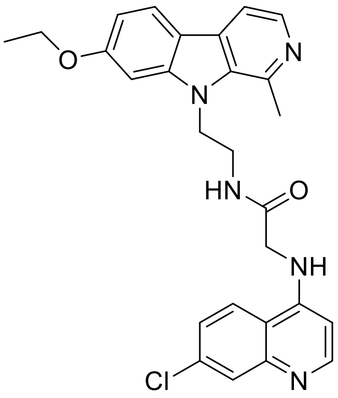

33

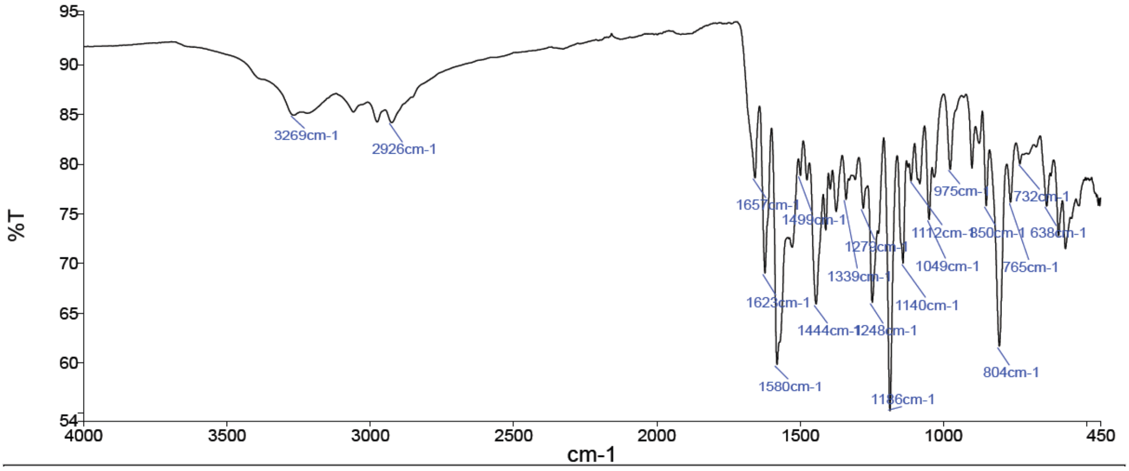

3269cm-1
2926cm-1
1657cm-1
1623cm-1
1580cm-1
1499cm-1
1444cm-1
1339cm-1
1279cm-1
1248cm-1
1186cm-1
1140cm-1
1112cm-1
1049cm-1
975cm-1
850cm-1
804cm-1
765cm-1
732cm-1
638cm-1
%T
cm-1

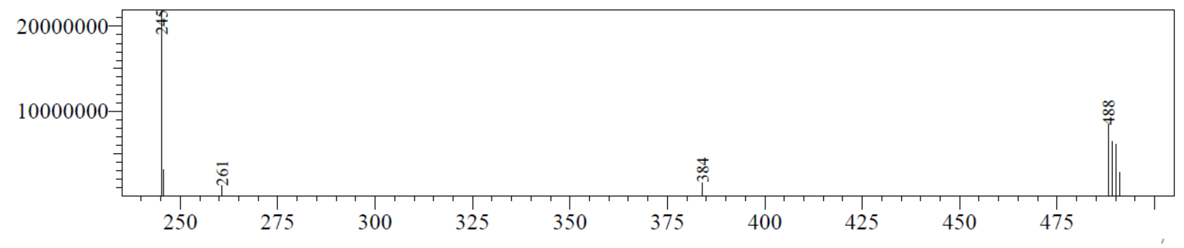

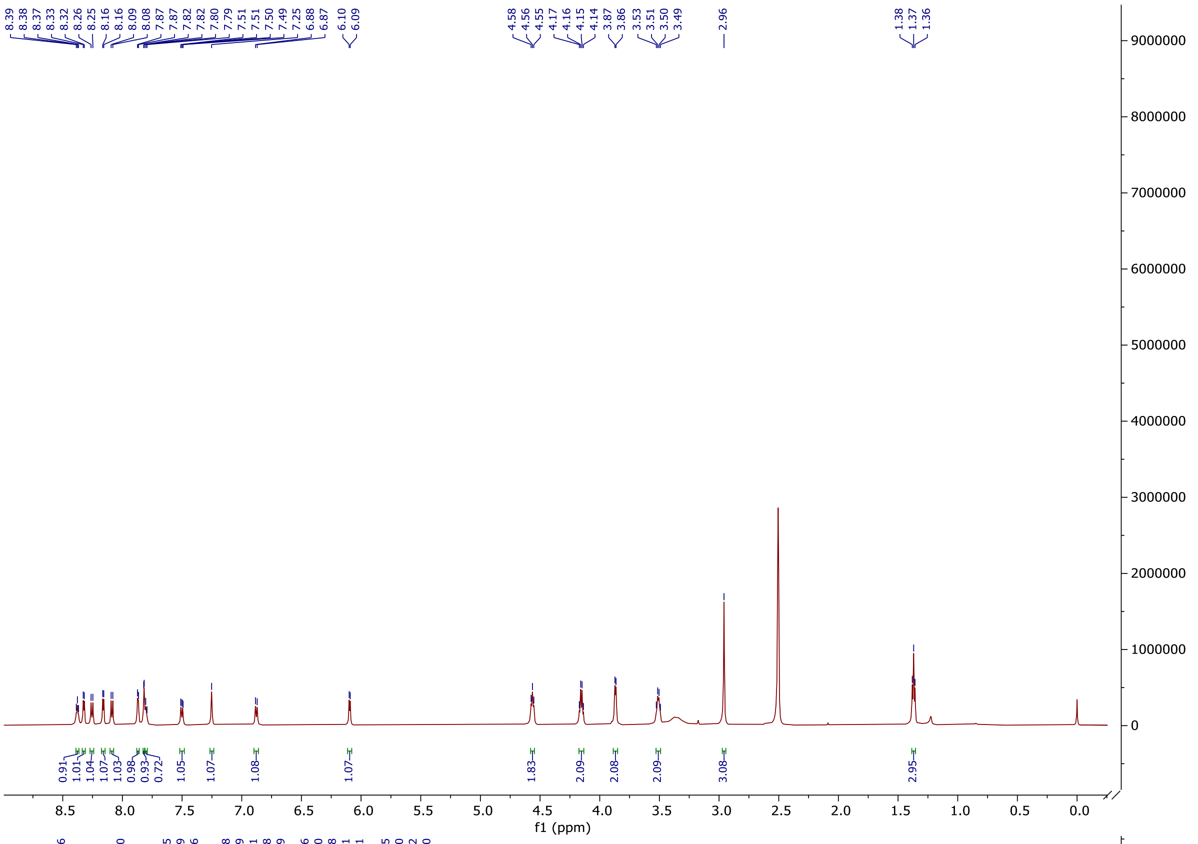

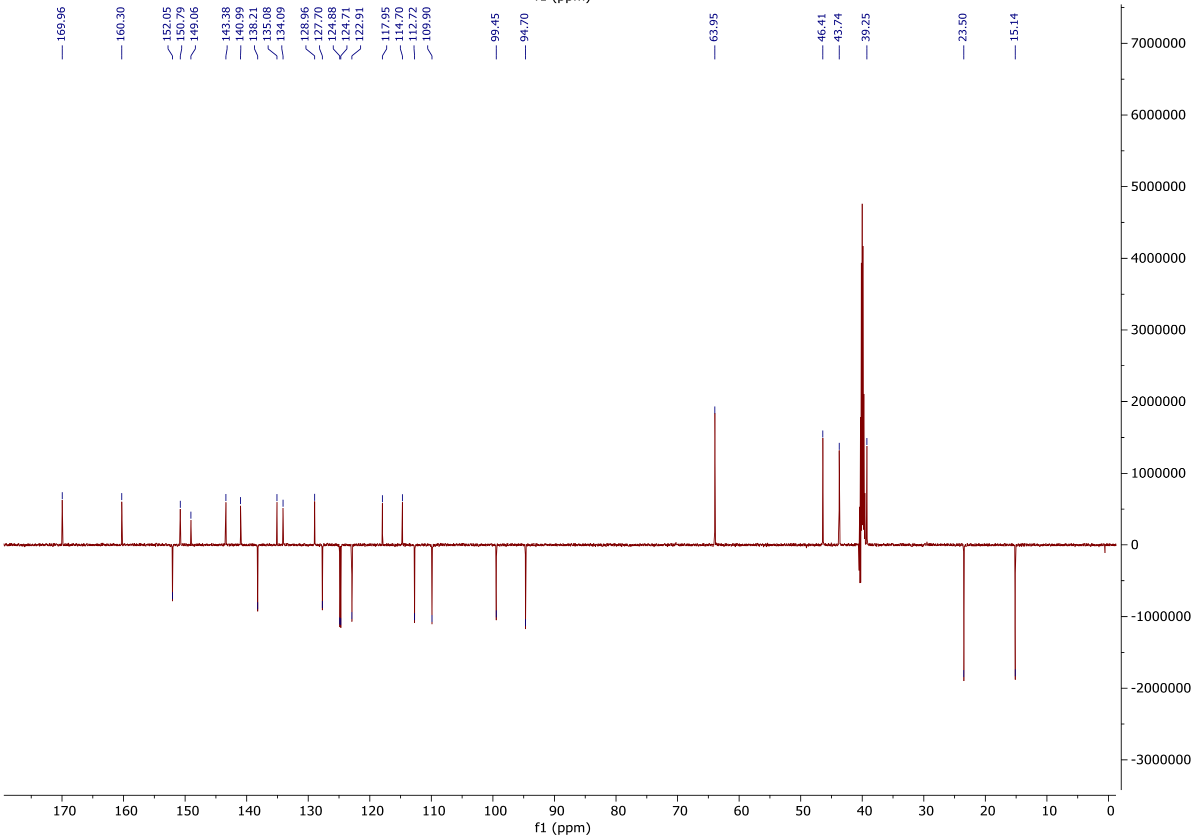

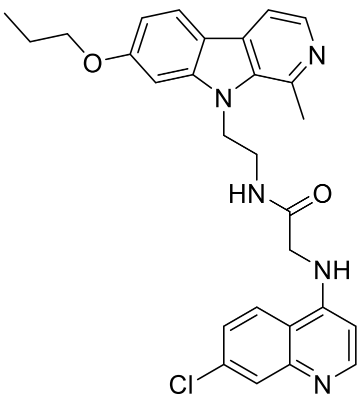
O
N
N
HN
O
NH
Cl
N

34

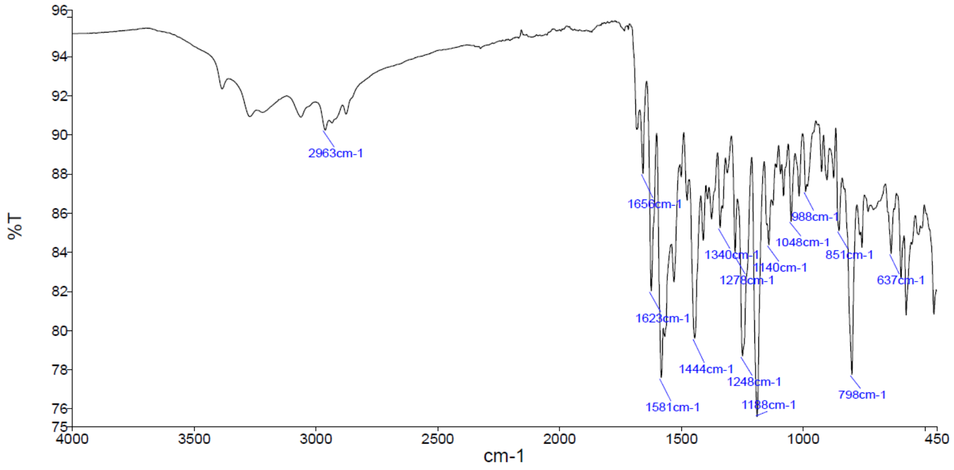
%T
cm-1
2963cm-1
1656cm-1
1623cm-1
1581cm-1
1444cm-1
1340cm-1
1278cm-1
1248cm-1
1188cm-1
1140cm-1
1048cm-1
988cm-1
851cm-1
798cm-1
637cm-1

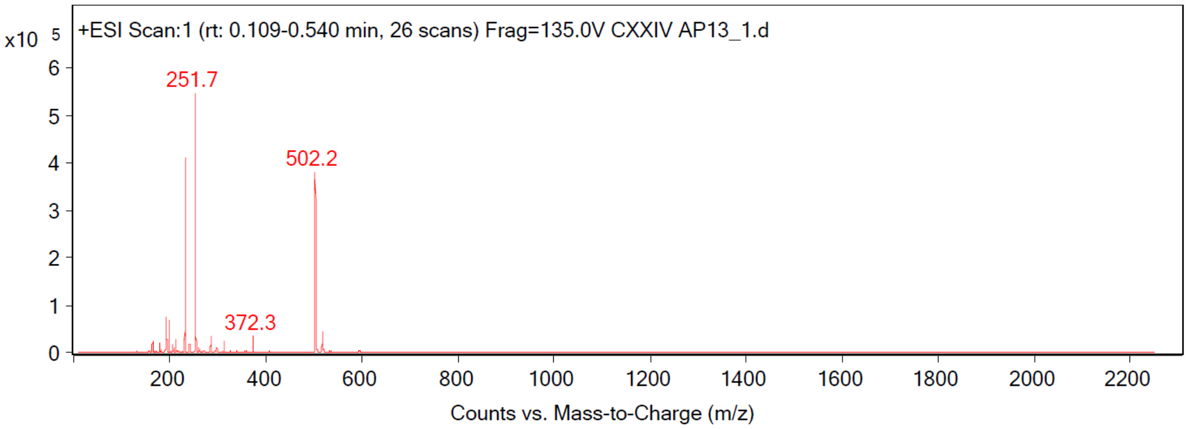
x10 5
+ESI Scan:1 (rt: 0.109-0.540 min, 26 scans) Frag=135.0V CXXIV AP13_1.d
251.7
502.2
372.3
Counts vs. Mass-to-Charge (m/z)

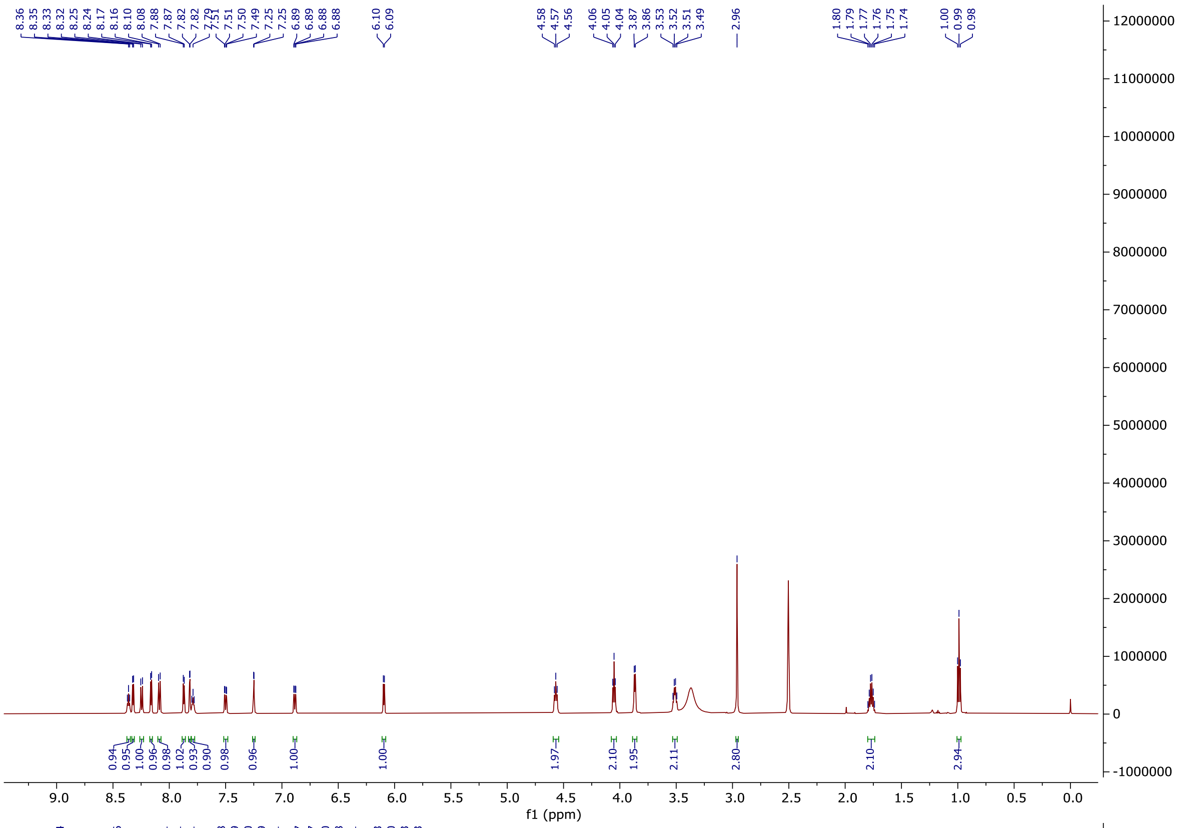
8.36 8.35 8.33 8.32 8.25 8.24 8.17 8.16 8.10 8.08 7.88 7.87 7.82 7.82 7.79 7.51 7.51 7.50 7.49 7.25 7.25 6.89 6.89 6.88 6.88 6.10 6.09 4.58 4.57 4.56 4.06 4.05 4.04 3.87 3.86 3.53 3.52 3.51 3.49 2.96 1.80 1.79 1.77 1.76 1.75 1.74 1.00 0.99 0.98
0.94 0.95 1.00 0.96 0.98 1.02 0.93 0.90 0.98 0.96 1.00 1.00 1.97 2.10 1.95 2.11 2.80 2.10 2.94
f1 (ppm)
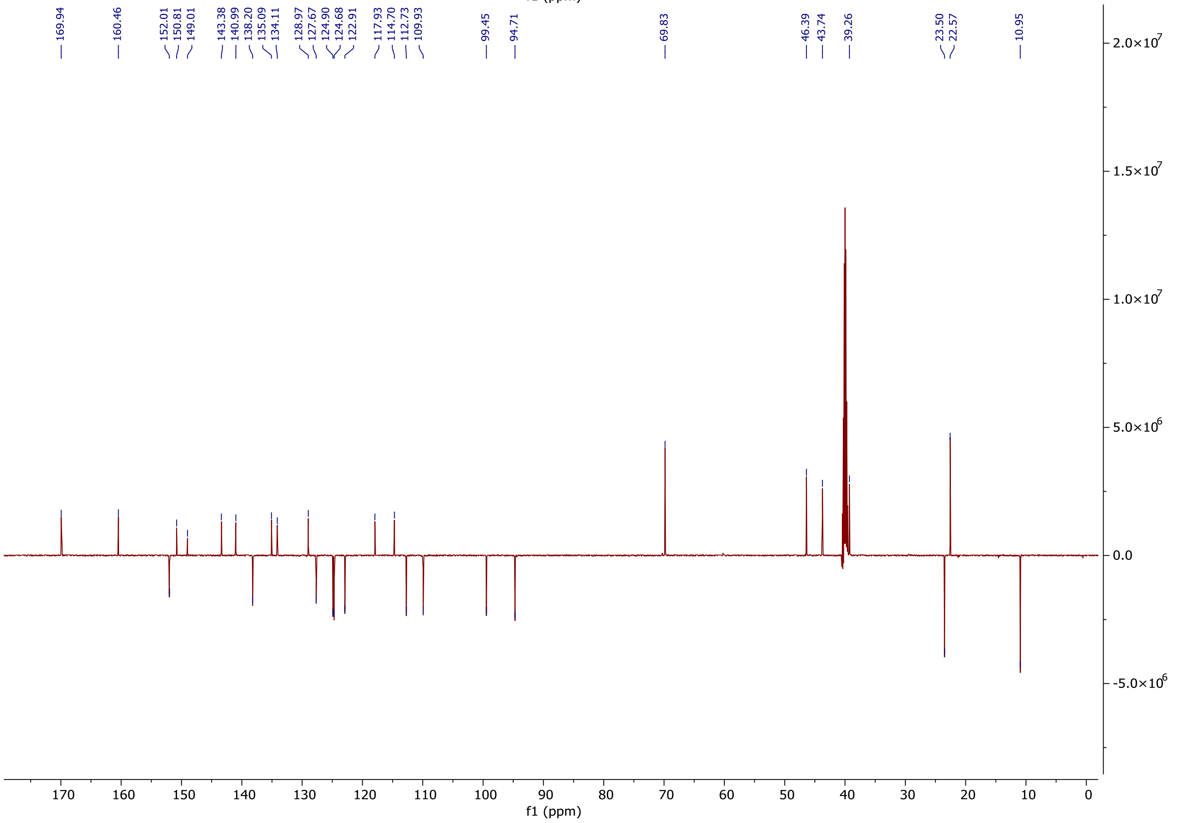
169.94 160.46 152.01 150.81 149.01 143.38 140.99 138.20 135.09 134.11 128.97 127.67 124.90 124.68 122.91 117.93 114.70 112.73 109.93 99.45 94.71 69.83 46.39 43.74 39.26 23.50 22.57 10.95
f1 (ppm)

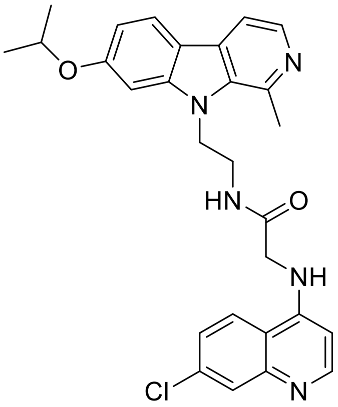
O
N
N
HN
O
NH
Cl
N

35

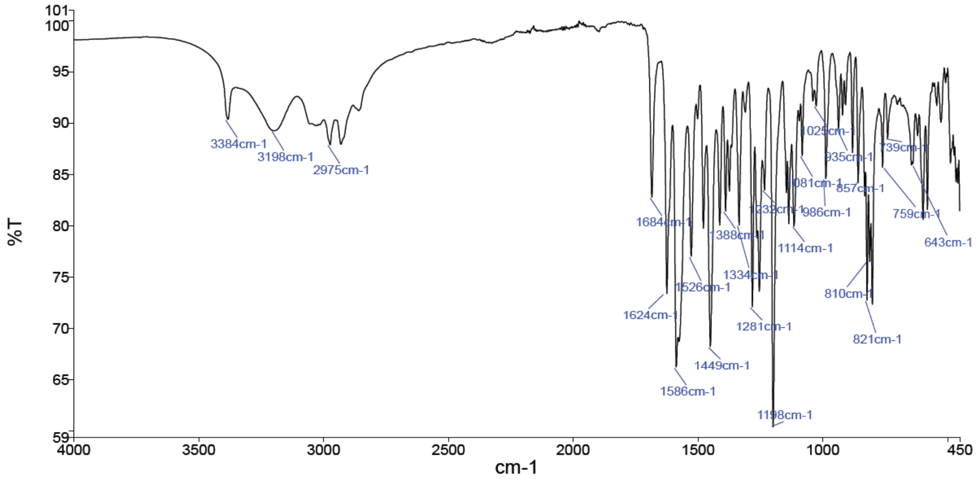
%T
cm-1
3384cm-1
3198cm-1
2975cm-1
1684cm-1
1624cm-1
1586cm-1
1526cm-1
1449cm-1
1388cm-1
1334cm-1
1281cm-1
1235cm-1
1198cm-1
1114cm-1
1081cm-1
1025cm-1
986cm-1
935cm-1
857cm-1
821cm-1
810cm-1
759cm-1
739cm-1
643cm-1

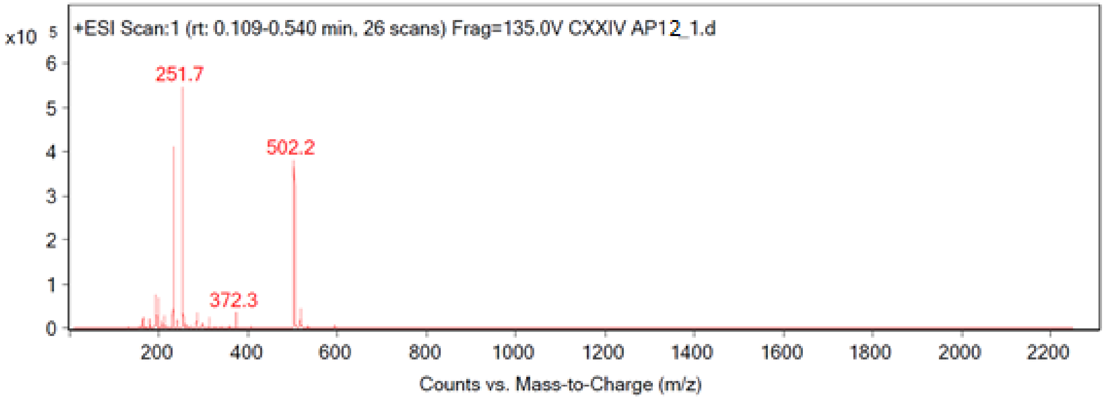
+ESI Scan:1 (rt: 0.109-0.540 min, 26 scans) Frag=135.0V CXXIV AP12_1.d
x10 5
251.7
502.2
372.3
Counts vs. Mass-to-Charge (m/z)

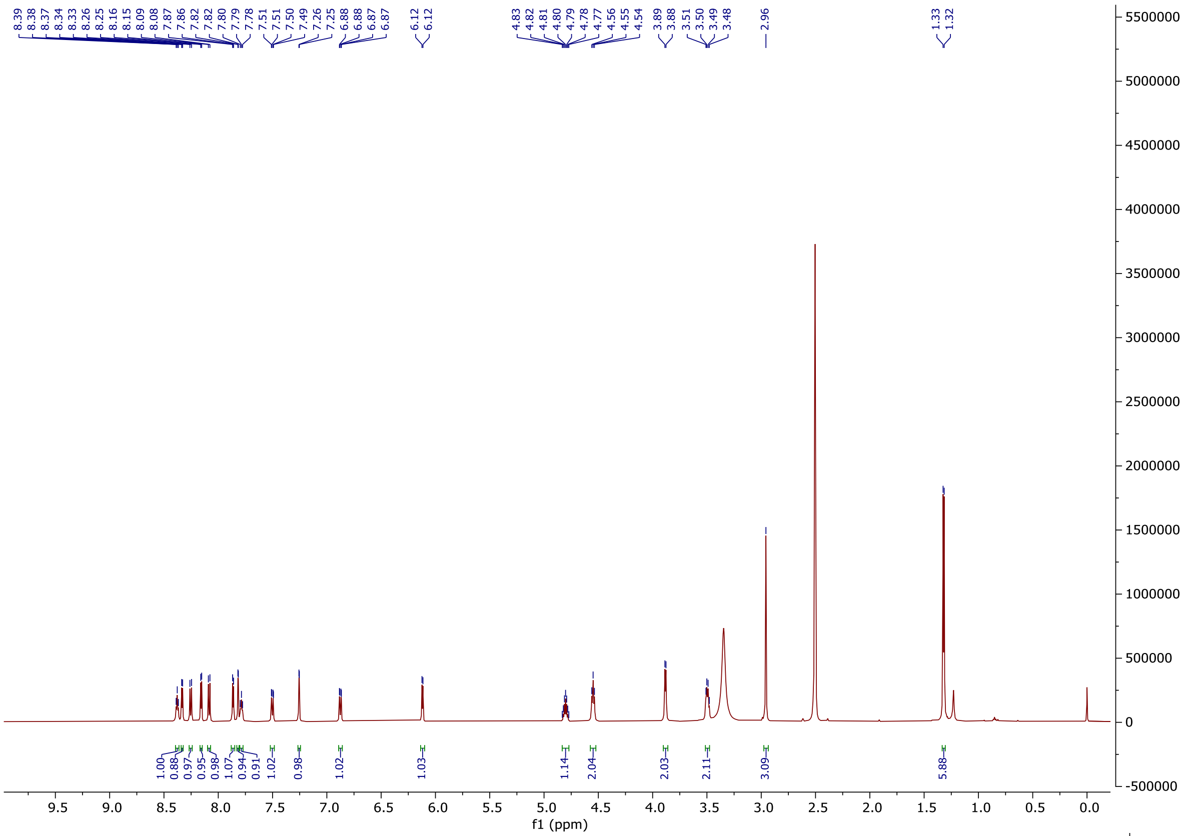

8.39 8.38 8.37 8.34 8.33 8.26 8.25 8.16 8.15 8.09 8.08 7.87 7.86 7.82 7.82 7.80 7.79 7.78 7.51 7.51 7.50 7.49 7.26 7.25 6.88 6.88 6.87 6.87 6.12 6.12 4.83 4.82 4.81 4.80 4.79 4.78 4.77 4.56 4.55 4.54 3.89 3.88 3.51 3.50 3.49 3.48 2.96 1.33 1.32
1.00 0.88 0.97 0.95 0.98 1.07 0.94 0.91 1.02 0.98 1.02 1.03 1.14 2.04 2.03 2.11 3.09 5.88
f1 (ppm)

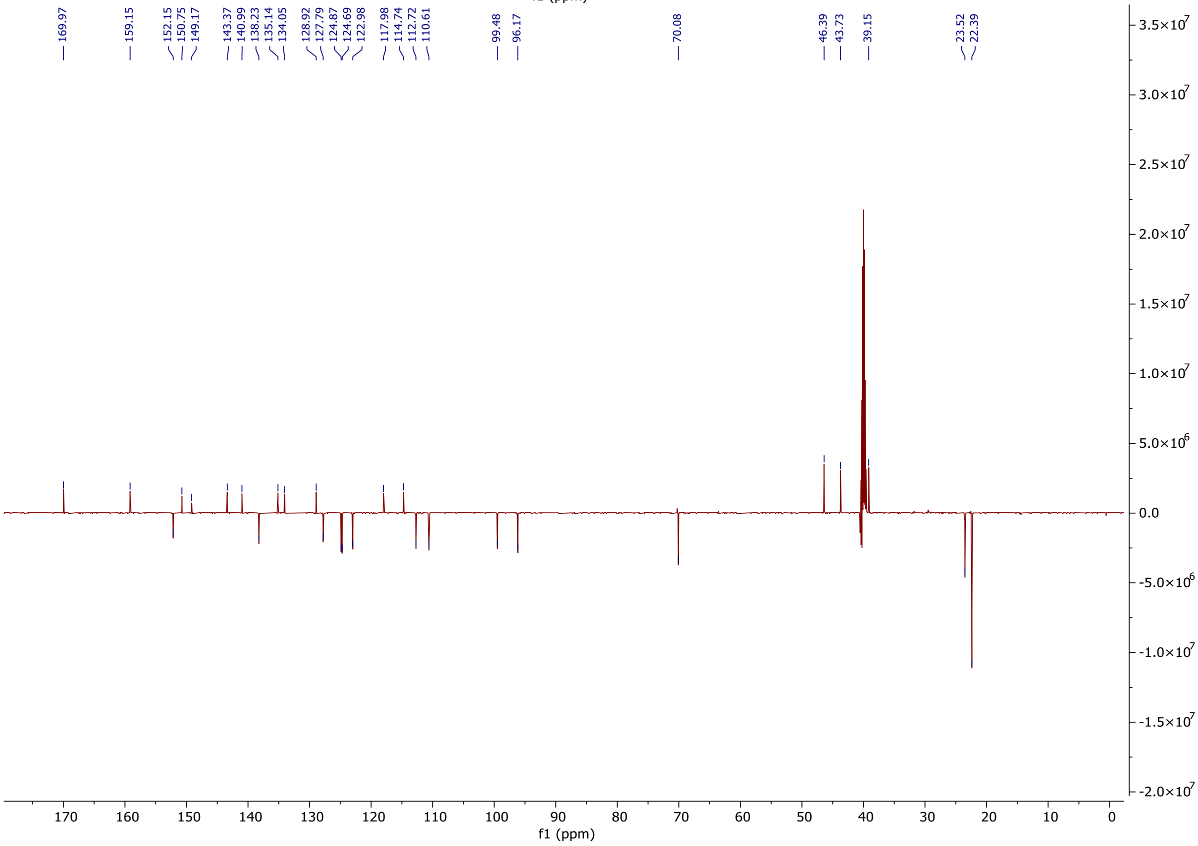

169.97 159.15 152.15 150.75 149.17 143.37 140.99 138.23 135.14 134.05 128.92 127.79 124.87 124.69 122.98 117.98 114.74 112.72 110.61 99.48 96.17 70.08 46.39 43.73 39.15 23.52 22.39
f1 (ppm)

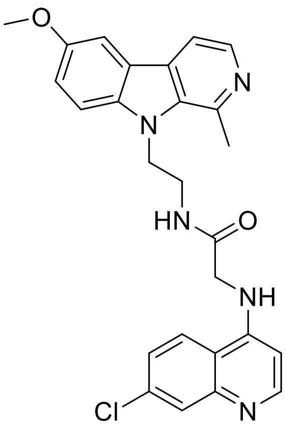

36

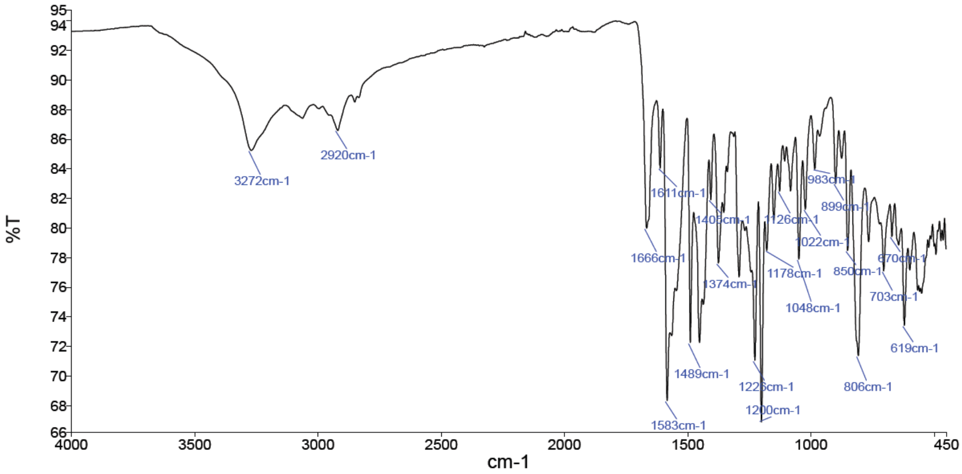
%T
cm-1
3272cm-1
2920cm-1
1666cm-1
1611cm-1
1583cm-1
1489cm-1
1374cm-1
1226cm-1
1200cm-1
1178cm-1
1126cm-1
1048cm-1
1022cm-1
983cm-1
899cm-1
850cm-1
806cm-1
703cm-1
670cm-1
619cm-1

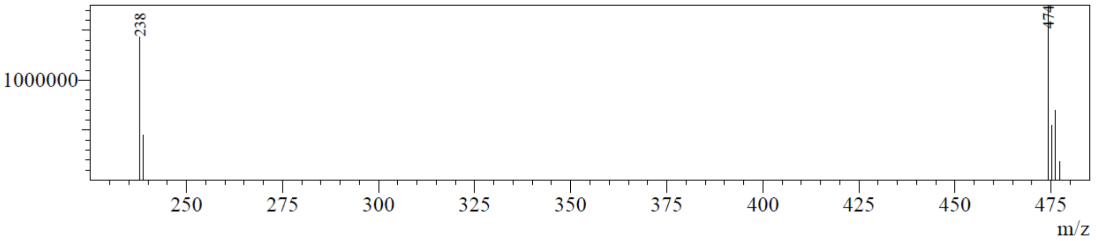
238
474
1000000
m/z

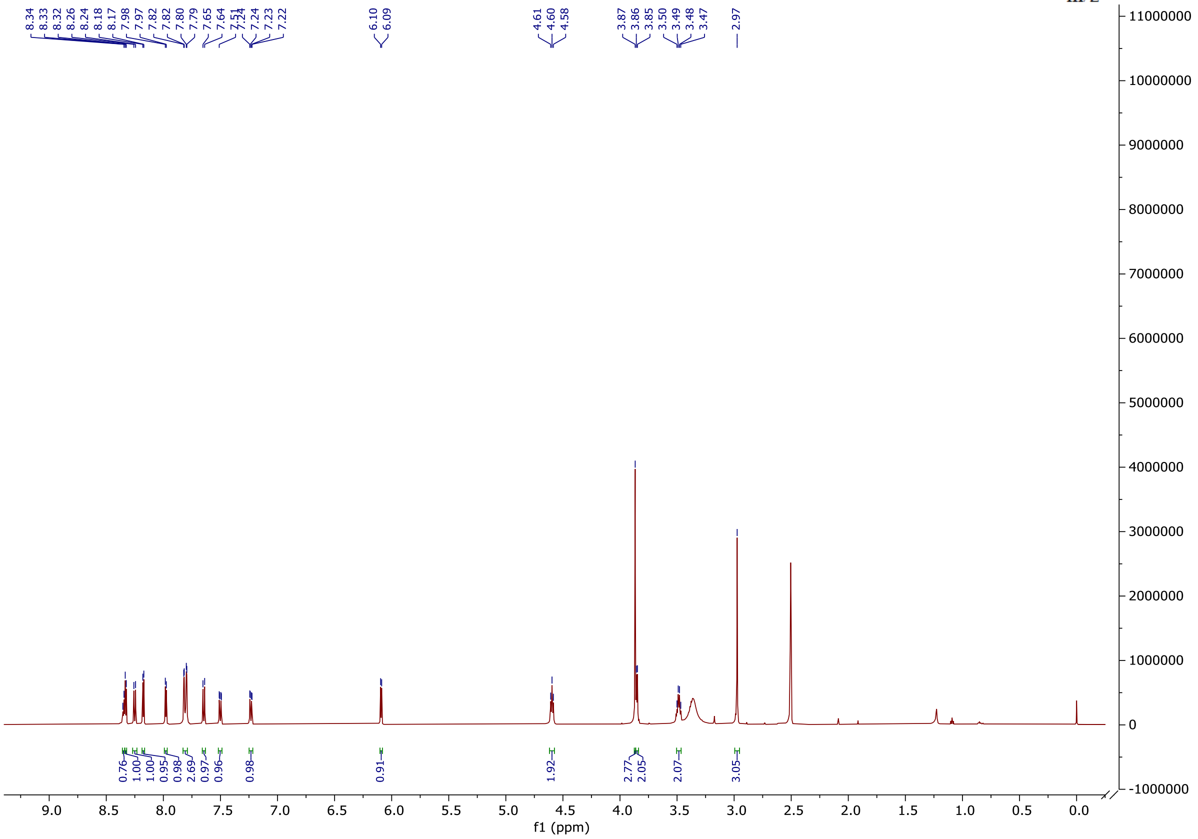
8.34
8.33
8.32
8.26
8.24
8.18
8.17
7.98
7.97
7.82
7.80
7.79
7.65
7.64
7.51
7.24
7.24
7.23
7.22
6.10
6.09
4.61
4.60
4.58
3.87
3.86
3.85
3.50
3.49
3.48
3.47
2.97
f1 (ppm)

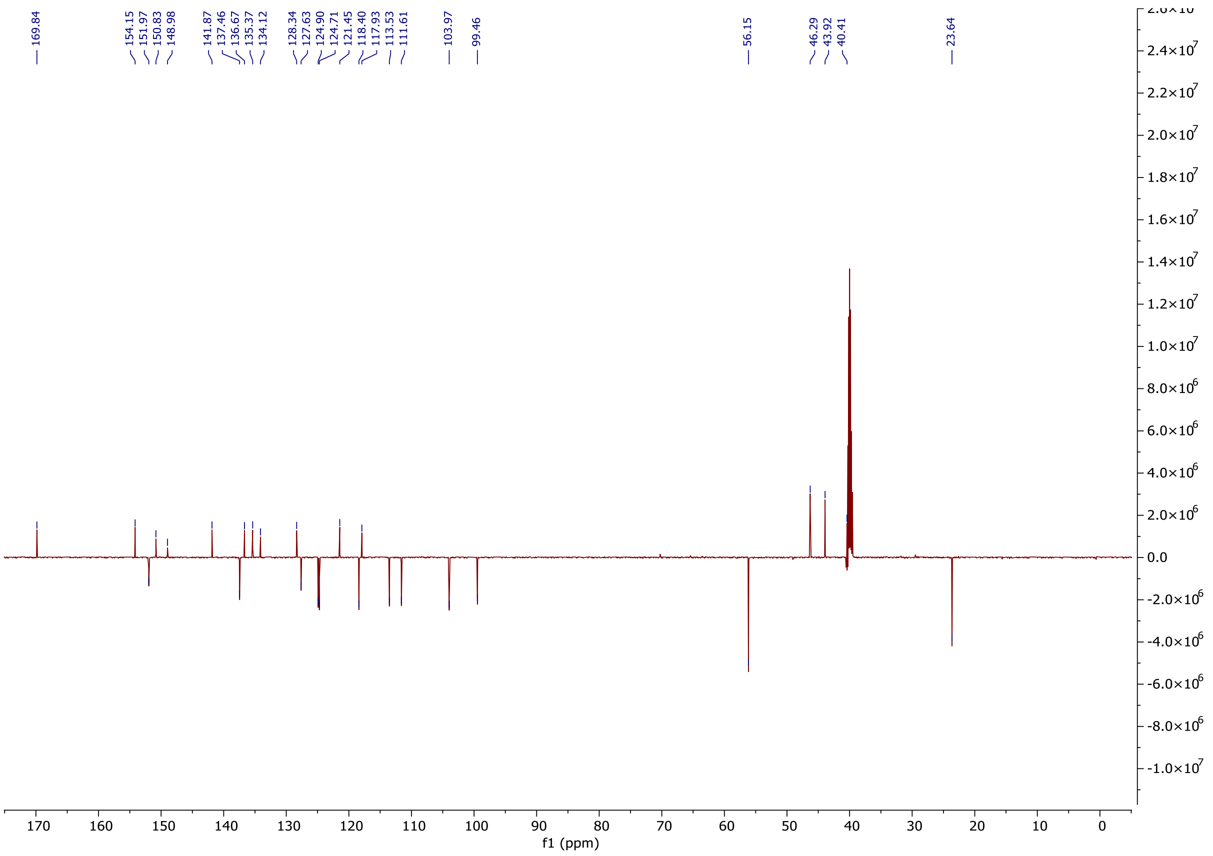
169.84
154.15
151.97
150.83
148.98
141.87
137.46
136.67
135.37
134.12
128.34
127.63
124.90
124.71
121.45
118.40
117.93
113.53
111.61
103.97
99.46
56.15
46.29
43.92
40.41
23.64
f1 (ppm)

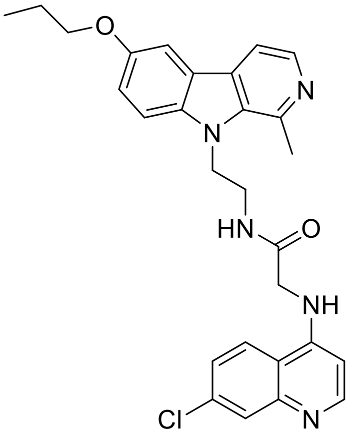
O
N
N
HN
O
NH
Cl
N

37

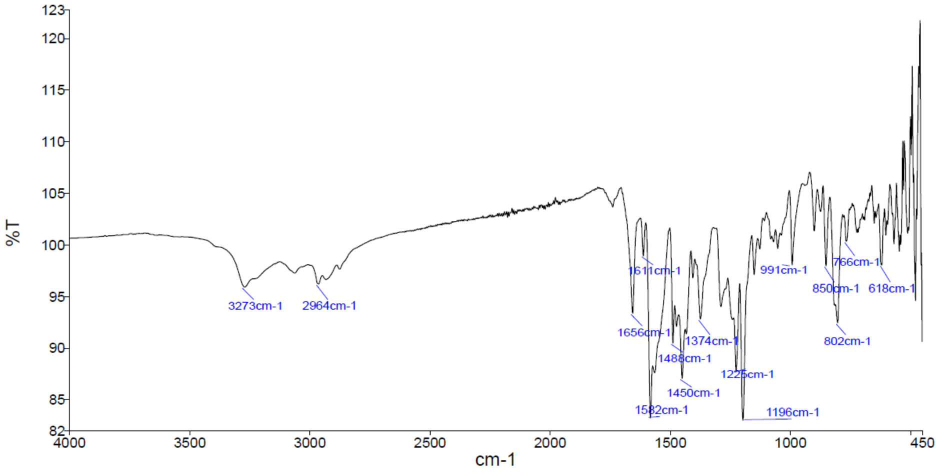
%T
cm-1
3273cm-1
2964cm-1
1611cm-1
1656cm-1
1582cm-1
1488cm-1
1450cm-1
1374cm-1
1225cm-1
1196cm-1
991cm-1
850cm-1
802cm-1
766cm-1
618cm-1

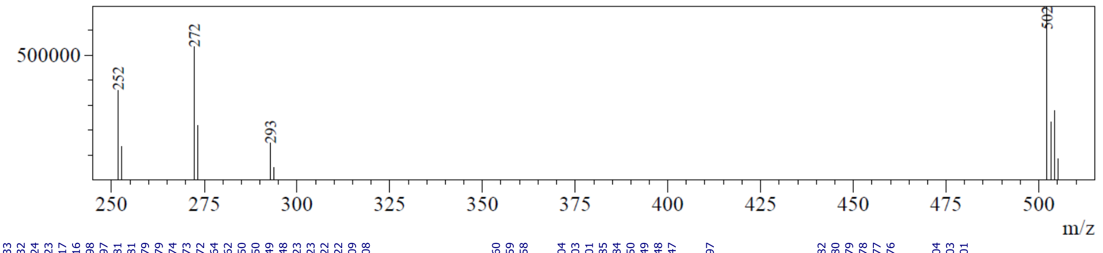

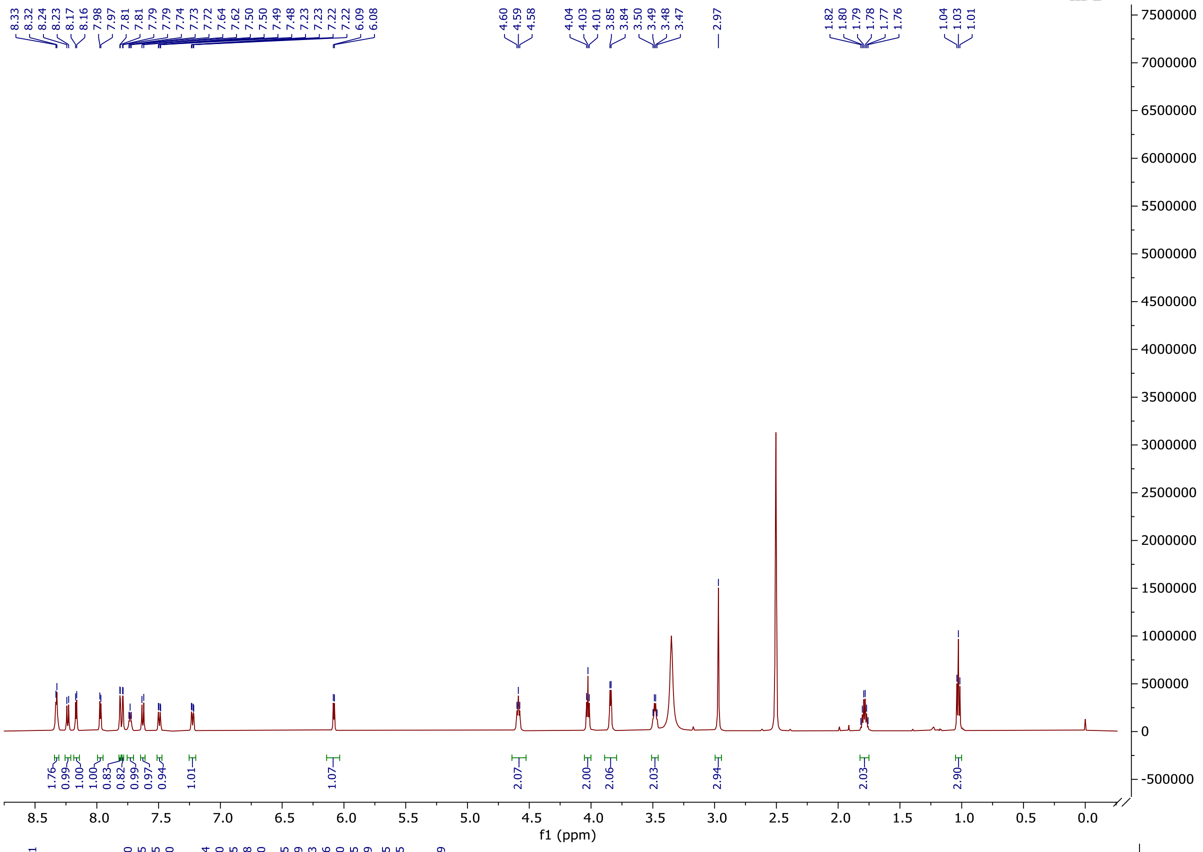

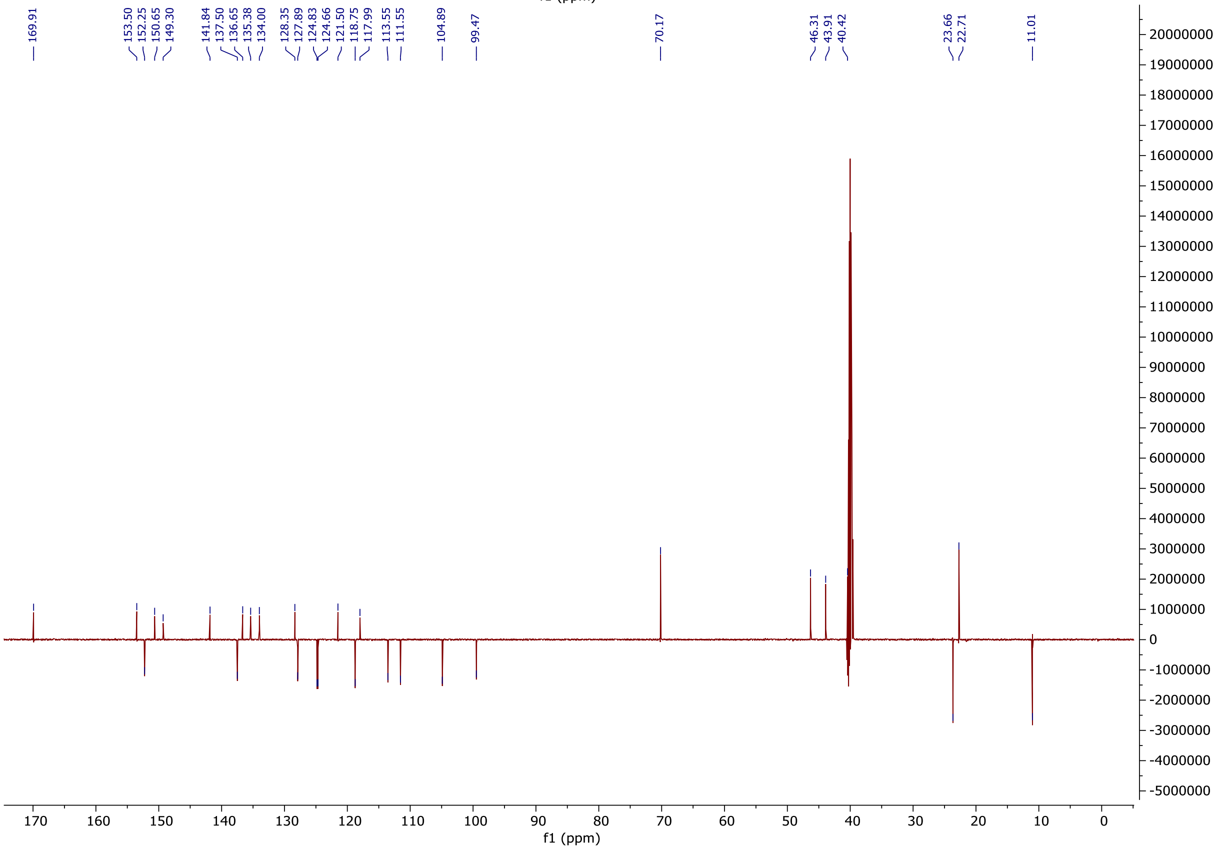

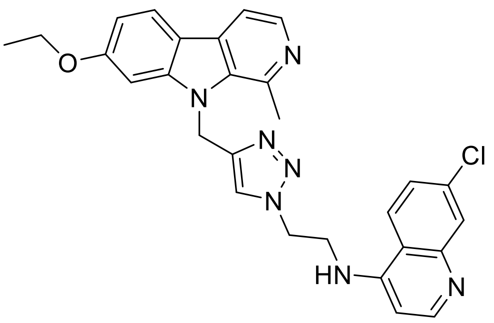

38

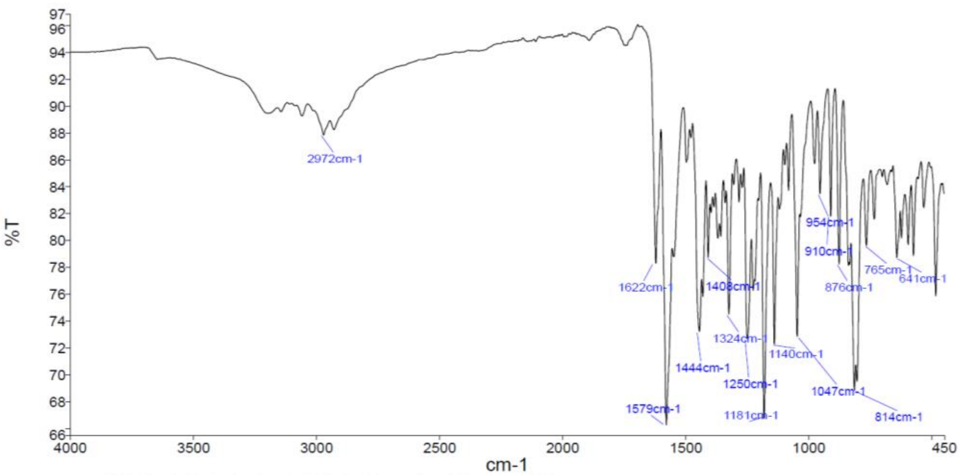

%T
cm-1
2972cm-1
1622cm-1
1579cm-1
1444cm-1
1408cm-1
1324cm-1
1250cm-1
1181cm-1
1140cm-1
1047cm-1
954cm-1
910cm-1
876cm-1
814cm-1
765cm-1
641cm-1

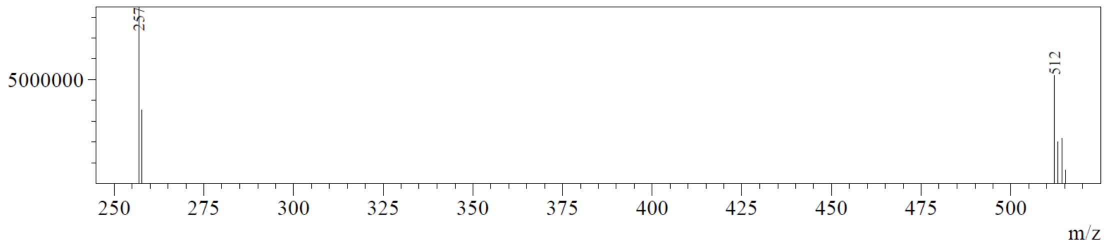

257
512
5000000
m/z

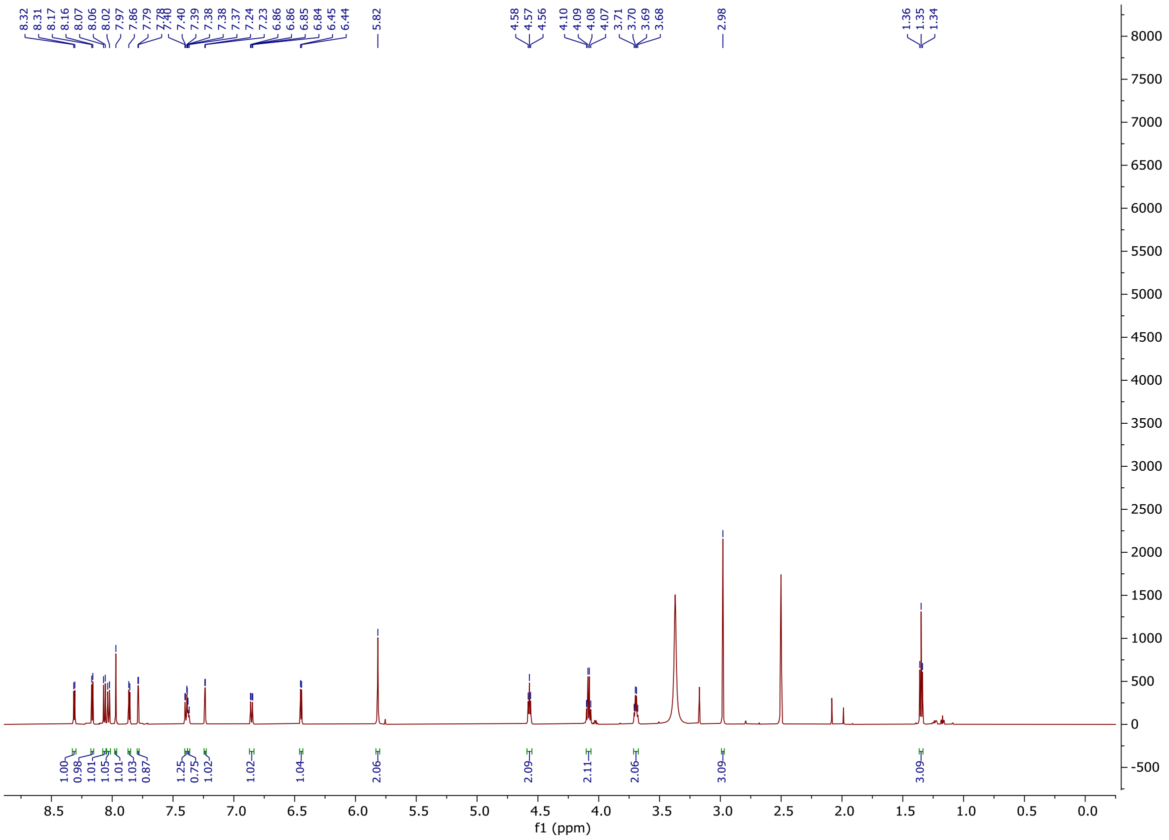

8.32 8.31 8.17 8.16 8.07 8.06 8.02 7.97 7.86 7.79 7.40 7.39 7.39 7.38 7.38 7.37 7.24 7.23 6.86 6.86 6.85 6.84 6.45 6.44 5.82
4.58 4.57 4.56 4.10 4.09 4.08 4.07 3.71 3.70 3.69 3.68 2.98 1.36 1.35 1.34
1.00 0.98 1.01 1.05 1.01 1.03 0.87 1.25 0.75 1.02 1.02 1.04 2.06 2.09 2.11 2.06 3.09 3.09
f1 (ppm)

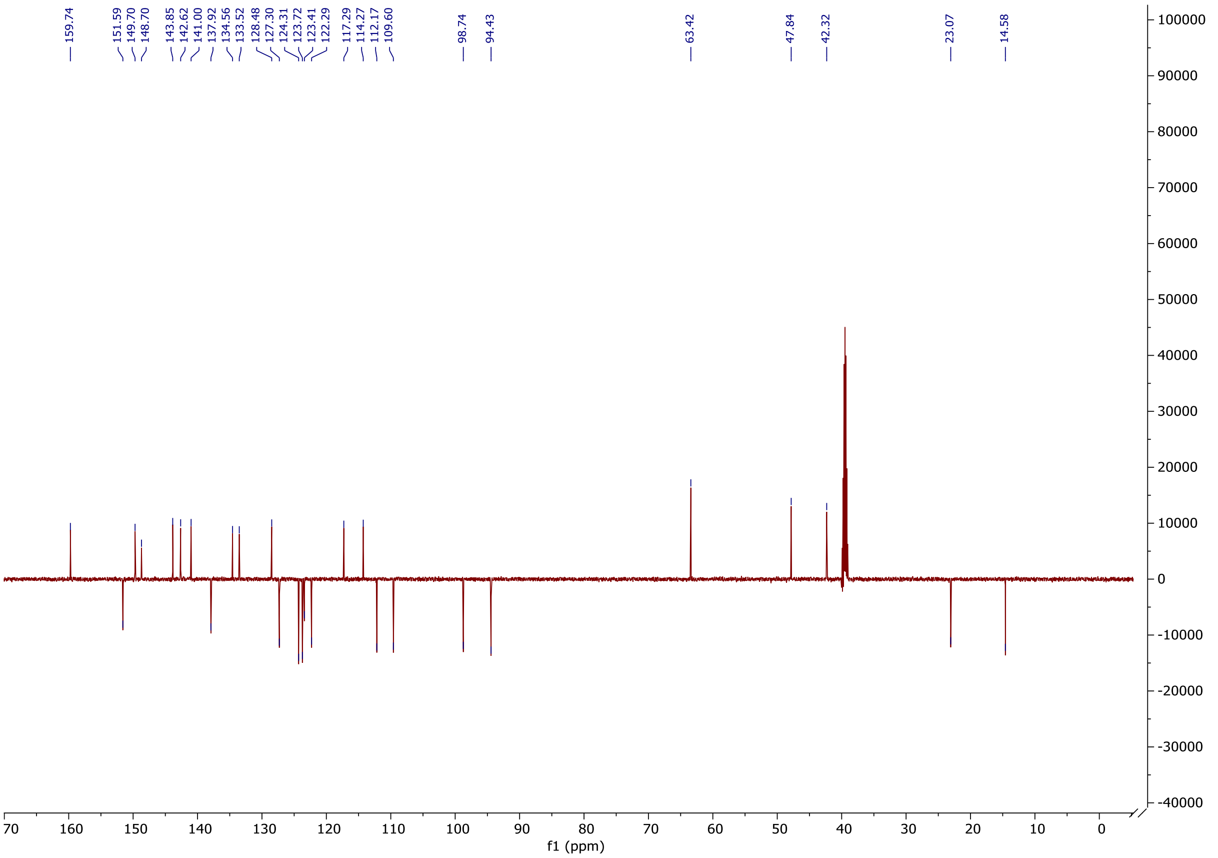
159.74
151.59
149.70
148.70
143.85
142.62
141.00
137.92
134.56
133.52
128.48
127.30
124.31
123.72
123.41
122.29
117.29
114.27
112.17
109.60
98.74
94.43
63.42
47.84
42.32
23.07
14.58
f1 (ppm)

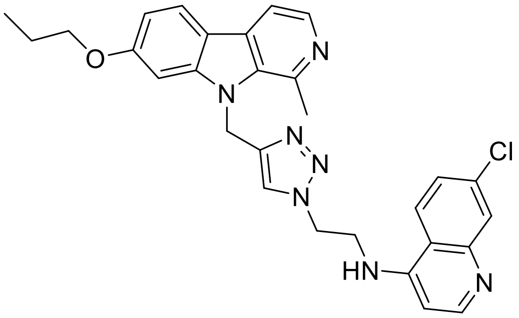

39

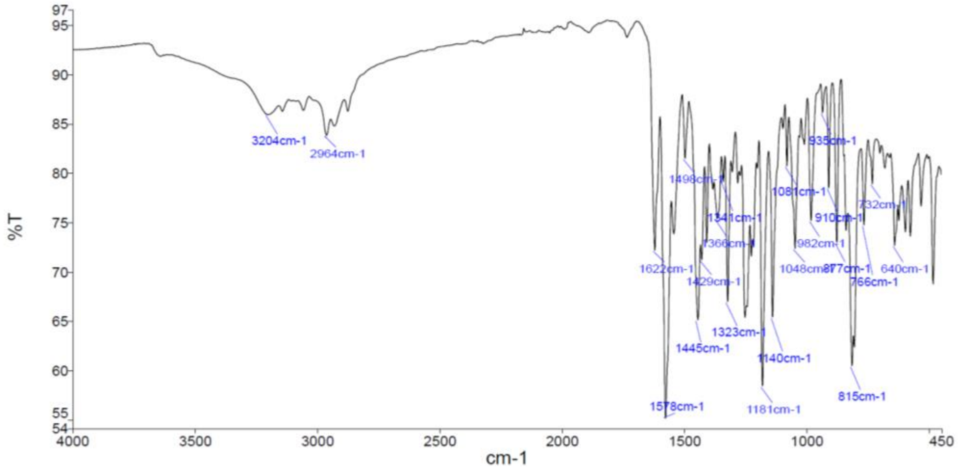
3204cm-1
2964cm-1
1578cm-1
1445cm-1
1323cm-1
1181cm-1
1140cm-1
815cm-1
%T
cm-1

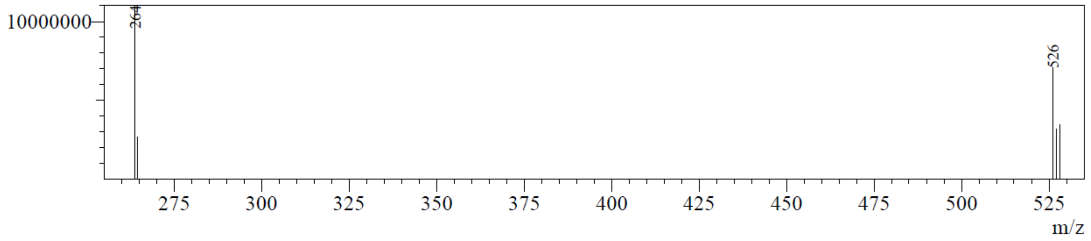
264
526
m/z

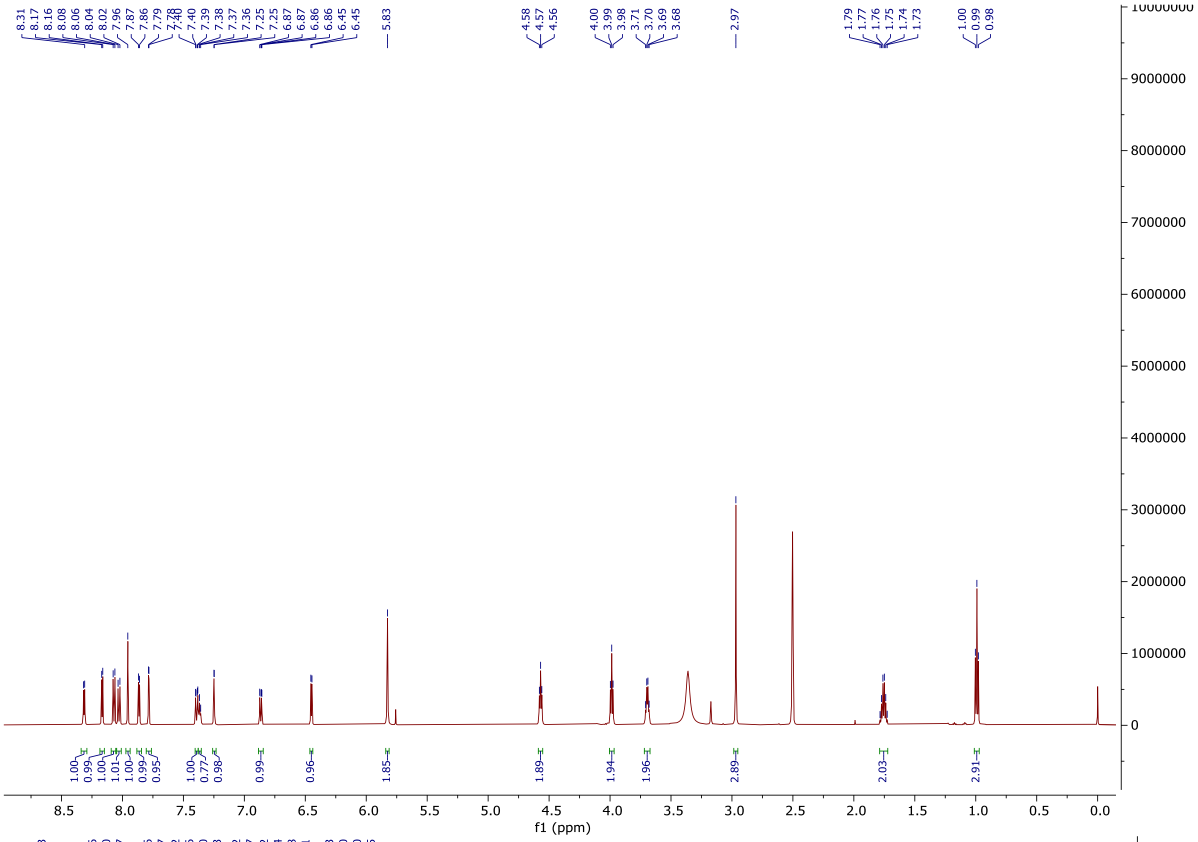
8.31
8.17
8.16
8.08
8.06
8.04
8.02
7.96
7.87
7.86
7.79
7.40
7.39
7.38
7.37
7.36
7.25
7.25
6.87
6.87
6.86
6.86
6.45
6.45
5.83
4.58
4.57
4.56
4.00
3.99
3.98
3.71
3.70
3.69
3.68
2.97
1.79
1.77
1.76
1.75
1.74
1.73
1.00
0.99
0.98
f1 (ppm)

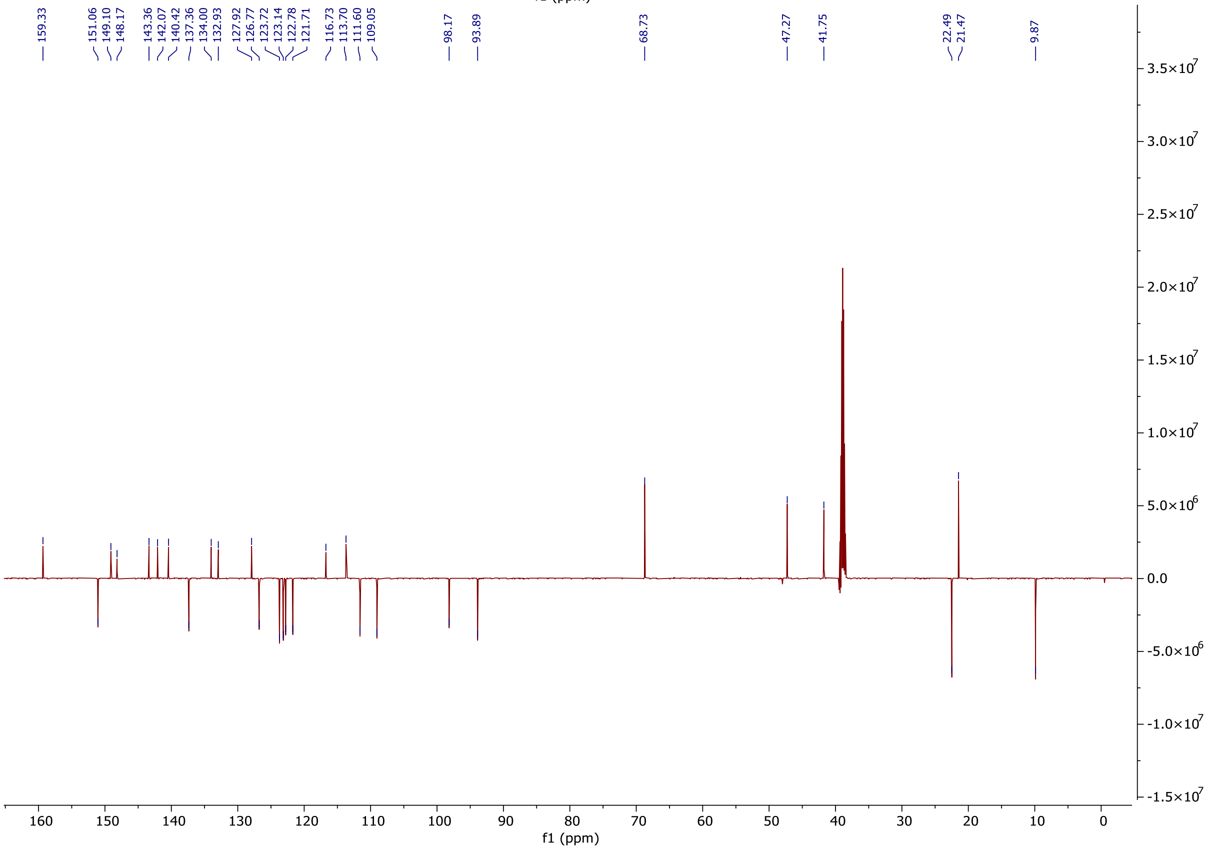
159.33
151.06
149.10
148.17
143.36
142.07
140.42
137.36
134.00
132.93
127.92
126.77
123.72
123.14
122.78
121.71
116.73
113.70
111.60
109.05
98.17
93.89
68.73
47.27
41.75
22.49
21.47
9.87
f1 (ppm)

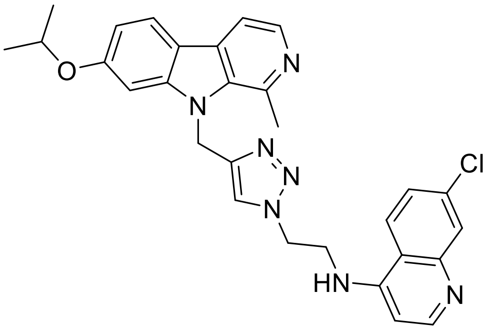

40

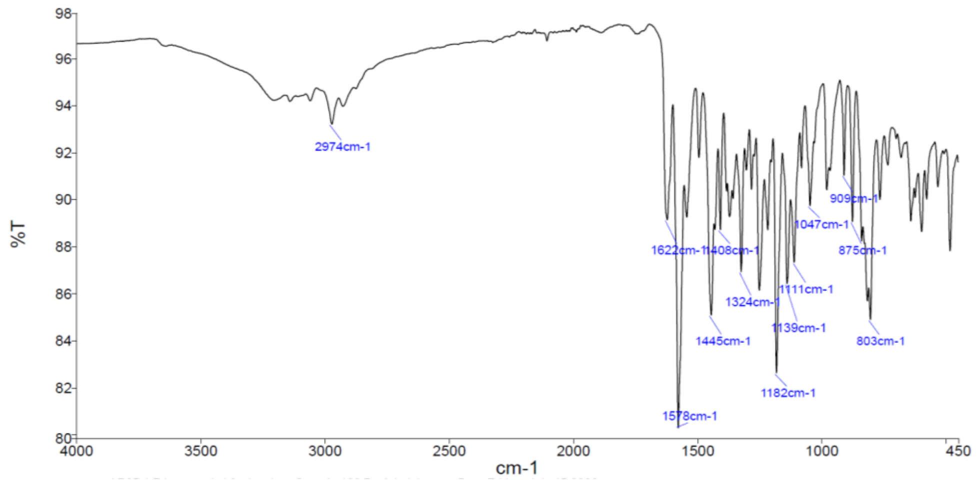

%T
cm-1
2974cm-1
1622cm-1
1578cm-1
1445cm-1
1408cm-1
1324cm-1
1182cm-1
1139cm-1
1111cm-1
1047cm-1
909cm-1
875cm-1
803cm-1

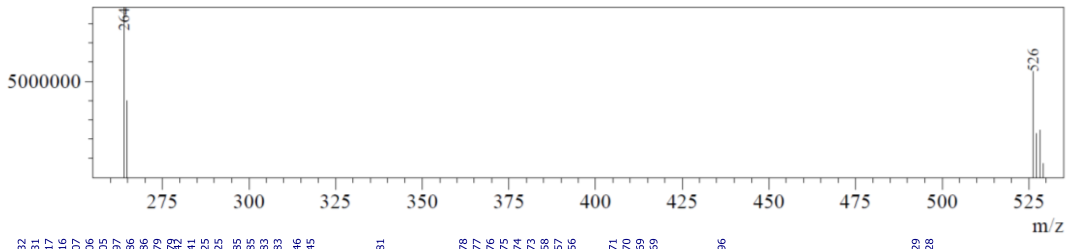

264
526
5000000
m/z

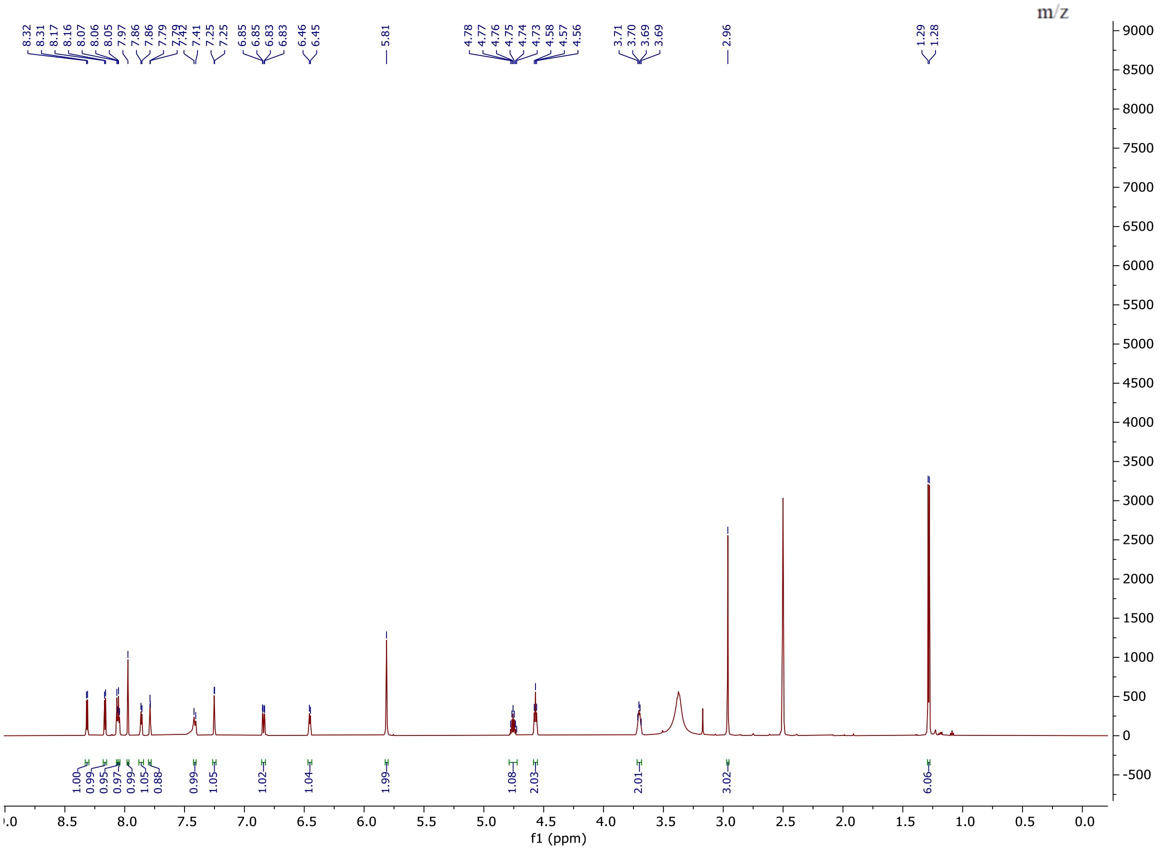

f1 (ppm)

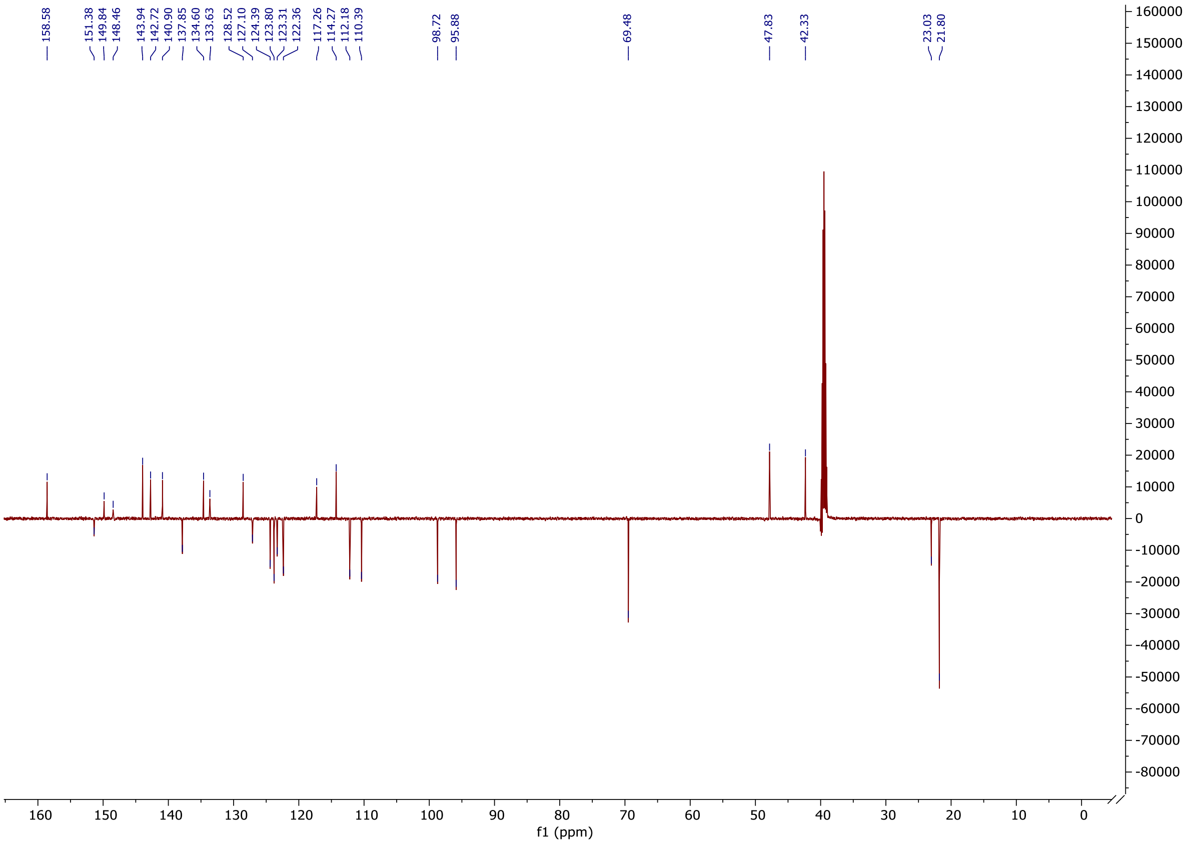
158.58
151.38
149.84
148.46
143.94
142.72
140.90
137.85
134.60
133.63
128.52
127.10
124.39
123.80
123.31
122.36
117.26
114.27
112.18
110.39
98.72
95.88
69.48
47.83
42.33
23.03
21.80
f1 (ppm)

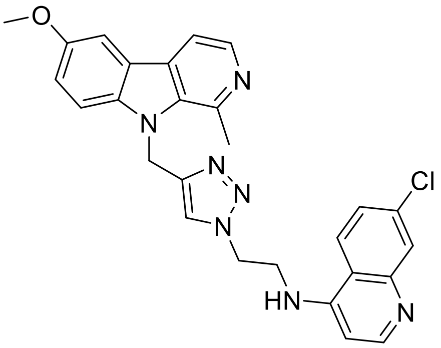
O
N
N
N
N
N
Cl
HN
N

41

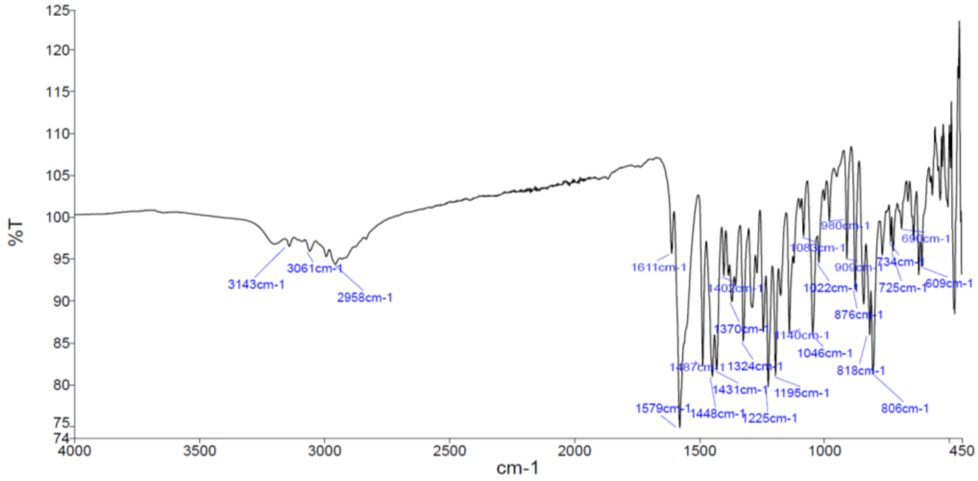
%T
cm-1
3143cm-1
3061cm-1
2958cm-1
1611cm-1
1579cm-1
1487cm-1
1448cm-1
1431cm-1
1402cm-1
1370cm-1
1324cm-1
1225cm-1
1195cm-1
1140cm-1
1083cm-1
1046cm-1
1022cm-1
980cm-1
909cm-1
876cm-1
818cm-1
806cm-1
734cm-1
725cm-1
690cm-1
609cm-1

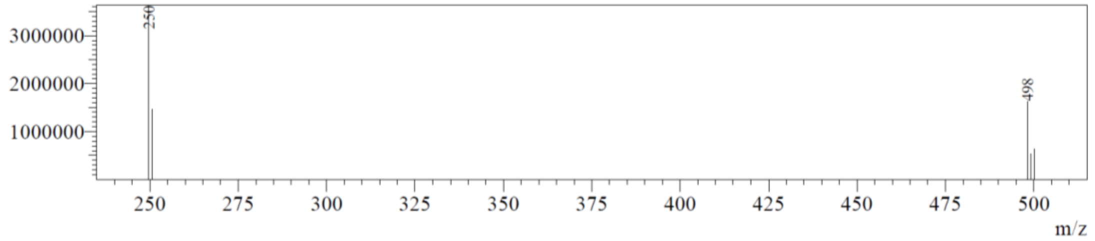
250
498
m/z

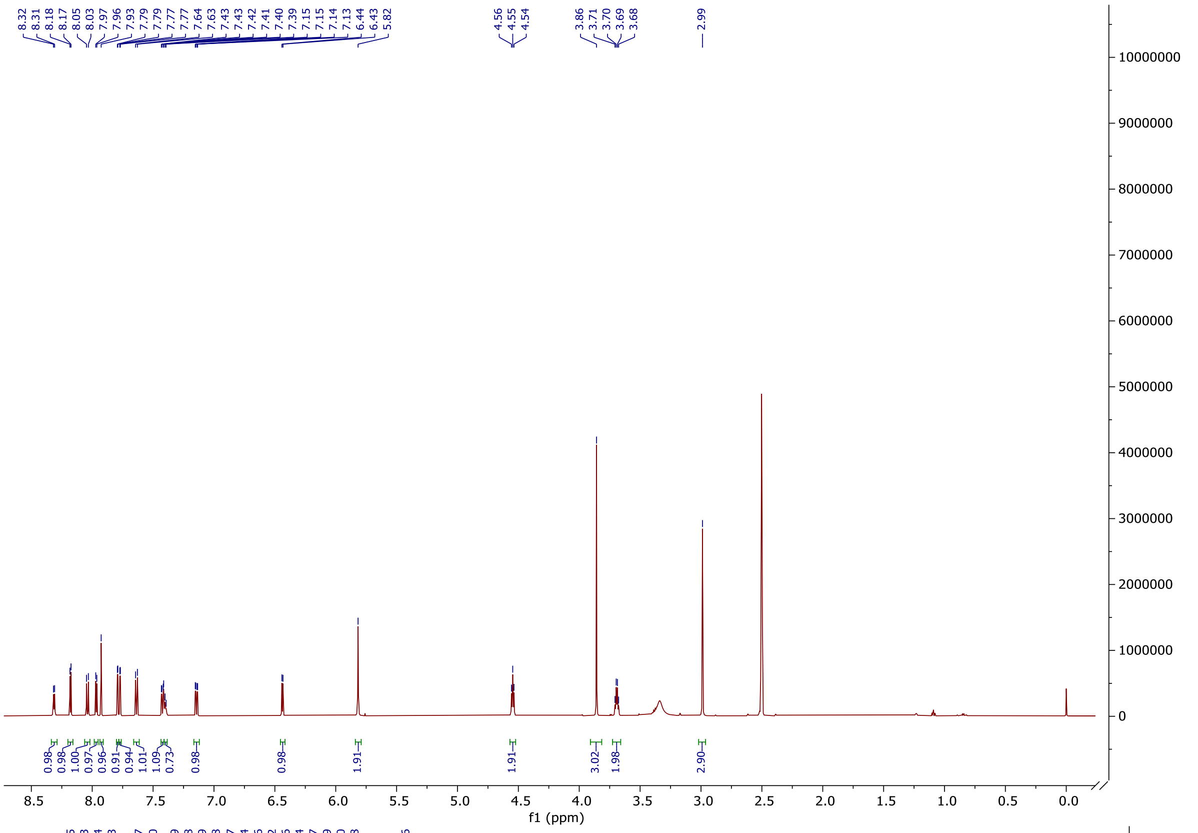

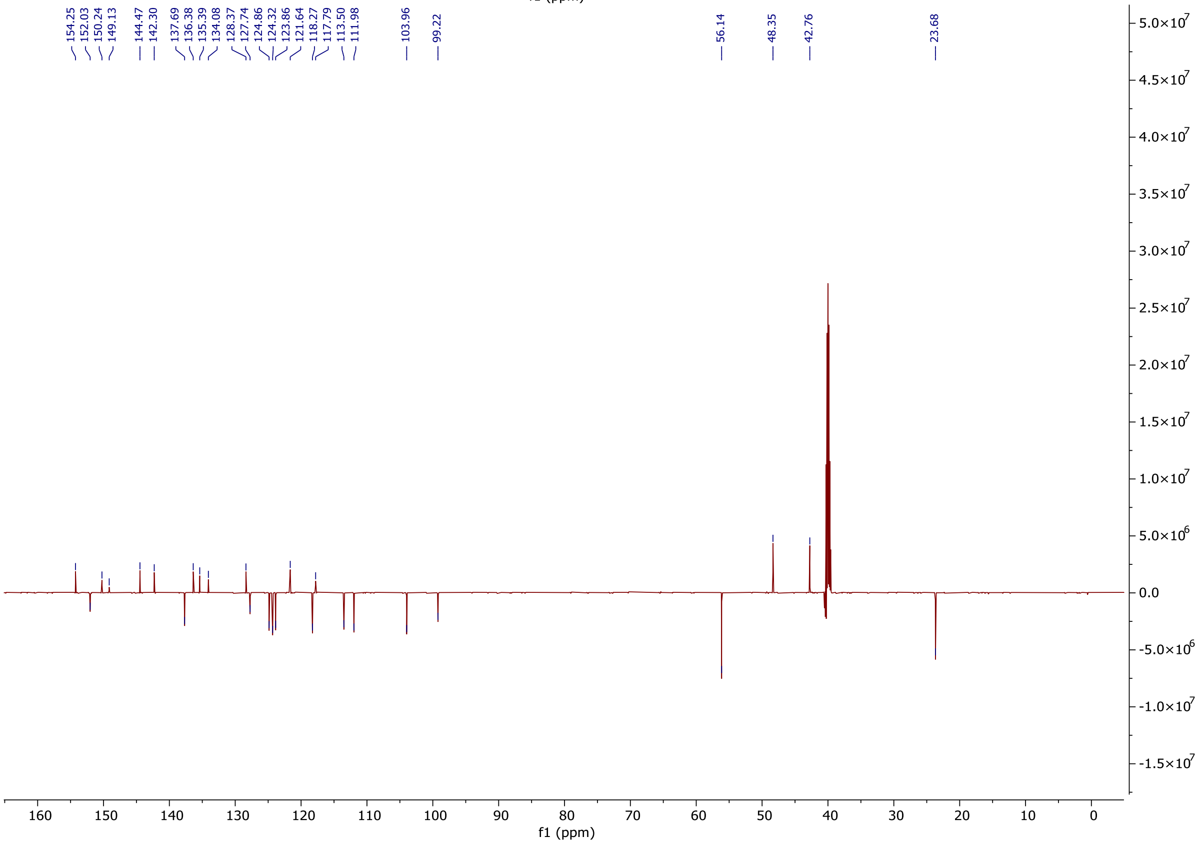

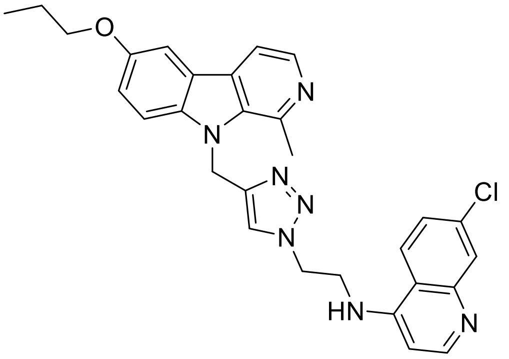

42

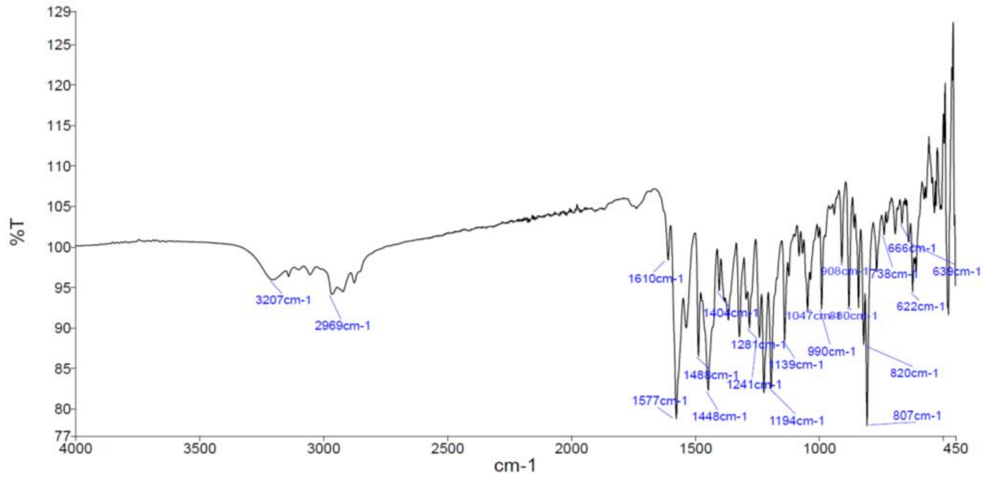
%T
cm-1
3207cm-1
2969cm-1
1610cm-1
1577cm-1
1488cm-1
1448cm-1
1404cm-1
1281cm-1
1241cm-1
1194cm-1
1139cm-1
1047cm-1
990cm-1
908cm-1
880cm-1
820cm-1
807cm-1
738cm-1
666cm-1
639cm-1
622cm-1

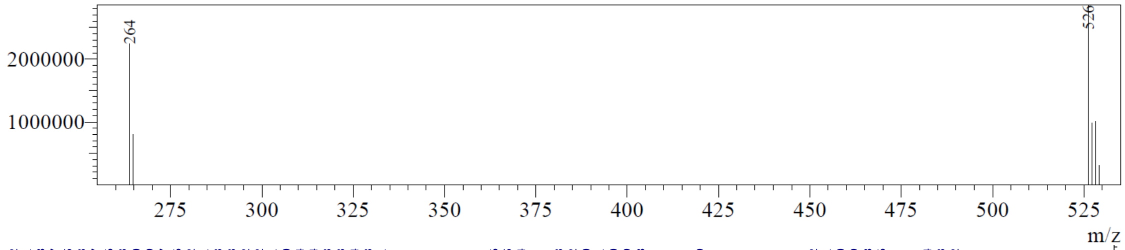
264
526
m/z

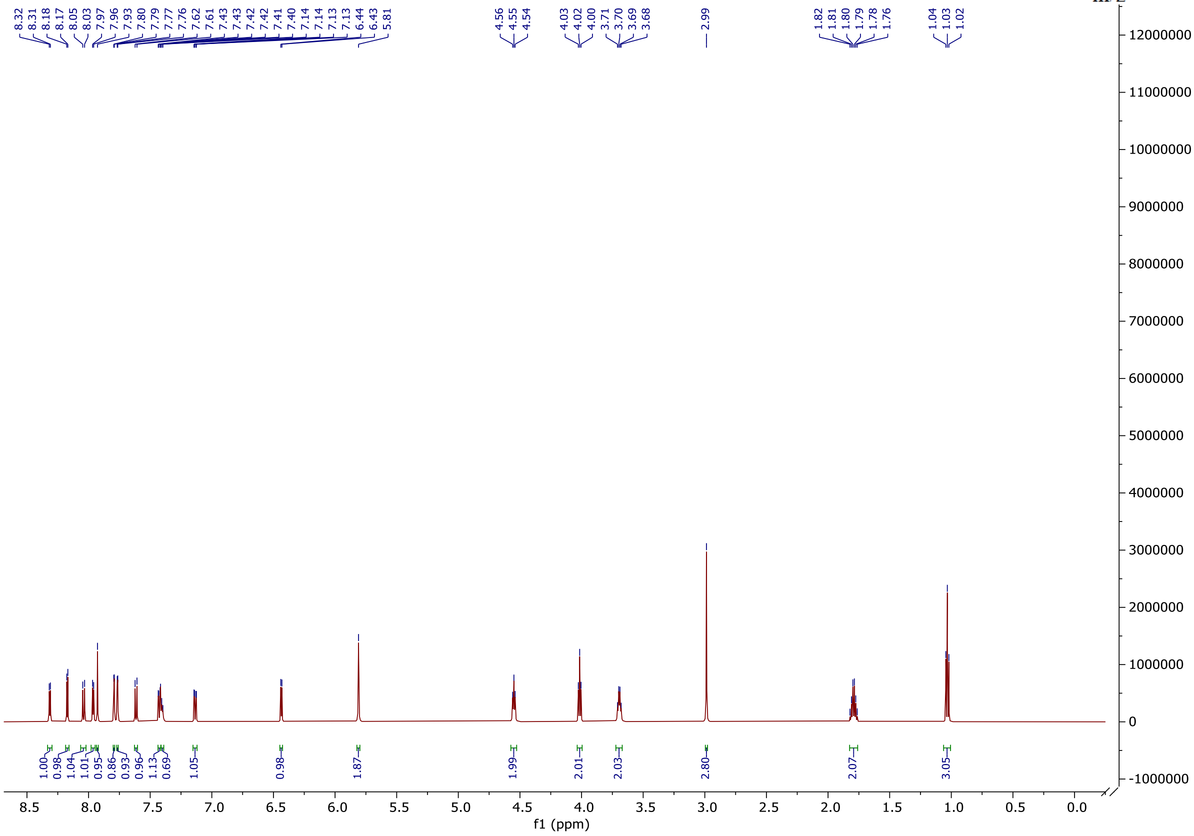
8.32
8.31
8.18
8.17
8.05
8.03
7.97
7.96
7.93
7.80
7.79
7.77
7.76
7.62
7.61
7.43
7.43
7.42
7.41
7.40
7.14
7.14
7.13
6.44
6.43
5.81
4.56
4.55
4.54
4.03
4.02
4.00
3.71
3.70
3.69
3.68
2.99
1.82
1.81
1.80
1.79
1.78
1.76
1.04
1.03
1.02
1.00
0.98
1.04
1.01
0.95
0.86
0.93
0.96
1.13
0.69
1.05
0.98
1.87
1.99
2.01
2.03
2.80
2.07
3.05
f1 (ppm)

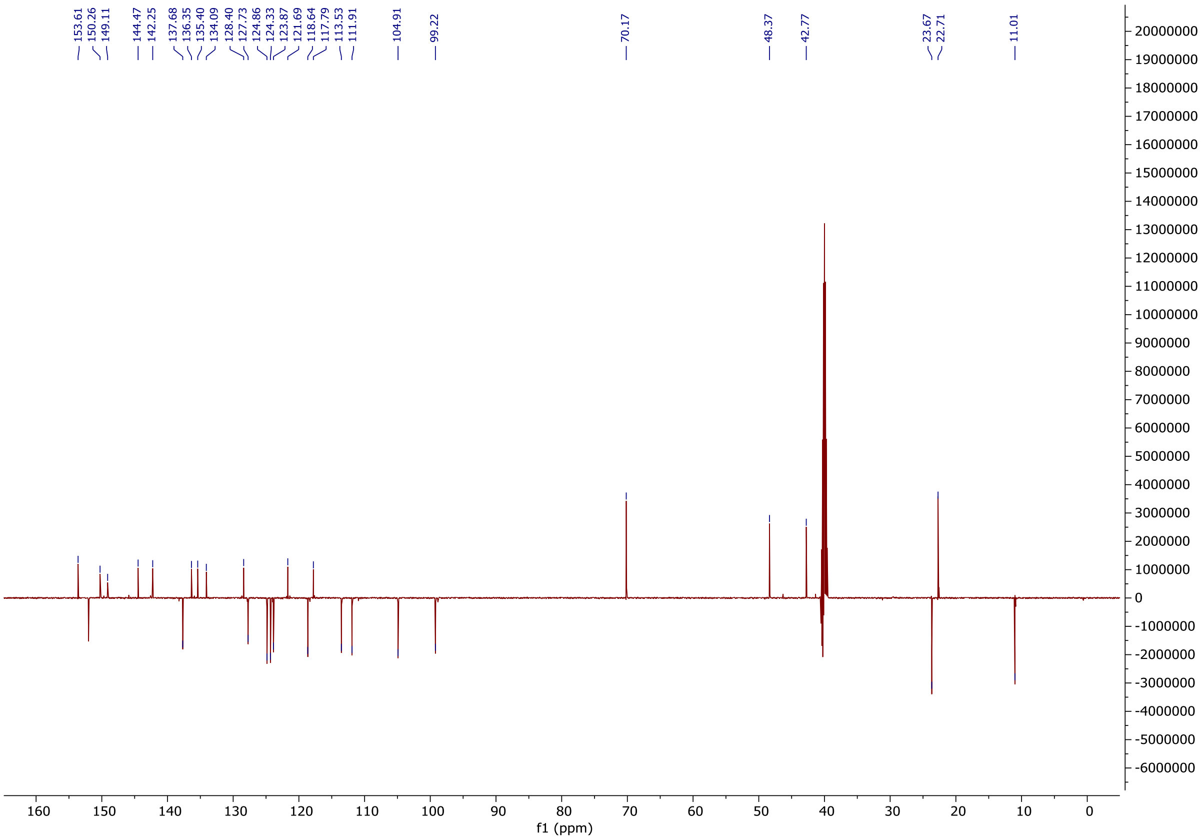
153.61
150.26
149.11
144.47
142.25
137.68
136.35
135.40
134.09
128.40
127.73
124.86
124.33
123.87
121.69
118.64
117.79
113.53
111.91
104.91
99.22
70.17
48.37
42.77
23.67
22.71
11.01
f1 (ppm)

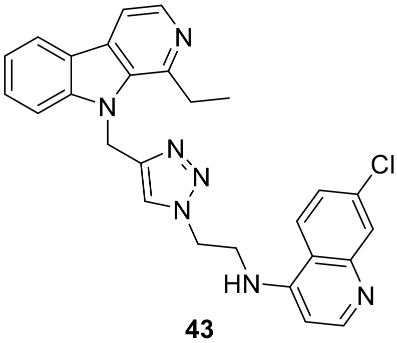
N
N
N
N
N
Cl
HN
N

43

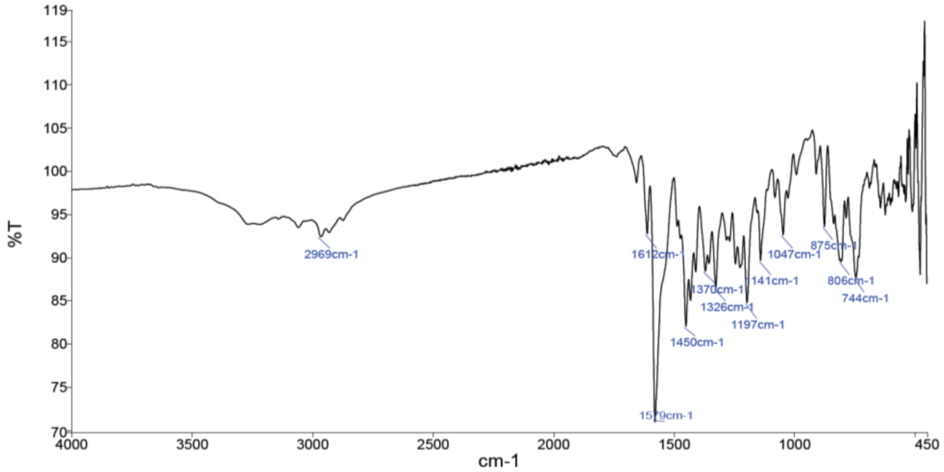
%T
cm-1
2969cm-1
1612cm-1
1579cm-1
1450cm-1
1370cm-1
1326cm-1
1197cm-1
141cm-1
1047cm-1
875cm-1
806cm-1
744cm-1

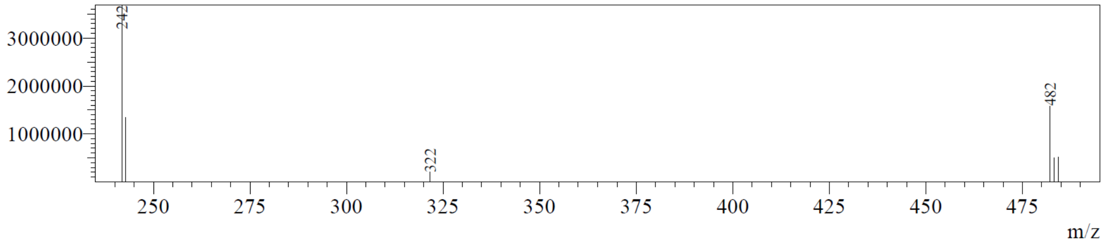
242
322
482
m/z

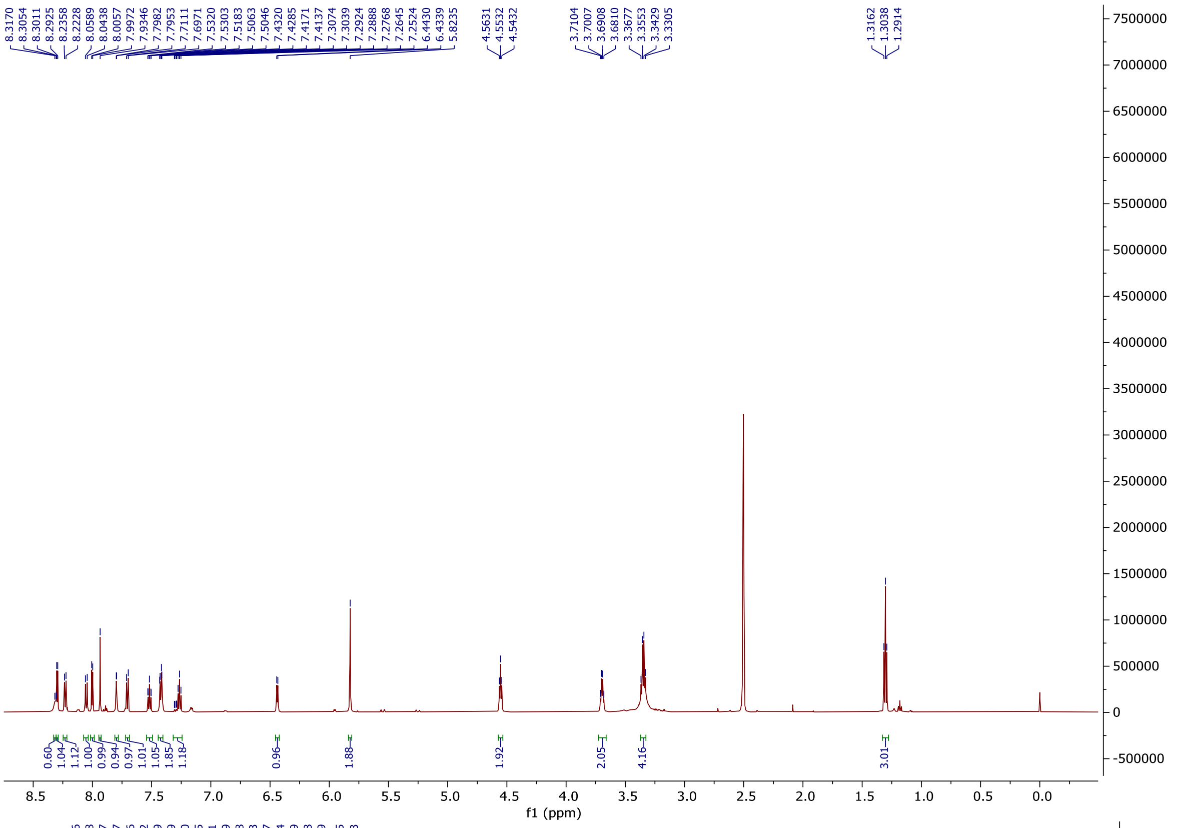

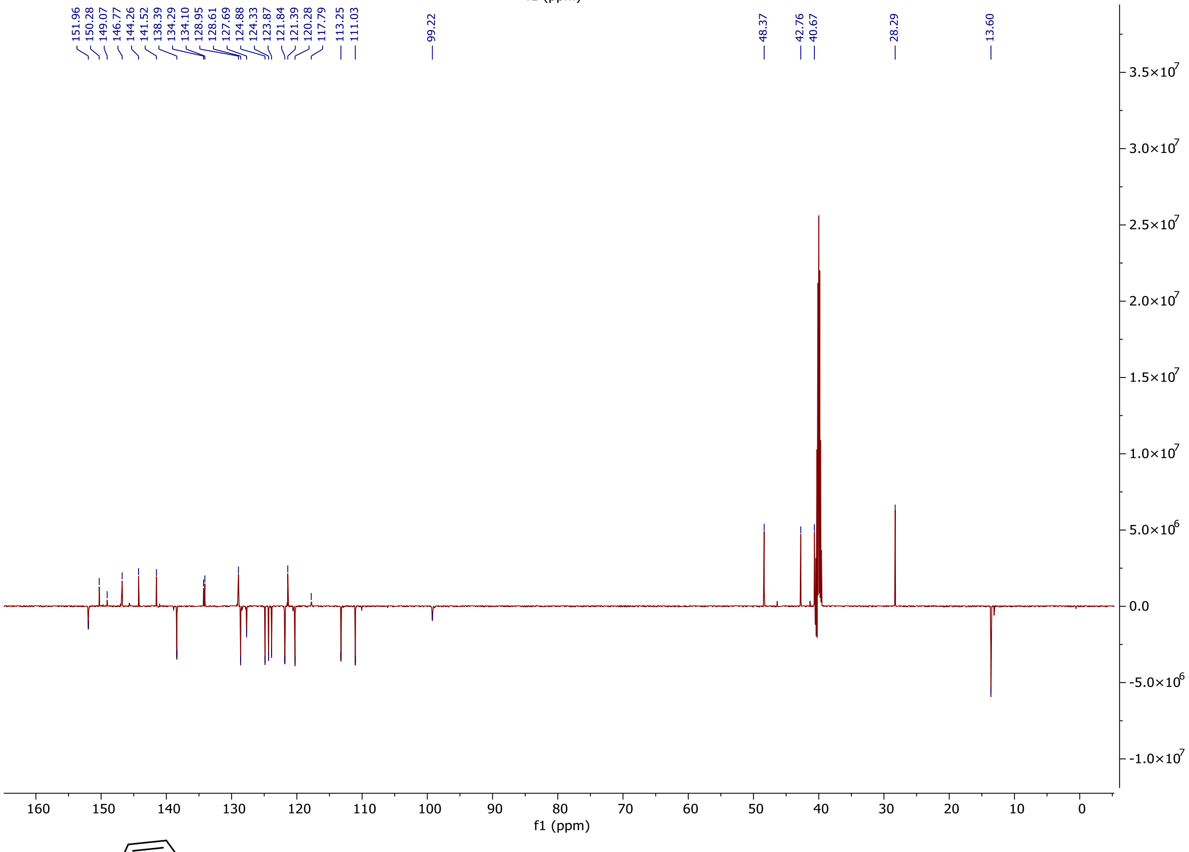

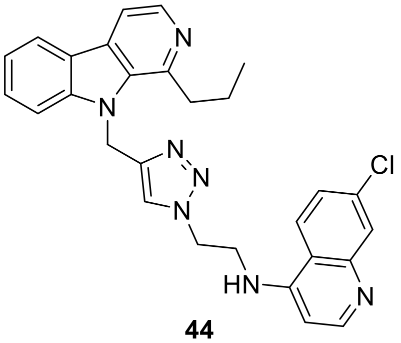

44

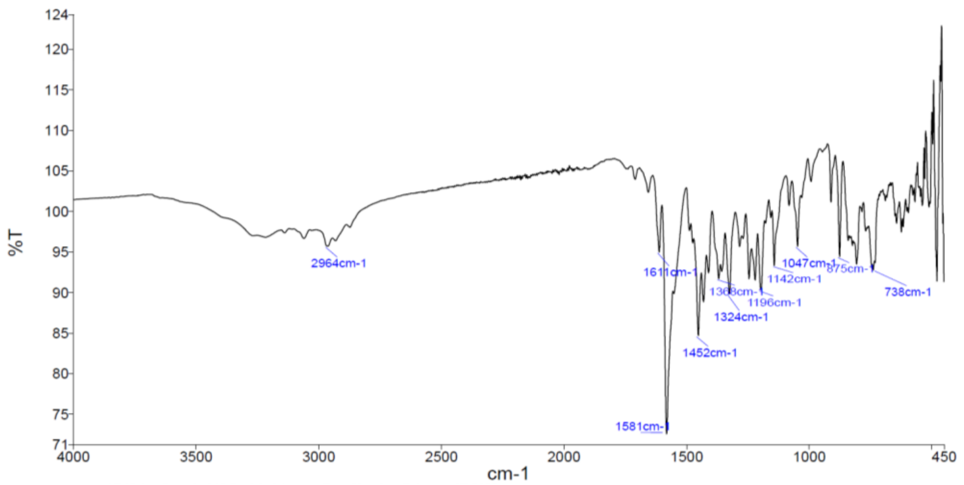

%T
cm-1
2964cm-1
1611cm-1
1581cm-1
1452cm-1
1368cm-1
1324cm-1
1196cm-1
1142cm-1
1047cm-1
875cm-1
738cm-1

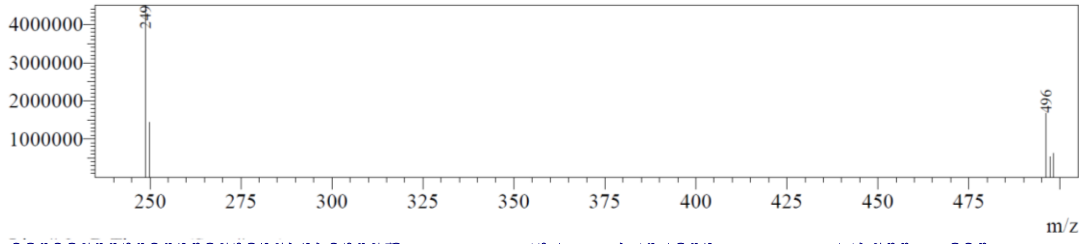

249
496
m/z

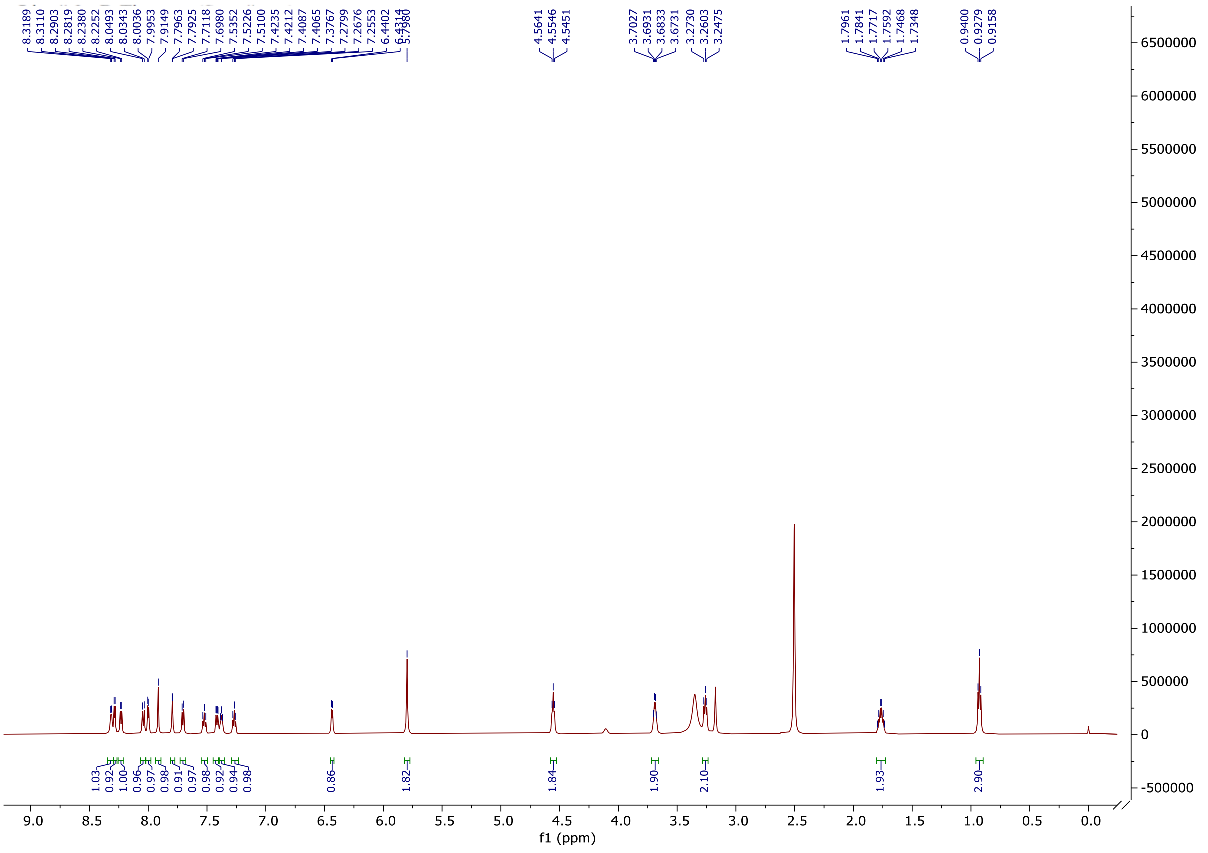

8.3189
8.3110
8.2903
8.2819
8.2380
8.2252
8.0493
8.0343
8.0036
7.9953
7.9149
7.7963
7.7925
7.7118
7.6980
7.5352
7.5226
7.5100
7.4235
7.4212
7.4087
7.4065
7.3767
7.2799
7.2676
7.2553
6.4402
6.4314
5.7980
4.5641
4.5546
4.5451
3.7027
3.6931
3.6833
3.6731
3.2730
3.2603
3.2475
1.7961
1.7841
1.7717
1.7592
1.7468
1.7348
0.9400
0.9279
0.9158
1.03
0.92
1.00
0.96
0.97
0.98
0.91
0.97
0.98
0.92
0.94
0.98
0.86
1.82
1.84
1.90
2.10
1.93
2.90
f1 (ppm)

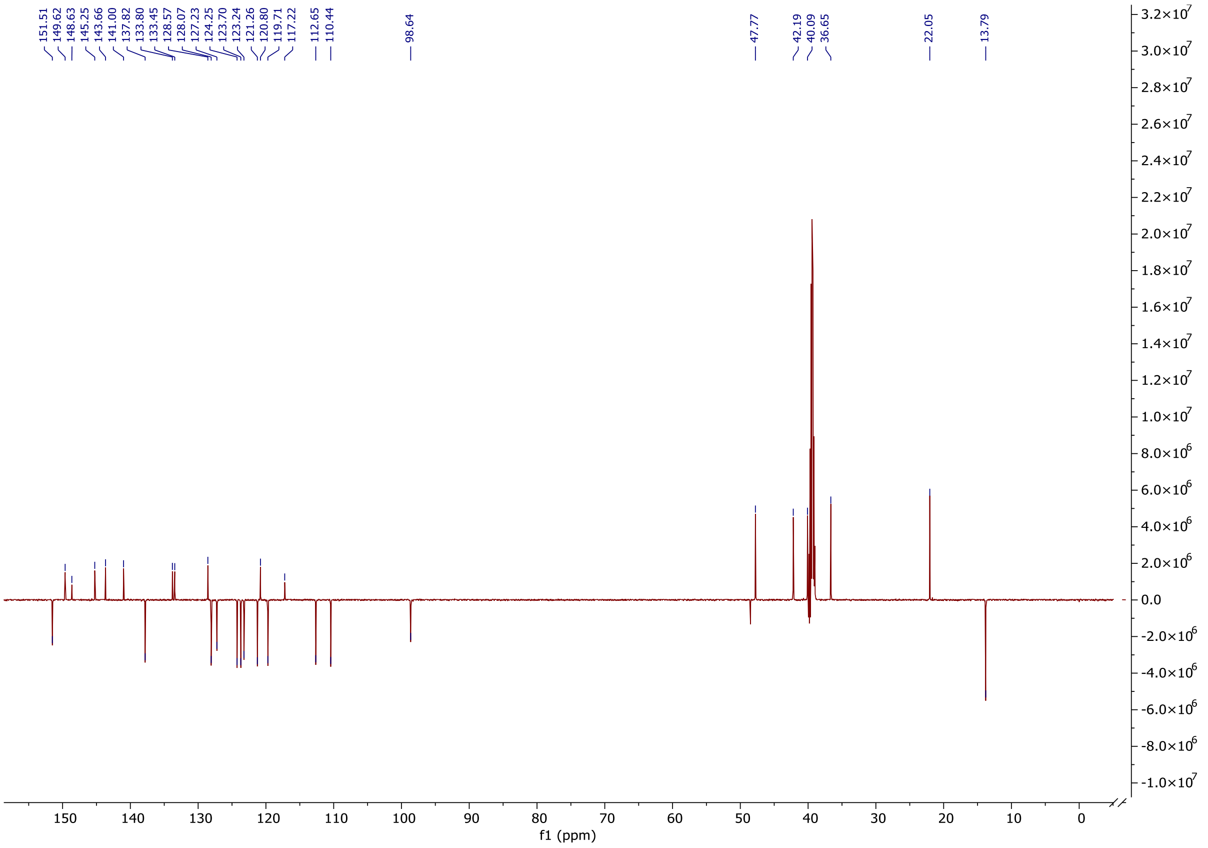
151.51
149.62
148.63
145.25
143.66
141.00
137.82
133.80
133.45
128.57
128.07
127.23
124.25
123.70
123.24
121.26
120.80
119.71
117.22
112.65
110.44
98.64
47.77
42.19
40.09
36.65
22.05
13.79
f1 (ppm)

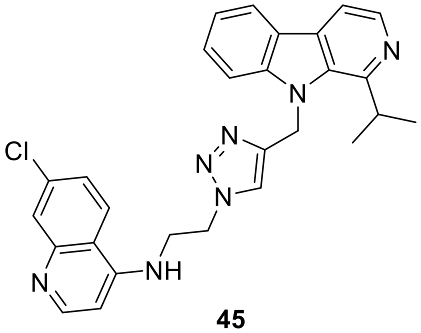

45

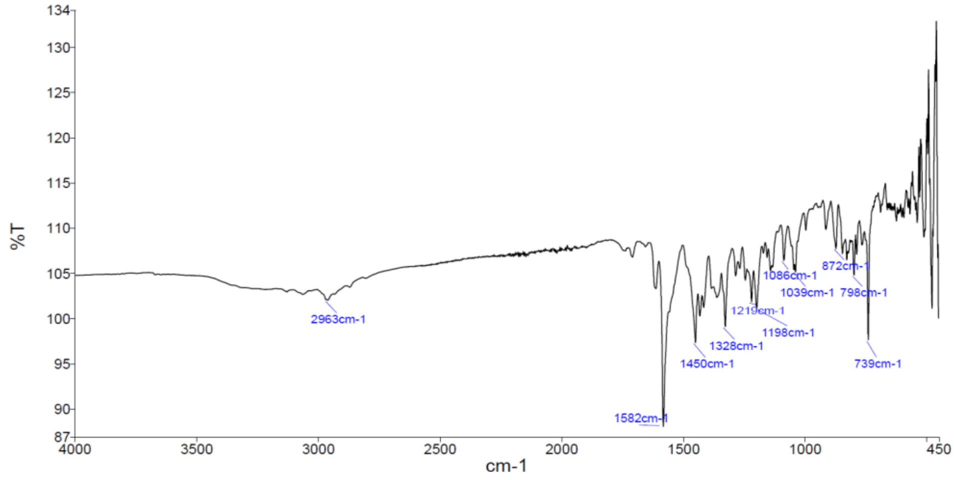
%T
cm-1
2963cm-1
1582cm-1
1450cm-1
1328cm-1
1219cm-1
1198cm-1
1086cm-1
1039cm-1
872cm-1
798cm-1
739cm-1

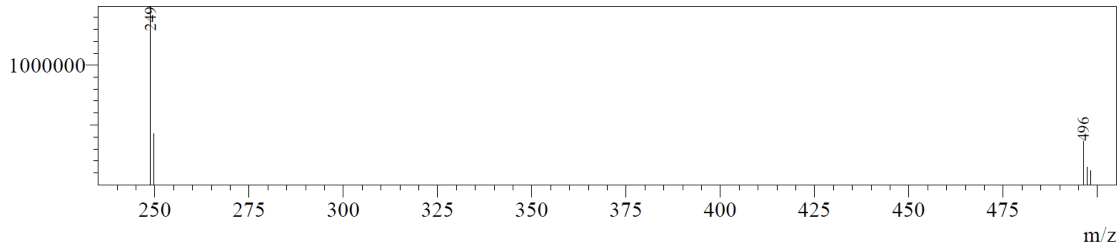
249
496
m/z

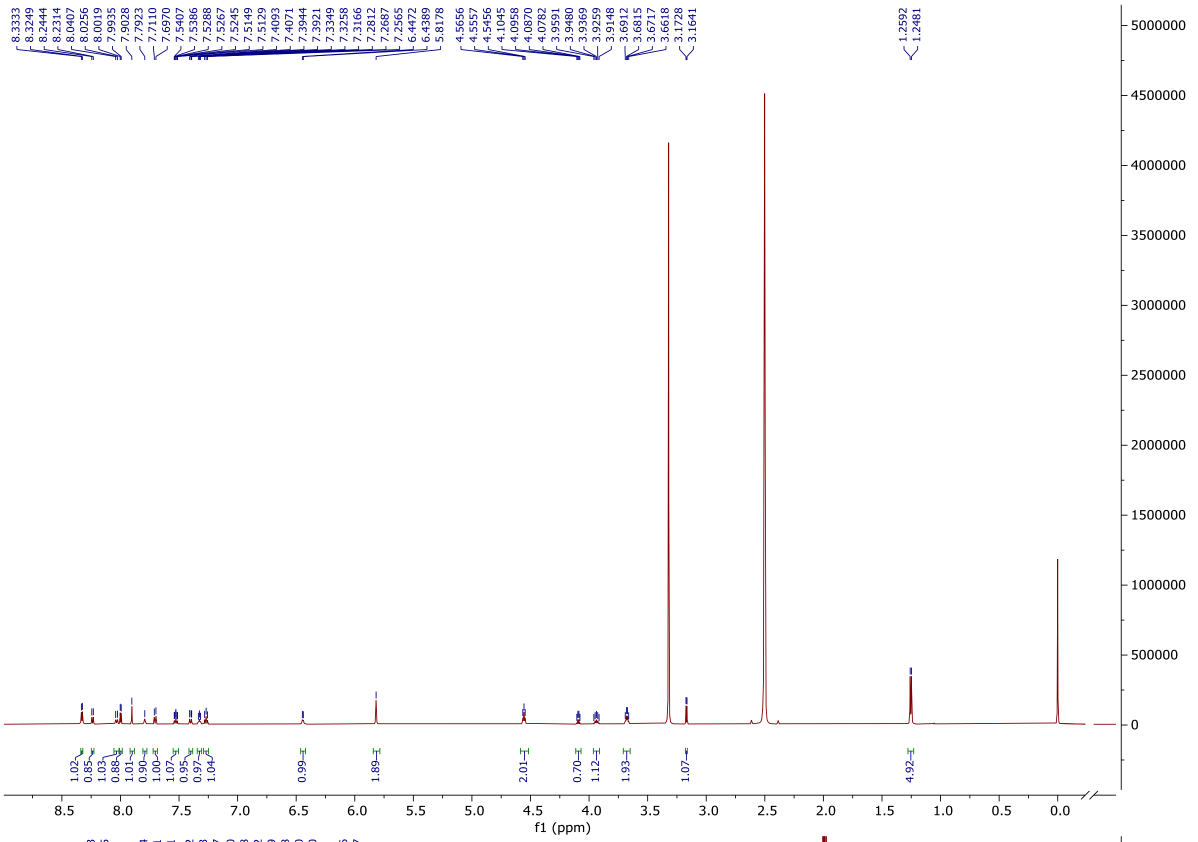
8.3333
8.3249
8.2444
8.2314
8.0407
8.0256
8.0019
7.9935
7.9028
7.7923
7.7110
7.6970
7.5407
7.5386
7.5288
7.5267
7.5245
7.5149
7.5129
7.4093
7.4071
7.3944
7.3921
7.3349
7.3258
7.3166
7.2812
7.2687
7.2565
6.4472
6.4389
5.8178
4.5656
4.5557
4.5456
4.1045
4.0958
4.0870
4.0782
3.9591
3.9480
3.9369
3.9259
3.9148
3.6912
3.6815
3.6717
3.6618
3.1728
3.1641
1.2592
1.2481
1.02
0.85
1.03
0.88
1.01
0.90
1.00
1.07
0.95
0.97
1.04
0.99
1.89
2.01
0.70
1.12
1.93
1.07
4.92
f1 (ppm)

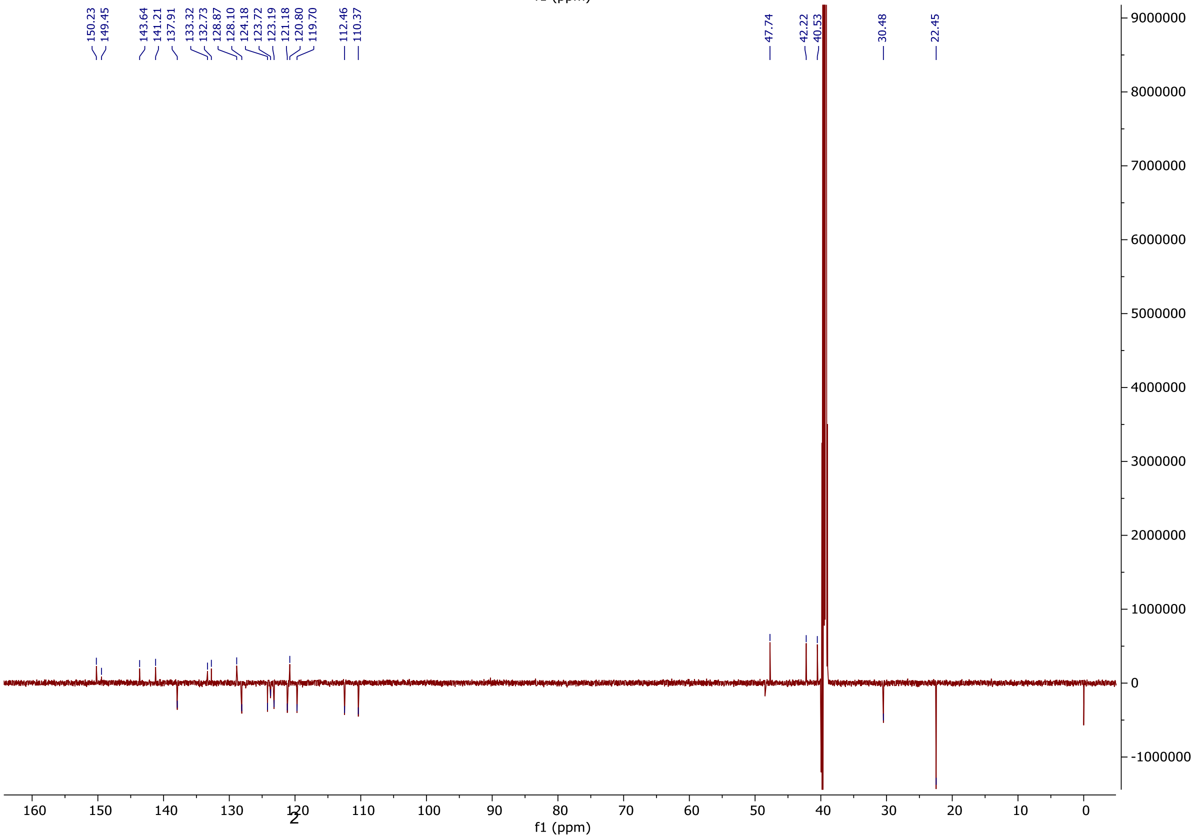
150.23
149.45
143.64
141.21
137.91
133.32
132.73
128.87
128.10
124.18
123.72
123.19
121.18
120.80
119.70
112.46
110.37
47.74
42.22
40.53
30.48
22.45
f1 (ppm)

3. Physico-chemical properties – spectroscopic experiments

3.1. Solubility

3.2. UV/Vis spectra

3.3. Fluorescence spectra

4. Study of interactions with double-stranded (ds-) DNA/RNA and HSA

4.1. Fluorescence titration experiments

4.2. Circular dichroism (CD) experiments

4.3. Thermal denaturation experiments

3. Physico-chemical properties

3.1. Solubility

Compounds (**34** and **39**) were dissolved in DMSO ($c = 1 \times 10^{-3}$ M).

3.2. UV/Vis spectra

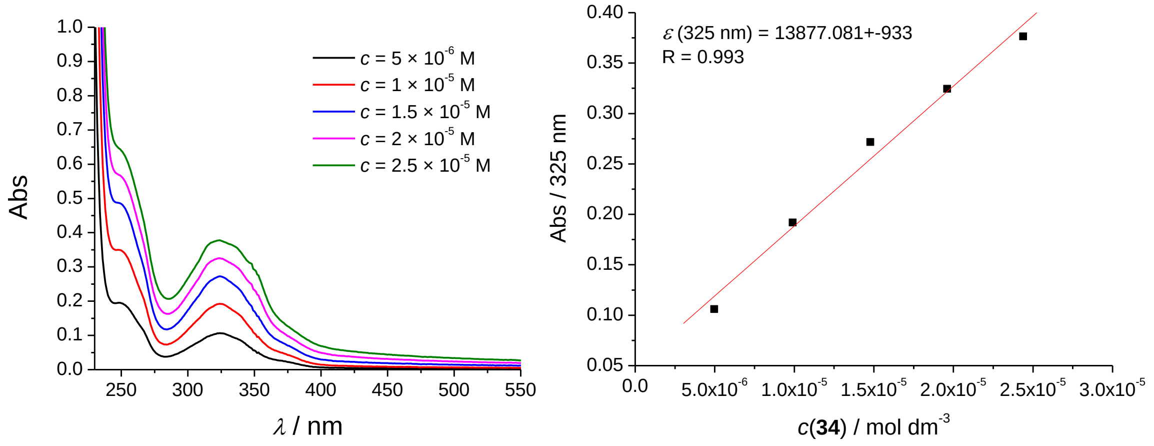

Figure S1. Dependence of the absorbance on the concentration of compound **34** (concentration range $5 \times 10^{-6} - 2.5 \times 10^{-5}$ M). Measured in sodium cacodylate buffer, pH 7, $I = 0.05$ M.

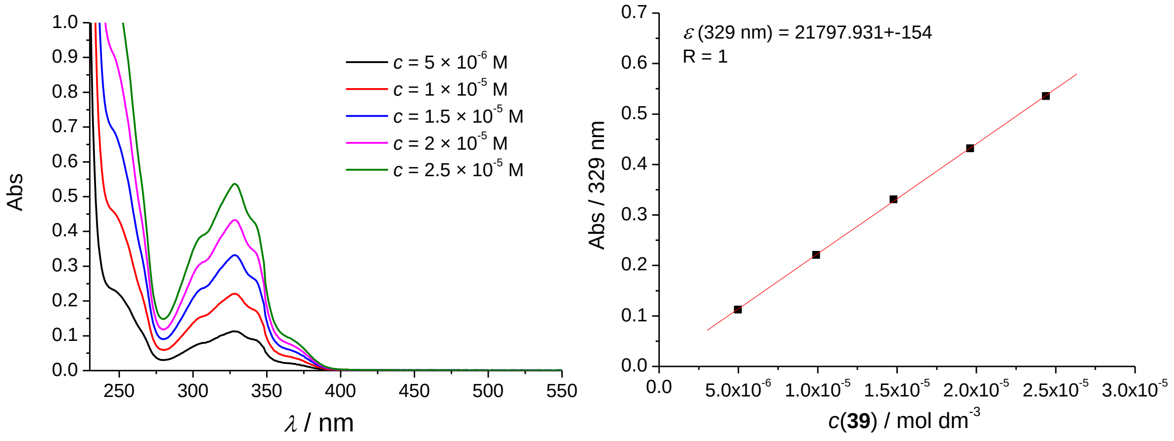

Figure S2. Dependence of the absorbance on the concentration of compound **39** (concentration range $5 \times 10^{-6} - 2.5 \times 10^{-5}$ M). Measured in sodium cacodylate buffer, pH 7, $I = 0.05$ M.

3.3. Fluorescence spectra

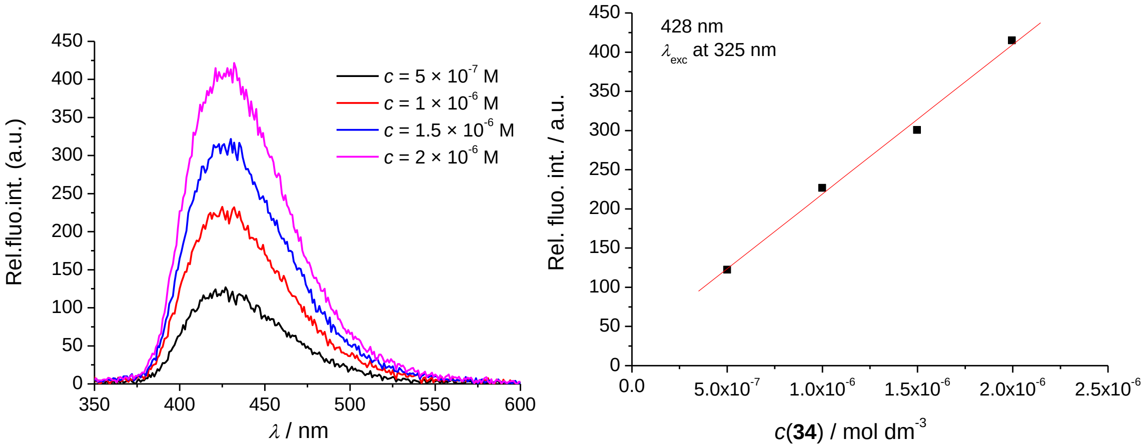

Figure S3. Dependence of the emission on the concentration of compound **34** (concentration range $5 \times 10^{-7} - 2 \times 10^{-6}$ M). Measured in sodium cacodylate buffer, pH 7, $I = 0.05$ M, $\lambda_{exc} = 325$ nm.

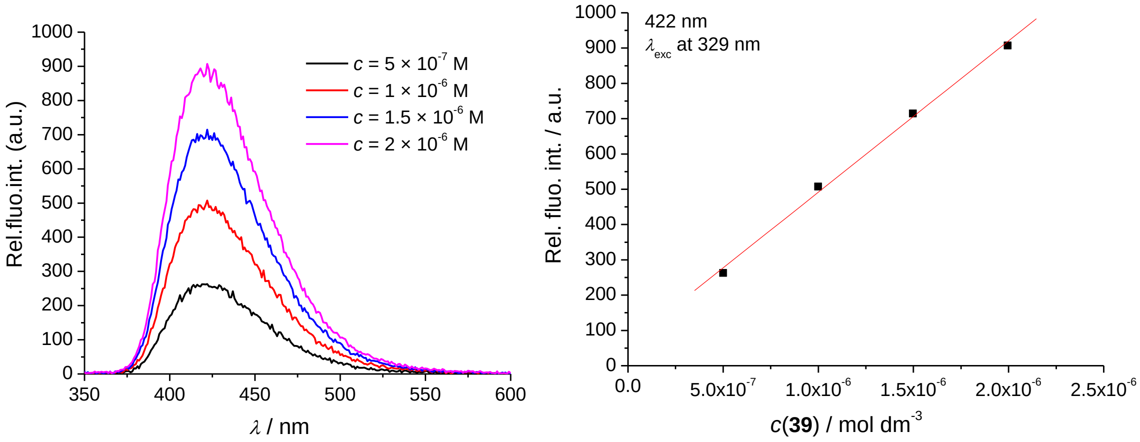

Figure S4. Dependence of the emission on the concentration of compound **39** (concentration range $5 \times 10^{-7} - 2 \times 10^{-6}$ M). Measured in sodium cacodylate buffer, pH 7, $I = 0.05$ M, $\lambda_{exc} = 329$ nm.

4. Study of interactions with double-stranded (ds-) DNA/RNA (ctDNA, pApU, p(dA-dT)$_2$ and p(dG-dC)$_2$), and HAS in buffered solution (sodium cacodylate, pH = 7, *I* = 0.05 M)

Table S13. Structural properties of studied ds-DNA and ds-RNA.[1,2]

Structure type	Groove width [Å]		Groove depth [Å]	
	major	minor	major	minor
[a] poly rA – poly rU	3.8	10.9	13.5	2.8
[b] ct-DNA (48% of GC-pairs)	11.4	3.3	7.5	7.9
[b] poly dAdT – poly dAdT	11.2	6.3	8.5	7.5
[c] poly dGdC – poly dGdC	13.5	9.5	10.0	7.2

[a] A - helical structure

[b] B - helical structure

[c] B - helical structure with sterically blocked minor groove by amino groups of guanines

4.1. Fluorescence titration experiments

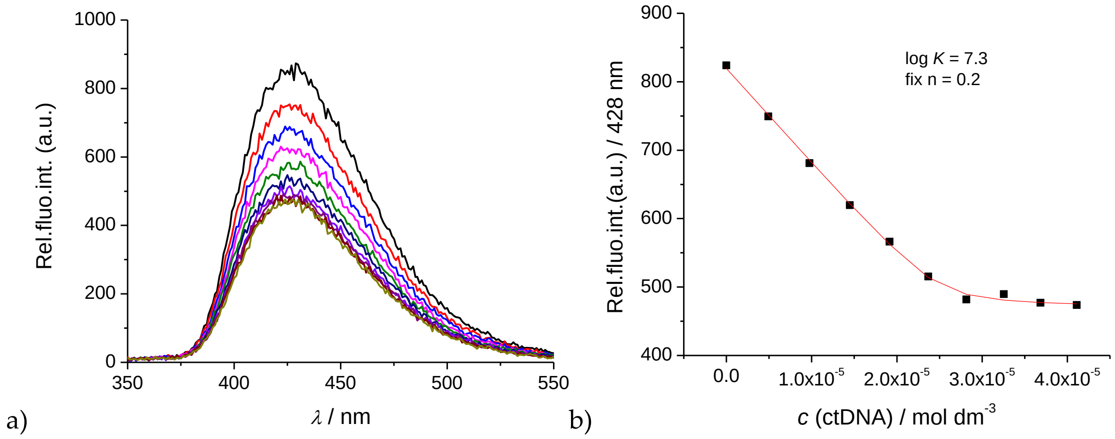

Figure S5. a) Changes in the fluorescence spectrum of **34** ($c = 5 \times 10^{-6}$ M, λ_{exc} = 325 nm) upon titration with **ctDNA** ($c = 5 \times 10^{-6} – 4.1 \times 10^{-5}$ M), slit: 5-10; b) Dependence of **34** emission at λ_{max} = 428 nm on *c*(ctDNA), at pH 7.0, sodium cacodylate buffer, *I* = 0.05 M.

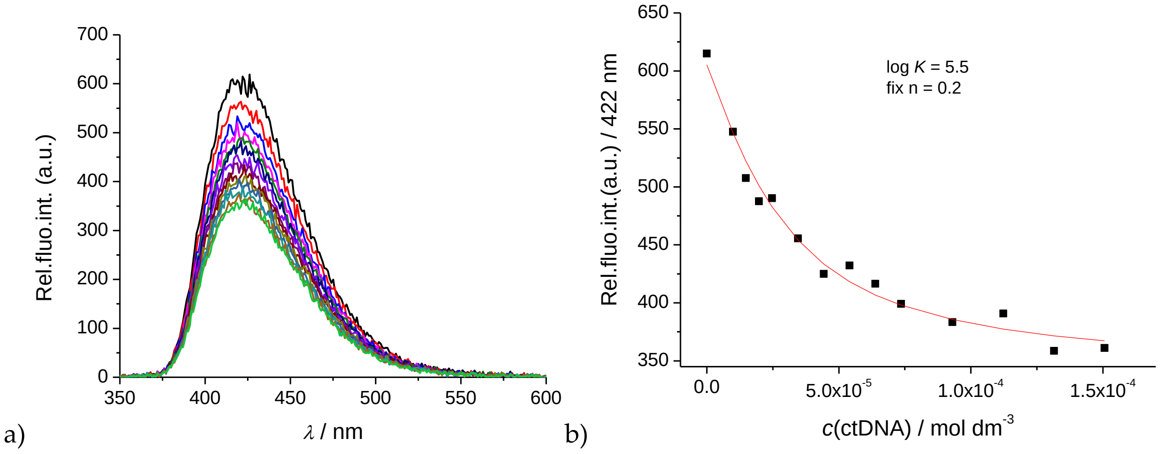

Figure S6. a) Changes in the fluorescence spectrum of **39** ($c = 5 \times 10^{-6}$ M, λ_{exc} = 329 nm) upon titration with **ctDNA** ($c = 5 \times 10^{-6} – 1.5 \times 10^{-4}$ M), slit: 5-5; b) Dependence of **39** emission at λ_{max} = 422 nm on *c*(ctDNA), at pH 7.0, sodium cacodylate buffer, *I* = 0.05 M.

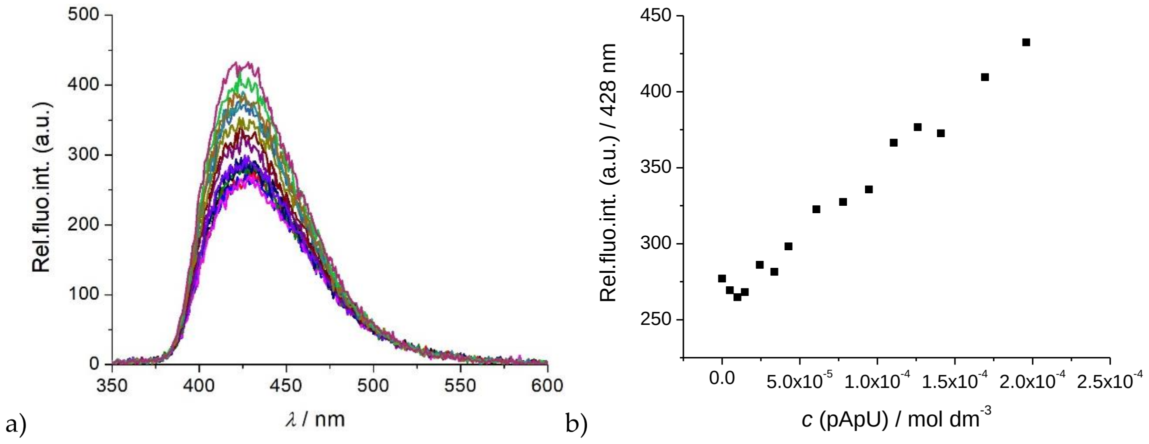

Figure S7. a) Changes in the fluorescence spectrum of **34** ($c = 5 \times 10^{-6}$ M, $\lambda_{exc} = 325$ nm) upon titration with **poly A – poly U** ($c = 5 \times 10^{-6} - 2 \times 10^{-4}$ M), slit: 5-5; b) Dependence of **34** emission at $\lambda_{max} = 428$ nm on c(pApU), at pH 7.0, sodium cacodylate buffer, $I = 0.05$ M.

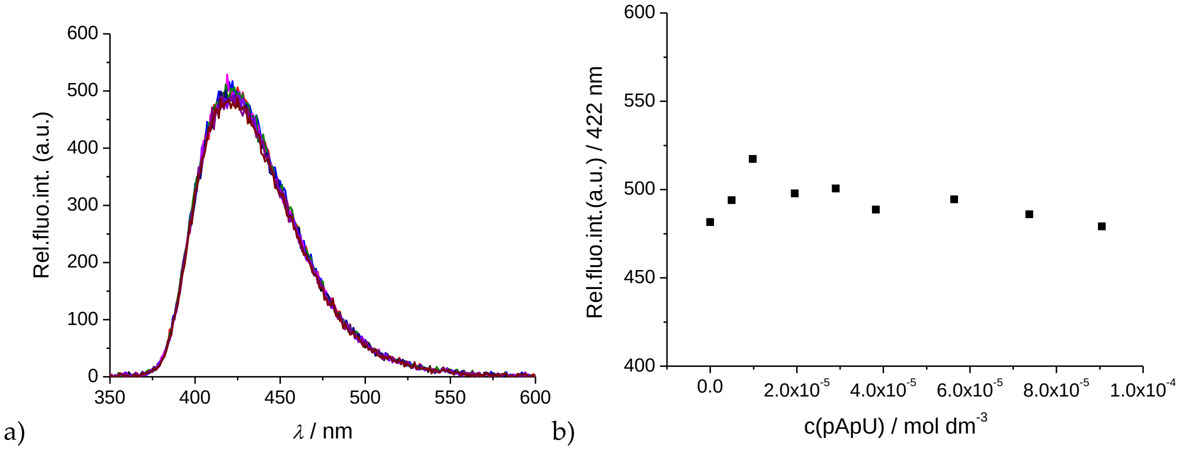

Figure S8. a) Changes in the fluorescence spectrum of **39** ($c = 5 \times 10^{-6}$ M, $\lambda_{exc} = 329$ nm) upon titration with **poly A – poly U** ($c = 5 \times 10^{-6} - 9 \times 10^{-5}$ M), slit: 5-5; b) Dependence of **39** emission at $\lambda_{max} = 422$ nm on c(pApU), at pH 7.0, sodium cacodylate buffer, $I = 0.05$ M.

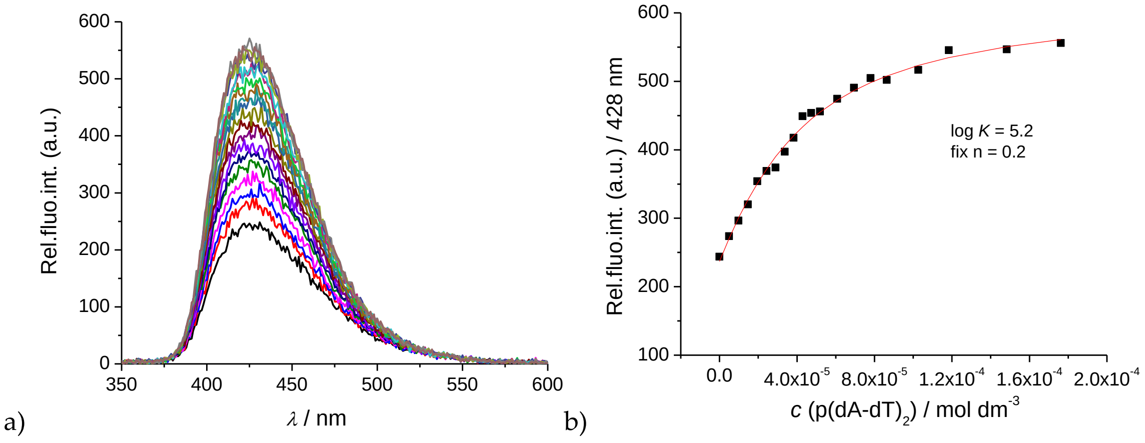

Figure S9. a) Changes in the fluorescence spectrum of **34** ($c = 5 \times 10^{-6}$ M, $\lambda_{exc} = 325$ nm) upon titration with **poly (dAdT) – poly (dAdT)** ($c = 5 \times 10^{-6} - 1.8 \times 10^{-4}$ M), slit: 5-5; b) Dependence of **34** emission at $\lambda_{max} = 428$ nm on c(p(dA-dT)$_2$), at pH 7.0, sodium cacodylate buffer, $I = 0.05$ M.

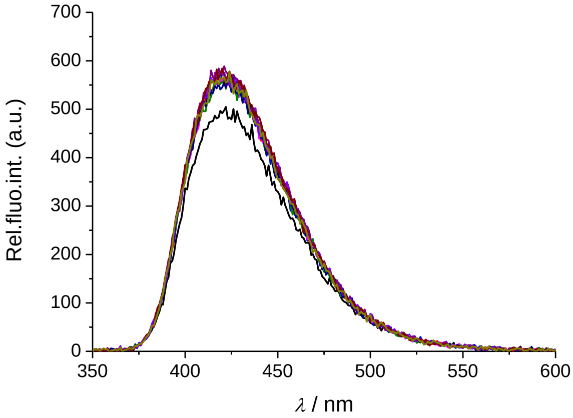

a)

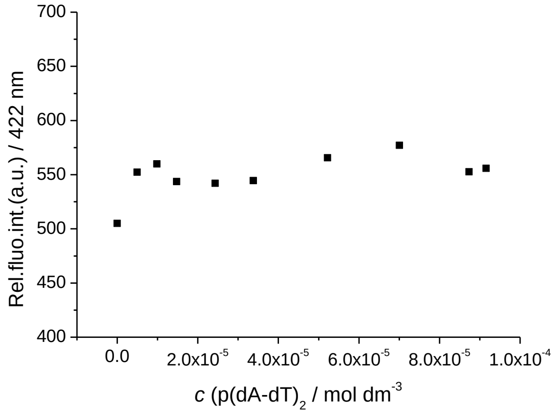

b)

Figure S10. a) Changes in the fluorescence spectrum of **39** (c = 5 × 10^{-6} M, λ_{exc} = 329 nm) upon titration with **poly (dAdT) – poly (dAdT)** (c = 5 × 10^{-6} – 9.2 × 10^{-5} M), slit: 5-5; b) Dependence of **39** emission at λ_{max} = 422 nm on c(p(dA-dT)$_2$), at pH 7.0, sodium cacodylate buffer, I = 0.05 M.

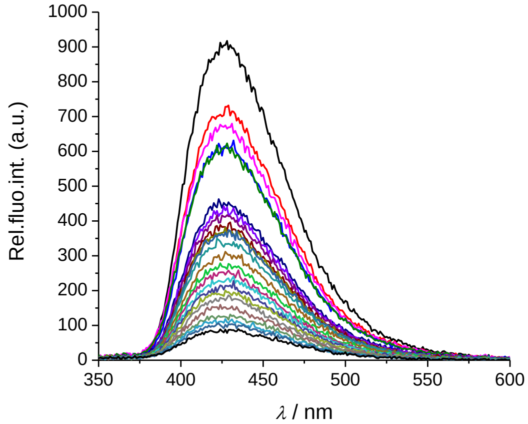

a)

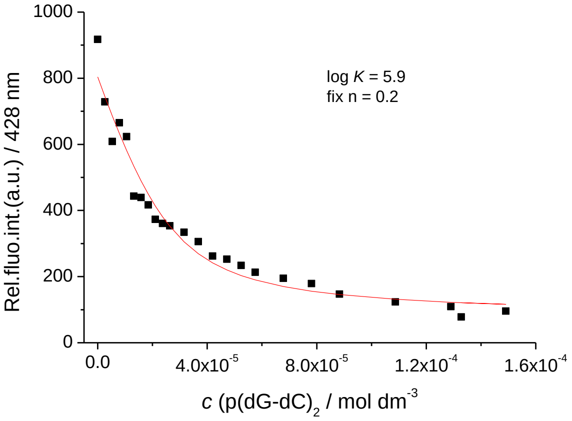

b)

Figure S11. a) Changes in the fluorescence spectrum of **34** (c = 5 × 10^{-6} M, λ_{exc} = 325 nm) upon titration with **poly (dGdC) – poly (dGdC)** (c = 5 × 10^{-6} – 2.5 × 10^{-4} M), slit: 5-10; b) Dependence of **34** emission at λ_{max} = 428 nm on c(p(dG-dC)$_2$), at pH 7.0, sodium cacodylate buffer, I = 0.05 M.

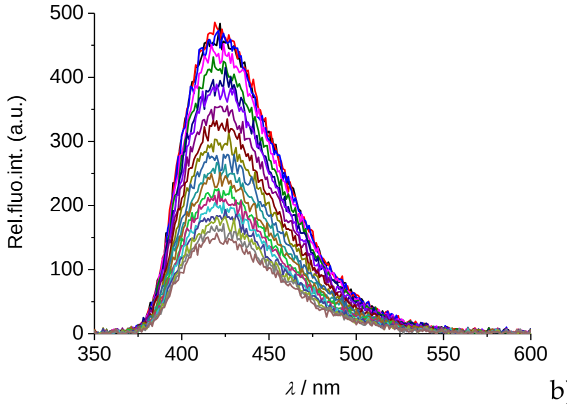

a)

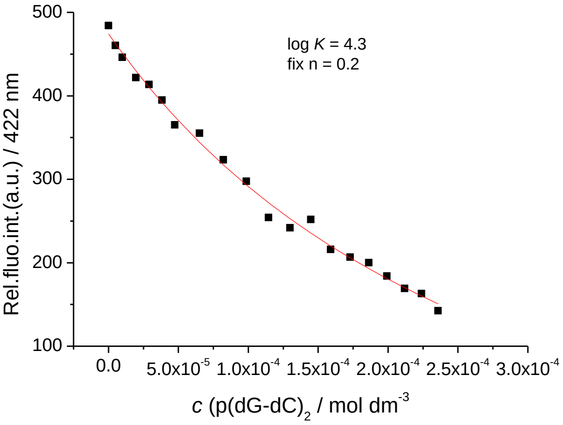

b)

Figure S12. a) Changes in the fluorescence spectrum of **39** (c = 5 × 10^{-6} M, λ_{exc} = 329 nm) upon titration with **poly (dGdC) – poly (dGdC)** (c = 5 × 10^{-6} – 2.4 × 10^{-4} M), slit: 5-5; b) Dependence of **39** emission at λ_{max} = 422 nm on c(p(dG-dC)$_2$), at pH 7.0, sodium cacodylate buffer, I = 0.05 M.

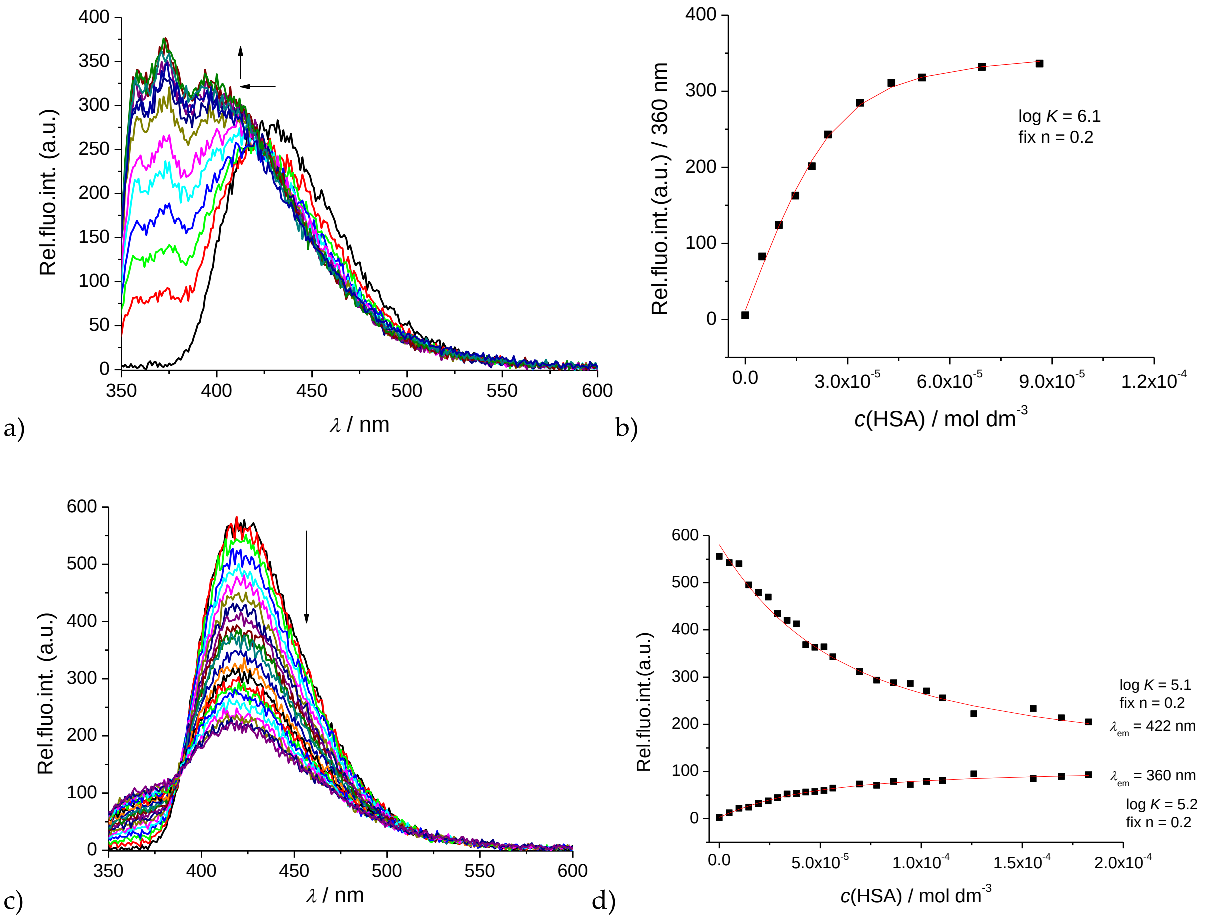

Figure S13. a) Changes in the fluorescence spectrum of **34** ($c = 5 \times 10^{-6}$ M, λ_{exc}= 325 nm) upon titration with **HSA** ($c = 5 \times 10^{-6} - 1.5 \times 10^{-4}$ M), slit: 5-5; b) Dependence of **34** emission at λ_{max} = 360 nm on c(HSA); c) Changes in the fluorescence spectrum of **39** ($c = 5 \times 10^{-6}$ M, λ_{exc}= 329 nm) upon titration with **HSA** ($c = 5 \times 10^{-6} - 1.8 \times 10^{-4}$ M), slit: 5-5; d) Dependence of **39** emission at λ_{max} = 360 and 422 nm on c(HSA), at pH 7.0, sodium cacodylate buffer, I = 0.05 M.

4.2. Circular dichroism (CD) experiments

So far, non-covalent interactions at 25 °C were studied by monitoring the spectroscopic properties of the studied compound upon the addition of the polynucleotides. To get insight into the changes in polynucleotide properties induced by small molecule binding, we have chosen CD spectroscopy as highly sensitive method toward conformational changes in the secondary structure of polynucleotides.[3] In addition, achiral small molecules can eventually acquire induced CD spectrum (ICD) upon binding to polynucleotides, which could give useful information about modes of interaction.[3] It should be noted that studied compounds are achiral and therefore do not possess intrinsic CD spectrum.

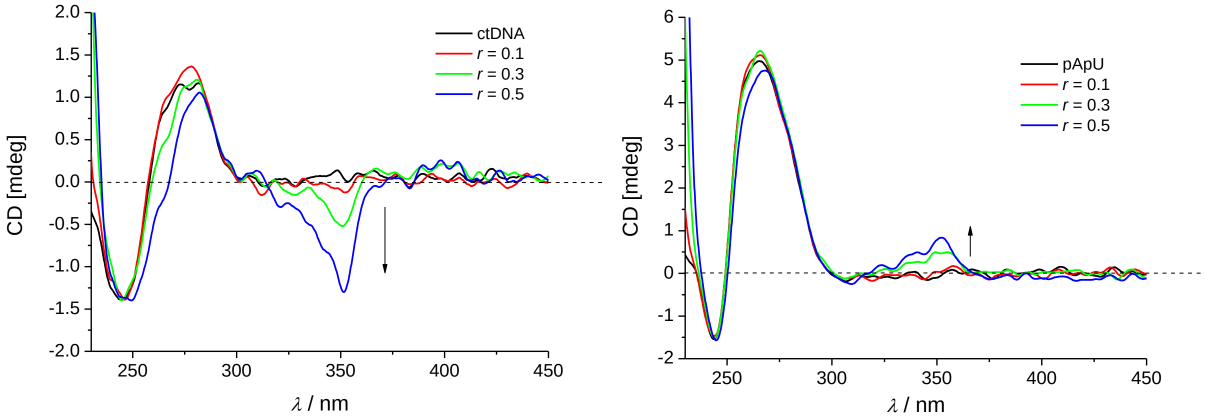

Figure S14. CD titration of **ctDNA** (c = 2 × 10^{-5} M), **poly A – poly U** (c = 2 × 10^{-5} M) with **34** at molar ratio r = [compound] / [polynucleotide] (pH 7.0, buffer sodium cacodylate, I = 0.05 M).

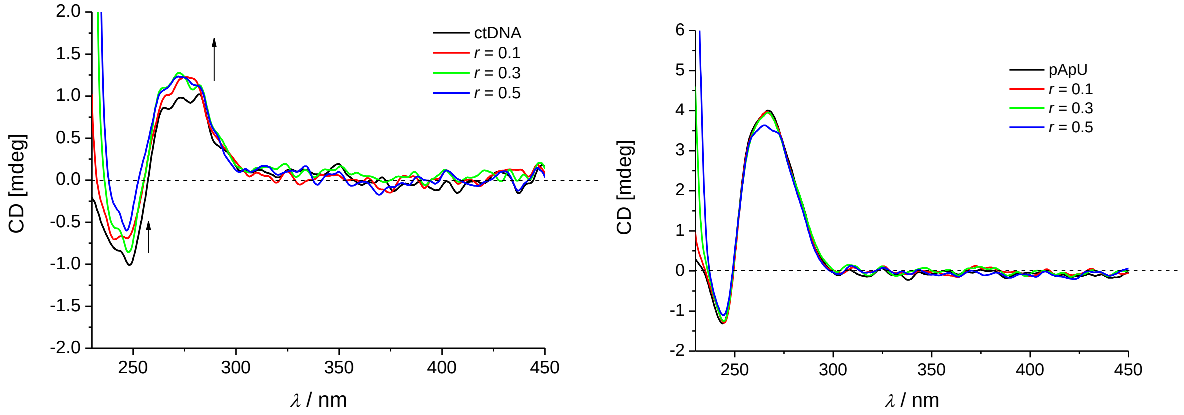

Figure S15. CD titration of **ctDNA** (c = 3 × 10^{-5} M) and **poly A – poly U** (c = 2 × 10^{-5} M), with **39** at molar ratio r = [compound] / [polynucleotide] (pH 7.0, buffer sodium cacodylate, I = 0.05 M).

4.3. Thermal denaturation experiments

It is well known that upon heating ds-helices of polynucleotides at well-defined temperature (T_m value) dissociate into two single-stranded polynucleotides. Non-covalent binding of small molecules to ds-polynucleotides usually has a certain effect on the thermal stability of helices thus giving different T_m values. The difference between the T_m value of free polynucleotide and the complex with a small molecule (ΔT_m value) is an important factor in the characterization of small molecule/ds-polynucleotide interactions.

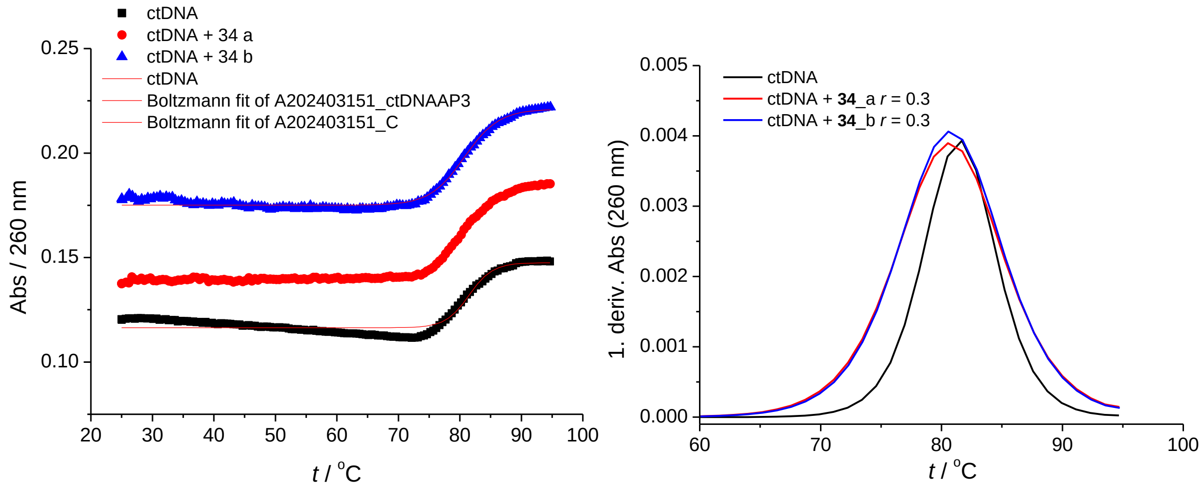

Figure S16. a) Denaturation of **ctDNA** upon addition of r = 0.3 ([compound/ [polynucleotide]) of **34** at pH 7.0 (buffer sodium cacodylate, I = 0.05 M), red lines denote fitting of experimental data to sigmoidal eq. by Origin 7.5; b) The first derivation of absorbance (fitted to sigmoidal eq.) on temperature dependence.

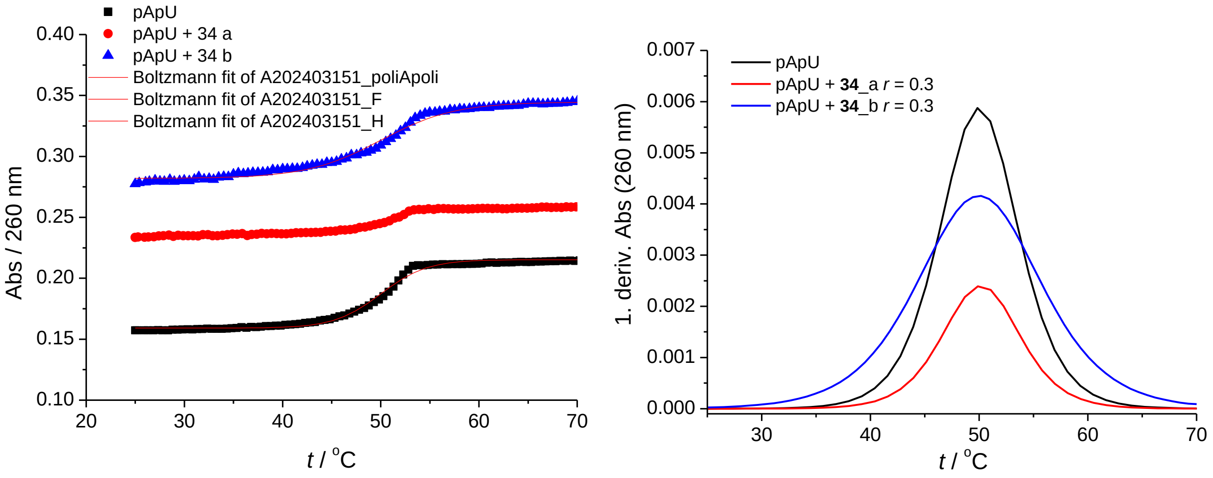

Figure S17. a) Denaturation of **pApU** upon addition of r = 0.3 ([compound/ [polynucleotide]) of **34** at pH 7.0 (buffer sodium cacodylate, I = 0.05 M), red lines denote fitting of experimental data to sigmoidal eq. by Origin 7.5; b) The first derivation of absorbance (fitted to sigmoidal eq.) on temperature dependence.

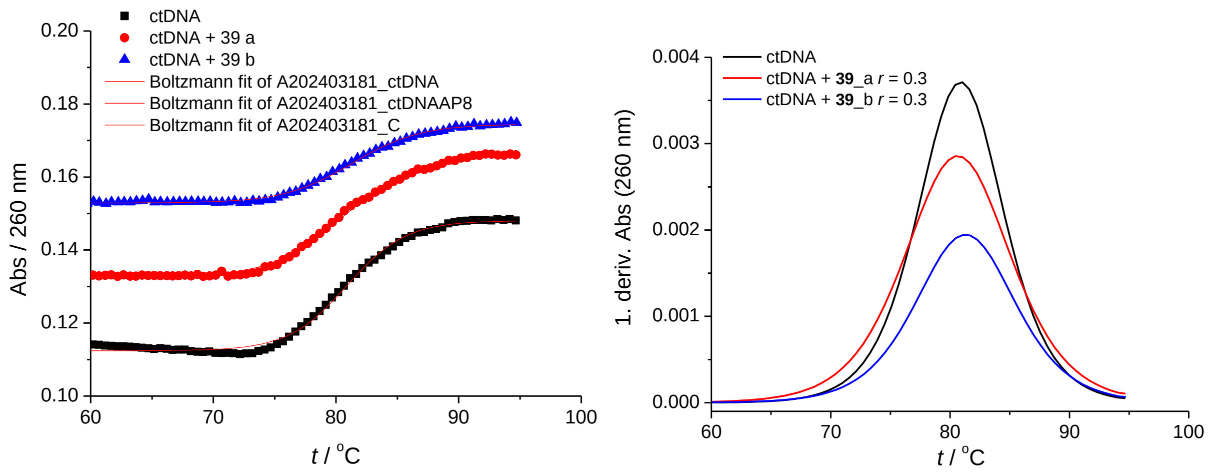

Figure S18. a) Denaturation of **ctDNA** upon addition of $r = 0.3$ ([compound/ [polynucleotide]) of **39** at pH 7.0 (buffer sodium cacodylate, $I = 0.05$ M), red lines denote fitting of experimental data to sigmoidal eq. by Origin 7.5; b) The first derivation of absorbance (fitted to sigmoidal eq.) on temperature dependence.

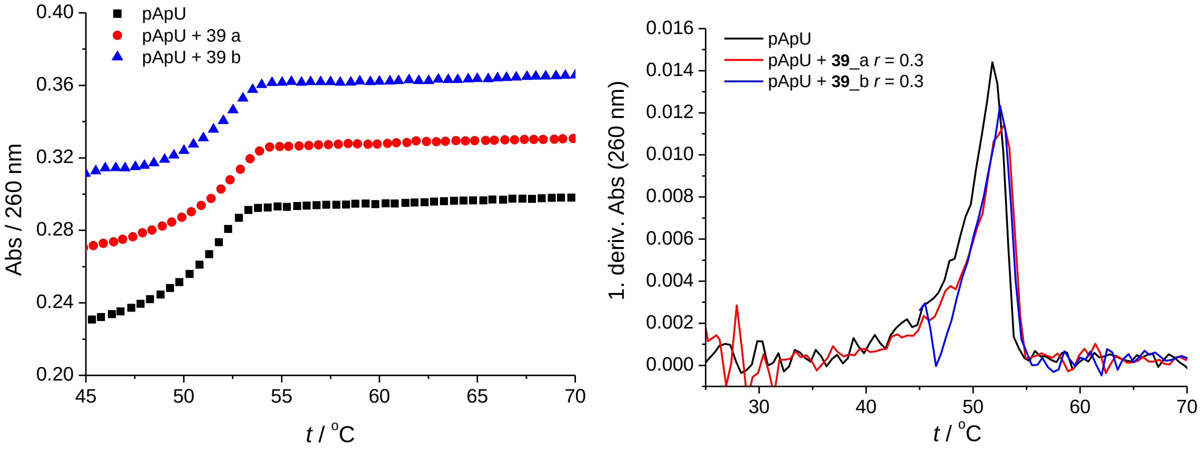

Figure S19. a) Denaturation of **pApU** upon addition of $r = 0.3$ ([compound/ [polynucleotide]) of **39** at pH 7.0 (buffer sodium cacodylate, $I = 0.05$ M), b) The first derivation of absorbance on temperature dependence.

---

[1] W. Saenger, Principles of Nucleic Acid Structure; Springer-Verlag: New York, USA, 1983.

[2] C. R. Cantor, P. R. Schimmel; Biophysical Chemistry, WH Freeman and Co.: San Francisco, USA, 1980; pp. 1109–1181.

[3] A. Rodger A., B. Norden, In *Circular Dichroism and Linear Dichroism*; Oxford University Press, New York, USA, 1997, Chapter 2.
